# Physical activity and sedentary behaviour in the Middle East and North Africa: An overview of systematic reviews and meta-analysis

**DOI:** 10.1038/s41598-020-66163-x

**Published:** 2020-06-09

**Authors:** Sonia Chaabane, Karima Chaabna, Amit Abraham, Ravinder Mamtani, Sohaila Cheema

**Affiliations:** Institute for Population Health, Weill Cornell Medicine-Qatar, Doha, Qatar

**Keywords:** Epidemiology, Risk factors

## Abstract

To support the global strategy to reduce risk factors for obesity, we synthesized the evidence on physical activity (PA) and sedentary behaviour in the Middle East and North Africa (MENA) region. Our systematic overview included seven systematic reviews reporting 229 primary studies. The meta-analysis included 125 prevalence measures from 20 MENA countries. After 2000, 50.8% of adults (ranging from 13.2% in Sudan to 94.9% in Jordan) and 25.6% of youth (ranging from 8.3% in Egypt to 51.0% in Lebanon) were sufficiently active. Limited data on PA behaviours is available for MENA countries, with the exception of Gulf Cooperation Council countries. The meta-regression identified gender and geographical coverage among youth, and the PA measurement as predictors of PA prevalence for both adults and youth. Our analysis suggests a significant PA prevalence increase among adults over the last two decades. The inconsistency in sedentary behaviour measurement is related to the absence of standardized guidelines for its quantification and interpretation. The global epidemic of insufficient PA is prevalent in MENA. Lower PA participation among youth and specifically females should be addressed by focused lifestyle interventions. The recognition of sedentary behaviour as a public health issue in the region remains unclear. Additional data on PA behaviours is needed from low- and middle-income countries in the region.

## Introduction

Non-communicable diseases (NCDs) kill 41 million people worldwide each year – equivalent to 71% of all deaths^1^. The Middle East and North Africa (MENA) region has one of the highest rates of NCDs in the world. In 2017, the region reported the second highest prevalence of diabetes in the world (10.8%)^2^ and is recording a rapid increase in obesity^3,4^. Insufficient physical activity (PA) and sedentary behaviour are key risk factors for obesity and other NCDs^5–15^ leading to premature mortality^10,11,16–18^. It has been suggested that PA has the potential to effectively control and reduce the burden of obesity during the various phases of human development^19^. Regular PA can also improve self-esteem, cognitive performance, and academic achievement in young people^7,20,21^ and is positively related to cardiorespiratory and metabolic health^6^. Recently, sedentary behaviour has received global attention as prolonged sedentary time is associated with an increased risk of chronic disease and an increase in all-cause mortality, regardless of individuals meeting the recommended levels of PA^13,15^.

The World Health Organization (WHO) and the Global Observatory for Physical Activity (GoPA) are targeting a relative reduction of 10% in the global prevalence of physical inactivity among adults by 2025^22,23^. Currently, one of the most pressing needs to improve population health is to develop appropriate policies and implement interventions to address the global pandemic of physical inactivity^24,25^. However, to support this action, country-level evidence on PA behaviour in various population groups is essential. Both regional-and country-level data contribute to the continuous surveillance of PA participation and are essential to track progress towards the global PA target.

In the MENA region, the proportion of the population not engaging in sufficient levels of PA (as per the recommendation of standardized international guidelines) remains unclear. Recent reports indicate physical inactivity prevalence measures of 32.8% for adults across the MENA and Central Asia regions^26^ and 78.4% in boys and 84.4% in girls globally^25^. However, gender-stratified measures in adults and youth are needed to develop evidence-based interventions informed by local data.

The aim of our study is to: 1) synthesize evidence from published systematic reviews (SRs) on PA behaviour in MENA countries, 2) quantify country-specific PA prevalence measures and assess demographic variations among youth and the general adult population within the region, 3) summarize measurement variations of PA and sedentary behaviour in the region, and 4) identify research gaps and provide specific recommendations pertaining to PA for the region. We tested the following null hypotheses that there is no difference in the PA prevalence between males and females, before and after 2000, and among nationals and non-nationals in the Gulf Cooperation Council countries (GCC).

## Methods

We conducted a systematic overview of published SRs on PA and sedentary behaviour in the MENA region. The current review is a co-product of a protocol planned for a systematic overview reporting the grey literature in systematic reviews on population health in the Middle East and North Africa (PROSPERO registration number CRD42017076736)^27,28^. This manuscript follows the Preferred Reporting Items for Systematic Reviews and Meta-Analyses (PRISMA) guidelines (Supplementary, Table S1)^29^, and the Preferred Reporting Items for Overviews of Systematic Reviews (PRIO-harms) tool (Supplementary, Table S2).

### Search strategy and selection criteria

A broad search strategy was developed to systematically identify any type of review on all health issues in any MENA country. Search terms related to countries’ names, MENA populations’ names, and MENA sub-regions’ names, such as North Africa, East Africa, and the Middle East, were used. No restrictions to a specific health condition or intervention and language of publication were applied at this stage. The full search strategy with search criteria is available in the published overview protocol^27^. Two independent reviewers (AA and HA) systematically searched the Medical Literature Analysis and Retrieval System Online (MEDLINE) through the search engine PubMed. We included publications since 2008 – the publication year of the first version of the Cochrane Handbook for Systematic Reviews of Interventions^30^ up to February 21, 2017. Additionally, we also searched grey and non-grey literature sources with no date or language restriction including Google Scholar, Epistemonikos, ProQuest, OpenGrey, Bioline International, E-Marefa, ALMANHAL platform, governmental websites of all MENA countries, and the WHO website for systematic reviews relevant to our topic. The literature search was then updated to identify recent SRs published up to November 2019.

A systematic review (SR) was defined as a literature review that has explicitly used a systematic literature search of at least one electronic database to identify all studies that meet pre-defined eligibility criteria along with a study selection^30^. Reviews not reporting a systematic methodology, such as narrative reviews, were excluded.

Based on the relevance of grey literature in the region when studying population health outcomes^27,31^, we included MENA countries where Arabic, English, French, and/or Urdu are the primary official languages and/or the medium of instruction in the colleges/universities. These languages are also those spoken by the authors of this overview (see the overview’s protocol^27^). The 20 MENA countries included are Algeria, Bahrain, Djibouti, Egypt, Iraq, Jordan, Kuwait, Lebanon, Libya, Morocco, Oman, Pakistan, Palestine, Qatar, Saudi Arabia, Sudan, Syria, Tunisia, the United Arab Emirates (UAE), and Yemen.

### Data screening

Records were downloaded into Endnote (version X8.2), and duplicates were removed. Using Rayyan software^32^, two independent reviewers (AA and HA) conducted the multi-stage screening following a standard process. Discrepancies in the inclusion of SRs were resolved through discussion, with the involvement of a third reviewer (AA, HA, and KC).

Retrieved SRs were then categorized based on the reported population health outcome. For the purpose of this overview, we included any SR reporting measurable PA-related outcomes including physical activity or inactivity prevalence and/or sedentary behaviour pertaining to MENA population of any age group residing in a MENA country.

### Data extraction

Data extraction was conducted by SC1 and checked for accuracy by KC. Extracted data included characteristics of the included SRs as well as the primary studies and were matched to PICOTS items (Population, Outcomes, Time of the study and Setting; Control and Intervention were not applicable to our overview question). From each included SR, the following characteristics were extracted: literature search terms and time period, geographical coverage, data literature sources, MENA countries with retrieved data, along with the number of included studies, inclusion and exclusion criteria, targeted review population, and reported PA-related outcomes. From each primary study included in a SR, the following characteristics were collected: study design and sample size, sampling method, study setting, years of data collection, population characteristics (type, age, gender, proportion of males and females, and response rate), and all PA-related outcomes (definition, measurement tool/administration, prevalence, and barriers to PA). In case of discordance between the reported data by the SR and data available in the primary study, the latter was retained. A consensus meeting with SC1, KC, and SC2 was held to resolve any disagreements.

In order to assess the methodological quality of the included primary studies and conduct the quantitative analyses, any relevant study characteristics not reported by the SR were extracted from the primary study publication. In addition, any additional data on PA-related outcomes from a MENA country found in an included primary study but not reported by the SR (usually not part of the objectives) was extracted and reported.

### Qualitative synthesis

The qualitative synthesis was done at two levels:

#### Qualitative synthesis of the SRs’ data

A summary of the geographical coverage, the methodology used by each SR, the main conclusions on reported outcomes, limitations and strengths, research gaps, and recommendations from each SR were synthesized in Supplementary, Tables S3 and S4. A synthesis of the methodological quality of the included SRs and our own overview was conducted.

#### Qualitative synthesis of the primary studies data

The characteristics of primary studies that reported PA in the SRs were synthesized. These included physical inactivity prevalence measures and/or sedentary behaviour data among youth (≤19-year-old) and adults (>19-years-old). If these were not reported, the prevalence of the outcome among the total study population (males and/or females) was calculated using raw data available in the primary study.

We synthesized the measurement tools and definitions of PA, physical inactivity and sedentary behaviour used by the primary studies as well as the characteristics of the study population and the official primary language of the MENA country where the study was conducted. These characteristics were contrasted with the population and linguistic validation parameters of each used tool. A measurement tool was considered to be validated in a specific population or language if a validation record in the same population or language was retrieved in the literature. The objective of this synthesis was to review the variability in the outcome measurements and appropriateness of their use in the primary study population^33^.

### Quantitative synthesis

By definition, a participant who does not meet the PA recommendations of 150 min of moderate physical activity/week or equivalent for adults and 60 min of moderate to vigorous PA daily for youth or equivalent is considered physically inactive and vice versa. Hence, in all included studies reporting physical inactivity outcomes, participants who did not meet the physical inactivity criteria were considered meeting the recommended level for PA. These measures were included in the meta-analysis as a PA-prevalence measure as long as they were not overlapping with other included data in the meta-analysis. The “poor level”, “mild level” or “low score” of PA was reported in some studies where there was a low PA participation level (lower than standard thresholds)^34–36^. These PA prevalence measures were considered as physical inactivity prevalence measures. All physical inactivity prevalence measures along with reasons for conversion or non-conversion to a PA prevalence measures are listed in Supplementary, Table S5. When two levels of PA (moderate and vigorous) were reported in the same study^34,36^, both levels were merged to one level of PA.

A meta-analysis of PA prevalence (proportion of participants meeting recommended levels of PA as defined by the primary studies) was conducted for the MENA countries for which data was available. The GCC subregion includes the following countries: Bahrain, Qatar, Oman, Kuwait, Saudi Arabia, and UAE. The PA-prevalence measures stratified by gender and age were prioritized for the inclusion in the meta-analysis rather than the overall measures for the entire study population. As there is no consensus on the standard definition and a threshold to quantify sedentary behaviour, no meta-analysis was conducted for this outcome. A publication of a primary study included in more than one SR and/or publications with overlapping data points were included once and all the replicates were excluded from the meta-analysis. A list of primary studies excluded from the meta-analysis with reasons is detailed in Supplementary, Table S6.

A subgroup meta-analysis of PA participation by gender (males and females) and year of data collection (before 2000 and after 2000) was then conducted for the MENA and GCC regions and for each MENA country with available data. A high proportion of the population residing in the GCC is non-national^37^; while in the other MENA countries, the populations are predominantly nationals. Therefore, meta-analyses according to the type of population (national population and mixed populations of nationals and non-nationals) were relevant for GCC countries only.

The threshold of data collection time was established at the year 2000 based on the demographic, socioeconomic, migration background in the GCC countries^37,38^, urbanization, and lifestyle transition observed in many MENA countries by the beginning of the 21^st^ century^39–42^. For the PA prevalence measures where the year of data collection was missing, we considered this as prior to the publication year and the prevalence measure was then classified accordingly for the analyses.

If not reported, the total or strata sample sizes were calculated based on the percentage of males and females in the sample. Random effects modelling was used to conduct the meta-analyses. We generated forest plots using the ‘meta/metaprop’ package in R (version 3.5.0) and inspected them visually to assess the variability of prevalence estimates across subgroups. Furthermore, the heterogeneity of the prevalence estimates was assessed using: (1) the I^2^ statistic, (2) the Cochran’s Q statistic, and (3) the Cochran’s Q between-subgroups. The I^2^ statistic was used to describe the percentage of variability in the prevalence estimates resulting from the between-study heterogeneity rather than chance^43^. The statistical significance (p-value < 0.05) of the Cochran’s Q statistic was used to test for evidence of the overall heterogeneity between prevalence estimates^44^. The statistical significance (p-value < 0.05) of the Cochran’s Q between-subgroups statistic was used to test for differences between prevalence estimates across subgroups^45^. The prediction interval estimated the 95% interval in which the true PA prevalence in a new PA prevalence study will lie.

To continue exploring the statistical heterogeneity, univariable random-effects meta-regression analyses were conducted to identify associations of higher PA prevalence and sources of between-study heterogeneity. Associations were assessed using odds ratios (ORs), 95% Confidence Intervals (CIs), and t-tests. Relevant covariates were specified *a priori* and included in the models separately: gender (males vs. females), years of data collection (after 2000 vs. before 2000), geographical coverage (country level vs. local level), and PA measurement tool (standard tools vs. non-standard tools). Standard PA measurement tools included: 1) the pedometer, accelerometer and continuous heart rate monitor (gold standard tool), and 2) validated PA questionnaires among adults and youth included the International Physical Activity Questionnaire (IPAQ), the Global Physical Activity Questionnaire (GPAQ), the Arab Teens Lifestyle Student Questionnaire (ATLS), the Nurses’ Health Study II Activity and Inactivity Questionnaire (NHS II-PAQ), “How physically active are you” questionnaire, the Health Behaviour in School-aged Children Survey (HBSC), and the Adolescent Physical Activity Measure Questionnaire (PACE+)^46^. Any other used measurement tool that was not included in the National Institutes of Health (NIH) Database of Standardized Questionnaires About Walking & Bicycling or does not reference to a published validation study of the used tool was considered as non-validated measurement tool.

Factors with *p*-value <0.05 in the univariate meta-regression were considered as statistically significant covariates. These covariates were explored separately in the adult and youth MENA general populations. Meta-analyses and meta-regressions were conducted using the ‘meta/metareg’ package in the R software (version 3.5.0).

### Methodological quality assessment

The methodological quality of the included SRs and primary studies was assessed by two independent reviewers.

The AMSTAR measurement tool^47^ was used by two independent reviewers (SC1 and AA) to perform the quality assessment of the included SRs. Discrepancies were resolved through discussion, with the involvement of a third reviewer (SC1, AA, and KC) when necessary. The methodological quality of the included SRs was then discussed according to the 11 criteria of the AMSTAR checklist^48^.

At primary study-level, an adapted quality assessment checklist was developed based on the PICOTS framework^49^ and other published tools utilized for similar contexts^50–55^. Our quality assessment checklist included criteria related to outcome definition, outcome measurement, population characteristics, and sampling method since the quality of PA and sedentary behavior measures are directly related to the study’s quantification method and its population characteristics. We utilized the guidelines of the World Health Organization (WHO), the Physical Activity Guidelines Advisory Committee, the Centers for Disease Control and Prevention (CDC), the American College of Sports Medicine, and the Sedentary Behavior Research Network (SBRN) to establish a list of standard definitions and valid methods utilized for PA-participation assessment. The detailed checklist and the definition of each criterion and the scoring system is presented in Supplementary, Table S7. Each of the seven included criteria was rated with a maximum score of three (0 = “Not defined”, 3 = “Clearly defined and appropriate”). Hence, a maximum quality score of 21 can be reported for one assessed PA-related outcome. The PA-related outcomes in each population subgroup were evaluated and scored by SC1 and checked by KC. Any discrepancies were resolved by discussion with the involvement of a third reviewer (SC1, KC, and SC2). The higher the quality score that was reported, the better the methodological quality of the reported outcome.

## Results

**Figure 1:**
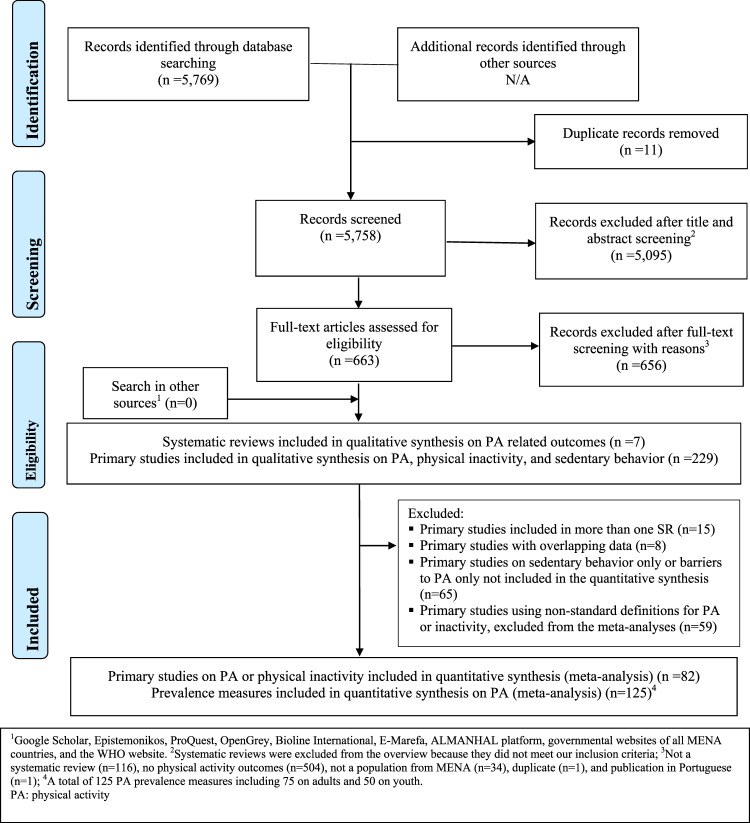
PRISMA 2009 flowchart of the systematic reviews inclusion.

A total of seven SRs and 229 primary studies on the epidemiology of PA, physical inactivity, and/or sedentary behaviour in at least one of the 20 selected MENA countries were included in the qualitative synthesis of this overview (Fig. 1). After exclusion of primary studies included in more than one SR (n = 15), primary studies with overlapping data (n = 8), primary studies reporting only sedentary behaviour data or barriers to PA data (n = 65), and those using non-standard definitions for PA or inactivity, excluded from the meta-analyses (n = 59), a total of 82 studies, including 125 PA prevalence measures were considered for the quantitative analyses.

### Characteristics of the included systematic reviews

The seven SRs included in our overview are described in Supplementary, Table S3. The summary of qualitative results, strengths and limitations, research gaps, and recommendations are synthesized in Supplementary, Table S4. PA-related outcomes were found for all the 20 MENA countries. The physical inactivity and sedentary behaviour measures were the primary outcomes in three SRs^54^. The other SRs reported these outcomes only if included in the primary studies on PA. No SR restricted the primary study inclusion to one specific definition of PA. All the identified data was based on the general population. Of note, only Yammine *et al*.^56^ conducted a meta-analysis of prevalence measures of different levels of PA among UAE adolescents. All the included SRs excluded clinical populations and three SR^53,57,58^ excluded primary studies based on their methodological quality of sampling and measurement methodology.

### Methodological quality assessment of the included systematic reviews and primary studies

No SR reported the list of excluded studies, assessed publication bias, or included the source of funding for the SR itself and of the included primary studies, as per the AMSTAR recommendations^48^ (Table 1**)**. Two SRs^56,58^ searched for grey literature sources (Table 1**)**.

**Table 1:** Quality assessment of the included systematic reviews and the actual overview using AMSTAR checklist.

Systematic review	1.Was an ‘a priori’ design provided?	2.Was there duplicate study selection and data extraction?	3.Was a comprehensive literature search performed?	4.Was the status of publication (i.e. grey literature) used as an inclusion criterion?	5.Was a list of studies (included and excluded) provided?	6.Were the characteristics of the included studies provided?	7.Was the scientific quality of the included studies assessed and documented?	8.Was the scientific quality of the included studies used appropriately in formulating conclusions?	9.Were the methods used to combine the findings of studies appropriate?	10.Was the likelihood of publication bias assessed?	11.Was the conflict of interest included? †
Sisson, 2008^88^	No	No	No	No	No	Yes	No	No	N/A	N/A	No
Mabry, 2010^53^	No	No	Yes	No	No	Yes	Yes	Yes	N/A	N/A	No
Ranasinghe, 2013^54^	No	Yes	Yes	No	No	Yes	Yes	No	N/A	N/A	No
Yammine, 2016^56^	No	No	Yes	Yes	No	Yes	No	No	Yes	No	No
Mabry, 2016^52^	No	Yes	No	No	No	Yes	Yes	Yes	N/A	N/A	No
Al-Hazzaa, 2018^57^	No	No	Yes	No	No	Yes	No	No	N/A	N/A	No
Sharara, 2018^58^	Yes	No	Yes	Yes	No	Yes	No	No	N/A	N/A	Yes
Current Overview	Yes	Yes	Yes	N/A	Yes	Yes	Yes	Yes	Yes	No	Yes

Notes: We used the AMSTAR checklist with additional notes made by Michelle Weir, Julia Worswick, and Carolyn Wayne based on conversations with Bev Shea and/or Jeremy Grimshaw in June and October 2008 and July and September 2010. Available on https://amstar.ca/docs/AMSTARguideline.pdf

The absence of a statement regarding any criteria of the quality assessment was considered as not done. The unit of analysis for the systematic reviews is the study. The unit of analysis for the actual overview is the systematic review.

Abbreviations: N/A: Not applicable;

† To get a ‘yes’ for the included SRs, the conflict of interest should be clearly acknowledged for the SR and the included original studies.

To get a ‘yes’ for the actual overview, the conflict of interest should be clearly acknowledged for the actual overview and all included SRs.

The checklist used for the quality assessment of the included studies is presented in Supplementary, Table S5. The quality assessment scores of each reported PA related outcome are reported in Supplementary, Tables S8–S11. The methodological quality scores varied between 11 and 20 out of a maximum score of 21. Most of the reported outcomes had a quality score closer to the upper score limit. Most of the primary studies were cross-sectional clearly describing the population characteristics and study setting, used PA definition consistent with international recommendations, utilized validated measurement tools, used probability-based sampling method, had an equal male and female ratio, and country-level coverage.

### Overview of studies with physical activity data

Forty-seven primary studies with 75 PA prevalence measures on adults and thirty-five primary studies with 50 PA prevalence measures on youth were included in the meta-analysis. The study characteristics and PA prevalence measures among the adult and youth population are summarized in Supplementary, Tables S8 and S9.

For both populations, limited data on PA was reported in MENA countries with the exception of the GCC countries (Bahrain, Kuwait, Oman, Qatar, Saudi Arabia and UAE). In most included studies pertaining to GCC countries, the distinction between the national and non-national population was unclear. A wide range of PA prevalence measures were reported for the MENA region. A higher PA prevalence measure among males than females was observed for all included MENA countries except among adults in Lebanon and Jordan. Considering all retrieved data (before and after 2000) among adult males, the highest pooled prevalence measures of sufficient PA participation was found in Jordan [94.2% (95% CI = 93.1–95.2%) and the lowest in Bahrain [23.1% (95% CI = 20.9–25.5%)]. Among adult females, the highest pooled prevalence measures of sufficient PA participation were found in Jordan [95.5% (95% CI = 94.4–96.4%)], and the lowest in Bahrain 1.3% (95% CI = 0.7–2.3%) (Table 2).

**Table 2:** Meta-analysis of physical activity prevalence in MENA countries among the adult general population.

Studied subgroups	Years of data collection	Number of prevalence measures	Total sample size	Prevalence range (%)^a^	Effect size		Subgroup comparison	Heterogeneity between studies		
					Weighted average prevalence (%)^b^	95% CI (%)	Q between-subgroups test *p*-value	Cochrane Q test’s *p*-value	I² (confidence limits)	95% Prediction interval (%)
**MENA**										
All*	1995–<2017	75	124,702	1.3–95.5	47.9	40.8–55.0	N/A	0.0000	99.8% (99.8–99.8%)	1.8–96.9
Males^c^	1995–<2015	29	49,343	6.1–94.2	54.1	42.3–65.7	0.2286	0.0000	99.9% (99.8–99.9%)	2.1–99.7
Females^c^	1995–<2016	32	44,167	1.3–95.5	43.6	31.7–55.9		0.0000	99.8% (99.8–99.9%)	0.0–98.8
<2000	1995–<2000	4	19,593	1.3–23.1	6.2	1.7–13.3	<0.0001	<0.0001	99.6% (99.4–99.7%)	0.0–56.0
>2000	2002–<2017	71	105,109	5.2–95.5	50.8	45.6–56.0		0.0000	99.7% (99.6–99.7%)	11.5–89.6
**GCC**										
All*	1995–2017	49	72,489	1.3–69.2	41.7	33.7–50	N/A	0.0000	99.8% (99.8–99.8%)	0.9–93.1
Males^c^	1995–<2015	19	34,270	6.1–69.2	47	33.1–61.2	0.3836	0.0000	99.8% (99.8–99.9%)	0.4–98.3
Females^c^	1995–<2016	22	27,256	1.3–65.7	38.6	26.6–51.3		0.0000	99.8% (99.7–99.8%)	0.0–93.9
<2000	1995–2000	4	19,593	1.3–23.1	6.2	1.7–13.3	<0.0001	<0.0001	99.6% (99.4–99.7%)	0.0–56.0
>2000	2000–2017	45	52,896	13.3–69.2	45.7	40.5–51.0		0.0000	99.3% (99.2–99.4%)	13.8–79.8
National population	1995–<2014	23	44,711	1.3–68.0	36.8	25.9–48.4	0.1737	0.0000	99.8% (99.8–99.8%)	0.1–90.8
National and non-national populations	<1996–<2016	26	27,778	13.3–69.2	46.2	38.9–53.6		0.0000	99.2% (99.1–99.3%)	11.6–83.1
**Saudi Arabia**										
All*	1995–2017	30	52,221	1.9–69.2	39.9	28.8–51.5	N/A	0.0000	99.8% (99.8–99.9%)	0.0–95.7
Males^c^	1995–<2015	11	25,990	6.1–69.2	42.9	22.7–64.4	0.8969	0.0000	99.9% (99.9–99.9%)	0.0–100.0
Females^c^	1995–<2016	14	18,145	1.9–65.7	41	24.5–58.6		0.0000	99.8% (99.7–99.8%)	0.0–98.8
<2000	1995–2000	2	17,395	1.9–6.1	3.7	0.7–9.0	<0.0001	<0.0001	99.5% (99.2–99.7%)	N/A
>2000	2003–2017	28	34,826	13.3–69.2	43.3	36.0–50.8		0.0000	99.4% (99.4–99.5%)	8.7–82.2
National population	1995–<2014	10	30,296	1.9–65.7	29.5	16.5–44.4	0.0712	0.0000	99.8% (99.8–99.9%)	0.0–85.2
National and non-national populations	2005–2017	20	21,925	13.3–69.2	45.2	36.5–54.1		0.0000	99.3% (99.1–99.4%)	9.3–84.4
**UAE**										
All*	2000–2003	5	2,459	43.2–67.4	57.8	49.1–66.3	N/A	< 0.0001	94.4% (89.6–96.9%)	25.2–87.0
Males^c^	2002–2003	2	1,646	62.1–66.5	64.3	59.9–68.6	<0.0001	0.0621	71.3% (0.0–93.5%)	N/A
Females^c^	2002–2003	2	638	43.2–49.4	46.3	40.3–52.4		0.1165	59.4% (0.0%–90.5%)	N/A
<2000	N/A	0	N/A	N/A	N/A	N/A	N/A	N/A	N/A	N/A
>2000	2000–2003	5	2,459	43.2–67.4	57.8	49.1–66.3		<0.0001	94.4% (89.6–96.9%)	25.2–87.0
National population	2003	2	1,180	49.4–66.5	58.2	41.2–74.2	0.9558	<0.0001	96.5% (90.5–98.7%)	N/A
National and non-national populations	2000–2003	3	1,279	43.2–67.4	57.6	44.0–70.6		<0.0001	94.9% (88.4–97.7%)	0.0–100.0
**Oman**										
All	2008	2	3,137	59.5–68.0	63.8	55.3–71.9	N/A	< 0.0001	95.9% (88.4–98.6%)	N/A
Males	2008	1	1,459	N/A	68.0	65.5–70.4	N/A	N/A	N/A	N/A
Females	2008	1	1,678	N/A	59.5	57.1–61.8		N/A	N/A	N/A
<2000	N/A	0	N/A	N/A	N/A	N/A	N/A	N/A	N/A	N/A
>2000	2008	2	3,137	59.5–68.0	63.8	55.3–71.9		<0.0001	95.9% (88.4–98.6%)	N/A
National population	2008	2	3,137	59.5–68.0	63.8	55.3–71.9	N/A	< 0.0001	95.9% (88.4–98.6%)	N/A
National and non-national populations	N/A	0	N/A	N/A	N/A	N/A		N/A	N/A	N/A
**Bahrain**										
All	1995–1996	2	2,198	1.3–23.1	9.3	0.0–40.2	N/A	< 0.0001	99.7% (99.5–99.8%)	N/A
Males	1995–1996	1	1,297	N/A	23.1	20.9–25.5		N/A	N/A	N/A
Females	1995–1996	1	901	N/A	1.3	0.7–2.3		N/A	N/A	N/A
<2000	1995–1996	2	2,198	1.3–23.1	9.3	0.0–40.2	N/A	< 0.0001	99.7% (99.5–99.8%)	N/A
>2000	N/A	0	N/A	N/A	N/A	N/A		N/A	N/A	N/A
National population	1995–1996	2	2,198	1.3–23.1	9.3	0.0–40.2	N/A	< 0.0001	99.7% (99.5–99.8%)	N/A
National and non-national populations	N/A	0	N/A	N/A	N/A	N/A		N/A	N/A	N/A
**Qatar**										
All*	2003–<2015	5	4,382	39.5–62.6	49.3	41.5–57.1	N/A	< 0.0001	96.3% (93.7–97.8%)	20.6–78.2
Males^c^	2003	2	1537	49.1–62.6	56.0	42.7–68.9	0.0782	<0.0001	96.0% (88.9–98.6%)	N/A
Females^c^	2003–2012	2	2,113	39.5–45.8	42.8	36.7–49		0.0058	86.8% (48.2% 96.7%)	N/A
<2000	N/A	0	N/A	N/A	N/A	N/A	N/A	N/A	N/A	N/A
>2000	2003–<2015	5	4,382	39.5–62.6	49.3	41.5–57.1		<0.0001	96.3% (93.7–97.8%)	20.6–78.2
National population	2003–2012	4	3,650	39.5–62.6	49.3	39.4–59.3	0.9982	<0.0001	97.2% (95.1–98.4%)	8.3–90.9
National and non-national populations	<2015	1	732	N/A	49.3	45.7–52.9		N/A	N/A	N/A
**Kuwait**										
All*	2006–2014	5	8,092	27.2–48.6	36.5	28.7–44.7	N/A	< 0.0001	98.2% (97.3–98.9%)	9.8–68.8
Males^c^	2006–2014	2	2341	42.1–48.6	45.4	39.1–51.9	<0.0001	0.0020	89.6% (61.2%–97.2%)	N/A
Females^c^	2006–2014	2	3,781	27.2–28.4	27.7	26.2–29.1		0.4233	0.0%	N/A
<2000	N/A	0	N/A	N/A	N/A	N/A	N/A	N/A	N/A	N/A
>2000	2006–2014	5	8,092	27.2–48.6	36.5	28.7–44.7		<0.0001	98.2% (97.3–98.9%)	9.8–68.8
National population	2006–<2013	3	4,250	28.4–42.1	35.7	28.5–43.2	0.8676	<0.0001	96.0% (91.4–98.1%)	0.0–100.0
National and non-national populations	2014	2	3,842	27.2–48.6	37.6	18.3–59.3		N/A	N/A	N/A
**Pakistan**										
All*	2002–<2010	4	6,144	30.2–87.2	61.0	41.4–78.9	N/A	< 0.0001	99.5% (99.3–99.6%)	0.0–100.0
Males	2002–<2010	2	3,114	47.9–87.2	69.5	27.7–97.7	0.5557	<0.0001	99.5% (99.2–99.7%)	N/A
Females	2002–<2010	2	3,030	30.2–72.7	51.7	13.2–89.1		<0.0001	99.4% (99.0–99.7%)	N/A
<2000	N/A	0	N/A	N/A	N/A	N/A	N/A	N/A	N/A	N/A
>2000	2002–<2010	4	6,144	30.2–87.2	61.0	41.4–78.9		<0.0001	99.5% (99.3–99.6%)	0.0–100.0
**Tunisia**										
All*	2002–2009	3	6,212	55.6–89.0	76.8	55.9–92.5	N/A	< 0.0001	99.7% (99.6–99.8%)	0.0–100.0
Males^c^	2002–2003	1	2,149	N/A	89.0	87.6–90.3	N/A	N/A	N/A	N/A
Females^c^	2002–2003	1	2,183	N/A	81.8	80.1–83.4		N/A	N/A	N/A
<2000	N/A	0	N/A	N/A	N/A	N/A	N/A	N/A	N/A	N/A
>2000	2002–2009	3	6,212	55.6–89.0	76.8	55.9–92.5		<0.0001	99.7% (99.6–99.8%)	0.0–100.0
**Lebanon**										
All*	2000–2014	4	5,461	24.0–59.8	41.5	27.0–56.7	N/A	< 0.0001	99.2% (98.9–99.5%)	0.0–98.6
Males^c^	2008–2009	1	893	N/A	47.8	44.5–51.2	N/A	N/A	N/A	N/A
Females^c^	2008–2009	1	1,089	N/A	59.8	56.8–62.7	N/A	N/A	N/A	N/A
<2000	N/A	0	N/A	N/A	N/A	N/A	N/A	N/A	N/A	N/A
>2000	2000–2014	4	5,461	24.0–59.8	41.5	27.0–56.7	N/A	< 0.0001	99.2% (98.9–99.5%)	0.0–98.6
**Morocco**										
All*	2008	3	5,233	54.6–83.5	69.3	50.6–85.2	N/A	< 0.0001	99.5% (99.2–99.6%)	0.0–100.0
Males^c^	2008	1	1,359	N/A	67.6	65.1–70.1	N/A	N/A	N/A	N/A
Females^c^	2008	1	1,261		54.6	51.8–57.4		N/A	N/A	N/A
<2000	N/A	N/A	N/A	N/A	N/A	N/A	N/A	N/A	N/A	N/A
>2000	2008	3	5,233	54.6–83.5	69.3	50.6–85.2		<0.0001	99.5% (99.2–99.6%)	0.0–100.0
**Egypt**										
All*	2012	1	5,300	N/A	67.9	66.6–69.2	N/A	N/A	N/A	N/A
Males	N/A	N/A	N/A	N/A	N/A	N/A	N/A	N/A	N/A	N/A
Females	N/A	N/A	N/A	N/A	N/A	N/A	N/A	N/A	N/A	N/A
<2000	N/A	N/A	N/A	N/A	N/A	N/A	N/A	N/A	N/A	N/A
>2000	2012	1	5,300	N/A	67.9	66.6–69.2		N/A	N/A	N/A
**Palestine**										
All*	2010–2011	1	6,957	N/A	53.5	52.3–54.7	N/A	N/A	N/A	N/A
Males	N/A	N/A	N/A	N/A	N/A	N/A	N/A	N/A	N/A	N/A
Females	N/A	N/A	N/A	N/A	N/A	N/A	N/A	N/A	N/A	N/A
<2000	N/A	N/A	N/A	N/A	N/A	N/A	N/A	N/A	N/A	N/A
>2000	2010–2011	1	6,957	N/A	53.5	52.3–54.7	N/A	N/A	N/A	N/A
**Sudan**										
All*	2005–2006	2	1,573	5.2–24.2	13.2	0.8–36.7	N/A	N/A	N/A	N/A
Males	2005–2006	1	652	N/A	24.2	21.0–27.7	N/A	N/A	N/A	N/A
Females	2005–2006	1	921	N/A	5.2	3.9–6.9	N/A	N/A	N/A	N/A
<2000	N/A	N/A	N/A	N/A	N/A	N/A	N/A	N/A	N/A	N/A
>2000	2005–2006	2	1,573	5.2–24.2	13.2	0.8–36.7	N/A	N/A	N/A	N/A
**Algeria**										
All*	2003	2	4,101	54.2–67.5	61.0	47.7–73.5	N/A	N/A	N/A	N/A
Males	2003	1	1,599	N/A	67.5	65.2–69.8	N/A	N/A	N/A	N/A
Females	2003	1	2,502	N/A	54.2	52.3–56.2		N/A	N/A	N/A
<2000	N/A	0	N/A	N/A	N/A	N/A	N/A	N/A	N/A	N/A
>2000	2003	2	4,101	54.2–67.5	61.0	47.7–73.5	N/A	N/A	N/A	N/A
**Libya**										
All*	2009	2	3,590	N/A	56.2	40.8–71.1	N/A	N/A	N/A	N/A
Males	2009	1	1,800	N/A	64.0	61.7–66.2	N/A	N/A	N/A	N/A
Females	2009	1	1,790	N/A	48.3	46.0–50.7		N/A	N/A	N/A
<2000	N/A	0	N/A	N/A	N/A	N/A	N/A	N/A	N/A	N/A
>2000	2009	2	3,590	N/A	56.2	40.8–71.1	N/A	N/A	N/A	N/A
**Jordan**										
All*	2007	2	3,654	N/A	94.9	93.5–96.1	N/A	N/A	N/A	N/A
Males	2007	1	1,939	N/A	94.2	93.1–95.2	N/A	N/A	N/A	N/A
Females	2007	1	1,715	N/A	95.5	94.4–96.4	N/A	N/A	N/A	N/A
<2000	N/A	0	N/A	N/A	N/A	N/A	N/A	N/A	N/A	N/A
>2000	2007	2	3,654	N/A	94.9	93.5–96.1	N/A	N/A	N/A	N/A
**Iraq**										
All*	2015	2	3,988	N/A	52.6	28.4–76.2	N/A	N/A	N/A	N/A
Males	2015	1	1,568	N/A	65.1	62.7–67.5	N/A	N/A	N/A	N/A
Females	2015	1	2,420	N/A	40.0	38.0–42.0	N/A	N/A	N/A	N/A
<2000	N/A	0	N/A	N/A	N/A	N/A	N/A	N/A	N/A	N/A
>2000	2015	2	3,988	N/A	52.5	28.4–76.2	N/A	N/A	N/A	N/A

Notes: Mean effect size reported as weighted average prevalence measure with their corresponding 95% confidence interval (CI); the *p-value* of the Cochran’s Q statistic is used to inform about the statistical significance of the heterogeneity in PA prevalence estimates within the group; the *p-value* of the Cochran’s Q between-subgroups statistic is used to inform about the statistical significance of the heterogeneity in PA prevalence estimates between the results of subgroups if applicable; the I^2^ is used to assess the magnitude of between-study variation that is due to differences in PA prevalence estimates across studies rather than chance; the prediction interval is used to estimate the 95% interval in which the true PA prevalence in a new PA prevalence study will lie. No meta-analyses according to the time of data collection were then performed. p-value <0.05 was considered statistically significant. Meta-analyses according to the type of population (nationals and non-national populations) were only relevant in the GCC countries as per the type of setting of the GCC population. Years of data collection were considered as prior to the publication year in studies where this information was missing. The prevalence measures were then classified accordantly for the meta-analyses. <2000: this analysis includes all studies where the data was collected before 2000. > 2000: this analysis includes all studies where the data was collected before 2000.

Symbols:

*Include converted data from physical inactivity to physical activity not overlapping with other included data.

^a^Stratified prevalence measures were used to conduct the meta-analyses.

^b^Weighted average prevalence measures were obtained using random-effect model.

^c^Excluded the studies with a mixed male and female population.

The highest pooled prevalence measures of sufficient PA participation among youth males was observed in Kuwait [70.4% (95% CI = 66.0–74.5%)] and the lowest in Egypt [14.0% (95% CI = 12.5–15.6%)]. The highest pooled prevalence measures of sufficient PA participation among youth females was observed in Kuwait 39.3% (95% CI = 34.7–44.0%) and the lowest in Egypt 4.0% (95% CI = 3.1–5.1. %) (Table 3**)**.

**Table 3:** Meta-analysis of physical activity prevalence in MENA countries among the youth general population.

Studied subgroups	Years of data collection	Number of prevalence measures	Total sample size	Prevalence range (%) ^a^	Effect size		Subgroup comparison	Heterogeneity between studies		
					Weighted average prevalence (%) ^b^	95% CI (%)	Q between-subgroups test *p*-value	Cochrane Q test’s *p*-value	I² (confidence limits)	95% Prediction interval (%)
**MENA**										
All*	<1993-<2018	50	78,915	4.0–79.9	25.4	21.8–29.1	N/A	0.0000	99.3% (99.2–99.3%)	4.9–54.7
Males^c^	<1993-<2015	19	20,811	14.0–79.9	37.1	28.8–45.8	<0.0001	0.0000	99.4% (99.3–99.5%)	5.2–77.7
Females^c^	2004-<2018	16	18,606	4.0–52.2	16.5	12.2–21.2		<0.0001	98.5% (98.2–98.8%)	2.1–40.4
<2000	<1993- <2000	1	212	N/A	15.1	10.6–20.6	0.0014	N/A	N/A	N/A
>2000	2002-<2018	49	78,703	4.0–79.9	25.6	22.0–29.4		0.0000	99.3% (99.2–99.4%)	5.0–55.0
GCC										
All*	<1993-<2018	25	34,323	4.0–79.9	33.3	26.7–40.3	N/A	0.0000	99.4% (99.4–99.5%)	5.1–71.0
Males^c^	<1993-<2015	12	12,522	15.1–79.9	48.9	36.7–61.2	0.0002	<0.0000	99.4% (99.3–99.5%)	7.1–91.7
Females^c^	2004-<2018	9	10,637	4.0–52.2	21.7	15.1–29.1		<0.0001	98.5% (98.0–98.9%)	2.6–52.1
<2000	<1993	1	212	N/A	15.1	10.6–20.6	<0.0001	N/A	N/A	N/A
>2000	2004-<2018	24	34,111	4.0–79.9	34.1	27.3–41.3		0.0000	99.5% (99.4–99.5%)	5.4–72.0
National population	2005–2010	5	4,110	21.9–70.4	47.7	29.3–66.6	0.0771	<0.0001	99.3% (99.0–99.5%)	0.0–99.8
National and non-national populations	<1993- <2018	20	30,213	4.0–79.9	29.9	23.4–36.8		0.0000	99.4% (99.3–99.5%)	4.7–64.9
**Saudi Arabia**										
All*	<1993-<2018	9	6,884	4.0–79.9	34.1	19.7–50.1	N/A	< 0.0001	99.4% (99.3–99.5%)	0.0–89.7
Males^c^	<1993-<2015	6	4,360	15.1–79.9	47.2	33.0–61.7	0.0007	<0.0001	98.9% (98.4–99.2%)	4.6–92.8
Females^c^	2009-<2018	3	2,524	4.0–21.9	12.2	3.1–26.1		<0.0001	98.6% (97.5–99. 2%)	0.0–100.0
<2000	<1993	1	212	N/A	15.1	10.6–20.6	0.0083	N/A	N/A	N/A
>2000	2005-<2018	8	6,672	4.0–79.9	36.7	21.1–53.9		<0.0001	99.5% (99.4–99.6%)	0.0–92.8
National population	2005–2010	3	3,204	21.9–55.5	42.9	19.2–68.4	0.4506	<0.0001	99.5% (99.2–99.7%)	0.0–100.0
National and non-national populations	<1993- <2018	6	3,680	4.0–79.9	29.8	11.0–53.1		<0.0001	99.5% (99.4–99.6%)	0.0–99.1
**UAE**										
All***	2004–2010	7	14,447	18.0–74.5	35.0	24.7–46.1	N/A	< 0.0001	99.4% (99.2–99.5%)	4.6–75.2
Males^c^	2004–2010	3	6,206	21.4–74.5	44.2	17.1–73.3	0.3889	<0.0001	99.7% (99.6–99.8%)	0.0–100.0
Females^c^	2004–2010	3	6,203	18.0–52.2	29.0	12.9–48.4		<0.0001	99.2% (98.7–99.5%)	0.0–100.0
<2000	N/A	0	N/A	N/A	N/A	N/A	N/A	N/A	N/A	N/A
>2000	2004–2010	7	14,447	18.0–74.5	35.0	24.7–46.1		<0.0001	99.4% (99.2–99.5%)	4.6–75.2
National population	N/A	0	N/A	N/A	N/A	N/A	N/A	N/A	N/A	N/A
National and non-national populations	2004–2010	7	14,447	18.0–74.5	35.0	24.7–46.1		<0.0001	99.4% (99.2–99.5%)	4.6–75.2
**Oman**										
All**	2005–2015	5	6,428	11.7–66.7	28.8	14.6–45.6	N/A	< 0.0001	99.4% (99.2–99.5%)	0.0–90.6
Males^c^	2005–2010	2	1,493	34.0–66.7	50.3	19.8–80.6	0.0532	<0.0001	99.2% (98.4–99.6%)	N/A
Females^c^	2005–2010	2	1,467	16.0–23.1	19.3	12.8–26.7		0.0016	90.0% (63.2–97.3%)	N/A
<2000	N/A	0	N/A	N/A	N/A	N/A	N/A	N/A	N/A	N/A
>2000	2005–2015	5	6,428	11.7–66.7	28.8	14.6–45.6		<0.0001	99.4% (99.2–99.5%)	0.0–90.6
National population	N/A	0	N/A	N/A	N/A	N/A	N/A	N/A	N/A	N/A
National and non-national populations	2005–2015	5	6,428	11.7–66.7	28.8	14.6–45.6		<0.0001	99.4% (99.2–99.5%)	0.0–90.6
**Kuwait**										
All*	2009–2015	3	4,543	17.1–70.4	41.4	11.7–75.2	N/A	< 0.0001	99.7% (99.5–99.8%)	0.0–100.0
Males^c^	2009	1	463	N/A	70.4	66.0–74.5	N/A	N/A	N/A	N/A
Females^c^	2009	2	443	N/A	39.3	34.7–44.0		N/A	N/A	N/A
<2000	N/A	0	N/A	N/A	N/A	N/A	N/A	N/A	N/A	N/A
>2000	2009–2015	3	4,543	17.1–70.4	41.4	11.7–75.2		<0.0001	99.7% (99.5–99.8%)	0.0–100.0
National population	2009	2	906	39.3–70.4	55.1	25.1–83.2	0.0099	<0.0001	98.9% (97.8–99.5%)	N/A
National and non-national populations	2015	1	3637	N/A	17.1	15.9–18.3		N/A	N/A	N/A
**Lebanon**										
All*	2009–2011	2	3154	34.6–67.4	51.0	20.4–81.3	N/A	< 0.0001	99.6% (99.4–99.8%)	N/A
Males^c^	N/A	0	N/A	N/A	N/A	N/A	N/A	N/A	N/A	N/A
Females^c^	N/A	0	N/A	N/A	N/A	N/A		N/A	N/A	N/A
<2000	N/A	0	N/A	N/A	N/A	N/A	N/A	N/A	N/A	N/A
>2000	2009–2011	2	3154	34.6–67.4	51.0	20.4–81.3		<0.0001	99.6% (99.4–99.8%)	N/A
**Palestine**										
All*	2002–2010	4	13,784	18.3–24.2	21	18.4–23.7	N/A	< 0.0001	89.7% (76.5–95.5%)	10.4–34.1
Males^c^	N/A	0	N/A	N/A	N/A	N/A	N/A	N/A	N/A	N/A
Females^c^	N/A	0	N/A	N/A	N/A	N/A		N/A	N/A	N/A
<2000	N/A	0	N/A	N/A	N/A	N/A	N/A	N/A	N/A	N/A
>2000	2002–2010	4	13,784	18.3–24.2	21	18.4–23.7		<0.0001	89.7% (76.5–95.5%)	10.4–34.1
**Iraq**										
All*	2012	1	2,038	N/A	20.0	18.3–21.8	N/A	N/A	N/A	N/A
Males^c^	N/A	0	N/A	N/A	N/A	N/A	N/A	N/A	N/A	N/A
Females^c^	N/A	0	N/A	N/A	N/A	N/A		N/A	N/A	N/A
<2000	N/A	0	N/A	N/A	N/A	N/A	N/A	N/A	N/A	N/A
>2000	2012	1	2,038	N/A	20.0	18.3–21.8		N/A	N/A	N/A
**Qatar**										
All*	2011	1	2,021	N/A	15.0	13.5–16.5	N/A	N/A	N/A	N/A
Males^c^	N/A	0	N/A	N/A	N/A	N/A	N/A	N/A	N/A	N/A
Females^c^	N/A	0	N/A	N/A	N/A	N/A		N/A	N/A	N/A
<2000	N/A	0	N/A	N/A	N/A	N/A	N/A	N/A	N/A	N/A
>2000	2011	1	2,021	N/A	15.0	13.5–16.5		N/A	N/A	N/A
National population	N/A	0	N/A	N/A	N/A	N/A	N/A	N/A	N/A	N/A
National and non-national populations	2011	1	2,021	N/A	15.0	13.5–16.5		N/A	N/A	N/A
**Morocco**										
All**	2006–2010	4	4,659	13.0–20.8	16.6	12.6–21	N/A	< 0.0001	93.3% (86.2–96.8%)	2.2–40.5
Males^c^	2006–2010	2	2,546	20.0–20.8	20.5	19.0–22.1	<0.0001	0.6257	0.0%	N/A
Females^c^	2006–2010	2	2,113	13.0–13.3	13.2	11.7–14.6		0.8581	0.0%	N/A
<2000	N/A	0	N/A	N/A	N/A	N/A	N/A	N/A	N/A	N/A
>2000	2006–2010	4	4,659	13.0–20.8	16.6	12.6–21		<0.0001	93.3% (86.2–96.8%)	2.2–40.5
**Libya**										
All**	2007	2	2,242	11.6–21.5	16.2	7.8–27.0	N/A	< 0.0001	97.5% (93.8–99.0%)	N/A
Males^c^	2007	1	1,123	N/A	21.5	19.1–24.0	N/A	N/A	N/A	N/A
Females^c^	2007	1	1,119	N/A	11.6	9.8–13.6		N/A	N/A	N/A
<2000	N/A	0	N/A	N/A	N/A	N/A	N/A	N/A	N/A	N/A
>2000	2007	2	2,242	11.6–21.5	16.2	7.8–27.0		<0.0001	97.5% (93.8–99.0%)	N/A
**Jordan**										
All**	2004–2007	4	3,916	11.0–20.0	15.9	12.1–20.1	N/A	< 0.0001	91.8% (82.2–96.2%)	2.2–38.6
Males^c^	2004–2007	2	1,938	18.2–20.0	19.0	17.3–20.8	0.0078	0.3143	1.2%	N/A
Females^c^	2004–2007	2	1,978	11.0–14.9	12.9	9.3–17.0		0.0098	85% (39.1–96.3%)	N/A
<2000	N/A	0	N/A	N/A	N/A	N/A	N/A	N/A	N/A	N/A
>2000	2004–2007	4	3,916	11.0–20.0	15.9	12.1–20.1		<0.0001	91.8% (82.2–96.2%)	2.2–38.6
**Sudan**										
All*	2012	1	2,211	N/A	11	9.7–12.4	N/A	N/A	N/A	N/A
Males^c^	N/A	0	N/A	N/A	N/A	N/A	N/A	N/A	N/A	N/A
Females^c^	N/A	0	N/A	N/A	N/A	N/A		N/A	N/A	N/A
<2000	N/A	0	N/A	N/A	N/A	N/A	N/A	N/A	N/A	N/A
>2000	2012	1	2,211	N/A	11	9.7–12.3		N/A	N/A	N/A
**Egypt**										
All**	2006	2	3,664	4.0–14.0	8.3	1.3–20.5	N/A	< 0.0001	99.2% (98.4–99.6%)	N/A
Males^c^	2006	1	1,975	N/A	14.0	12.5–15.6	0.0000	N/A	N/A	N/A
Females^c^	2006	1	1,689	N/A	4.0	3.1–5.1		N/A	N/A	N/A
<2000	N/A	0	N/A	N/A	N/A	N/A	N/A	N/A	N/A	N/A
>2000	2006	2	3,664	4.0–14.0	8.3	1.3–20.5		<0.0001	99.2% (98.4–99.6%)	N/A
**Djibouti**										
All**	2007	2	1,777	9.2–18.8	13.6	5.6–24.4	N/A	< 0.0001	97.1% (92.3–98.9%)	N/A
Males^c^	2^c^007	1	707	N/A	18.8	16.0–21.9	<0.0000	N/A	N/A	N/A
Females	2007	1	1,070	N/A	9.2	7.5–11.0		N/A	N/A	N/A
<2000	N/A	0	N/A	N/A	N/A	N/A	N/A	N/A	N/A	N/A
>2000	2007	2	1,777	9.2–18.8	13.6	5.6–24.4		<0.0001	97.1% (92.3–98.9%)	N/A
**Syria**										
All*	2010	1	3,102	N/A	15.1	13.8–16.4	N/A	N/A	N/A	N/A
Males^c^	N/A	0	N/A	N/A	N/A	N/A	N/A	N/A	N/A	N/A
Females^c^	N/A	0	N/A	N/A	N/A	N/A		N/A	N/A	N/A
<2000	N/A	0	N/A	N/A	N/A	N/A	N/A	N/A	N/A	N/A
>2000	2010	1	3,102	N/A	15.1	13.8–16.4		N/A	N/A	N/A
**Tunisia**										
All*	2008	1	2,870	N/A	18.5	17.1–20.0	N/A	N/A	N/A	N/A
Males^c^	N/A	0	N/A	N/A	N/A	N/A	N/A	N/A	N/A	N/A
Females^c^	N/A	0	N/A	N/A	N/A	N/A		N/A	N/A	N/A
<2000	N/A	0	N/A	N/A	N/A	N/A	N/A	N/A	N/A	N/A
>2000	2008	1	28,70	N/A	18.5	17.1–20.0		N/A	N/A	N/A
**Yemen**										
All*	2008	1	1,175	N/A	15.2	13.2–17.4	N/A	N/A	N/A	N/A
Males^c^	N/A	0	N/A	N/A	N/A	N/A	N/A	N/A	N/A	N/A
Females^c^	N/A	0	N/A	N/A	N/A	N/A		N/A	N/A	N/A
<2000	N/A	0	N/A	N/A	N/A	N/A	N/A	N/A	N/A	N/A
>2000	2008	1	1,175	N/A	15.2	13.2–17.4		N/A	N/A	N/A

Notes: Mean effect size reported as weighted average prevalence measure with their corresponding 95% confidence interval (CI); the *p-value* of the Cochran’s Q statistic is used to inform about the statistical significance of the heterogeneity in PA prevalence estimates within the group; the *p-value* of the Cochran’s Q between-subgroups statistic is used to inform about the statistical significance of the heterogeneity in PA prevalence estimates between the results of subgroups if applicable; the I^2^ is used to assess the magnitude of between-study variation that is due to differences in PA prevalence estimates across studies rather than chance; the prediction interval is used to estimate the 95% interval in which the true PA prevalence in a new PA prevalence study will lie. p-value <0.05 was considered statistically significant. <2000: this analysis includes all studies where the data was collected before 2000. > 2000: this analysis includes all studies where the data was collected before 2000.

Meta-analyses according to the type of population (nationals and non-national populations) were only relevant in the GCC countries as per the type of setting of the GCC population. Years of data collection were considered as prior to the publication year in studies where this information was missing. The prevalence measures were then classified accordantly for the meta-analyses. All data among youth was collected after 2000.

Symbols:

* Include converted data from physical inactivity to physical activity not overlapping with other included data.

**include data from an included primary study not reported by the SR.

*** Data on moderate and vigorous physical activity levels were merged in the studies of Wasfi, 2008^36^ and Mehairi, 2013^34^, respectively, to ovoid partial overlapping of denominators data from the same study.

^a^Stratified prevalence measures were used to conduct the meta-analyses.

^b^Weighted average prevalence measures were obtained using random-effect model.

^c^Excluded the studies with a mixed male and female population.

**Figure 2:**
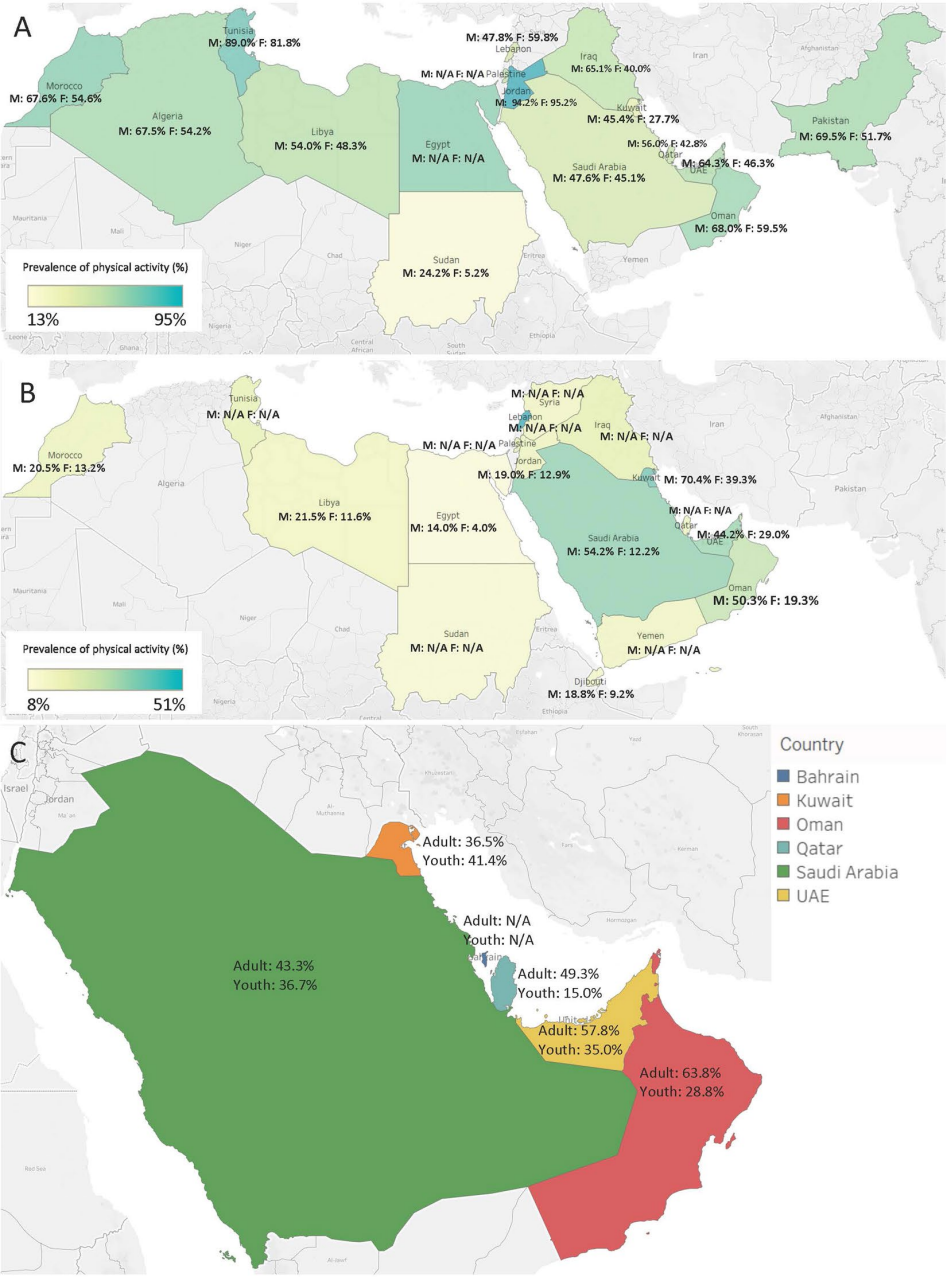
Map of the physical activity prevalence measures, using data collected after 2000: (**A**) In the adult MENA population, (**B**) in the youth MENA population, and (**C**) in the national GCC adult and youth populations. All PA data were collected after 2000. MENA countries with no PA prevalence measures among adults include Djibouti, Yemen, Bahrain, and Syria. MENA countries with no PA prevalence measures among youth include Algeria, Bahrain and Pakistan. Heterogeneity (*I*^2^) between the PA prevalence measures varied between 99.6–99.7% in adults and 99.2–99.4% in youth in MENA countries. Among GCC nationals, heterogeneity (*I*^2^) between the PA prevalence measures varied between 99.8–99.8% in adults and 99.0–99.5% in youth. N/A is used to indicate the non-availability of disaggregated prevalence data according to gender in **(A,B**) and age (adult/youth) in **(C**).

In most of the MENA countries among both adult and youth populations, more males engage in PA than females except for adults in Lebanon and Jordan (Tables 2, 3, Fig. 2). In MENA as a whole and within GCC countries, greater PA participation was observed among adults than in youth. However, caution must be practiced in interpreting these results given the limited number of included studies and the high heterogeneity between the included prevalence measures.

Only the SR of Yammine *et al*. 2016 pooled the PA prevalence measures among adolescents in the UAE^56^ for moderate and vigorous levels of PA [19.2% (95% CI = 18.5–19.9%, I^2^ = 98.6%) and 24.7% (95% CI = 23.2–26.3%, I^2^ = 97.8%)]. This pooled prevalence was higher among males than females for both moderate and vigorous levels of PA [OR_pooled_: 1.23% (95% CI = 1.13–1.35%, I^2^ = 89.5%) and OR_pooled_: 2.6% (95% CI = 2.14–3.14%, I^2^ = 84.5%), respectively]. Our pooled prevalence of both moderate and vigorous levels of PA in the UAE among youth was 36.0% (95% CI = 23.9–49.9%).

For adults, most studies used PA definitions consistent with international recommendations of i) at least 150 minutes of moderate-intensity activity per week or ii) at least 600 or more metabolic equivalent of task (MET)-minutes per week of vigorous or moderate activity^6,59,60^. For youth, most studies used a PA definition corresponding to 60 minutes of moderate intensity activity as per the Arab Teens Lifestyle Study (ATLS) questionnaire PA measurement and compilation (Supplementary, Table S12)^61^.

Only one study^62^ used a daily threshold of ≥13,000 steps using the pedometer to quantify sufficient PA participation (Supplementary, Table S12). Most studies pertaining to adults and youth utilized validated tools along with a validated version in Arabic or Urdu language, depending on the primary language of the country.

The results from univariate meta-regression analyses assessing the relationship between study-level covariates and the PA prevalence measures are summarized in Table 4. Factors associated with a higher prevalence of PA among adults were the use of the two validated questionnaires- IPAQ and GPAQ, and data collected after the year 2000. Factors associated with a higher prevalence of PA among youth were male gender and the use of IPAQ. The HBSC, PACE+ questionnaires and country level coverage of the study are associated with a lower PA prevalence among youth (Table 4**)**.

**Table 4:** Univariate meta-regression models for physical activity prevalence in MENA adult and youth general population.

Meta-regression variables	Variables categories	Number of prevalence measures	Total sample size	Effect size		Univariable analyses			Proportion of explained true variance
				Weighted average prevalence (%)	95% CI (%)	OR	95% CI	p-value^†^	R^2^ (%)
**Adults**									
Gender	Male	29	49,343	54.1	42.3–65.7	1.11	0.94–1.32	0.2286	2.72
	Female	32	44,167	43.6	31.7–55.9	Ref.	Ref.		
Data collection time	> 2000	71	105,109	50.8	45.6–56.0	**1.72**	**1.38–2.13**	**<.0001**	52.89
	<2000	4	19,593	6.2	1.7–13.3	Ref.	Ref.		
PA measurement tools	IPAQ	19	19,317	57.4	47.2–67.1	**1.32**	**1.12–1.57**	**0.0012**	41.42
	“How physically active are you” questionnaire	2	322	21.4	17.3–26.2	0.92	0.64–1.32	0.6408	
	GPAQ	37	74,049	51.1	42.8–59.3	**1.25**	**1.07–1.45**	**0.0040**	
	Nurses’ Health Study II	1	175	67.4	60.1–74.0	1.48	0.91–2.42	1.5641	
	ATLS	3	1,177	55.8	48.1–63.1	1.31	0.97–1.78	0.0782	
	Non-validated	13	29,662	23.9	11.9–42.1	Ref.	Ref.	Ref.	
Geographical coverage	Country level	42	98,662	50.4	39.8–60.9	1.08	0.93–1.25	0.3034	0.00
	Local level	33	26,040	42.7	36.1–49.7	Ref.	Ref.		
**Youth**									
Gender	Male	19	20,811	37.1	28.8–45.8	**1.27**	**1.13–1.41**	**<0.0001**	19.37
	Female	16	18,606	16.5	12.2–21.2	Ref.	Ref.		
Data collection time	> 2000	49	78,703	25.6	22.0–29.4	1.14	0.84–1.54	0.4050	0.00
	<2000	1	212	15.1	10.6–20.3	Ref.	Ref.		
PA measurement tools	Objective measurement	2	508	32.6	3.9–72.3	0.91	0.75–1.11	0.3569	35.35
	ATLS	8	5,886	38.9	22.7–56.4	0.97	0.85–1.10	0.6381	
	HBSC	2	9199	20.1	19.1–21.0	**0.80**	**0.66–0.97**	**0.0233**	
	PACE +	30	57,763	17.1	14.9–19.5	**0.76**	**0.68–0.84**	**<0.0001**	
	IPAQ	2	1,018	63.7	41.0–83.6	**1.25**	**1.03–1.52**	**0.0272**	
	Non-validated	6	4,541	28.0	13.9–48.3	Ref.	Ref.	Ref.	
Geographical coverage	Country level	34	68,422	20.3	17.3–23.3	**0.83**	**0.76–0.89**	**<0.0001**	21.70
	Local level	16	10,493	37.4	26.7–48.8	Ref.	Ref.		

Notes: Significant results are highlighted in bold. Association between the meta-regression variable and the prevalence of PA is reported as an odds ratio (OR) with its corresponding 95% confidence interval. Mean effect size reported as weighted average prevalence measure with their corresponding 95% confidence interval (CI). Weighted average prevalence measures were obtained using random-effect model. Proportion of total between-study variance explained by the model (R^2^) is used to assess the magnitude of the covariates relationship with effect size^30^. All data among youth was collected after 2000. No meta-analyses according to the time of data collection were then performed. The category “Country level” includes all prevalence measures based on a national sample of individuals representative of the entire country where the study was conducted (e.g. all cities of the country). To be included in this category, a study should clearly mention that the study was conducted at the national level. Any study based on a sample from a restricted part of the country (one or few cities, specific university, specific school, etc.) is considered in the “Local level” category.

^†^p-value <0.05 was considered statistically significant.

Abbreviations: PA: Physical activity; MENA: Middle East and North Africa; IPAQ: International physical activity questionnaire; GPAQ: Global physical activity questionnaire; ATLS: Arab Teens Lifestyle Study questionnaire; HBSC: Health Behaviour in School-aged Children Survey, PACE+: Adolescent physical activity measure questionnaire.

### Overview of studies with physical inactivity data

To define physical inactivity, we used physical activity levels below the recommended levels of PA. Hence, the validated measurement tools used to assess PA participation were also used to assess physical inactivity (Supplementary, Table S12).

Two studies used a daily threshold of <10,000 steps using the pedometer to quantify insufficient PA participation. Two studies used a daily threshold of a daily heart rate <159 bpm for at least 20 min and a daily heart rate <140 bpm for at least 30 min to quantify physical inactivity.

Among adults and youths, a total of 134 primary studies reported data on physical inactivity (Supplementary, Tables S10 and S11). Studies using a standard definition for physical inactivity and not overlapping with an already included data/study were included in the meta-analysis and are listed in Supplementary, Tables S6 and S7. Overall, higher prevalence measures of physical inactivity among female adults compared to male adults was noted.

A total of 58 primary studies reported factors positively correlated to physical inactivity and barriers to physical activity.

Among the included SRs, only the SR of Ranasinghe 2013^54^ reported an OR comparing the prevalence of physical inactivity between males and females in Pakistan. This SR concluded that females were significantly more inactive than males [OR: 2.1 (95% CI = 1.5–3.1), *p*-value<0.001]. In UAE, Yammine *et al*.^56^ concluded that adolescent females had a significantly higher mild level of PA compared to males [OR_pooled_: 0.82 (95% CI = 0.698–0.961%); I^2^ = 98.0%; n = 2]^56^.

### Overview of studies with sedentary behaviour

A total of 22 primary studies^63–84^ on sedentary behaviour among adults and youth were included (Supplementary, Tables S8 and S9). Adult sedentary behaviour data was reported for Kuwait, Oman, Qatar, and Saudi Arabia and youth sedentary behaviour measures were available for Bahrain, Djibouti, Jordan, Kuwait, Libya, Morocco, Oman, Saudi Arabia, and the UAE. However, the wide variability in sedentary behaviour measurement prevents measurable conclusions on age and sex-related differences.

Sedentary behaviour definitions varied for adults (three definitions) and youth (17 definitions). Definition variations were related to the type of sedentary activities considered (screen time, sitting time, game time, reading time, standing time, talking time, or total sedentary time) and the measurement unit (minutes or hours per day or week). Sedentary behaviour was assessed using the mean time (minutes per day or week) or the proportion of the population exhibiting sedentary behaviour (prevalence measure, %). The threshold used for sedentary behaviour prevalence measures (6+ hours per day, 3+ hours per day, or 2+ hours per day) also varied among the studies. Within the 13 sedentary behaviour measures in adults, 54% were reported as a sedentary behaviour prevalence measure and 46% as the mean amount of time spent sitting (Supplementary, Table S10). Among the 79 sedentary behaviour measures in youth, 63% were reported as a sedentary behaviour prevalence measure and 37% as the mean amount of time spent sitting (Supplementary, Table S11). The validated tools utilized for sedentary behaviour measurement were the same tools used for PA assessment among adults and youth.

## Discussion

Our overview included seven SRs, 229 primary studies and 203,617 participants spanning the 20 MENA countries. Our meta-analysis on PA participation after the year 2000 demonstrates that 49.2% of adults and 74.4% of the youth population were not sufficiently active. Insufficient evidence is available on sedentary behaviour due to the absence of clear measurement guidelines.

Our regional pooled PA prevalence of 50.8% among adults was similar to the recent estimate in the region of MENA and Central Asia (67.2%)^26^ but lower than the global estimates of 72.5–77%^25,26^. Minor differences in PA prevalence estimates can be explained by the differences in the number of countries considered for the calculation, the measurement of PA, and data management^25^. Evolving PA recommendations could also be an explanation; previously, sufficient PA was defined as undertaking at least 30 minutes per day of moderate intensity activity for at least five days per week or at least 20 minutes per day of vigorous intensity activity for at least three days per week or an equivalent combination achieving 600 METs-minutes per week^85,86^. This recommendation is likely more difficult to be achieved when compared to the updated recommendation of 150 minutes of moderate intensity activity or 75 minutes of vigorous intensity activity per week, or an equivalent combination regardless of the weekly frequency^6,7^, since this has more flexibility.

Our regional pooled PA prevalence of 25.6% among youth is consistent with the global estimate of about 20% of youth who are sufficiently active as per the international recommendation for PA^9,25^. The lack of awareness pertaining to the recommendation of 60 minutes of moderate- to vigorous-intensity PA daily for children and youth^6,7,87^ and its benefits can explain the very low PA participation in the region^57,58^and globally. Our findings in MENA confirm the lower PA participation of youth in comparison to adults which is observed globally^9,25^. This could partially be due to the higher recommended levels of PA for youth and in part due to the tools utilized to measure PA. Because PA habits developed during youth may persist in adulthood, young people should be encouraged to participate in a variety of physical activity that supports their natural development^6^. Implementation of locally informed, evidence-based interventions promoting PA participation at the standardized recommended levels and addressing barriers for PA would be a step in the right direction.

Our findings confirm the higher PA participation among males as compared to females which is observed globally^9,22,88,89^ and regionally^52,53,56–58^ among both adults and youth. Hence, the female youth should be a target population for future interventions to increase PA participation in the region. Understanding the barriers and determinants of PA in the MENA population is essential for developing culturally- and age-appropriate PA interventions (Table 5**)**. The sex differences in adults may be attributable to the sociocultural role of men and women in the region^54,58^, however, barriers related to the lower participation of female youth in PA requires further investigation (Table 5**)**. Physical activity interventions should be inspired from countries with the highest PA prevalence measures among girls, for example in China (75%)^88^, where walking and biking are a common form of commuting. Our analyses provide some indication of an increased PA participation among adults after 2000 in all MENA countries with available data in both periods. Although definitions used for sufficient PA participation were similar between the studies conducted before and after 2000, caution must be practiced while interpreting these results given the limited data collected before 2000. In addition, none of the studies conducted before 2000 assessed PA participation using a standard PA questionnaire (GPAQ, IPAQ) as these questionnaires were developed in 2002^90^ and 2003^91^ respectively.

**Table 5:** Barriers to physical activity and correlates of physical inactivity in MENA.

In high income countries*	In low- and middle-income income countries**
• Lower levels of urbanization and built environment (eg: Lack of parks, greenery, squares, playgrounds, sports venues)^57,68–70,72,117–139^.	• Rapid urbanization (eg: Access to motorization; Highdensity traffic; Low air quality; Pollution)^22,25,168–180,77^
• The physical environment factors (eg: Proximity to	• The physical environment factors (eg: Proximity to
destinations; Neighbourhood aesthetics; Access toopen space)^25,140^.	The physical environment factors (eg: Proximity to destinations; Neighborhood aesthetics; Access to open space)^25,176,180^.
• Psychological and social factors (eg: Unperceived	• Psychological and social factors (eg: Unperceived
benefits of PA and healthy status; Low self-efficacy;	benefits of PA and healthy status; Low self-efficacy;
Absence of social support from friends and peers;	Absence of social support from friends and peers;
Lack of exercise partner)^25,57,69,70,72,118–121,123–125,128–130,134,141,142^.	Lack of exercise partner)^25,176,177,179–182^.
• Hot arid climate^53,57,143–148^.	• Hot arid climate^176,180^.
• The employment of domestic helpers^53^.	• Cultural expectations (traditional role of women in taking care of household work and supporting extended family members)^54,171,174–180,183–190^.
• Conservative social norms particularly relevant for women^53,57,81,121,132–134,146,148–153^.	• Sociodemographic factors (eg: Age; Sex; Education level)^78,176,186,187,191–202^^.^
• Work-related factors (eg: Working long hours; Working in private sector)^124,149,154^:	• Lifestyle factors (eg: Lack of time; Poor sleeping habits; High screen time) ^203,183,181,192,204,192,191,193,182,205,176,206,207,142,180^.
• Sociodemographic factors (eg: Age; Sex; Education level)^62,70,78,121,126,131,142,149,153,155–165^.	
• Lifestyle factors (eg: Lack of time; Poor sleeping habits; High screen time)^62,65,70,72,81,94,117–126,129,131,132,134,140,141,145,149,151,154,155,157,158,164,166,167^.	

*Including GCC countries.

**Including MENA countries other than GCC.

Unlike other MENA countries, a substantial proportion of the population in the GCC countries is non-national^37^. Physical activity data differentiating nationals and non-nationals is scarce. The increase in PA in the GCC after 2000 may be explained by urbanization and socioeconomic improvement, see Table 5. Additional studies considering the demographic specificities in the GCC are needed to provide evidence on PA behaviour to develop, implement, and monitor public health programs.

We found a high proportion of heterogeneity between PA prevalence measures attributable to the type of questionnaire used. Prevalence estimates using the IPAQ were higher than those using the GPAQ. This is consistent with previous findings demonstrating PA over-reporting using the IPAQ compared to other similar questionnaires such as the GPAQ^27^, the Behavioural Risk Factor Surveillance System (BRFSS)^92^, or the accelerometer^93,94^. This results in an overestimation of the PA prevalence measures and consequently an underestimation of the physical inactivity prevalence measures^95^. Moreover, the GPAQ and IPAQ measure the total PA for a typical week making comparison difficult with the BRFSS (non-occupational PA for the past month)^96^ and the Active Australia Survey (leisure-time PA in the week preceding interview)^97^ used in Western countries.

The GPAQ is a valid measurement tool to assess moderate-to-vigorous PA endorsed by the World Health Organization^90^. However, this tool demonstrated a weak agreement with accelerometer use in young Saudi men^98^. Adapting the current GPAQ to build a regional PA questionnaire has been recommended^98^. Both measurement tool-based factors (question phrasing, time period coverage, and single or multipart questions)^99^ and individual-based factors (demographic and socioeconomic characteristics)^98^ should be considered in its development.

The international PA recommendations for youth require a mix of moderate and vigorous intensity activity (vigorous-intensity activity, including those that strengthen muscle and bone, at least three days per week)^6,7,87^. Physical activity participation among youth assessed using the ATLS questionnaire was based on the conversion of all moderate and vigorous activities during the week to a summary measure in MET-minutes with a cut-off of 1,680 METs-minutes per week corresponding to 60 minutes of moderate intensity activity every day of the week^61^. This methodology of quantifying PA participation is inclusive of participants engaging in solely moderate activities while the standard definition includes only those participants who engage in a combination of moderate activities most days of the week and vigorous activities three times per week^6,7,87^. The ATLS questionnaire could then overestimate the proportion of the population which is physically active. Nevertheless, the ATLS questionnaire was the only PA questionnaire validated among youth in MENA countries.

Objectively measured PA using accelerometer, pedometer or continuous heart rate monitor can avoid recall bias and is practical for use particularly among youth when compared to self-reported data^100^. However, the minimum number of steps measured by the pedometer required for health benefits is still debatable^101–104^ and no health-based criteria for steps per day have been developed for adults and youth^88,100,102,104,105^. Despite attempts to establish the steps per day threshold equivalent to the daily recommended PA level for youth^106^, validation studies are inconsistent on the standard threshold steps count^62,102,103,105,107,108^. Moreover, none of the international guidelines use the steps count to define sufficient PA^6,7,49,86^. Additionally, even if it was used as a gold standard in the ATLS validation study^109^, the pedometer is not designed to capture pattern, intensity, or type of PA and most are sensitive to ambulatory activities^105^.

The most basic dimensions of PA assessment are frequency, intensity, time (duration), and type of activities^100^. Currently, no single tool can adequately assess all these dimensions^101^. A combination of self-reported measures (to capture important domain- and behaviour-specific sedentary time information) and device-based measures (to measure both total sedentary time and patterns of sedentary time accumulation) is recommended^99,110^.

Sedentary behaviour is a different concept than physical inactivity and is expressed as a total number of minutes or hours spent in sedentary activities on a typical day^60^. The variability in the type of sedentary activities considered in the sedentary behaviour questionnaires prevent comparisons between countries in the region and globally. To date there is no well-accepted threshold to categorize sedentary behaviour data^59^.

The accelerometer is acknowledged as a valid and reliable instrument for objective measurement of sedentary behaviour in adults and youth^111–115^. However, it is a costly option for researchers. Moreover, it cannot provide information on the type and setting of sedentary behaviour and there is no consensus on the number of steps to identify individuals with sedentary behavior^110,116^. Sedentary behaviour has been recognized as a public health issue only in the past decade^9^ and therefore, only minimal standardized instruments exist for its assessment with no clear interpretation guidelines.

To our knowledge, this is the most comprehensive systematic overview and meta-analysis covering several dimensions of PA behaviour in the MENA region. Our overview is the first to bring together the available evidence on sedentary behaviour and PA. Moreover, most of the included primary studies have assessed PA prevalence using validated measurement tools in random national-level samples supporting the quality of the reported estimates. The country-, time-, age-, and sex-specific pooled PA prevalence measures are a major addition to the evidence on PA. This compilation will serve as a benchmark for epidemiologists and public health interventionists (Table 6). This overview identifies barriers (Table 5) and research gaps (Table 7) that could help direct future funding and investigation.

**Table 6:** Public health implications and recommendations.

• Public health efforts to increase PA and decrease sedentary time require standardized PA surveillance among all countries in the region. These measures are necessary to understand locally informed interventional strategies and to identify target populations affected by physical inactivity. This requires building and strengthening country capacity with a systems approach to scale up efforts aimed at increasing PA.
• Health-care providers can adopt a comprehensive curriculum that potentially closes the gap in medical schools, residency programs, graduate education, and nursing curricula on topics related to PA, and exercise prescription and lifestyle health^57^.
• Current data suggests that MENA youth population is insufficiently active. Physical environment interventions like parks, playgrounds, sports venues, proximity to destinations, and neighbourhood aesthetics can be beneficial. Additionally, psychosocial factors such as self-efficacy and social support from peers should also be considered while planning interventions.
• Identifying and considering PA-relevant domains (e.g. transportation, leisure-time PA) and barriers specific to the MENA populations are essential for adapting national actions and policies to tackle obesity and other non-communicable diseases.
• Considering the increasing urbanization in the region^115^, urban design favourable for PA has been suggested as a potential strategy to mitigate the impacts of urbanization on PA in low- and middle-income MENA countries^25^.
• Clear guidelines for sedentary behaviour measurement and interpretation in order to characterize patterns of sedentary behaviour in the MENA countries are required.

**Table 7:** Evidence gap and future research.

There is a need to:
• Clarify standardized methods of measuring PA, inactivity and sedentary behaviour for future studies.
• Conduct large and up-to-date country-level studies using standardized tools and definitions to quantify PA participation^52–54,56^ and sedentary behavior^52,54^.
• Generate data on PA-related outcomes in MENA countries other than the GCC countries.
• Explore differences in PA participation between nationals and non-nationals in the GCC countries.
• Further understand the personal, social and environmental barriers to PA, particularly in relation to the different domains of PA (leisure time, occupational, transports, and households PA) specific to the region^53,56^ to facilitate effective locally informed interventions.
• Identify target population groups (girls, youth, obese) for focused interventions^52,53^.
• Follow PA trends over time^53^.

Limitations include the restriction of the search strategy to PubMed/MEDLINE. However, we searched additional sources indexing grey and non-grey literature relevant to the region. Moreover, the included SRs have searched in total 21 literature sources for primary studies including grey literature sources, which are potentially important data sources for MENA countries^27,31^. This minimizes the risk of publication bias in our overview. We also extracted the data from the primary studies to complete the missing information and provide a complete overview of the available data.

The data that we used on PA and sedentary behaviour was collected before 2018; new PA data from MENA countries may have been published since. The limited data on PA behaviour in the MENA countries could be explained by the targeted population and outcomes of the included SRs. To optimize the quantitative synthesis, we converted physical inactivity data, using a standard definition, to PA measures. Despite attempts to minimize the heterogeneity using several subgroup analyses, a high heterogeneity between the PA prevalence measures persisted. Although most of the primary studies used validated PA-measurement tools, differences in PA domains captured by these measurement tools contributes to the heterogeneity between estimates. Another limitation of our metanalysis could be the minor differences in the definitions of the ‘active’ category among youth (e.g. number of days per week or steps). However, since only a limited number of studies had these differences, we feel this may have a minimal impact on the results. Differences according to the area of residence (rural *versus* urban) and the study coverage (country *versus* local coverage) might have been additional sources of heterogeneity in the reported prevalence measures.

In comparison to the global average estimates, our regional PA prevalence estimates for MENA were lower among both adults and youth. The lower PA participation by females and youth observed globally was also found in several MENA countries. More recent data suggests an increased PA participation in the region as a whole. Some of the variability in the PA measures might be attributable to the type of questionnaires used for data collection. Trends and patterns need to be confirmed in future investigations. Whether sedentary behaviour is a public health issue in the MENA region remains unclear, due to the absence of guidelines to quantify and interpret it. Harmonizing definitions of sedentary behaviour would be useful to generate evidence for the region. Promoting PA and limiting sedentary behaviour should be health priorities for both adults and the youth to reduce the overall burden of obesity and other non-communicable diseases. Additionally, promoting PA surveillance and identifying and investigating sedentary behaviour trends utilizing validated and reliable measurement tools consistent at national and international levels is essential to allow meaningful comparisons and to implement effective evidence-based interventions.

## Supplementary information

Supplementary information.

## Data Availability

The datasets generated during and/or analysed during the current study are available from the corresponding author on reasonable request.
