# Supplementary material for: Physical activity and sedentary behaviour in the Middle East and North Africa: An overview of systematic reviews and meta-analysis
Source: Sci Rep. 2020 Jun 9;10:9363. doi: 10.1038/s41598-020-66163-x (PMC7283267; doi:10.1038/s41598-020-66163-x)
Supplement: Supplementary file 1 — Supplementary information. [file 41598_2020_66163_MOESM1_ESM.pdf]

Supplementary Information

Physical activity and sedentary behaviour in the Middle East and North Africa: An overview of systematic reviews and meta-analysis

Sonia Chaabane[1], Karima Chaabna[1], Amit Abraham[1], Ravinder Mamtani[1], Sohaila Cheema[1]

AUTHORS AFFILIATION AND ADDRESS

[1]Institute for Population Health, Weill Cornell Medicine-Qatar
Qatar Foundation - Education City
P.O.Box : 24144
Doha - Qatar

Table S1. The 2009 PRISMA checklist for reporting a systematic review.

Section/topic	#	Checklist item	Reported on page #
**TITLE**			
Title	1	Identify the report as a systematic review, meta-analysis, or both.	1
**ABSTRACT**			
Structured summary	2	Provide a structured summary including, as applicable: background; objectives; data sources; study eligibility criteria, participants, and interventions; study appraisal and synthesis methods; results; limitations; conclusions and implications of key findings; systematic review registration number.	2
**INTRODUCTION**			
Rationale	3	Describe the rationale for the review in the context of what is already known.	3
Objectives	4	Provide an explicit statement of questions being addressed with reference to participants, interventions, comparisons, outcomes, and study design (PICOS).	3
**METHODS**			
Protocol and registration	5	Indicate if a review protocol exists, if and where it can be accessed (e.g., Web address), and, if available, provide registration information including registration number.	4
Eligibility criteria	6	Specify study characteristics (e.g., PICOS, length of follow-up) and report characteristics (e.g., years considered, language, publication status) used as criteria for eligibility, giving rationale.	5
Information sources	7	Describe all information sources (e.g., databases with dates of coverage, contact with study authors to identify additional studies) in the search and date last searched.	4
Search	8	Present full electronic search strategy for at least one database, including any limits used, such that it could be repeated.	4
Study selection	9	State the process for selecting studies (i.e., screening, eligibility, included in systematic review, and, if applicable, included in the meta-analysis).	5
Data collection process	10	Describe method of data extraction from reports (e.g., piloted forms, independently, in duplicate) and any processes for obtaining and confirming data from investigators.	5
Data items	11	List and define all variables for which data were sought (e.g., PICOS, funding sources) and any assumptions and simplifications made.	5-7
Risk of bias in individual studies	12	Describe methods used for assessing risk of bias of individual studies (including specification of whether this was done at the study or outcome level), and how this information is to be used in any data synthesis.	9<br>Table S7
Summary measures	13	State the principal summary measures (e.g., risk ratio, difference in means).	6-8
Synthesis of results	14	Describe the methods of handling data and combining results of studies, if done, including measures of consistency (e.g., I^2) for each meta-analysis.	6-8

Section/topic	#	Checklist item	Reported on page #
Risk of bias across studies	15	Specify any assessment of risk of bias that may affect the cumulative evidence (e.g., publication bias, selective reporting within studies).	9
Additional analyses	16	Describe methods of additional analyses (e.g., sensitivity or subgroup analyses, meta-regression), if done, indicating which were pre-specified.	9
**RESULTS**			
Study selection	17	Give numbers of studies screened, assessed for eligibility, and included in the review, with reasons for exclusions at each stage, ideally with a flow diagram.	10, 34
Study characteristics	18	For each study, present characteristics for which data were extracted (e.g., study size, PICOS, follow-up period) and provide the citations.	10-13 Tables S8-11
Risk of bias within studies	19	Present data on risk of bias of each study and, if available, any outcome level assessment (see item 12).	10 Tables S8-11
Results of individual studies	20	For all outcomes considered (benefits or harms), present, for each study: (a) simple summary data for each intervention group (b) effect estimates and confidence intervals, ideally with a forest plot.	10-14 Tables S8-11
Synthesis of results	21	Present results of each meta-analysis done, including confidence intervals and measures of consistency.	10-14 Tables 2-3
Risk of bias across studies	22	Present results of any assessment of risk of bias across studies (see Item 15).	Table 4
Additional analysis	23	Give results of additional analyses, if done (e.g., sensitivity or subgroup analyses, meta-regression [see Item 16]).	Tables 2-3 Figure 2
**DISCUSSION**			
Summary of evidence	24	Summarize the main findings including the strength of evidence for each main outcome; consider their relevance to key groups (e.g., healthcare providers, users, and policy makers).	15-18 Tables 6-7
Limitations	25	Discuss limitations at study and outcome level (e.g., risk of bias), and at review-level (e.g., incomplete retrieval of identified research, reporting bias).	18-19
Conclusions	26	Provide a general interpretation of the results in the context of other evidence, and implications for future research.	19
**FUNDING**			
Funding	27	Describe sources of funding for the systematic review and other support (e.g., supply of data); role of funders for the systematic review.	20

Source: Moher D, et al 2009 [1]

Available at: http://www.prisma-statement.org/documents/PRISMA%202009%20checklist.pdf

Table S2. PRIO-harms checklist for reporting an overview of systematic reviews (OoSRs).

Section/topic	(Sub-)item#	Checklist item	Reported on page#
Title<br>1. Title	1a	Specify the study design with terms such as ‘‘overview of (systematic) reviews,’’ ‘‘umbrella review,’’ ‘‘(systematic) review of systematic reviews,’’ or ‘‘(systematic) meta-review’’ in the title of the OoSRs.	1
	1b	Mention ‘‘safety’’ or harms related terms, or the adverse event(s) of interest in the title of the OoSRs.	N/A
Abstract			
2. Structured-like summary	2a	Provide a structured-like abstract, as applicable: background, objective, data sources, selection criteria, data extraction, review appraisal, data synthesis methods, results, limitations, conclusions.	2
	2b	Report the main findings of analysis of harms undertaken in the OoSRs or/and in the included SRs.	2
Introduction			
3. Rationale	3a	Specify the rationale and the scope (wide or narrow agendas) for the overview in the context of an existing body of knowledge on the topic.	3
	3b	Provide a balanced presentation of potential benefits and harms of the intervention(s).	3
	3c[a]	Define which events are considered harms according to previous literature and provide a clear rationale for the specific harms included in the OoSRs.	3
4. Objectives (PICOS)	4	Provide an explicit statement of research question(s) that specifies PICOS: *Participants *Interventions *Comparators *Outcomes *Study design	3
Methods			
5. Protocol and registration	5a	Indicate if a protocol exists or not.	4
	5b	If registered, provide the name of the registry (such as a valid Web address, PROSPERO).	4
6. Eligibility criteria and outcomes of interest	6a	Specify inclusion and exclusion criteria for study design, participants, interventions, and comparators in detail.	4-5
	6b	List (and define whenever it is necessary) the outcomes for which data were recorded, ideally include prioritization of main and additional outcomes.	4-5
	6c	Include adverse events as (primary or secondary) outcome of interest. Define them and grade their severity (such as mild, moderate, severe, fatal; severity could also be described in the appendix), if appropriate.	4-5
	6d[b]	Specify report characteristics (such as language restrictions, publication status, and years considered) used as criteria for eligibility for the OoSRs (see also item 7).	4-5
7. Information sources	7a	Search at least two electronic databases.	4
	7b	Search supplementary sources (e.g., hand searching, reference lists, related reviews and guidelines, protocol registries, conference abstracts, and other gray literature).	4
	7c	Report the date of last search and/or dates of coverage for each database.	4
8. Search strategy[c]	8a	Specify full electronic search strategy (algorithm) for at least one database including any limits used (e.g., language and date restrictions-see also subitems 6d and 7c) such that it could be repeated.	Published in the protocol

	8b	Present any additional search process (e.g., algorithm or filter for adverse events, searches in pertinent websites) specifically to identify adverse events that have been investigated.	Published in the protocol
9. Data management and selection process	9a[d]	Describe the software that was used to manage records and data throughout the OoSRs.	4-5
	9b	Define what is an SR and provide the process for selecting SRs and its relevant details (screening the title and abstract or full text by at least two reviewers, selection by multiple independent investigators and resolving disagreements by consensus).	4-5
	9c	Report any attempt to handle overlapping (include one review among multiple potential candidates by choosing for example the most updated SR, the most methodologically rigorous SR or the SR with larger number of primary studies).	4-5
10. Additional search for primary studies	10	Report additional search to identify eligible primary studies (e.g., searching in more databases or update the search) and its relevant details.	No
11. Data collection process	11a	Describe the method of data extraction from included SRs (e.g., data collection form, extraction in duplicate and independently, resolving disagreements by consensus).	4-5
	11b	Report any processes for obtaining, confirming, or updating data from investigators (e.g., contact with authors of included reviews, obtain data from primary studies of included reviews).	4-5
12. Data items	12	List (and define whenever is necessary) the variables for which data were recorded (e.g., PICOS items, number of included studies and participants, dose, length of follow up, results, funding sources) and any data assumptions and simplifications made.	4-5
13. Assessment of methodological quality and quality of evidence	13a	State the evaluation of reporting or/and methodological quality (e.g., using PRISMA or PRISMA-harms, AMSTAR or R-AMSTAR) of the included reviews.	9<br>Table S7
	13b[e]	State the evaluation of quality for individual studies that are included in the SRs (inform whether tools such as Jadad or RoB of Cochrane were used by the included reviews) and for the additional primary studies.	9<br>Table S7
	13c	State the evaluation of quality of evidence (e.g., using GRADE approach).	Table 1
	13d	Describe the methods (e.g., piloted forms, independently, in duplicate) used for the quality assessment.	9<br>Table S7<br>Table 1
14. Meta-bias(es)	14	Specify any planned assessment of meta-bias(es) (such as publication bias or selective reporting across studies, ROBIS tool).	Table 1
15. Data synthesis	15a	Specify clearly the method (narrative, meta-analysis, or network meta- analysis) of handling or synthesizing data and their details (e.g., state the principal summary measures that were extracted or calculated, how heterogeneity was assessed, what statistical approaches were used if a quantitative synthesis has been conducted)	6-9
	15b	Describe the software that was used to analyze the data if a quantitative synthesis has been conducted.	9
	15c	Report if zero events are included in the studies and how they were handled in statistical analyses, if relevant.	N/A
	15d	Describe methods of any prespecified additional analyses (such as sensitivity or subgroup analyses, meta-regression).	6-9
Results			

16. Review and primary study selection	16a	Provide the details of review selection (e.g., numbers of reviews screened, retrieved, and included and excluded in the overview) and the number of the additional eligible primary studies that were included, ideally with a flow diagram of the overview process.	10-14
	16b	Present a flow diagram that gives separately the number of studies focused on harms outcomes.	10-14
	16c[c]	List the studies (full citation) that were excluded after reading the full text and provide reasons.	Tables S8-S11
17. Review and primary study characteristics	17a[c]	Describe characteristics of each included SR in tables (such as title or author, search date, PICOS, design and number of studies included, number and age range of participants, dose/frequency, follow up period [treatment duration], review limitations, results or conclusion) and of each additional primary study.	10-14<br>Tables S8-S11
	17b	For each included SR report language and publication status restrictions that have been used.	10-12<br>Tables S3-S4
18. Overlapping	18	Present or/and discuss about overlapping of studies within SRs (at least one of the following):<br>• Present measures of overlap (such as CCA).<br>• Provide citation matrix.[c]<br>• Give the number of index publications or/and discuss about overlapping. [f]	Tables S5-S6
19. Present assessment of methodological quality and quality of evidence	19	Present results in text or/and tables[c] of any quality assessment (see also subitems 13a-c):<br>• Reporting or/and methodological quality of the included SRs.<br>• Inform for the quality of the individual studies that were included in the SRs (report results for sequence generation, allocation concealment, blinding, withdrawals, bias etc.) and for the additional included primary studies.<br>• Quality of evidence.	Tables S8-S11
20. Present meta-bias(es)	20	Present results of any assessment of meta-bias(es) (such as publication bias or selective reporting across studies, ROBIS assessment).	Table 1
21. Synthesis of results	21a	Summarize and present the main findings of the overview for benefits and harms. If a quantitative synthesis has been conducted, present each summary measure with a confidence interval, prediction interval, or a credible interval and measures of heterogeneity or inconsistency.	10-14<br>Tables S8-S11<br>Tables 2-3
	21b	Give results of any additional analyses, if done (such as sensitivity, subgroup analyses, or meta-regression).	Tables 4<br>Table S12
	21c	Report results for adverse events separately for each intervention.	N/A
Discussion			
22. Summary of evidence	22	Provide a concise summary of the main findings with the strengths and shortcomings of evidence for each main outcome.	15-19<br>Tables 5-7
23. Limitations	23a	Discuss limitations of either the overview or included studies (or both) (e.g., different eligibility criteria, limitations of searching reviews, language restrictions, publication and selection bias).	18-19
	23b	Report possible limitations of the included reviews related to harms (issues of missing data and information, definitions of harms, rare adverse effects).	18-19

24. Conclusions	24a	Provide a general interpretation of the results in coherence with the review findings and present implications for practice; consider the harms equally as carefully as the benefits and in the context of other evidence.	19
	24b	Present implications for future research.	Tables 6-7
Authorship			
25. Contributions of authors	25	Provide contributions of authors.	20
26. Dual (co-)authorship	26	Report about dual (co-)authorship in the limitation or declarations of interest section.	N/A
Funding			
27. Funding or other support	27a	Indicate sources of financial and other support for the OoSRs (direct funding) or for the authors (indirect funding) or report no funding.	20
	27b	Provide name for the overview funder and/or sponsor, or for the authors' supporters.	20
	27c	Describe roles of funder(s), sponsor(s), and/or institution(s), if any, in conducted the OoSRs.	20

Source: Konstantinos I. et al 2017 [2]

Abbreviations: PRIO-harms: Preferred Reporting Items for OoSRs; SRs: systematic reviews; PICOS: Participants, Interventions, Comparisons, Outcomes, and Study design; CCA: Corrected Covered Area.

[a] Applicable mainly for OoSRs that focus on adverse events. The description could be placed in methods section.

[b] Language restrictions, publication status, and years could also be reported in information sources topic-see item 7.

[c] It could also be placed in an appendix as a supplementary material.

[d] The software used for the management of the records and data could be placed in the data collection process-see item 11.

[e] The way of evaluation (e.g., instruments) can be reported in item 19.

[f] Index publication is the first occurrence of a primary publication in the included reviews. Discussion for overlapping might be placed in the discussion section

Table S3. Characteristics of the included systematic reviews.

Systematics review	Literature search period	Literature search geographical coverage	Search terms	Data sources	MENA countries with identified data	Inclusion/exclusion criteria	Number of included studies on MENA	Targeted review population	Reported physical activity related outcomes
**Sisson, 2008** [3]	1996-2008	Global	'national prevalence of physical activity' and 'Asia', 'Europe', 'Australia', 'United Kingdom', 'Africa', 'South America', 'America', 'North America', 'Great Britain', 'Mexico', 'Canada' and 'USA'	PubMed<br>BRFSS<br>Related articles and references	Saudi Arabia, Pakistan, Tunisia, UAE	Included<br>No more than 12 years since publication,<br>In the case where successive years of data were available , the most recently available data were used,<br>In countries where no representative population data were available, studies that, in the author's best judgment, represent that country were included in the data abstraction process and were included in this compilation to provide some measure of PA for that country.<br>Excluded<br>If not presenting the data in ways that were comparable to other studies,<br>The existence of more recent versions or cycles of the survey being available, or more representative data being available for the region.	3	Youth and adult general populations	PA<br>Physical inactivity (if reported in the included study on physical activity)
**Mabry, 2010** [4]	N/S	GCC	'physical activity' or 'sedentary' and the country name of each of the Member States of the Gulf Cooperation Council (Bahrain, Kuwait, Oman, Qatar, Saudi Arabia and United Arab Emirates	PubMed<br>WHO website<br>Contacted WHO staff members in the field	Saudi Arabia, Kuwait, Bahrain, UAE, Qatar	Included<br>English language,<br>Reported the prevalence of sufficient PA in adults,<br>Used population survey methods in an attempt to obtain a representative sample.<br>Excluded<br>Reported only on the prevalence of non-communicable disease or related risk factors,<br>Were on issues not related to PA participation,<br>Were review articles without primary data on PA,	8	Adult general population	PA<br>Sedentary behavior (if reported in the included study on physical activity)

						Focused on children and adolescents,<br>Were not population-based samples, or only reported on sedentary behavior.<br>Studies with unacceptable quality of sampling and measurement methodology (n=3).			
**Ranasinghe, 2013** [5]	Inception -2012	South-Asia	'Exercise' and 'Walking', with keywords 'Physical activity', 'Inactivity', 'Physical Activity Questionnaire', 'International Physical Activity Questionnaire','IPAQ',' Global Physical Activity Questionnaire' and 'GPAQ') AND ('Sri Lanka', 'Lanka', 'Ceylon', 'India', 'Bangladesh', 'Pakistan', 'Nepal', 'Bhutan', 'Maldives'	PubMed<br>Scopus<br>Web of Science Knowledge<br>WHO website<br>The reference lists of articles<br>Possible forward citations<br>Relevant web sites and online resources	Pakistan	<u>Included</u><br>Population based studies among healthy non-institutionalized human adults (>15 years of age),<br>Cross-sectional study design or being the first phase of a longitudinal study,<br>Geographically and temporally defined population from any of the South Asian countries mentioned above,<br>Being an original study presenting data on PA,<br>Evaluating PA using questionnaires,<br>Published in English, or with detailed summaries in English<br>Peer-reviewed fully published research papers.<br><u>Excluded</u><br>Studies limited to adults engaged in a particular profession, based in hospitals/institutions and confined to those with diagnosed illnesses,<br>Conference proceedings, editorials, commentaries and book chapters/book reviews.	1	South Asian adult general population	Sedentary lifestyle or inactivity
**Yammine, 2016** [6]	Inception -2015	UAE	Boolean combination of broad terms such as [('physical activity' OR sport) AND (children OR adolescent* OR student*) AND UAE]	EMBASE<br>Medline<br>SSciELO<br>AMED<br>AUSPORT<br>SPORTDiscus<br>Google_Scholar<br>Dubai Ministry of Health<br>Health Authority of Abu Dhabi	UAE, Morocco*, Djibouti*, Libya*, Jordan*, Egypt*	<u>Included</u><br>Published and unpublished studies reporting PA level<br>On adolescents or young subjects less than 25 years old,<br>Have reported level of PA,<br>The studied population is limited to the residents of UAE,<br>Studies with prospective design.<br>We included studies reporting validated and non-validated	5	Young adult and adolescent general population	PA levels (including physical inactivity levels)

				Country reports of the WHO STEPS survey<br>Official reports not published in the academic literature<br>Hand search of the references of the Included studies<br>unpublished studies		(personal) questionnaires based on a classification of PA duration and intensity, usually over a typical week, whatever the used definition and if the PA is not the primary outcome.<br><u>Excluded</u><br>Studies which only reported the level of sedentary behavior.			
**Mabry, 2016** [7]	Inception -2016	Oil-producing countries of the Arabian Peninsula	Active living; exercise; lifestyle; physical activity; walking; screen time; sedentary; sitting or television viewing; and the name of each country in the Region (Bahrain, Kuwait, Oman, Qatar, Saudi Arabia and United Arab Emirates) or Arab	PubMed<br>Web of Science Knowledge<br>Google Scholar<br>Web Search Engine	Saudi Arabia, UAE, Oman, Qatar, Kuwait, Bahrain	<u>Included</u><br>The primary inclusion criteria were:<br>Peer-reviewed publications in the English language,<br>Country specific studies which gathered original data, fit into any phase of the Behavioral Epidemiology framework,<br>Full-texts were available.<br>Additional secondary inclusion criteria were used for the first three phases to facilitate within and cross-country comparison:<br>Phase 1: Cross-sectional studies used a clearly described measure for PA/sedentary behavior and prospective studies involved a PA intervention<br>Phase 3: Studies clearly defined PA as meeting the recommendation of 150 min/week for adults or 60 min/day for children/adolescents.<br>Phase 4: For demographic correlates, studies used a clearly described measure for PA/sedentary.<br>The secondary inclusion criteria were not used for the studies examining the non-demographic articles to ensure a comprehensive review of available research in the	27	Youth and adult general population	PA<br>Sedentary behavior

						region.<br>Excluded<br>Publications in other languages, conference proceedings and theses as well as articles in unrelated disciplines.			
**Al-Hazzaa, 2018[8]**	Up to Jan 15, 2018	Saudi Arabia	PA, exercise, physical inactivity, PA barriers and PA determinants in combination with the words "Saudi Arabia," "Saudi population," and "Saudi people	MEDLINE and Google Scholar	Saudi Arabia	Included<br>Articles in English literature, which included Saudi adults, adolescents or children without orthopedic problems and have a clear description of PA assessment methods and physical inactivity criteria<br><br>Excluded<br>Articles with no clear criteria for physical inactivity (not determined) or there was no clear description of the PA instruments used	42	Youth and adult population	Physical inactivity Correlates/Barriers to PA
**Sharara, 2018[9]**	January 2000 and January 2016	22 Arab countries	Various combinations of MeSH terms and key words were used, related to physical activity/ inactivity, sedentary lifestyle, exercise, sports, its prevalence, incidence, epidemiology, the burden it represents, and social or cultural factors	MEDLINE, Popline and Social Sciences, Citation Index, reference lists of the articles, WHO surveys on non-communicable disease risk factors (STEPS), Global School-based Student Health Surveys (GSHS)	Algeria, Bahrain, Djibouti, Egypt, Iraq, Jordan, Kingdom of Saudi Arabia (KSA), Kuwait, Lebanon, Libya, Morocco, Oman, Palestine, Qatar, Sudan, Syria, Tunisia, UAE, and Yemen	Included<br>Physical activity or inactivity as an outcome or a determinant,<br>ere conducted among residents of Arab countries, Described the design and methods,<br>Reported on sample size,<br>Described how physical activity/inactivity was measured,<br>Reported on the prevalence of physical activity/inactivity,<br>Multi-country studies were included if they presented data on at least one Arab country<br>Articles which fulfilled the quality criteria informed by the Preferred Reporting Items for Systematic Reviews and Meta-Analyses (PRISMA) guidelines including clear eligibility criteria for study selection, description of information sources, data and variables	143	Adults and children/adolescents	Physical inactivity Barriers to PA Factors associated with physical inactivity

						Excluded<br>Studies conducted exclusively on patients with a particular disease diagnosis,<br>Studies conducted on Arabs residing outside the Arab region<br>Studies that did not report on sample size, age range of study population, and those that presented unclear or inconsistent numbers			
**Actual overview**	Inception -2016	20 MENA countries	Search terms used by the included SRs	PubMed<br>BRFSS<br>WHO website<br>Contacted WHO staff members in the field<br>Scopus<br>Web of Science Knowledge<br>The reference lists of articles<br>Possible forward citations<br>EMBASE<br>SSciELO<br>AMED<br>AUSPORT<br>SPORTDiscus<br>Google_Scholar<br>Dubai Ministry of Health<br>Health Authority of Abu Dhabi<br>Country reports of the WHO STEPS survey<br>Official reports not published in	Saudi Arabia, UAE, Oman, Qatar, Kuwait, Bahrain, Pakistan, Tunisia, Morocco, Djibouti, Libya, Yemen, Sudan, Syria, Palestine, Algeria, Iraq, Lebanon, Jordan, Egypt	Inclusion and exclusion criteria used by the seven included SRs	229	Youth and adult general population	PA<br>Physical inactivity<br>Sedentary behavior<br>Barriers to PA<br>Factors associated with physical inactivity

				the academic literature<br>Hand search of the references of the Included studies<br>unpublished studies<br>Relevant web sites and online resources<br>Related articles and references					

PA: physical activity; UAE: United Arab Emirates; GCC: Gulf cooperation council; MENA: Middle-East and North Africa; SR: systematic review; USA: United States of America; BRFSS: Behavioral Risk Factor Surveillance System; WHO: World Health Organization; IPAQ: International physical activity questionnaire; GPAQ: Global physical activity questionnaire; min: minutes.

* Country data was extracted from the study of Guthold, 2010 [10] included in the SR of Yammine, 2016 [6]. These country data were not reported in the SR of Yammine, 2016 [6].

Table S4: Summary of qualitative results from the included systematic reviews.

Systematic review	Outcomes/population	Main conclusions	Identified limitations by the systematic review	Identified Strengths by the systematic review	Identified research gaps/the review	Recommendations
**Sisson, 2008** [3]	PA<br>Physical inactivity (if reported in the included study on physical activity)<br><br>Global youth and adult general populations	"In most studies of represented countries, less than 50% of the population was active."<br><br>"Males were more active than females in adult and youth populations."<br><br>"Countries with the highest prevalence of PA in adults are: Switzerland, Sweden and Denmark and those with the lowest were Thailand, Saudi Arabia, Brazil and Iran."<br><br>"Countries with the highest prevalence of PA in youth are: Australia, China and Ireland and those with the lowest were Belgium, France and Tonga."<br><br>"The highest prevalence of physical inactivity was among Saudi Arabian women and the ones with the lowest prevalence were men from the United States."<br>"The prevalence of meeting PA recommendations was reported more frequently than prevalence of physical inactivity or 'no leisure-time physical activity'. "<br><br>"The prominent used surveys are BRFSS, IPAQ, and Active Australia."	"Discrepancy in the methods of data collection and classification of 'sufficient physical activity' across studies. "<br><br>"The use of different surveys, all reporting time in PA, may not be comparable when it comes to reporting the prevalence of the sample meeting the minimum levels of PA."<br><br>"The IPAQ overestimate the prevalence of PA compared with BRFSS."<br><br>"Different studies used different age ranges, causing difficulties when comparing activity levels across studies among both youth and adults."<br><br>"Difficulty and variability to capture transportation, occupation and active living behaviors such as chores and gardening (important for accumulating health-enhancing physical activity) from instrument to instrument."<br><br>"The use of the same instrument across populations will not capture the variability in the type of activities between populations and could not cover all aspects of physical activities. As a consequence, differences in PA levels across populations may be masked."	"The compilation of existing international data available on the prevalence of meeting public health PA recommendations in adults and youth will serve as a resource to epidemiologists and interventionists alike as it provides a benchmark for improvement and inter-country comparisons, as well as a basis for further examinations."<br><br>"Three multinational examinations of PA behavior that examine at least 23 countries each, which allows for some more direct comparisons across countries."	"Need to discuss on the most appropriate and feasible methods to use in the measurement of PA in epidemiologic studies have not been recently instituted, as they have been ongoing for more than 20 years."<br><br>"No health-based criteria for steps per day have been developed in adults or youth. It is not known that the accumulation of >13 0000 steps/ day for boys is sufficient to result in health benefits."<br><br>"Surveillance studies need to strive for an international standardization and recommendation-based metric to allow for greater comparisons between countries and regions and to determine the prevalence of meeting recommendations that are based on improved health such as 30 min moderate PA on 5 days per week or 20 min of vigorous PA on 3 days per week."<br><br>"Need for more PA data globally, there is a pressing need to understand changes in PA levels in several regions, in particular those countries undergoing economic and social transition."<br><br>"Greater collaboration among researchers in the standardization of surveillance methods to ensure that quality physical activity and inactivity surveillance is being conducted on a global level."<br><br>"Designing studies that are comprehensive in the measurement of activity and sedentary behaviors	Global need to increase PA.

					including transportation, occupation, recreation and other utilitarian tasks."<br><br>"Models of 'best practice' in the surveillance of PA should be identified and utilized in a collaborative fashion across countries."	
**Mabry, 2010** [4]	PA<br>Sedentary behaviour (if reported in the included study on physical activity)<br><br>GCC Adult general population	"Low prevalence of participation in levels of PA sufficient for health benefits among men and women in the GCC."<br><br>"The best available evidence indicates that the prevalence of sufficient PA participation ranged from 39.0% to 42.1% for men and 26.3% to 28.4% for women in the GCC."<br><br>"The levels of PA in the GCC are lower than those found in national studies conducted in 51 developing countries, which also utilized the IPAQ instrument [11]."<br><br>"The prevalence rates for non-occupational PA in the GCC States are lower than those found in developed countries."<br><br>"Four studies have used the GPAQ or IPAQ instruments classified PA on duration only (150 min per week) and not in combination with frequency (at least five sessions a week), an important part of the recommended amount of	"Due to the variations in methodological quality, it is difficult to compare prevalence rates and draw conclusions on the differences in prevalence of sufficient PA between countries of the GCC."<br><br>"While pedometers and accelerometers can provide objective data on 'movement', they do not identify the particular PA 'behaviors' that can be captured using self-report instruments; also, because of cost and study logistics, these instruments (while they are beginning to be used in some developed countries) at this stage tend to be used for evaluating outcomes of small-scale intervention trials, more so than for population surveillance."<br><br>"Good-quality population data on PA in the region is scarce."<br><br>"English language restriction."<br><br>"No gray literature and Arabic-language publications search."<br><br>"Only the national population was included in the sample when the GCC population is predominantly non-nationals."	"The results of three studies in this review utilized the GPAQ, which is a WHO instrument, and would have details on PA behaviour in three domains (occupation, transport and leisure), providing detailed evidence to inform related policy and program development."<br><br>"The IPAQ and GPAQ self-report instruments are beginning to deliver internationally comparative data, which recent peer-reviewed publications suggest is of reasonable quality. There is some recent evidence on their reliability and repeatability, and they can provide a cost-effective method for population monitoring of PA levels."	"Further research on the determinants of physical inactivity in the region is required, in order to have a better understanding of what might be done to increase PA participation."<br><br>"Need to examine potential seasonal variations in PA. This is of potential interest, given the particular climates of GCC countries."<br><br>"Future studies in the GCC neighbors, carrying out surveys in different seasons, would help to identify potential climatic determinants of PA participation."<br><br>"The need for countries to obtain accurate population-level information on prevalence and trends in PA participation, by using internationally recognized standard instruments."<br><br>"More research on sedentary behaviour, in addition to developing a better understanding of PA in this region."<br><br>"Accurate information on the prevalence of PA in the GCC region is also necessary in order to establish the nature of the links between physical inactivity and obesity – evidence that is needed in order to advocate for implementation of the most-relevant preventive initiatives."	GPAQ should be incorporated into the surveillance system for obesity and related chronic disease risk factors in all six GCC countries.<br><br>Health promotion strategies should aim to increase PA among both men and women as a priority public health issue.<br><br>The use of a standard, valid and reliable measure for PA is crucial, not only for population surveillance but for program development, evaluation and inter-country comparison.<br><br>Standardized study protocols to allow cross-country comparisons.

		PA needed for health benefits."<br><br>"The GCC studies measured total PA while the Australian and the USA studies report on non-occupational PA, thus making comparisons difficult."<br><br>"The studies in this review identified two primary correlates of PA – gender and age."<br><br>"Based on the two highest quality studies in this review, the proportion of physically active men was 13–14% points higher than women."<br><br>"The observed gender differences in PA participation are highly likely contributing to the higher prevalence of obesity in women compared with men in the GCC States."<br><br>'Correlation with age is less clear."	"The variation in the methodological quality of the studies:<br>• Non- population-based sampling,<br>• Use of unvalidated measurement instruments,<br>• Varying PA definitions.<br>• Only two of the reviewed studies provide any insight into sedentary behavior in the GCC States (women spend less time sitting than do men)."<br><br>"These prevalence rates are based on total PA from three domains (occupational, transport and recreational), posing a challenge when comparing these rates with studies using the BRFSS tool in the USA and those using the Active Australia instrument, which measure non-occupational PA."<br><br>"Evidence using IPAQ or GPAQ in developed countries is scarce and any comparisons must be made with caution."<br><br>"Barriers for PA in GCC are:<br>• Conservative social norms pose a particular barrier for women who have restricted freedom of movement outside the home,<br>• The hot arid climate,<br>• The high dependency on automobiles,<br>• The employment of domestic helpers."		"Identify the determinants of PA that may be responsive to shifts in the social contexts of the lives of men and women."<br><br>"Use of standardized research methodologies with nationally representative samples are urgently required in order to document properly levels of PA participation, gender and other social variations, and prevalence and trends over time."	"Prevalence estimates tend to be higher using IPAQ compared with the BRFSS tool; and thus, direct comparison is not recommended."

**Ranasinghe, 2013** [5]	Sedentary lifestyle or inactivity<br><br>South Asian adult general population	"A marked variation between studies in the definition of physical inactivity and in the questionnaires used."<br><br>"Skilled workers and professionals were more inactive than unskilled workers in the region. Similarly, higher education was a significant factor."<br><br>"Females were more inactive in the South Asian region when compared to males, a finding which is seen in most other regions and even in developed countries. Gender is an important factor to determining PA levels of a population."<br><br>"Cultural expectations may restrict the participation of women in certain forms of PA in some religious and ethnic groups in the region. Several studies reported higher physical inactivity among South Asian women."<br><br>"The traditional role of South Asian women in taking care of household work and supporting extended family members may limit the time available for them to engage in PA, in particularly leisure time physical activities."	"Most of studies were limited to regional populations."<br><br>"As South Asian countries have considerably diverse ethnic groups, the regional findings may not be generalized to whole country or for the entire South Asian region."<br><br>"No uniformity on PA assessment tools."<br>"Several studies have used IPAQ, differences in the formats used limit comparability."<br><br>"A marked heterogeneity on the definition of physical inactivity/sedentary life style and physical activity levels and in the used tools. Hence, comparisons between studies were undertaken with caution."<br><br>"Only limited number of articles reported data on the sub-domains of physical activity (work, transport, and leisure)."	"The comprehensive and easily replicable search strategy applied to three major medical databases."<br><br>"Systematic selection of the studies through the application of well-defined inclusion/exclusion criteria. The GPAQ is known to be reliable for surveys in developing countries, adaptable to incorporate cultural differences."<br><br>"The GPAQ used in the WHO STEPwise surveys and in the included studies give uniformity to the gathered data and allow meaningful comparisons."	"It is important for future researchers to adhere to a uniform way to evaluate PA in order to derive intra- and inter-regional comparable data and observe secular trends in PA."	"It is important to consider individual country profiles, proportions of urbanity and cultures when delivering public health messages focused on leisure time activity."<br><br>"Countries in the South Asian region in economic transition also need public health planning to enable the people to maintain their already existing active."<br><br>'Promoting leisure time activity has also become a challenge in South Asia due to cultural and attitudinal barriers."<br><br>'Public health messages in the region should be directed at improving all domains of activity with work and transport policies of the countries supporting them."

		"In the South Asian region, we observed that activity during work and transport is higher than leisure time activity."<br><br>"Low PA is one of the contributing risk factors for the higher obesity levels seen among Asian Indians."<br><br>"In all regional countries work and transport related activity was higher compared to leisure time activity."<br><br>"South Asian activity levels were in parallel or sometimes better than some developed countries."				'Although exercise is promoted in public health campaigns to increase the overall PA level of a population, it is important to understand the socio-economic factors associated with different populations in order to deliver effective PA interventions."
**Yammine, 2016** [6]	PA levels<br>Young adult and adolescent general population	"Almost a quarter of the young population have a total sedentary lifestyle with no PA."<br><br>"Less than half have been mildly implicated in physical activities."<br><br>"Around a fifth practiced a moderate level of PA."<br><br>"A quarter were involved in a vigorous PA."<br><br>"The adolescent subgroup analyses revealed the same results except for the nil level of PA where one large study reported this level in almost half of its sample."	"Only five studies included."<br><br>"The reported data were retrospective, recalled data, as it is the case where questionnaires are used for data collection."<br><br>"Only two studies reported PA level as their primary outcome (non-significant impact as the data is on the same relevant population)."<br><br>"The age variable was not always accurately reported but mostly limited to secondary school students."<br><br>"Confounders such as body mass index, parents' income/education, and ancestry were seldom included for	"A pooled sample of 12,988 young adults could be fairly considered as representative of UAE population."<br><br>"The meta-analysis could yield an answer as to whether the young adults and mainly the adolescents residing in UAE are getting enough PA to avoid disease in their lifetime."	"A predictable higher rate of coronary disease, diabetes, and other non-communicable chronic disease in the future."<br><br>"Need to conduct large-sampled prospective studies in order to look for potential variables and barriers which would have an impact on the behavior of the young population vis-à-vis sports and physical activities as regular lifestyle habits."	"The findings could predict a higher rate of coronary disease, diabetes, and other non-communicable chronic disease in the future."<br><br>"Need to stimulate more political commitment to promote PA among school and university students."<br><br>"Decision makers such as the ministry of health, school and

		"Mild PA was more common in female adolescents, whereas moderate and high PA were significantly higher in male adolescents."<br><br>"Young adults, mainly adolescents, residing in UAE are not getting enough PA to keep themselves healthy."<br><br>"Adolescents in UAE were more involved in moderate PA and in vigorous PA than adults in the DHA."<br><br>"Male adolescents were more involved into moderate/vigorous PA than female adolescents."<br><br>"The negative relationship between PA and age observed in the DHA is also true at the adolescent/young adult phase and that there is a gender shift where young adult males become less interested in PA than male adolescents, while female adults showed a trend to practice more PA when compared to female adolescents."<br><br>"Higher level of PA among UAE nationals indicates a behavioral shift among UAE national adolescents when becoming young adults."	analysis in most of the included studies."<br><br>"High degree of heterogeneity between studies.<br>Absence of accurate definition of PA levels among and between studies."<br><br>"Two out the five studies reported the use of personal questionnaire elaborated by the authors, whereas the remaining three studies used validated questionnaires."			university board committees, parent's committees, and local communities could work on establishing national policies in order to dedicate more 'sport' hours in a student week and to develop a wider panel of PA activities in their neighborhoods which might encourage greater involvement among students."

**Mabry, 2016** [7]	PA<br>Sedentary behaviour<br><br>Oil-producing countries of the Arabian Peninsula youth and adult general population	"A 100 publications were identified since 2000, over half of these were published since 2013."<br><br>"Overall low levels of participation in PA (particularly among young people), and high levels of sedentary behaviour (particularly among men and young people)."<br><br>"Although the prevalence of PA among adolescent was generally higher than in adults, a large percentage of both adults and adolescents did not engage in sufficient amounts."<br><br>"Gender and age were consistently association with PA."<br><br>"Men were found to be more active than women and younger people more active than older people which is consistent with other countries."<br><br>"Four studies in this review reported an inverse association of PA with education which was inconsistent with other countries. This correlate likely being more context and/or culture-specific."<br><br>"Most studies depended on self-report instruments with only a limited number	"The body of evidence included only a small number of prospective and cross-sectional studies which reported generally consistent associations between PA and sedentary behaviors and various health outcomes."<br><br>"Many of the studies to date have employed a narrow understanding of PA behavior, with their focus on "exercise" where this is a formal and structured activity."<br><br>"A wide variability and quality in the measurement instruments used and the presentation of outcomes variables of exposure which severely limits within-and between- country comparisons."<br><br>"Limited adoption of other valid and reliable tools, other than GPAQ and IPAQ, to assess PA and sedentary behaviors, measures of the physical environment, self-reported cognitive, psychosocial measures and domain-specific measures."<br><br>"Fewer published studies addressed the measurement of PA and sedentary behaviors."<br><br>"Studies were mostly focused on adults rather than children."<br><br>"The sedentary behavior research identified in this review was much more limited than that related to PA."<br><br>"Our review was restricted to published studies in the English	"The Arabic version of the Questionnaire l' Activite Physique en Altitude Chez les Enfants for children under the age of 10 years and the ATLS, the Arabic Version of the Physical Activity Self-Efficacy Scale for Adolescents were identified as valid PA instruments for young people in the Arab world."<br><br>"Publications were found from all six countries in the study area."	"The findings point towards the need for more and higher quality research."<br><br>"Examining the associations between patterns of PA and sedentary behaviors with various health outcomes in Arab populations should continue to address the knowledge gaps but future studies should employ rigorous methodologies including prospective study design and use objective measures of exposure to increase the quality of evidence available from this region."<br><br>"Further studies using standardized methods of nationally representative samples are needed to monitor trends as well as to identify population variations and vulnerable groups. This point is particularly important since countries are expected to report on PA levels for both adult and adolescent populations as part of the WHO Global Plan of Action."<br><br>"Research on domain-specific PA for different populations groups is urgently needed to guide the development of targeted regionally appropriate interventions in light of regional trends like motorization and shifts in occupational patterns."<br><br>"Evidence on potential multiple levels of influence on PA (intrapersonal, perceived, social cultural, information, natural and policy environments) for countries in this region is needed to understand the particular influences that may operate in Arab populations and their social and environmental contexts so as to inform policy and practice."	Future studies should employ rigorous methodologies including prospective study design and use objective measures of exposure to increase the quality of evidence available from this region.<br><br>Instruments such as accelerometers, pedometers, mobile phones or other electronic devices are increasingly being used across the international literature to address the limitations of self-report measures and are strongly recommended, especially in studies with children."<br><br>"Given the importance of increasing PA and reducing sedentary behaviors as part of a comprehensive population-based approach to the prevention of non-communicable

		employing objective instruments to assess either behaviors.”<br><br>“The study of sedentary behaviour, relatively new globally, is only now beginning to receive the attention of researchers in the countries of the Arabian Peninsula.”	language. It is likely that additional studies such as government reports and scientific papers published in Arabic journals do exist but were not included.”<br><br>“The search was limited to only three multidisciplinary literature databases it is possible that additional databases may have identified more studies.”<br><br>“The variation in tools and methodologies as well as methodological quality limited within and cross-country comparability.”<br><br>“These limitations may bias our view of the gaps in evidence and potential solutions.”		“Accurate measurement is critical for policy development and necessary for population monitoring of trends over time and differences between populations. It is also essential for research aimed at program evaluation of individual-based and population-based actions.”<br><br>“The proposed research agenda would be similar to that outlined globally; an ecological model of four domains of sedentary behavior focusing specifically on domestic screen time, extended sitting time in workplaces and schools, and time spent sitting in cars – not only to better understand their determinants but also in designing appropriate interventions.”<br><br>“More-basic descriptive data in the region should be a higher priority.”	disease, greater priority should be given to encouraging an increase in multidisciplinary intervention research to guide national policy and programs.”
**Al-Hazzaa., 2018** [8]	Physical Inactivity, Perceived barriers and correlates of PA<br><br>Saudi children, youth and adults	“The current review confirmed the presence of high inactivity prevalence among the majority of Saudi children, youth and adult population”<br><br>“Saudi females are much more inactive than males, beginning from early school years”<br><br>“The findings from national or subnational studies indicated that the mean physical inactivity prevalence ranged from 50% to 85% among males and from 73% to 91% among females”	“Some of the reviewed studies are lacking in one or more of the following limiting factors: The sample was not accurately represented, especially for some of the local studies, not reporting response rate, using less validated PA questionnaires and lack of statistical power calculation of the sample size”<br><br>“Unfortunately, many of the studies reporting PA prevalence among Saudi population used questionnaires that were not validated”<br><br>“The real overall prevalence of physical inactivity among Saudi females in studies using IPAQ may be much higher than what	“This systematic review is considered as the first comprehensive and updated overview on PA in Saudi Arabia, covering inactivity prevalence, reasons for exercise, correlates, and barriers to PA”	“Although time constraint, lack of facilities and resources, low self-efficacy, and lack of social support (especially in the part of women) represent a major barriers to being physically active among Saudis, there remains a need to further understand the personal, social and environmental barriers to PA in Saudi population, particularly in relation to different domain of PA (leisure time, occupational, transports, and households PA)”<br><br>“Possible barriers to PA must be determined and eliminated to promote active living and reduce inactivity among the population”	“Saudis need to be more physically active than the current estimates, to control the escalating prevalence of NCDs in this country, such as obesity, diabetes mellitus, cardiovascular diseases, and cancer”<br><br>“Saudis need to create ways to make PA opportunities more available in

		"The proportions of Saudis who are at risk of inactivity are exceedingly higher than those at risk for other coronary heart diseases"<br><br>"The majority of Saudi children and youth were not active enough to meet the minimal weekly requirements of MVPA levels"<br><br>"Sedentary behaviors appear to be highly prevalent among Saudi adolescents, as 84% of males and 91.2% of females spent more than 2 h of screen time daily"<br><br>"Active Saudi boys tend to have favorable levels of blood lipids and body composition profile compared to inactive boys"<br><br>"Reduced PA was associated with obesity and WC in adults, children, and adolescents"<br><br>"Physical inactivity increased with advancing age in children and adolescents as well as in adults"<br><br>"Increased urbanization, crowded traffic, extreme weather, cultural barriers, lack of social support, the absence of female school PA program and lack of time and resources, all	have been previously reported. This may be due to the fact that, previous studies have shown that there was the possibility of over-reporting moderate levels of PA when using IPAQ"<br><br>"PA prevalence for a population from different studies using varied PA instruments must be viewed with caution"<br><br>"The vast majority of the PA activity studies were conducted in urbanized areas of the country, apart from the few national studies and very limited studies included samples from rural areas"			schools, workplaces, and within the community settings"<br><br>"A national policy encouraging active living and discouraging inactivity be established"<br><br>"Health-care providers have an important role in promoting PA, by providing routine assessment and counseling on increasing PA, improving fitness and reducing sedentary behaviors for their patients and communities. They can adopt a comprehensive curriculum that potentially closes the gap in medical schools, residency programs, graduate education, and nursing curricula on topics related to PA, and exercise prescription and lifestyle health"

		make PA a difficult choice for the Saudis"<br><br>"Saudi women have less access to exercising facilities and limited opportunities to engage in PA"<br><br>"A large percentage of schoolgirls lacked proper information and skills on how to exercise or to be physically active"<br><br>"The most important reasons for engaging in PA among adolescents and adults are health, losing or maintaining weight, recreation, and socializing"<br><br>"Variables that are found to positively associate with adult's PA include high education level, high income, enjoyment of PA, self-efficacy, social support, and safe environment"<br><br>"The factors that are believed to be negatively associated with adult PA include advancing age, low income, lack of time, low motivation, obesity, and poor health"<br><br>"Gender (boys), self-efficacy, parental support, parental education, physical education, or sports at school and				

		support from friends and family have been found to positively associate with PA”				
**Sharara et al., 2018** [9]	Physical inactivity and its social determinants in Arab countries<br><br>Adults and children/ adolescents	“Prevalence of inactivity among adults and children/adolescents is high across countries, and is higher among women”<br><br>“Among children and adolescents, inactivity is alarmingly high, around 80% in all national surveys except Tunisia”<br><br>“Inactivity increased with age, being married, and urban residence; it decreased with increased education and employment; parity was positively associated with inactivity in one study<br><br>“Screen time, smoking and alcohol was positively associated with physical inactivity”<br><br>“Consuming fruits and vegetables were negatively correlated with physical inactivity”	“The diversity of definitions and methods among studies published in journals and the fact that only 43/157 studies used validated instruments hampers comparisons of the prevalence and correlates of physical activity, and it is possible that some of the differences we found are artifactual”<br><br>“Only 16/143 journal articles reported on surveys using nationally representative samples”<br><br>“Age categories in journal articles are more diverse”<br><br>“Standardized data available from surveys by the World Health Organization for almost all countries and journal articles show great variability in definitions, measurements and methodology”<br><br>“For some countries there are very few studies (Algeria, Comoros, Djibouti, Iraq, Somalia, Sudan and Yemen), while for others many more sources are available (for example 40 for Saudi Arabia)”	This is a comprehensive assessment of the prevalence and determinants of physical inactivity across the Arab region	“Studies using harmonized approaches, rigorous analytic techniques and a deeper examination of context are needed to design appropriate interventions”	“The high levels of inactivity in the region call for considerable efforts to tackle the material and socio-cultural aspects of the cultural context that discourage physical activity”<br><br>“Multi-sectoral efforts are needed, including collaborations among ministries of health, sports, youth and education, as well as wider collaborations that involve sectors such as transport, environment and urban planning”

			"There is also a variability in sample size, with most studies in the range of 200-2000 and a few large studies including several thousand respondents"			

Note: the evidence retrieved from each SR is related to the included countries target by the SR. Could not be totally applicable to the included MENA countries.

Abbreviations: PA: Physical activity; IPAQ: International physical activity questionnaire; GPAQ: Global physical activity questionnaire; ATLS: Arab teens lifestyle student questionnaire; PACE+: Adolescent physical activity measure questionnaire; DHA: Household Health Survey; SBRN: Sedentary Behavior Research Network MENA: Middle-East and North Africa; UAE: United Arab Emirates; GCC: Gulf cooperation council; SR: Systematic review; BRFSS: Behavioral Risk Factor Surveillance System; WHO: World Health Organization; Min: Minutes; VPA: Vigorous physical activity; MPA: Moderate physical activity; NCDs: Non-communicable diseases; CS: Cross-sectional; RS: Random sampling: RCS: Random cluster sampling; MET: Metabolic Equivalent of Task; VG: Video games; km: Kilometer; min: Minutes; wk: Week; d: day

Table S5: Converted data from physical inactivity to physical activity.

Review source, study source, country	Physical inactivity definition, population	Physical inactivity prevalence	Inclusion or exclusion/reason	Converted physical activity prevalence
Sisson, 2008[3]; Sharara, 2018[9]; Al- Hazzaa 2018[8] Al-Nozha, 2007 [12]; Saudi-Arabia	Inactive: <600 METmin/wk Adults	Males: 94% Females: 98%	Included: PA data not overlapping with previously included data	Males: 6% Females: 2%
Yammine, 2016 [6]; Amine, 1996 [13]; UAE	Nil practice of PA – (Never participated in physical exercises**) Adults	Females: 48.5%	Excluded: Used a non-standard definition, could not be converted to PA prevalence measure	No
Yammine, 2016 [6]; Mehairi, 2013 [14]; UAE	Mild level of PA- (low score of PA**)	Males: 24.1 % Females: 45.1%	Excluded: PA data on the same population already included [14].	No
Yammine, 2016 [6]; Muhairi, 2013 [15]; UAE	Mild level of PA- (low score of PA**)	Males and females: 36.5 %	Excluded: PA data on the same population already included [15].	No
Yammine, 2016 [6]; Amine, 1996 [13]; UAE	Mild level of PA- (Mild participation in physical exercises**) Adults	Females: 47.1%	Excluded: PA data on the same population already included [13].	No
Yammine, 2016 [6]; Wasfi, 2008 [16]; UAE	Nil practice of PA-(did not practice PA at all**)	Males:10.7 % Females: 35.0 %	Excluded: PA data on the same population already included [16].	No
Yammine, 2016 [6]; Wasfi, 2008 [16]; UAE	Mild level of PA – (No vigorous activity or irregularly practiced vigorous exercise < 60 min/wk and < 30 min of moderate PA most days of the week**)	Males:51.6 % Females: 45.2%	Excluded: PA data on the same population already included [16].	No
Yammine, 2016 [6]; Wasfi, 2008 [16]; UAE	Nil practice of PA- (did not practice PA at all**) among Emiratis youth	Males and females: 18.9%	Excluded: PA data on the same population already included [16].	No
Ranasinghe, 2013[5]; Khuwaja, 2010 [17]; Pakistan	Inactivity: (< 30 minutes of moderate to vigorous activity most of the days (at least 4) in last 7 days**) Adults	Males: 52.1% Females: 69.8%	Included: PA data not overlapping with previously included data	Males: 47.9% Females: 30.2%
Sisson, 2008[3]; Guthold, 2008 [11]; Pakistan	Inactivity: < 3 days of VPA at 20 min/session or <5 days MPA at 30 min/session Adults	Males:12.8%† Females: 27.3%†	Included: PA data not overlapping with previously included data	Males: 87.2%‡ Females: 72.7%‡
Sisson, 2008[3], Sharara, 2018[9]; Guthold, 2008 [11]; Tunisia	Inactivity: < 3 days of VPA at 20 min/session or <5 days MPA at 30 min/session Adults	Males: 11.0%† Females: 18.2%†	Included: PA data not overlapping with previously included data	Males: 89.0%‡ Females: 81.8%‡
Sisson, 2008[3], Sharara, 2018 [9]; Guthold, 2008 [11]; UAE	Inactivity: < 3 days of VPA at 20 min/session or <5 days MPA at 30 min/session Adults	Males: 37.9%† Females: 56.7%†	Included: PA data not overlapping with previously included data	Males: 62.1%‡ Females: 43.2%‡
Sharara, 2018[9]; Al-Baghli, 2008[18] Saudi Arabia	No PA or mild PA (ordinary housework, walking)	Males and Females: 79.20%	Excluded: Used a non-standard definition, could not be converted to PA prevalence measure	No
Sharara, 2018[9]; Memish, 2014[19] Saudi Arabia	Neither moderate nor vigorous PA	Males and Females: 69.10%	Excluded: Used a non-standard definition, could not be converted to PA prevalence measure	No
Sharara, 2018[9]; Almurshed, 2009[20] Saudi Arabia	No exercise	Males and Females: 52%	Excluded: Used a non-standard definition, could not be converted to PA prevalence measure	No
Sharara, 2018[9]; Al-Hazzaa, 2018[8]; Al-Quaiz, 2009[21] Saudi Arabia	Not practicing in any regular sport and leisure time PA	Males and Females: 82.40%	Excluded: Used a non-standard definition, could not be converted to PA prevalence measure	No
Sharara, 2018[9]; Al-Senany, 2015[22] Saudi Arabia	Less than one-hour weekly activity	Males and Females: 69%	Excluded: Used a non-standard definition, could not be converted to PA prevalence measure	No
Sharara, 2018[9]; Amin, 2014[23] Saudi Arabia	<30 minutes /≥ 5 days/week	Males and Females: 80.0%	Included: PA data not overlapping with previously included data	Males and Females: 20.0%

Sharara, 2018[9]; Garawi, 2015[24] Saudi Arabia	<600 MET-minutes/week	Males and Females: 67%	Included: PA data not overlapping with previously included data	Males and Females: 33%
Sharara, 2018[9]; WHO-STEPS surveys, 2005[25] Saudi Arabia	<600 MET-minute per week	Males: 61.0% Females: 73.7%	Excluded: PA data from the same study already included	No
Sharara, 2018[9]; Maatoug, 2013[26] Tunisia	<150 min/week of moderate level of PA	Males and Females: 44.4%	Included: PA data not overlapping with previously included data	Males and Females: 55.6%
Sharara, 2018[9]; Guthold, 2008[11] UAE	Inactivity: < 3 days of VPA at 20 min/session or <5 days MPA at 30 min/session	Males: 40.0%	Included: PA data not overlapping with previously included data	Males: 60.0%
Sharara, 2018[9]; Guthold, 2008[11] UAE	Inactivity: < 3 days of VPA at 20 min/session or <5 days MPA at 30 min/session	Females: 59.0%	Included: PA data not overlapping with previously included data	Females: 41.0%
Sharara, 2018[9]; Abdulle, 2006[27] UAE	Less than one hour, <3 times per week	Males and Females: 39.4%	Excluded: Used a non-standard definition, could not be converted to PA prevalence measure	No
Sharara, 2018[9]; McIlvenny, 2000[28] UAE	No regular exercise	Males and Females:54.0%	Excluded: Used a non-standard definition, could not be converted to PA prevalence measure	No
Sharara, 2018[9]; Sabri, 2004[29] UAE	< 1 hour/week of sport	Males and Females: 47.5%	Excluded: Used a non-standard definition, could not be converted to PA prevalence measure	No
Sharara, 2018[9]; Ahmed, 2013[30] Kuwait	No deliberate non-work-related exercise outside the home such as walking, running or cycling	Males and Females: 68.4%	Excluded: Used a non-standard definition, could not be converted to PA prevalence measure	No
Sharara, 2018[9]; Al-Zenki, 2012[31] Kuwait	(Neither moderately nor very Active) Lightly active**	Males and Females: 57.9%	Excluded: Used a non-standard definition, could not be converted to PA prevalence measure	No
Sharara, 2018[9]; Alarouj, 2013[32] Kuwait	(Neither moderate nor vigorous PA) Moderate PA was considered if participants engaged in physical efforts that cause light sweating or a slight increase in breathing or heart rate; examples of moderate PA included, brisk walking, painting houses, gardening and climbing stairs**	Males and Females: 63%	Included: PA data not overlapping with previously included data	Males and Females: 37%
Sharara, 2018[9]; Naser Al-Isa, 2011[33] Kuwait	Not engaging in regular PA	Males and Females: 45%	Excluded: Used a non-standard definition, could not be converted to PA prevalence measure	No
Sharara, 2018[9]; WHO-STEPS survey, 2014[34] Kuwait	<600 MET-minutes/week	Males: 51.4% Females: 72.8%	Included: PA data not overlapping with previously included data	Male: 37.4% Females: 27.2%
Sharara, 2018[9]; Al-Nakeeb, 2015[35] Qatar	<840 MET-min/week	Males and Females: 50. 8%	Included: PA data not overlapping with previously included data	Males and Females: 49.2%
Sharara, 2018[9]; WHO-STEPS survey, 2012[36] Qatar	<600 MET-minutes/week	Males: 37.4% Females: 54.2%	Included: PA data not overlapping with previously included data	Males: 62.6% Females: 45.8%
Sharara, 2018[9]; Zindah, 2008[37] Jordan	Not engaging in moderate activity (resulting in light sweating, small increases in breathing or heart rate)	Males and Females: 51.8%	Excluded: Used a non-standard definition, could not be converted to PA prevalence measure	No
Sharara, 2018[9]; Centers for Disease Control, Prevention, 2003[38] Jordan	Less than having moderate: activity that caused light sweating and small increases in heart rate or breathing for 30 minutes	Males and Females: 47.4%	Excluded: Used a non-standard definition, could not be converted to PA prevalence measure	No
Sharara, 2018[9]; Kulwicki, 2001[39] Jordan	No exercise	Males and Females: 22.5%	Excluded: Used a non-standard definition, could not be converted to PA prevalence measure	No
Sharara, 2018[9]; Madanat, 2006[40] Jordan	<30 min of PA 3 or more days/week**	Males and Females: 81.5%	Excluded: Used a non-standard definition, could not be converted to PA prevalence measure	No
Sharara, 2018[9]; WHO-STEPS survey, 2007[41] Jordan	<600 MET-minutes/week	Males: 5.8% Females: 4.5%	Included: PA data not overlapping with previously included data	Males: 94.2% Females: 95.5%

Sharara, 2018[9]; Mohannad, 2008[42] Jordan	No activity that caused light sweating and small increases in heart rate or breathing	Males and Females: 58.7%	Excluded: Used a non-standard definition, could not be converted to PA prevalence measure	No
Sharara, 2018[9]; WHO-STEPS survey, 2011-12[43] Egypt	<600 MET-minutes/week	Males: 23.3% Females: 42%	Excluded: Total sample size not provided	No
Sharara, 2018[9]; Abolfotouh, 2007[44] Egypt	(No non-vigorous PA for at least 20 minutes or 3 times per week) No exercise **	Males and Females: 33.8%	Excluded: Used a non-standard definition, could not be converted to PA prevalence measure	No
Sharara, 2018[9]; Kamel, 2013[45] Egypt	Not active	Males and Females: 33.80%	Excluded: Used a non-standard definition, could not be converted to PA prevalence measure	No
Sharara, 2018[9]; Mahfouz, 2014[46] Egypt	No exercise	Males and Females: 63.80%	Excluded: Used a non-standard definition, could not be converted to PA prevalence measure	No
Sharara, 2018[9]; Mahfouz, 2014[46] Egypt	No exercise	Males and Females: 100%	Excluded: Used a non-standard definition, could not be converted to PA prevalence measure	No
Sharara, 2018[9]; WHO-STEPS survey, 2015[47] Iraq	<600 MET-minutes/week	Males: 34.9% Females: 60%	Included: PA data not overlapping with previously included data	Males: 65.1% Females: 40%
Sharara, 2018[9]; WHO-STEPS survey, 2009[48] Libya	<600 MET-minutes/week	Males: 36% Females: 51.7%	Included: PA data not overlapping with previously included data	Males: 64% Females: 48.3%
Sharara, 2018[9]; WHO-STEPS survey, 2009[49] Lebanon	<600 MET-minutes/week	Males: 52.2% Females: 40.3%	Included: PA data not overlapping with previously included data	Males: 47.8% Females: 59.7%
Sharara, 2018[9]; Al- Al-Tannir, 2008[50] Lebanon	Less than 3 days/week	Males and Females: 42.7%	Excluded: Used a non-standard definition, could not be converted to PA prevalence measure	No
Sharara, 2018[9]; Musharrafieh, 2008[51] Lebanon	Physical exercise for <0.5 h/week	Males and Females: 73.6%	Excluded: Used a non-standard definition, could not be converted to PA prevalence measure	No
Sharara, 2018[9]; Tamim, 2003[52] Lebanon	<3 hours/week	Males and Females: 64.3%	Included: PA data not overlapping with previously included data	Males and Females: 35.7%
Sharara, 2018[9]; Farah, 2015[53] Lebanon	Neither moderate intensity PA for at least 150 min per week or vigorous intensity PA for 75 min at least per week	Male and Female 76%	Included: PA data not overlapping with previously included data	Male and Female 24%
Sharara, 2018[9]; Tohme, 2005[54] Lebanon	Never engaging in any PA	Male 40.3%	Excluded: Used a non-standard definition, could not be converted to PA prevalence measure	No
Sharara, 2018[9]; WHO-STEPS survey, 2010-11[55] Palestine	<600 MET-minutes/week	Male and Female 46.5%	Included: PA data not overlapping with previously included data	Male and Female 53.5%
Sharara, 2018[9]; WHO-STEPS survey, 2010-11[55] Palestine	<600 MET-minutes/week	Males: 33.8% Females: 59.2%	Excluded: Total sample size not provided	No
Sharara, 2018[9]; Abdul-Rahim, 2003[56] Palestine	Occupation-related sedentarylight PA for men AND no exercise for women	Males and Females: 56.2%	Excluded: Used a non-standard definition, could not be converted to PA prevalence measure	No
Sharara, 2018[9]; Abu-Mourad, 2008[57] Palestine	No home exercise or sports	Males and Females: 78%	Excluded: Used a non-standard definition, could not be converted to PA prevalence measure	No
Sharara, 2018[9]; WHO-STEPS survey, 2003[58] Algeria	<600 MET-minutes/week	Males: 32.5% Females: 45.8%	Included: PA data not overlapping with previously included data	Males: 67.5% Females: 54.2%
Sharara, 2018[9]; WHO-STEPS survey, 2005-2006[59] Sudan	<600 MET-minutes/week	Malea: 75.9% Females: 94.8%	Included: PA data not overlapping with previously included data	Males: 24.1% Females: 5.2%
Sharara, 2018[9]; El Rhazi, 2011[60] Morocco	Less than 30 min of regular, moderate or intense PA on most days**	Males: 32.4% Females: 45.4%	Included: PA data not overlapping with previously included data	Males: 67.6% Females: 54.6%
Sharara, 2018[9]; Najdi, 2011[61] Morocco	<600 METmin/wk**	Males and Females: 16.5%	Included: PA data not overlapping with previously included data	Males and Females: 83.5%
Sharara, 2018[9]; Hamadeh, 2000[62] Bahrain	No exercise	Males and Females: 89.1%	Excluded: Used a non-standard definition, could not be converted to PA prevalence measure	No

Sharara, 2018[9]; Al Ali, 2011[63] Syria	Less than 15 min/ week of sport or brisk walking	Males and Females: 82.3%	Excluded: Used a non-standard definition, could not be converted to PA prevalence measure	No
Al Hazzaa, 2018 [8]; Al-Hazzaa, 1990[64] Saudi Arabia	No regular activity	Males: 78.2%	Excluded: Used a non-standard definition, could not be converted to PA prevalence measure	No
Al Hazzaa, 2018 [8]; Al-Gelban, 2008[65] Saudi Arabia	<3 times/week	Males: 83.3%	Excluded: Used a non-standard definition, could not be converted to PA prevalence measure	No
Al Hazzaa, 2018 [8]; Gawwad, 2008[66] Saudi Arabia	<3 days/week	Males: 72% Females: 78.9%	Excluded: Used a non-standard definition, could not be converted to PA prevalence measure	No
Mabry, 2016 [7]/Al Hazzaa, 2018 [8]; Khalaf A, 2013[67] Saudi Arabia	<150 min/week	Females: 37.6%	Included: PA data not overlapping with previously included data	Females: 62.4%
Al Hazzaa, 2018 [8]/ Mabry, 2016 [7]/Sharara, 2018[9]; Awadalla, 2014[68] Saudi Arabia	<600 METs-min per week	Males: 56.1% Females: 58.8%	Included: PA data not overlapping with previously included data	Males: 43.9% Females: 41.2%
Al Hazzaa, 2018 [8]; Majeed, 2015[69] Saudi Arabia	<1 time per week	Females: 72.5%	Excluded: Used a non-standard definition, could not be converted to PA prevalence measure	No
Al Hazzaa, 2018 [8]; Samara, 2015[70] Saudi Arabia	< 600 METs-min/ week	Females: 44%	Included: PA data not overlapping with previously included data	Females: 56%
Mabry, 2016[7]; Al Hazzaa, 2018 [8]; Allam, 2012[71] Saudi Arabia	<150 min/ week moderate activity or <60 min/ week vigorous activity	Males: 63.8% Females: 65%	Included: PA data not overlapping with previously included data	Males: 31.2% Females: 35%
Al Hazzaa, 2018 [8]; Al-Shahri, 1998[72] Saudi Arabia	No regular activity	Males: 76.5%	Excluded: Used a non-standard definition, could not be converted to PA prevalence measure	No
Al Hazzaa, 2018 [8]; Al Alwan, 2013[73] Saudi Arabia	No PA	Males: 41% Females: 45%	Excluded: Used a non-standard definition, could not be converted to PA prevalence measure	No
Al Hazzaa, 2018 [8]; Al Ateeq, 2014[74] Saudi Arabia	Active category: 30 min or more a day of moderate PA, 5 or more days a week. OR 20 min or more a day of vigorous PA, 3 or more days a week**	Males: 78.7% Females: 79%	Included: PA data not overlapping with previously included data	Males: 21.3% Females: 21%
Al Hazzaa, 2018 [8]; Taha, 1998[75] Saudi Arabia	<3 times/ week for 20 min	Males: 43.3% Females: 84.7%	Excluded: Used a non-standard definition, could not be converted to PA prevalence measure	No
Al Hazzaa, 2018 [8]; Abozaid, 2010[76] Saudi Arabia	<150 min./week moderate activity or <60 min/ week vigorous activity	Males: 46% Females: 47%	Included: PA data not overlapping with previously included data	Males: 54% Females: 53%
Al Hazzaa, 2018 [8]; Sharara, 2018; Amin, 2011[77] Saudi Arabia	<600 METs-min/week	Males: 50.5% Females: 44.8%	Included: PA data not overlapping with previously included data	Males: 49.5% Females: 55.2%
Al Hazzaa, 2018 [8]; Khalid, 1995[78] Saudi Arabia	No strenuous exercise for >= 3 times per week	Males: 59.4% Females: 99.5%	Excluded: Used a non-standard definition, could not be converted to PA prevalence measure	No
Al Hazzaa, 2018 [8]; Bin Horaib, 2013[79] Saudi Arabia	<30 min per day/ 5 days**	Males: 30.8%	Included: PA data not overlapping with previously included data	Males: 69.2%
Al Hazzaa, 2018 [8]; Al-Rafaee, 2001[80] Saudi Arabia	No regular activity	Males: 80.9%	Excluded: Used a non-standard definition, could not be converted to PA prevalence measure	No
Al Hazzaa, 2018 [8]; Al-Hazzaa, 2007[81] Saudi Arabia	<600 METs-min per week	Males: 43.7% Females: 34.3%	Included: PA data not overlapping with previously included data	Males: 56.3% Females: 65.7%
Al Hazzaa, 2018 [8]; Amin, 2014[82] Saudi Arabia	<600 METs-min per week	Males and Females: 73%	Included: PA data not overlapping with previously included data	Males and Females: 27%
Al Hazzaa, 2018 [8]; Al- Zalabani, 2015[83] Saudi Arabia	<600 METs-min per week of moderate, or <60 min / week vigorous activity	Males: 60.1% Females: 72.9%	Included: PA data not overlapping with previously included data	Males: 39.9% Females; 27.1%
Al Hazzaa, 2018 [8]; El Bcheraoui, 2016[84] Saudi Arabia	<150 MET-min/ week	Males and Females: 66.39%	Excluded: Used a non-standard definition, could not be converted to PA prevalence measure	No

Al Hazzaa, 2018 [8]; Albawardi, 2016[85] Saudi Arabia	<600 MET-s min per week	Females: 52.1%	Included: PA data not overlapping with previously included data	Females: 47.9%
Al Hazzaa, 2018 [8]; Mandil, 2016[86] Saudi Arabia	MVPA	Males and Females: 36.9%	Included: PA data not overlapping with previously included data	Males and Females: 63.1%
Al Hazzaa, 2018 [8]; Al-Mountashiri, 2017[87] Saudi Arabia	<30 min/day, 5 days/week	Males and Females: 86.7%	Included: PA data not overlapping with previously included data	Males and Females: 13.3%
YOUTH				
Sharara, 2018[9]; AlBuhairan, 2015[88] Saudi Arabia	Complete absence of exercise	Males and Females: 45.20%	Excluded: Used a non-standard definition, could not be converted to PA prevalence measure	No
Sharara, 2018[9]; Al-Muhaimeed, 2015[89] Saudi Arabia	Not engaging in sports	Males and Females: 27.30%	Excluded: Used a non-standard definition, could not be converted to PA prevalence measure	No
Sharara, 2018[9]; Al-Mutairi, 2015 [90] Saudi Arabia	No regular exercise	Males and Females: 31.90%	Excluded: Used a non-standard definition, could not be converted to PA prevalence measure	No
Sharara, 2018[9]; Al-Othman, 2012[91] Saudi Arabia	Based on weekly frequency: Never	Males and Females: 15.7%	Excluded: Used a non-standard definition, could not be converted to PA prevalence measure	No
Sharara, 2018[9]; Mahfouz, 2011 [92] Saudi Arabia	Less than 30 min of physical exercise during the previous week	Males and Females: 34.3%	Excluded: Used a non-standard definition, could not be converted to PA prevalence measure	No
Sharara, 2018[9]; GSHS, 2010[93] UAE	< 60 min per day on five or more days during the past seven days	Males and Females: 72.5%	Included: PA data not overlapping with previously included data	Males and Females: 27.5%
Sharara, 2018[9]; GSHS, 2010[94] UAE	< 60 min per day on five or more days during the past seven days	Males: 65.5% Females: 77.2%	Excluded: Total sample size not provided. Could not be included in the MA	No
Sharara, 2018[9]; GSHS, 2015[95] Oman	< 60 min per day on all seven days during the past seven days	Males and Females: 88.3%	Included: PA data not overlapping with previously included data	Males and Females: 11.7%
Sharara, 2018[9]; GSHS, 2015[95] Oman	< 60 min per day on all seven days during the past seven days	Males: 84.6% Females: 91.7%	Excluded: Total sample size not provided. Could not be included in the MA	No
Sharara, 2018[9]; Afifi, 2006[96] Oman	Engaging in physical activities <once per week, apart from school physical education	Males and Females: 66.3%	Excluded: Used a non-standard definition, could not be converted to PA prevalence measure	No
Sharara, 2018[9]; Musaiger, 2014[97] Bahrain	<5days/week of playing sport	Males and Females: 72.1%	Excluded: Used a non-standard definition, could not be converted to PA prevalence measure	No
Sharara, 2018[9]; GSHS, 2015[98] Kuwait	< 60 min per day on all seven days during the past seven days	Males and Females: 82.9%	Included: PA data not overlapping with previously included data	Males and Females: 17.1%
Sharara, 2018[9]; GSHS, 2015[98] Kuwait	< 60 min per day on all seven days during the past seven days	Males: 81% Females 84.9%	Excluded: Total sample size not provided. Could not be included in the MA	No
Sharara, 2018[9]; Shehab, 2005[99] Kuwait	Only performing normal daily routine with some recreational activities or walking slowly and doing no structured exercise	Males and Females: 71.3%	Excluded: Used a non-standard definition, could not be converted to PA prevalence measure	No
Yammine, 2016 [6]; Sharara, 2018[9]; GSHS, 2007[100] Libya	< 60 min per day on all seven days during the past seven days	Males: 78.5% Females: 88.4%	Included: Prioritized on the data from Guthold, 2010[10] as it includes higher sample size	Males: 21.5% Females: 11.6%
Yammine, 2016 [6]; Sharara, 2018[9]; GSHS, 2007[101] Djibouti	< 60 min per day on all seven days during the past seven days	Males: 81.2% Females: 90.8%	Included: Prioritized on the data from Guthold, 2010[10] as it includes higher sample size	Males: 81.2% Females: 90.8%
Yammine, 2016 [6]; Sharara, 2018[9]; GSHS, 2007[102]	< 60 min per day on all seven days during the past seven days	Males: 81.8% Females: 89%	Included: PA data not overlapping with previously included data	Males: 18.2% Females: 11%
Sharara, 2018[9]; Haddad, 2009 [103] Jordan	Not very physically nor moderately active	Males and Females: 4%	Excluded: Used a non-standard definition, could not be converted to PA prevalence measure	No
Yammine, 2016[6]; Sharara, 2018[9]; GSHS, 2010[104] Morocco	< 60 min per day on five or more days during the past seven days	Males and Females: 82.6%	Included: Prioritized on the data from Guthold, 2010[10] as it includes higher sample size	Males and Females: 17.4%
Yammine, 2016 [6]; Sharara, 2018[9]; GSHS, 2010[104]	< 60 min per day on five or more days during the past seven days	Males: 79.2% Females:	Included: PA data not overlapping with previously included data	Males: 79.2% Females:

Morocco		86.7%		86.7%
Yammine, 2016 [6]; Sharara, 2018[9]; GSHS, 2006[105] Egypt	< 60 min per day on all seven days during the past seven days	Males and Females: 90.6%	Included: PA data not overlapping with previously included data	Males and Females: 9.4%
Yammine, 2016 [6]; Sharara, 2018[9]; GSHS, 2006[105] Egypt	< 60 min per day on all seven days during the past seven days	Males: 85.5% Females: 96.6%	Excluded: Data overlapping with Guthold, 2010[10]	No
Sharara, 2018[9]; Salazar-Martinez, 2006[106] Egypt	Not engaged in sports	Males and Females: 62.30%	Excluded: Used a non-standard definition, could not be converted to PA prevalence measure	No
Sharara, 2018[9]; Shady, 2015[107] Egypt	< 4 hours/week	Males and Females: 65.5%	Excluded: Used a non-standard definition, could not be converted to PA prevalence measure	No
Sharara, 2018[9]; GSHS, 2011[108] Lebanon	< 60 min per day on five or more days during the past seven days	Males and Females: 65.4%	Included: PA data not overlapping with previously included	Males and Females: 34.6%
Sharara, 2018[9]; GSHS, 2011[108] Lebanon	< 60 min per day on five or more days during the past seven days	Males: 57.6% Females: 72.3%	Excluded: Total sample size not provided. Could not be included in the MA	No
Sharara, 2018[9]; Nasreddine, 2014[109] Lebanon	Moderate intensity activities included: playground activities, brisk walking, dancing, and bicycle riding. Higher intensity activities included: ball games, jumping rope, active games involving running and chasing, and swimming	Males and Females: 32.6%	Included: PA data not overlapping with previously included	Males and Females: 67.4%
Sharara, 2018[9]; GSHS, 2012[110] Iraq	< 60 min per day on five or more days during the past seven days	Males and Females: 80%	Included: PA data not overlapping with previously included	Males and Females: 20%
Sharara, 2018[9]; GSHS, 2012[110] Iraq	< 60 min per day on five or more days during the past seven days	Males: 74.7% Females: 86.4%	Excluded: Total sample size not provided. Could not be included in the MA	No
Sharara, 2018[9]; GSHS oPT Gaza, 2010[111] Palestine	< 60 min per day on five or more days during the past seven days	Males and Females: 75.8%	Included: PA data not overlapping with previously included	Males and Females: 24.2%%
Sharara, 2018[9]; GSHS oPT Gaza, 2010[111] Palestine	< 60 min per day on five or more days during the past seven days	Males: 74.7% Females: 86.4%	Excluded: Total sample size not provided. Could not be included in the MA	No
Sharara, 2018[9]; GSHS oPT West Bank, 2010[112] Palestine	< 60 min per day on five or more days during the past seven days	Males and Females: 81.7%	Included: PA data not overlapping with previously included	Males and Females: 18.3%
Sharara, 2018[9]; GSHS oPT West Bank, 2010[112] Palestine	< 60 min per day on five or more days during the past seven days	Males: 77.2% Females: 86.5%	Excluded: Total sample size not provided. Could not be included in the MA	No
Sharara, 2018[9]; Al Sabbah, 2007[113] Palestine	< 60 min/day, <5/7 days per week	Males and Females: 80%	Included: PA data not overlapping with previously included	Males and Females: 20%
Sharara, 2018[9]; Jildeh, 2011[114] Palestine	<5 days per week active in sport for at least one hour + mode of transport to and from school**	Males and Females: 77.6%	Included: PA data not overlapping with previously included	Males and Females: 13.5%
Sharara, 2018[9]; GSHS, 2011[115] Qatar	< 60 min per day on five or more days during the past seven days	Males and Females: 85%	Included: PA data not overlapping with previously included	Males and Females: 15%
Sharara, 2018[9]; GSHS, 2011[115] Qatar	< 60 min per day on five or more days during the past seven days	Males: 80.1% Females: 89.8%	Excluded: Total sample size not provided. Could not be included in the MA	No
Sharara, 2018[9]; GSHS, 2012[116] Sudan	< 60 min per day on five or more days during the past seven days	Males and Females: 89%%	Included: PA data not overlapping with previously included	Males and Females: 11%
Sharara, 2018[9]; GSHS, 2012[116] Sudan	< 60 min per day on five or more days during the past seven days	Males: 89.1% Females: 88.8%	Excluded: Total sample size not provided. Could not be included in the MA	No
Sharara, 2018[9]; Moukhyer, 2008[117] Sudan	Not engaging in sports activities	Males and Females: 33.4%	Excluded: Used a non-standard definition, could not be converted to PA prevalence measure	No

Sharara, 2018[9]; GSHS, 2010[118] Syria	< 60 min per day on five or more days during the past seven days	Males and Females: 84.9%	Included: PA data not overlapping with previously included	Males and Females: 15.1%
Sharara, 2018[9]; GSHS, 2010[118] Syria	< 60 min per day on five or more days during the past seven days	Males: 81.9% Females: 89.2%	Excluded: Total sample size not provided. Could not be included in the MA	No
Sharara, 2018[9]; GSHS, 2008[119] Yemen	< 60 min per day on all seven days during the past seven days	Males and Females: 84.8%	Included: PA data not overlapping with previously included	Males and Females: 15.2%
Sharara, 2018[9]; GSHS, 2008[119] Yemen	< 60 min per day on all seven days during the past seven days	Males: 83.2% Females: 87.7%	Excluded: Total sample size not provided. Could not be included in the MA	No
Sharara, 2018[9]; GSHS, 2008[120] Tunisia	< 60 min per day on all seven days during the past seven days	Males and Females: 81.5%	Included: PA data not overlapping with previously included	Males and Females: 18.5%
Sharara, 2018[9]; GSHS, 2008[120] Tunisia	< 60 min per day on all seven days during the past seven days	Males: 73.8% Females: 89%	Excluded: Total sample size not provided. Could not be included in the MA	No
Sharara, 2018[9]; Nouira, 2014[121] Tunisia	Doesn't do recommended PA	Males and Females: 88.1%	Excluded: Used a non-standard definition, could not be converted to PA prevalence measure	No
Sharara, 2018[9]; Aounallah-Skhiri, 2012[122] Tunisia	< 3 Mets	Males and Females: 29.4%	Excluded: Used a non-standard definition, could not be converted to PA prevalence measure	No
Sharara, 2018[9]; Abbes, 2016[123] Algeria	Not engaged in sports	Males and Females: 92.8%	Excluded: Used a non-standard definition, could not be converted to PA prevalence measure	No
Sharara, 2018[9]; Abbes, 2016[123] Algeria	>3hrs watching TV, video games, computers **	Males and Females: 71.67%	Excluded: Used a non-standard definition, could not be converted to PA prevalence measure	No
Mabry, 2016 [7]; Sharara, 2018[9]; Al-Hazzaa, 2018[8]; Al-Hazzaa, 2011 [124] Saudi Arabia	<1680 METs-min per week	Males: 44.5% Females: 78.1%	Included: PA data not overlapping with previously included	Males: 55.5% Females: 21.9%
Al-Hazzaa, 2018[8]; Al-Hazzaa, 2007[125] Saudi Arabia	<10000 step counts per day	Males: 72.9% Females: 81.4%	Excluded: Used a non-standard definition, could not be converted to PA prevalence measure	No
Al-Hazzaa, 2018[8]; Al-Hazzaa, 1993[126] Saudi Arabia	Daily heart rate <159 bpm for atleast 20 min/day	Males: 85%	Included: PA data not overlapping with previously included	Males: 15%
Al-Hazzaa, 2018[8] ; Al Hazzaa, 2002[127] Saudi Arabia	Daily heart rate <140 bpm for atleast 30min/day	Males: 57.1%	Excluded: PA data overlapping with previously included data	No
Sisson, 2008[3]; Al-Hazzaa, 2018[8]; Al Hazzaa, 2007[128] Saudi Arabia	<13000 step counts	Male: 47.1%	Included: PA data not overlapping with previously included	Males: 52.9%
Al-Hazzaa, 2018[8]; Al-Kutbe, 2017[129] Saudi Arabia	<10000 step counts/ day	Females: Low 6757 step/day (20.2 min/ day of MVPA)	Excluded: 1) PA data provided in mean steps number (no prevalence data) 2) Used a non-standard definition, could not be converted to PA prevalence measure	No
Al-Hazzaa, 2018[8]; Al-Rukban, 2003[130] Saudi Arabia	<30 min for <4 days/ week	Males: 72.3%	Excluded: Used a non-standard definition, could not be converted to PA prevalence measure	No
Al-Hazzaa, 2018[8]; Taha, 2008[131] Saudi Arabia	Any bodily movement produced by skeletal muscles that resulted in energy expenditure above the basal level for at least 20 minutes per session** <3 times/ week	Males: 54.4% Females: 66.3%	Excluded: Used a non-standard definition, could not be converted to PA prevalence measure	No
Al-Hazzaa, 2018 [8]; Mahfouz, 2012[132] Saudi Arabia	<30 min of PA in previous week	Males: 25.7% Females: 42.9%	Excluded: Used a non-standard definition, could not be converted to PA prevalence measure	No
Al-Hazzaa, 2018[8]; Alzahrani, 2014[133] Saudi Arabia	<60 min, 5 day/week	Males: 63.7%	Included: PA data not overlapping with previously included	Males: 36.3%

Al-Hazzaa, 2018 [8]; Alsubaie, 2015[134] Saudi Arabia	Moderate to vigorous PA for 60 min/ day** <5 days a week	Males: 20.1%	Included: PA data not overlapping with previously included	Males: 79.9%
Al-Hazzaa, 2018[8]; Bajamal, 2017[135] Saudi Arabia	Sum of moderate to vigorous activity score during the past 7 days**	Females: low	Excluded: Used a non-standard definition, could not be converted to PA prevalence measure	No
Al-Hazzaa, 2018[8]; Al-Raddadi, 2018[136] Saudi Arabia	<150 min per week	Females: 86.1%	Included: PA data not overlapping with previously included	Females: 13.9%
Al-Hazzaa, 2018[8]; El Bcheraoui, 2013 [84] Saudi Arabia	<420 MET-min/week	Males and Females: 55.71%	Excluded: Used a non-standard definition, could not be converted to PA prevalence measure	No

Notes: From the total number of 7 studies [11-17] with physical inactivity data (reported alone or with PA data), only 2 studies [11,17], with physical inactivity data not overlapping with an included PA data, were included. A total of eight data points on physical inactivity, from these 2 studies of Guthold, 2008 and Khuwaja, 2010, in three different MENA countries were added to PA activity data and used for the meta-analyses among ADULTS.
No converted data from physical inactivity to PA among YOUTH was included. The 2 publications have reported nil and mild/poor PA levels (considered as physical inactivity) among youth was Wasfi, 2007 and Mehairi, 2013 [14,15]. These later reported also acceptable/moderate and high/good physical activity levels among youth that were already included in the PA table 3.
Physical inactivity prevalence measures not converted to PA have already been included PA data and were not excluded from the quantitative data.
† Prevalence measure reported in the original study
‡ Prevalence measure calculated using the original study data
Abbreviations:
UAE: United Arab Emirates
MET Metabolic Equivalent of task
min Minutes
Wk week
PA Physical activity
d day
d-1 per day
MPA Moderate Physical Activity
VPA Vigorous Physical Activity

Table S6: List of excluded overlapping studies and combined outcome measures.

**List of overlapping publications, among adults, excluded from the meta-analyses** (number of excluded=13):
1) The publication of Al-Nozha et al 2007 [12] was included in the SR of Sisson et al, 2008 [3], Al Hazzaa, 2018 [8], Sharara, 2018 [9] Mabry et al 2010 [4], and Mabry et al, 2016 [7]. Data from Al-Nozha et al 2007 [12] was used only one time in the same meta-analysis group. (n=4)<br>2) The publication of Al-Hazzaa et al 2007 [81] was included in the SR of Mabry et al 2010 [4] and Mabry et al, 2016 [7]. Data from Al-Hazzaa et al 2007 [81] was used only one time in the same meta-analysis group. (n=1)<br>3) The publication of Amin, 2011[77] was included in the SR of Al Hazzaa, 2018 [8], Sharara, 2018 [9]. Data from Amin, 2011[77] was used only one time in the same meta-analysis group. (n=1)<br>4) The publication of Awadalla, 2014 [68] was included in the SR of Mabry et al, 2016 [7] Al Hazzaa, 2018 [8], Sharara, 2018 [9]. Data from Awadalla, 2014 [68] was used only one time in the same meta-analysis group. (n=2)<br>5) The publication of Ministry of Health, Saudi Arabia, 2005[25] was included in the SR of Mabry et al 2010 [4], Sharara, 2018 [9]. Data from Ministry of Health, Saudi Arabia, 2005 [25] was used only one time in the same meta-analysis group. (n=1)<br>6) The publication of Guthold, 2008 [11] (Tunisia) was included in the SR Sharara, 2018 [9] and Sisson et al, 2008 [3]. The publication of Guthold, 2008 [11] (UAE) was included in the SR Sharara, 2018 [9] and Sisson et al, 2008 [3]. Data from Guthold, 2008[11] was used only one time in the same meta-analysis group. (n=1)<br>7) The publication of Khalaf A, 2013[67] was included in the SR Mabry, 2016 [7] and Al Hazzaa, 2018 [8]; Data from Khalaf A, 2013[67] was used only one time in the same meta-analysis group. (n=1)<br>8) The publication of Allam, 2012[71] was included in the SR Mabry, 2016 [7] and Al Hazzaa, 2018 [8]; Data from Allam, 2012[71] was used only one time in the same meta-analysis group. (n=1)<br>9) The publication of Bener, 2004 [137] was included in the SR Mabry, 2010[4] and Al Sharara, 2018[9]; Data from Bener, 2004 [137] was used only one time in the same meta-analysis group. (n=1)<br>Note: The publication was used only one time in the same subgroup. In case of overlapping publications, the publication with the most comprehensive extracted data and the highest sample size was prioritized.

**List of overlapping data, among <u>adults,</u> excluded from the meta-analyses** (only one study on the same population was included, number of excluded=2):
1. The PA data of Al-Mahroos et al 2001[138] was overlapping with PA data used in Al-Mahroos et al 1998 [139]. ter [139] was retained for the analysis as the used definition for physical activity participation is concordant with the standard definition. (n=1)<br>2. Both Al Thani, 2015[140] and WHO-STEPS survey, 2012[141] included data on PA from WHO-STEPS survey on Qatari females. Only data from WHO-STEPS survey, 2012, including a higher sample size, was included (n=1)
**List of publications using non-standard definitions, among <u>adults,</u> excluded from the meta-analyses** (number of excluded= 37):
1) Amine, 1996 [13] was excluded because they used a non-standard definition of physical activity.<br>2) Al-Baghli, 2008[18] was excluded because they used a non-standard definition of physical activity.<br>3) Memish, 2014[19] was excluded because they used a non-standard definition of physical activity.<br>4) Almurshed, 2009[20] was excluded because they used a non-standard definition of physical activity.<br>5) Al-Quaiz, 2009[21] was excluded because they used a non-standard definition of physical activity.<br>6) Al-Senany, 2015[22] was excluded because they used a non-standard definition of physical activity.<br>7) Abdulle, 2006[27] was excluded because they used a non-standard definition of physical activity.<br>8) McIlvenny, 2000[28] was excluded because they used a non-standard definition of physical activity.<br>9) Sabri, 2004[29] was excluded because they used a non-standard definition of physical activity.<br>10) Ahmed, 2013[30] was excluded because they used a non-standard definition of physical activity.<br>11) Al-Zenki, 2012[31] was excluded because they used a non-standard definition of physical activity.<br>12) Naser Al-Isa, 2011[33] was excluded because they used a non-standard definition of physical activity.<br>13) Zindah, 2008[37] was excluded because they used a non-standard definition of physical activity.<br>14) Centers for Disease Control, Prevention, 2003[38] was excluded because they used a non-standard definition of physical activity.<br>15) Kulwicki, 2001[39] was excluded because they used a non-standard definition of physical activity.<br>16) Madanat, 2006[40] was excluded because they used a non-standard definition of physical activity.<br>17) Mohannad, 2008[42] was excluded because they used a non-standard definition of physical activity.<br>18) Abolfotouh, 2007[44] was excluded because they used a non-standard definition of physical activity.<br>19) Kamel, 2013[45] was excluded because they used a non-standard definition of physical activity.<br>20) Mahfouz, 2014[46] was excluded because they used a non-standard definition of physical activity.<br>21) Al-Tannir, 2008[50] was excluded because they used a non-standard definition of physical activity.<br>22) Musharrafieh, 2008[51] was excluded because they used a non-standard definition of physical activity.<br>23) Tohme, 2005[54] was excluded because they used a non-standard definition of physical activity.<br>24) Abdul-Rahim, 2003[56] was excluded because they used a non-standard definition of physical activity.<br>25) Abu-Mourad, 2008[57] was excluded because they used a non-standard definition of physical activity.<br>26) Hamadeh, 2000[62] was excluded because they used a non-standard definition of physical activity.<br>27) Al Ali, 2011[63] was excluded because they used a non-standard definition of physical activity.<br>28) Al-Hazzaa, 1990[64] was excluded because they used a non-standard definition of physical activity.<br>29) Al-Gelban, 2008[65] was excluded because they used a non-standard definition of physical activity.<br>30) Gawwad, 2008[66] was excluded because they used a non-standard definition of physical activity.<br>31) Majeed, 2015[69] was excluded because they used a non-standard definition of physical activity.<br>32) Al-Shahri, 1998[72] was excluded because they used a non-standard definition of physical activity.<br>33) Al Alwan, 2013[73] was excluded because they used a non-standard definition of physical activity.<br>34) Taha, 1998[142] was excluded because they used a non-standard definition of physical activity.<br>35) Khalid, 1995[78] was excluded because they used a non-standard definition of physical activity.<br>36) Al-Rafaee, 2001[80] was excluded because they used a non-standard definition of physical activity.<br>37) El Bcheraoui, 2016 [84] was excluded because they used a non-standard definition of physical activity.
**List of overlapping publications, among <u>youth</u>, excluded from the meta-analyses** (number of excluded=2):

1) The publication of Al-Hazzaa, 2011[124] was included in the SR of Al Hazzaa, 2018 [8], Sharara, 2018 [9], Mabry et al, 2016 [7]. Data from Al-Hazzaa, 2011[124] was used only one time in the same meta-analysis group. (n=2)

List of overlapping data, among youth, excluded from the meta-analyses (only one study on the same population was included, number of excluded=6):

1) The PA data from Al-Hazzaa, 2014 [143], Al-Hazzaa, 2011 [124], Al-Hazzaa, 2013 [144], Al-Hazzaa, 2013 [145], included in the SR of Mabry et al, 2016 [7], were overlapping. Only data from Al-Hazzaa, 2011 [124], with the highest sample size, were retained. (n=3)
2) The PA data from Al-Nakeeb, 2012 [146] and Al-Nuaim, 2012 [147], included in the SR of Mabry, 2016, were overlapping. Only data from Al-Nuaim, 2012 [147], with the highest sample size, were retained. (n=1)
3) The PA data from Mehairi, 2013 [14] and Muhairi, 2013 [15], included in the SR of Yammine, 2016[6] were overlapping. Only data from Mehairi, 2013 [14] with males and females stratified results and the highest sample size, were retained. (n=1)
4) Guthold, 2010[10] is overlapping with data from GSHS Libya, 2007[100], GSHS Djibouti, 2007[101], GSHS Morocco, 2010[104]. Only data from GSHS Libya, 2007 [100], GSHS Djibouti, 2007[101], GSHS Morocco, 2010[104], with males and females stratified results and the highest sample size. (n=0)
5) GSHS Egypt 2006[105] is overlapping with data from Guthold, 2010[10]. Data from Guthold, 2010[10] for Egypt with stratified sample sizes were retained. (n=0)
6) The PA data from Hazzaa, 2002[127] is overlapping with data from Al-Hazzaa, 1993[126]. Only data from Al-Hazzaa, 1993[126] with males stratified results and the highest sample size, were retained (n=1)

List of publications using non-standard definitions, among youth, excluded from the meta-analyses (number of excluded= 22):

1) AlBuhairan, 2015[88] was excluded because they used a non-standard definition of physical activity.
2) Al-Muhaimeed, 2015[89]was excluded because they used a non-standard definition of physical activity.
3) Al-Mutairi, 2015 [90] was excluded because they used a non-standard definition of physical activity.
4) Al-Othman, 2012[91] was excluded because they used a non-standard definition of physical activity.
5) Mahfouz, 2011 [92] was excluded because they used a non-standard definition of physical activity.
6) Afifi, 2006[96] was excluded because they used a non-standard definition of physical activity.
7) Musaiger, 2014[97] was excluded because they used a non-standard definition of physical activity.
8) Shehab, 2005[99] was excluded because they used a non-standard definition of physical activity.
9) Haddad, 2009 [103] was excluded because they used a non-standard definition of physical activity.
10) Salazar-Martinez, 2006[106] was excluded because they used a non-standard definition of physical activity.
11) Shady, 2015[107] was excluded because they used a non-standard definition of physical activity.
12) Moukhyer, 2008[117] was excluded because they used a non-standard definition of physical activity.
13) Nouira, 2014[121] was excluded because they used a non-standard definition of physical activity.
14) Aounallah-Skhiri, 2012[122] was excluded because they used a non-standard definition of physical activity.
15) Abbes, 2016[123] was excluded because they used a non-standard definition of physical activity.
16) Al-Hazzaa, 2007[125] was excluded because they used a non-standard definition of physical activity.
17) Al-Kutbe, 2017[129] was excluded because they used a non-standard definition of physical activity.
18) Al-Rukban, 2003[130] was excluded because they used a non-standard definition of physical activity.
19) Taha, 2008[142] was excluded because they used a non-standard definition of physical activity.
20) Mahfouz, 2012 [132] was excluded because they used a non-standard definition of physical activity.
21) Bajamal, 2017[135] was excluded because they used a non-standard definition of physical activity.
22) El Bcheraoui, 2013[84] was excluded because they used a non-standard definition of physical activity.

Abbreviations:

PA Physical activity

SR Systematic review

Table S7: Quality assessment (QA) checklist for the original studies (Maximum score of 21).

QA_Criteria's name	QA_Criteria's scoring<br>**(Max score of 3/ criteria)**	QA_Criteria's definition
QA_Population_characteristics<br><br>***Used variables from the reporting tables: type population, age groups***	**0** Not defined	Missing all characteristics listed below
	**3** Clearly defined<br>(**1** point for each population characteristic)	The following characteristics were specified:<br>a) Nationality (**1 point**)<br>b) Population type (general or specific population of students, nurses,) (**1 point**)<br>c) Adults/youth/ age group/adolescents (**1 point**)
QA_Outcome_definition<br><br>***Used variables from the reporting tables: Physical activity definition in the study***	**0** Not defined	No definition
	**1** Unclear non-standard definition	Any incomplete /partial definition of the outcome without details on the time and duration<br>No specification of the included sedentary/ sitting activities using or not mean time as final measure,<br>Didn't used mean time/day as final measure,<br>Any sedentary behavior outcome definition using a cut-off to report the prevalence of the sedentary behavior (e.g. vigorous physical activity level: high score of physical activity, sedentary (6h+))[148]
	**2** Clear non-standard definition	Physical activity:<br>The stated definition of physical activity or inactivity is clear (time and duration) but not consistent with international recommendations<br>Example: number of steps/day [149,150]<br>Even if some studies have reported that the steps count could be equivalent to > 60 minutes moderate activity in youth [151-155], they do not all agree on the threshold steps count. The reason we consider this definition non-standard.<br><br>Sedentary behavior<br>Any clear definition (time and duration) of only one type of sedentary behavior using mean time/day or week (e.g. mean time spent watching TV only)[148]
	**3** Standard definition (Clearly stated definition of PA/sedentary behavior/ inactivity consistent with international recommendations for the general population from the WHO and similar organizations with published physical activity guidelines) [41,156,157]. Any used measure should be converted in international units (min or hrs/d, min or hrs/wk, METS, or PAL)<br><br>General population exclude pregnant and lactating women	Physical activity<br>*Infants aged 3-5:*<br>3 hours per day of high-impact, dynamic, short duration exercise [41]<br><br>*Youth aged 5–17:*[157]<br>= or > 60 minutes of moderate to vigorous intensity physical activity per day [41,157]<br><br>*Adults aged 18-64:* [157]<br>= or >150 minutes of moderate-intensity aerobic PA/week<br>**OR**<br>= or >75 minutes of vigorous-intensity aerobic physical activity/week<br>**OR**<br>An equivalent combination of moderate and vigorous-intensity activity<br>**OR**<br>= or > 30 minutes of moderate to vigorous activity, 3 or more days/week [156]

		**OR**<br>> 30 minutes of moderate-intensity physical activity on most days of the week [158]<br>**OR**<br>Daily PAL $_{\text{Active or moderately active lifestyle}}$ = 1.70-1.99 [156]<br>Daily PAL $_{\text{Vigorous or vigorously active lifestyle}}$= 2.00-2.40 [156]<br>**OR**<br>500 to 1,000 MET-minutes per week of aerobic physical activity [41]<br>**OR**<br>3 or more days of vigorous-intensity activity of at least 20 minutes per day[148]<br>**OR**<br>5 or more days of moderate-intensity activity and/or walking of at least 30 minutes per day[148]<br>**OR**<br>5 or more days of any combination of walking, moderate-intensity or vigorous intensity activities achieving a minimum [148]<br>**OR**<br>Total physical activity of at least 600 MET-minutes/week. [148,159,160]<br><br>Physical inactivity:<br>An absence of physical activity or exercise [157]<br>**OR**<br>Insufficient amounts of PA (under the recommended levels (minutes or hours/d, minutes or hours/week, METS, PAL)) [161,162]<br>**OR**<br>Not achieving 180 minutes of PA of any intensity per day among 1-4 years [45] [163]
	Sedentary behavior and sedentary activities are similar but not synonymous<br>Sedentary activity includes standing but not sedentary behavior<br><br>Data on sitting should be reported as median values and interquartile ranges. To-date there are few data on sedentary (sitting) behaviors and no well-accepted thresholds for data presented as categorical levels.[148]	Sedentary behavior:<br>Minutes spent in sedentary activities on a typical day. [159]<br>Any waking behavior with an energy expenditure of ≤1.5 METs, such as sitting and reading or watching television, or standing quietly.<br>Sedentary activities include: Minutes spent in sedentary activities on a typical day<br>Sedentary time, screen time, sitting time, etc.[41,161]<br>**OR**<br>Daily PAL value 1.40-1.69 [156]<br><br>To be considered clear definition of sedentary behavior the following information should be reported:<br>1. **Mean total time spent sitting** including sitting at work, in an office, reading, watching television, using a computer, doing hand craft like knitting, resting etc. Should not include time spent sleeping. [148]<br>2. **Period of sedentary** behavior assessment is usually per day [148] (Could be deduced from any specified period)
QA_Measurement_methodology	**0** Not defined	The used instrument was not defined

**Used variables from the reporting tables: Physical activity instrument or used items**	**1** A non-validated questionnaire (subjective)	Use of a non-validated and non-standard questionnaire not included in the list of the 83 validated questionnaires [164] available at https://epi.grants.cancer.gov/paq/validation.html
	**2** Validated questionnaire (subjective)	Use of a validated standard physical activity or inactivity/sedentary behavior instrument included in the list of the 83 validated questionnaires [164] available at https://epi.grants.cancer.gov/paq/validation.html
	**3** A gold standard (objective)	Used and objective instrument, such as, accelerometer (gold standard) or pedometer
QA_Setting<br><br>**Used variables from the reporting tables: Setting**	**0** Not defined/ not clearly defined	No definition
	**3** National coverage **c**learly defined	Local or national geographical coverage clearly defined: name or number of the city or governorate, university, school, households<br>Urban or Rural
QA_Timing<br><br>**Used variables from the reporting tables: Years of data collection**	**0** Not defined	Data collection time not stated
	**3** Clearly defined	Data collection time stated
QA_Sampling<br><br>**Used variables from the reporting tables: Sampling method**	**0** Not defined	Not stated
	**1** Unclear	Self-selection or unclear method [165]
	**2** Non-random sampling	Clearly defined non-random sampling method (e.g. Convenient)
	**3** Random sampling	Clearly defined random sampling
QA_Response_rate<br><br>**For each reported data point if only global response rate is reported, the response rate per strata (male/female) would be missing**<br><br>**Used variables from the reporting tables: Response rate (%)**	**0** Not defined	The response rate was not reported for the study population for which the prevalence was reported e.g. the males and female's response rate are different than the response rate for the whole study population.
	**1** <49%	Low response rate
	**2** 50-79%	Acceptable response rate in this context
	**3** >=80%	Standard response rate [165]

Notes:
The quality appraisal was based on the reported primary studies data as reported in the SR and the original primary study.
Only PA recommendations for the general population were considered.
Populations from Tunisia and Pakistan are predominantly national. The specification of the nationality was not used as criteria to score the population characteristics in these countries.
Low levels of PA were scored according to the physical inactivity recommended definitions.
Metabolic equivalent of task (MET): "A unit that represents the metabolic cost of physical activity. One MET is the rate of energy expenditure while sitting at rest, which, for most people approximates an oxygen uptake of 3.5 ml per kg per min. The energy expenditure of other activities is expressed in multiples of METs. For example, for the average adult, sitting and reading requires about 1.3 METs, strolling or walking slowly requires about 2.0 METs, and running at 5 miles per hour requires about 8.3 METS" [41].
Physical activity level (PAL): "the total energy expenditure (TEE) for 24 hours expressed as a multiple of basal metabolic rate (BMR), and calculated as TEE/BMR for 24 hours" [156].
Data on sitting should be reported as median values and interquartile ranges. To-date there are no well-accepted thresholds for data presented as categorical levels [148]. Also, there is no standard guidelines setting the minimum sedentary time required to classify a person as having a sedentary behaviour. The reported sedentary outcome was scored clear only if the mean or median total sedentary time of sedentary activities with an energy expenditure of ≤1.5 METs' (such as, mean time spent watching TV, reading, screen time, sitting time, or sedentary time without specification) was reported. Studies with sedentary prevalence measures, using any threshold, were considered unclear.
Quality assessment criteria were not applied to odd ratios and prevalence ratios and pooled prevalence.
Abbreviations: QA: Quality assessment.

Table S8: The prevalence of physical activity among the adult general population of the included MENA countries.

Systematic review source	Study source	Study design	Sampling method	Setting	Years of data collection	Population	Physical activity definition/study	Physical activity instrument or used items	Instrument administration	Age group (years)	Prevalence of physical activity (%)	Gender	Sample size	♀/♂ (%)	Res p-rate (%)	QA score (/21)
Saudi-Arabia (number of studies = 11)																
Sisson, 2008[3]	Al-Nozha, 2007[12]	CS**	MCRS**	National level	1995-00	Saudi participants in the Public-School Coronary Artery Disease Study	Active ≥600 METmin/wk	PA questionnaire **	Self-reported (Face-to-face interviews**)	30-70	3.9**	Male and Female	17395 **	Equal **	N/S	16
Sisson, 2008[3]	Al-Nozha, 2007[12]	CS**	MCRS**	National level	1995-00	Saudi participants in the Public-School Coronary Artery Disease Study	Active ≥600 METmin/wk	PA questionnaire **	Self-reported (Face-to-face interviews**)	30-70	6	Male	8297**	48**	N/S	16
Sisson, 2008[3]	Al-Nozha, 2007[12]	CS**	MCRS**	National level	1995-00	Saudi participants in the Public-School Coronary Artery Disease Study	Active ≥600 METmin/wk	PA questionnaire **	Self-reported (Face-to-face interviews**)	30-70	2	Female	9098**	52**	N/S	16
Mabry, 2010[4]	Ministry of Health, Saudi Arabia, 2005[25]	World Health Survey (CS**)	MCRS**	National level	2004**	Saudi general population	At least 600 MET-min/wk of vigorous or moderate activity	GPAQ	Face-to-face interviews	25-64	32.65*	Male and Female	4758	Equal	97.7	20
Mabry, 2010[4]	Ministry of Health, Saudi Arabia, 2005[25]	World Health Survey (CS**)	MCRS**	National level	2004**	Saudi general population	At least 600 MET-min/wk of vigorous or moderate activity	GPAQ	Face-to-face interviews	25-64	39.0	Male	2284*	48	N/S	17
Mabry, 2010[4]	Ministry of Health, Saudi Arabia, 2005[25]	World Health Survey (CS**)	MCRS**	National level	2004**	Saudi general population	At least 600 MET-min/wk of vigorous or moderate activity	GPAQ	Face-to-face interviews	25-64	26.3	Female	2474*	52	N/S	17
Mabry, 2010[4]	Al-Nozha, 2007 [12]	CS**	MCRS	National level	1995-00**	Saudi general population	Active 600 or more MET min/wk	Instrument included items regarding	Face-to-face interviews	30-70	3.9*	Male and	17395	Equal	99	19

Systematic review source	Study source	Study design	Sampling method	Setting	Years of data collection	Population	Physical activity definition/study	Physical activity instrument or used items	Instrument administration	Age group (years)	Prevalence of physical activity (%)	Gender	Sample size	♀/♂ (%)	Resp-rate (%)	QA score (/21)
								sports and leisure activities				Female				
Mabry, 2010[4]	Al-Nozha, 2007 [12]	CS**	MCRS	National-level	1995-00**	Saudi general population	Active 600 or more MET-min/wk	Instrument included items regarding sports and leisure activities	Face-to-face interviews	30-70	6.1	Male	8350*	48	N/S	16
Mabry, 2010[4]	Al-Nozha, 2007 [12]	CS**	MCRS	National-level	1995-00**	Saudi general population	Active 600 or more MET-min/wk	Instrument included items regarding sports and leisure activities	Face-to-face interviews	30-70	1.9	Female	9045*	52	N/S	16
Mabry, 2010[4]	Al-Hazzaa, 2007 [81]	N/S	RS	Riyadh	2003**	Saudi general population	At least 600 MET-min/wk of vigorous or moderate activity	IPAQ (Short version**)	Telephone interviews	15-78	59.5*	Male and Female	1064	♂>♀	66	19
Mabry, 2010[4]	Al-Hazzaa, 2007 [81]	N/S	RS	Riyadh	2003**	Saudi general population	At least 600 MET-min/wk of vigorous or moderate activity	IPAQ (Short version**)	Telephone interviews	15-78	56.3	Male	702*	66	N/S	17
Mabry, 2010[4]	Al-Hazzaa, 2007 [81]	N/S	RS	Riyadh	2003**	Saudi general population	At least 600 MET-min/wk of vigorous or moderate activity	IPAQ (Short version**)	Telephone interviews	15-78	65.7	Female	362*	34	N/S	17
Mabry, 2016[7]	Al-Hazzaa, 2007 [81]	Population-based survey	Simple RS**	Riyadh**	2003**	Saudi**adult general population	150 min of moderate-intensity PA/wk	IPAQ short version	Telephone interviews**	15-78	59.40	Male and Female	1064	♂>♀**	66**	19
Mabry, 2016[7]	Al-Hazzaa, 2007 [81]	Population-based survey	Simple RS**	Riyadh**	2003**	Saudi**adult general population	150 min of moderate-intensity PA/wk	IPAQ short version	Telephone interviews**	15-78	56.3	Male	702**	66**	N/A	17
Mabry, 2016[7]	Al-Hazzaa, 2007 [81]	Population-based survey	Simple RS**	Riyadh**	2003**	Saudi**adult general population	150 min of moderate-intensity PA/wk	IPAQ short version	Telephone interviews**	15-78	65.7	Female	362**	34**	N/A	17

Systematic review source	Study source	Study design	Sampling method	Setting	Years of data collection	Population	Physical activity definition/study	Physical activity instrument or used items	Instrument administration	Age group (years)	Prevalence of physical activity (%)	Gender	Sample size	♀/♂ (%)	Res p-rate (%)	QA score (/21)
Mabry, 2016[7]	Allam, 2012 [71]	Population-based survey (CS**)	Systematic randomization	Taibah University, Madinah **	2011**	Saudi** medical students	150 min of moderate-intensity PA/wk	IPAQ short version	Self-administrated **	Adults	35.50	Male and Female	194	♂<♀**	97**	20
Mabry, 2016[7]	Allam, 2012 [71]	Population-based survey (CS**)	Systematic randomization**	Taibah University, Madinah **	2011**	Saudi** medical students	150 min of moderate-intensity PA/wk	IPAQ short version	Self-administrated **	Adults	36.2	Male	94**	48.5**	96**	18
Mabry, 2016[7]	Allam, 2012 [71]	Population-based survey (CS**)	Systematic randomization**	Taibah University, Madinah **	2011**	Saudi** medical students	150 min of moderate-intensity PA/wk	IPAQ short version	Self-administrated **	Adults	35.0	Female	100**	51.5**	100 **	18
Mabry, 2016[7]	Al-Nozha, 2007 [12]	Population-based survey (CS**)	MCRS**	National-level**	1995-00**	Saudi **adult general population	150 min of moderate-intensity PA/wk	Validated questionnaire on leisure time PA and walking †	Face-to-face interviews**	30-70	3.9**	Male and Female	17395	Equal **	N/S	16
Mabry, 2016[7]	Al-Nozha, 2007 [12]	Population-based survey (CS**)	MCRS**	National-level**	1995-00**	Saudi **adult general population	150 min of moderate-intensity PA/wk	Validated questionnaire on leisure time PA and walking †	Face-to-face interviews**	30-70	6.1	Male	8297**	48**	N/S	16
Mabry, 2016[7]	Al-Nozha, 2007 [12]	Population-based survey (CS**)	MCRS**	National-level**	1995-00**	Saudi **adult general population	150 min of moderate-intensity PA/wk	Validated questionnaire on leisure time PA and walking †	Face-to-face interviews**	30-70	1.9	Female	9098**	52**	N/S	16
Mabry, 2016[7]	Awadalla, 2014 [68]	Population-based survey (CS**)	RCS**	King Khalid University**	2012-13**	Health professional college students	150 min of moderate-intensity PA/wk	IPAQ short version	Self-administrated **	Adults (17-25**)	42.0	Male and Female	1257	♂<♀**	85.6 **	19
Mabry, 2016[7]	Awadalla, 2014 [68]	Population-based survey (CS**)	RCS**	King Khalid University**	2012-13**	Health professional college students	150 min of moderate-intensity PA/wk	IPAQ short version	Self-administrated **	Adults (17-25 **)	43.7	Male	426**	33.9**	N/A	16
Mabry, 2016[7]	Awadalla, 2014 [68]	Population-based survey (CS**)	RCS**	King Khalid University**	2012-13**	Health professional college students	150 min of moderate-intensity PA/wk	IPAQ short version	Self-administrated **	Adults (17-25 **)	41.2	Female	831**	66.1**	N/A	16

Systematic review source	Study source	Study design	Sampling method	Setting	Years of data collection	Population	Physical activity definition/study	Physical activity instrument or used items	Instrument administration	Age group (years)	Prevalence of physical activity (%)	Gender	Sample size	♀/♂ (%)	Resp-rate (%)	QA score (/21)
Mabry, 2016[7]	Banday, 2015 [166]	Population-based survey (CS**)	Voluntary **	Primary health care centers and general hospitals of Sakaka and Dumat Al-Jandal areas of Aljouf region**	N/S	Primary Health care Physicians	150 min of moderate-intensity PA/wk	GPAQ	Face-to-face interviews**	27-63	65.20	Male and Female	106	♂>♀**	64**	13
Mabry, 2016[7]	Banday, 2015 [166]	Population-based survey (CS**)	Voluntary **	Primary health care centers and general hospitals of Sakaka and Dumat Al-Jandal areas of Aljouf region**	N/S	Primary Health care Physicians	150 min of moderate-intensity PA/wk	GPAQ	Face-to-face interviews**	27-63	66.3**	Male	92**	86**	N/A	11
Mabry, 2016[7]	Banday, 2015 [166]	Population-based survey (CS**)	Voluntary **	Primary health care centers and general hospitals of Sakaka and Dumat Al-Jandal areas of Aljouf region**	N/S	Primary Health care Physicians	150 min of moderate-intensity PA/wk	GPAQ	Face-to-face interviews**	27-63	57.1**	Female	14**	13**	N/A	11

Systematic review source	Study source	Study design	Sampling method	Setting	Years of data collection	Population	Physical activity definition/study	Physical activity instrument or used items	Instrument administration	Age group (years)	Prevalence of physical activity (%)	Gender	Sample size	♀/♂ (%)	Resp-rate (%)	QA score (/21)
Mabry, 2016[7]	Khalaf, 2013 [67]	Population-based survey (CS**)	MCRS**	University centre for women's studies in southwestern**	2010**	Female university students	150 min of moderate-intensity PA/wk	ATLS	Self-administrated **	Adults	62.4	Female	663	N/A	94.7**	19
Mabry, 2016[7]	Koura, 2012 [167]	Population-based survey (CS**)	MCRS**	University of Dammam**	2005-06**	Women college students	150 min of moderate-intensity PA/wk	GPAQ	Face-to-face interviews**	Adults	46.8	Female	370	N/A	28**	17
UAE (number of studies = 3)																
Mabry, 2010[4]	World Health Organization, 2003 [168]	Survey**	RS from national population register	National-level	2003**	Emirati general population	At least 600 MET-min/wk of vigorous or moderate activity	GPAQ	Face-to-face interviews	≥18	61.7	Male and Female	1180	♂>♀	72.0	19
Mabry, 2010[4]	World Health Organization, 2003 [168]	Survey**	RS from national population register	National-level	2003**	Emirati general population	At least 600 MET-min/wk of vigorous or moderate activity	GPAQ	Face-to-face interviews	≥18	66.5	Male	850*	72	N/A	17
Mabry, 2010[4]	World Health Organization, 2003 [168]	Survey**	RS from national population register	National-level	2003**	Emirati general population	At least 600 MET-min/wk of vigorous or moderate activity	GPAQ	Face-to-face interviews	≥18	49.3	Female	330*	28	N/A	17
Yammine, 2016[6]	Amine, 1996 [13]	N/S	RS	UAE University**	N/S	University students	Vigorous level of PA-(Severe participation in physical exercises**)	Personal PA questionnaire	Face-to-face interviews with students or their parents**	17-24	4.40	Female	206	N/A	N/S	11
Mabry, 2016[7]	Carter, 2003 [169]	Population-based survey (CS**)	N/S	Faculty of Medicine and Health Sciences, UAE	2000**	Medical students	150 min of moderate-intensity PA/wk	Nurses' Health Study II ‡	Self-administrated **	19-27	67.0	Male and Female	175	♂<♀**	84 for those present and	15

Systematic review source	Study source	Study design	Sampling method	Setting	Years of data collection	Population	Physical activity definition/study	Physical activity instrument or used items	Instrument administration	Age group (years)	Prevalence of physical activity (%)	Gender	Sample size	♀/♂ (%)	Resp-rate (%)	QA score (/21)
				University**											75 for all registered students**	
Oman (number of studies = 1)																
Mabry, 2016[7]	El-Aty, 2014 [170]	Oman World Health Survey**	MCRS**	National level**	2008**	Adults (Omani household**)	150 min of moderate-intensity PA/wk	GPAQ	N/S	18+	63.4	Male and Female	3137	♂<♀**	86.3**	20
Mabry, 2016[7]	El-Aty, 2014 [170]	Oman World Health Survey**	MCRS**	National level**	2008**	Adults (Omani household**)	150 min of moderate-intensity PA/wk	GPAQ	N/S	18+	68.0	Male	1459**	46.5**	N/S	17
Mabry, 2016[7]	El-Aty, 2014 [170]	Oman World Health Survey**	MCRS**	National level**	2008**	Adults (Omani household**)	150 min of moderate-intensity PA/wk	GPAQ	N/S	18+	59.5	Female	1678**	53.5**	N/S	17
Bahrain (number of studies = 2)																
Mabry, 2010[4]	Al-Mahroos, 1998 [139]	N/S	Systematic RS	National-level	1995-96**	Bahraini general population	Active: energy expenditure >0.3 MJ d-1 (Equivalent to walking daily for 30 min at 5km/h**)	Instrument contained questions on walking, cycling and recreational activities based on which energy expenditure was calculated	Face-to-face interviews	40-69	13.8	Male and Female	2128	♂>♀	59.0	17
Mabry, 2010[4]	Al-Mahroos, 1998 [139]	N/S	Systematic RS	National-level	1995-96**	Bahraini general population	Active: energy expenditure >0.3 MJ d-1 (Equivalent to walking daily for 30 min at 5km/h**)	Instrument contained questions on walking, cycling and recreational activities based on which energy	Face-to-face interviews	40-69	23.1	Male	1297*	59	N/S	15

Systematic review source	Study source	Study design	Sampling method	Setting	Years of data collection	Population	Physical activity definition/study	Physical activity instrument or used items	Instrument administration	Age group (years)	Prevalence of physical activity (%)	Gender	Sample size	♀/♂ (%)	Resp-rate (%)	QA score (/21)
								expenditure was calculated								
Mabry, 2010[4]	Al-Mahroos, 1998 [139]	N/S	Systematic RS	National-level	1995-96**	Bahraini general population	Active: energy expenditure >0.3 MJ d-1 (Equivalent to walking daily for 30 min at 5km/h**)	Instrument contained questions on walking, cycling and recreational activities based on which energy expenditure was calculated	Face-to-face interviews	40-69	1.3	Female	901*	41	N/S	15
Mabry, 2010[4]	Al-Mahroos, 2001 [138]	CS**	Systematic RS	National-level	N/S	Bahraini general population	Active: walk ≥1 km d-1	Instrument contained WHO Heart and Health Questionnaire (validated in Arabic**)	Face-to-face interviews	40-59	35**	Male	1168**	58**	N/S	12
Mabry, 2010[4]	Al-Mahroos, 2001 [138]	CS**	Systematic RS	National-level	N/S	Bahraini general population	Active: walk ≥1 km d-1	Instrument contained WHO Heart and Health Questionnaire (validated in Arabic**)	Face-to-face interviews	40-49	36	Male	668**	N/A	N/S	12
Mabry, 2010[4]	Al-Mahroos, 2001 [138]	CS**	Systematic RS	National-level	N/S	Bahraini general population	Active: walk ≥1 km d-1	Instrument contained WHO Heart and Health Questionnaire (validated in Arabic**)	Face-to-face interviews	50-59	32	Male	500**	N/A	N/S	12
Mabry, 2010[4]	Al-Mahroos, 2001 [138]	CS**	Systematic RS	National-level	N/S	Bahraini general population	Active: walk ≥1 km d-1	Instrument contained WHO Heart and Health Questionnaire (validated in Arabic**)	Face-to-face interviews	50-69	6.1**	Female	845**	42**	N/S	12
Mabry, 2010[4]	Al-Mahroos, 2001 [138]	CS**	Systematic RS	National-level	N/S	Bahraini general population	Active: walk ≥1 km d-1	Instrument contained WHO Heart and Health Questionnaire	Face-to-face interviews	60-69	5	Female	377**	N/A	N/S	12

Systematic review source	Study source	Study design	Sampling method	Setting	Years of data collection	Population	Physical activity definition/study	Physical activity instrument or used items	Instrument administration	Age group (years)	Prevalence of physical activity (%)	Gender	Sample size	♀/♂ (%)	Resp-rate (%)	QA score (/21)
								(validated in Arabic**)								
Mabry, 2010[4]	Al-Mahroos, 2001 [138]	CS**	Systematic RS	National-level	N/S	Bahraini general population	Active: walk ≥1 km d-1	Instrument contained WHO Heart and Health Questionnaire (validated in Arabic**)	Face-to-face interviews	50-59	7	Female	468**	N/A	N/S	12
Mabry, 2010[4]/ Sharara, 2018[9]	Al-Mahroos, 2001 [138]	CS**	Systematic RS	National-level	N/S	Bahraini general population	Active: walk ≥1 km d-1	Instrument contained WHO Heart and Health Questionnaire (validated in Arabic**)	Face-to-face interviews	40-69	22.9%**	Male and Female	2013**	70	♂> ♀	14
Kuwait (number of studies = 1)																
Mabry, 2010[4]	Ministry of Health, Kuwait, 2006 [171]	World Health Survey (CS**)	RSS	National-level	2006**	Kuwaiti general population	At least 600 MET-min/wk of vigorous or moderate activity	IPAQ	Face-to-face interviews	20-65	35.3	Male and Female	2280	♂<♀	77.6	19
Mabry, 2010[4]	Ministry of Health, Kuwait, 2006 [171]	World Health Survey (CS**)	RSS	National-level	2006**	Kuwaiti general population	At least 600 MET-min/wk of vigorous or moderate activity	IPAQ	Face-to-face interviews	20-65	42.1	Male	912*	40	72.2	19
Mabry, 2010[4]	Ministry of Health, Kuwait, 2006 [171]	World Health Survey (CS**)	RSS	National-level	2006**	Kuwaiti general population	At least 600 MET-min/wk of vigorous or moderate activity	IPAQ	Face-to-face interviews	20-65	28.4	Female	1368*	60	81.6	20
Qatar (number of studies = 2)																
Mabry, 2010[4]; Sharara, 2018[9]	Bener, 2004 [137]	CS**	MCRS	National-level	2003**	Qatari general population	Active: walked or cycled for at least 30 min d-1	Instrument contained a question if person walked or cycled at least 30 min d-1	Face-to-face interviews	25-65	43.5*	Male and Female	1208	♂<♀	80.5	18

Systematic review source	Study source	Study design	Sampling method	Setting	Years of data collection	Population	Physical activity definition/study	Physical activity instrument or used items	Instrument administration	Age group (years)	Prevalence of physical activity (%)	Gender	Sample size	♀/♂ (%)	Resp-rate (%)	QA score (/21)
Mabry, 2010[4]; Sharara, 2018[9]	Bener, 2004 [137]	CS**	MCRS	National-level	2003**	Qatari general population	Active: walked or cycled for at least 30 min d-1	Instrument contained a question if person walked or cycled at least 30 min d-1	Face-to-face interviews	25-65	49.2	Male	507*	42	N/A	15
Mabry, 2010[4]; Sharara, 2018[9]	Bener, 2004 [137]	CS**	MCRS	National-level	2003**	Qatari general population	Active: walked or cycled for at least 30 min d-1	Instrument contained a question if person walked or cycled at least 30 min d-1	Face-to-face interviews	25-65	39.5	Female	701*	58	N/A	15
Mabry, 2016[7]	Al Thani, 2015 [140]	Qatar STEPwise survey**	MCRS	National level **	2012**	Qatari** women	150 min of moderate-intensity PA/wk	GPAQ	Face-to-face interviews**	18-64	44.2	Female	747	N/A	88**	20

Notes: All non-reported data was searched and extracted from the original study. Any additional information found relevant in the original study was added to the reported data for the purpose of completeness.

If not reported, the prevalence of the outcome among the total study population (males and/or females) was calculated using row and/or calculated data available in the original study. The calculated prevalence measure was reported and marked using two stars (**).

If not reported, the total sample size for each gender strata was calculated based on the percentage of males or females in the sample.

If not reported, the number for cases in each gender strata was calculated based on the reported prevalence and the total sample size in the strata.

If not reported, the total number for cases in the entire sample was calculated by the addition of the number of cases in each reported stratum.

If any discordance between the reported data in the SR and data available in the original study, this later was retained.

Abbreviations: SR: Systematic review; PA: Physical activity; VPA: Vigorous physical activity: MPA: Moderate physical activity; KSA: Kingdom of Saudi Arabia; UAE: United Arab Emirates; USA: United States of America; GCC: Gulf cooperation council; MENA: Middle-East and North Africa; QA: Quality assessment; ATLS: Arab teens lifestyle student questionnaire; GPAQ: Global Physical Activity Questionnaire; IPAQ: International Physical Activity Questionnaire: CS: Cross sectional: MCRS: Multistage stratified/cluster random sampling: RS: Random sampling: RCS: Random cluster sampling; RSS: Random stratified sampling; MET: Metabolic Equivalent of Task: VG: Video games; Resp-rate: response rate; N/S: Not stated; N/A: Not applicable;

Symbols:

† The use of a validated questionnaire was not reported in the original study.

‡ The use of Nurses' Health Study II questionnaire was not reported in the original study.

* Calculated from reported data in the SR

** Calculated or reported from data in the original study

*** Calculated from the prevalence measure reported in the original study (different from the prevalence measure reported in the SR)

♂: Males; ♀: Females; km: Kilometer; min: Minutes; wk: Week; d: Day: d-1: Per day; hrs: hours; h: hour

MJ: The megajoule is equal to one million (106) joules; Energy expenditure of >0.3 MJ/day is equivalent to walking daily for half an hour at 5 km/h.

Table S9: The prevalence of physical activity among the youth general population of the included MENA countries.

Systematic review source	Study source	Study design	Sampling method	Setting	Years of data collection	Population	Physical activity definition/study	Physical activity instrument or used items	Instrument administration	Age group (years)	Prevalence of physical activity (%)	Gender	Sample size	♀/♂ (%)	Resp-rate (%)	QA score (/21)
Saudi-Arabia (number of studies = 6)																
Sisson, 2008[3]	Al-Hazzaa, 2007[128]	N/S	RS**	Riyadh	2005**	Saudi public-school children	Active ≥13,000 steps/d	Pedometers	Objectively measured	8-12	53	Male	296	N/A	N/S	18
Mabry, 2016[7]	Al-Hazzaa, 2014[143]	Population-based survey (CS**)	MCRS**	Al-Khobar, Jeddah and Riyadh **	2009**	Secondary** school students	60 min of moderate-intensity 7 d/wk	ATLS	N/S	15-19	31.50	Male and Female	2866	Equal**	N/S	16
Mabry, 2016[7]	Al-Hazzaa, 2014[143]	Population-based survey (CS**)	MCRS**	Al-Khobar, Jeddah and Riyadh **	2009**	Secondary** school students	60 min of moderate-intensity 7 d/wk	ATLS	N/S	15-19	43.8	Male	1384**	48.3**	N/S	16
Mabry, 2016[7]	Al-Hazzaa, 2014[143]	Population-based survey (CS**)	MCRS**	Al-Khobar, Jeddah and Riyadh **	2009**	Secondary** school students	60 min of moderate-intensity 7 d/wk	ATLS	N/S	15-19	20.2	Female	1482**	51.7**	N/S	16
Mabry, 2016[7]	Al-Hazzaa, 2011[124]	Population-based survey (CS**)	MCRS**	Al-Khobar, Jeddah and Riyadh **	2009-10**	Saudi** secondary-school students	60 min of moderate-intensity 7 d/wk	ATLS	Self-administrated **	14-19	38.1**	Male and Female*	2908	Equal**	N/S	17
Mabry, 2016[7]	Al-Hazzaa, 2011[172]	Population-based survey (CS**)	MCRS**	Al-Khobar, Jeddah and Riyadh **	2009-10**	Saudi** secondary-school students	60 min of moderate-intensity 7 d/wk	ATLS	Self-administrated **	14-19	55.5	Male	1401**	48.2**	N/S	17
Mabry, 2016[7]	Al-Hazzaa, 2011[124]	Population-based survey (CS**)	MCRS**	Al-Khobar, Jeddah and Riyadh **	2009-10**	Saudi** secondary-school students	60 min of moderate-intensity 7 d/wk	ATLS	Self-administrated **	14-19	21.9	Female	1507**	51.8**	N/S	17
Mabry, 2016[7]	Al-Hazzaa, 2013[144]	Population-based survey (CS**)	MCRS**	Al-Khobar, Jeddah and Riyadh **	2009-10**	Saudi** school students	60 min of moderate-intensity 7 d/wk	ATLS	Self-administrated **	15-19	37.8**	Male and Female*	2886 (2888**)	Equal**	99.2**	20
Mabry, 2016[7]	Al-Hazzaa, 2013[144]	Population-based survey (CS**)	MCRS**	Al-Khobar, Jeddah	2009-10**	Saudi** school students	60 min of moderate-intensity 7 d/wk	ATLS	Self-administrated **	15-19	55.0	Male	1388**	48.1** (based on	N/S	17

Systematic review source	Study source	Study design	Sampling method	Setting	Years of data collection	Population	Physical activity definition/study	Physical activity instrument or used items	Instrument administration	Age group (years)	Prevalence of physical activity (%)	Gender	Sample size	♀/♂ (%)	Resp-rate (%)	QA score (/21)
				and Riyadh **										a total sample size of 2888)		
Mabry, 2016[7]	Al-Hazzaa, 2013 [144]	Population-based survey (CS**)	MCRS**	Al-Khobar, Jeddah and Riyadh **	2009-10**	Saudi** school students	60 min of moderate-intensity 7 d/wk	ATLS	Self-administrated **	15-19	21.7	Female	1500**	51.9** (based on a total sample size of 2888)	N/S	17
Mabry, 2016[7]	Al-Hazzaa, 2013 [144]	Population-based survey (CS**)	MCRS**	Al-Khobar, Jeddah and Riyadh **	2009-10**	Saudi** school students	60 min of moderate-intensity 7 d/wk	ATLS	Self-administrated **	15-19	76.0**	Male and Female*	2822**	Equal**	99.2**	20
Mabry, 2016[7]	Al-Hazzaa, 2013 [144]	Population-based survey (CS**)	MCRS**	Al-Khobar and Riyadh **	2009-10**	Saudi** school students	60 min of moderate-intensity 7 d/wk	ATLS	N/S	14-18	36.0 (35.7**)	Male and Female	1648	Equal**	N/S	14
Mabry, 2016[7]	Al-Hazzaa, 2013 [144]	Population-based survey (CS**)	MCRS**	Al-Khobar and Riyadh **	2009-10**	Saudi** school students	60 min of moderate-intensity 7 d/wk	ATLS	N/S	14-18	53.4	Male	797**	48.4**	N/S	14
Mabry, 2016[7]	Al-Hazzaa, 2013 [144]	Population-based survey (CS**)	MCRS**	Al-Khobar and Riyadh **	2009-10**	Saudi** school students	60 min of moderate-intensity 7 d/wk	ATLS	N/S	14-18	19.1	Female	851**	51.6**	N/S	14
Mabry, 2016[7]	Al-Nakeeb, 2012 [146]	Population-based survey (N/S) **	RSS**	Al-Ahsa**	N/S	School students	60 min of moderate-intensity 7 d/wk	ATLS	Self-administrated **	15-17	26.0	Male and Female	2290 (UK+KSA**) 1107 (from KSA	Equal**	N/S	13

Systematic review source	Study source	Study design	Sampling method	Setting	Years of data collection	Population	Physical activity definition/study	Physical activity instrument or used items	Instrument administration	Age group (years)	Prevalence of physical activity (%)	Gender	Sample size	♀/♂ (%)	Resp-rate (%)	QA score (/21)
													only **)			
Mabry, 2016[7]	Al-Nakeeb, 2012 [146]	Population-based survey (N/S**)	RSS**	Al-Ahsa**	N/S	School students	60 min of moderate-intensity 7 d/wk	ATLS	Self-administrated **	15-17	45.8	Male	576**	52.0**	N/S	13
Mabry, 2016[7]	Al-Nakeeb, 2012 [146]	Population-based survey (N/S**)	RSS**	Al-Ahsa**	N/S	School students	60 min of moderate-intensity 7 d/wk	ATLS	Self-administrated **	15-17	4.5	Female	531**	48.0**	N/S	13
Mabry, 2016[7]	Al-Nuaim, 2012 [147]	Population-based survey (CS**)	RS**	Al-Ahsa**	N/S	Secondary ** school students	60 min of moderate-intensity 7 d/wk	ATLS	Self-administrated **	15-19	25.1**	Male and Female*	1270	Equal**	N/S	13
Mabry, 2016[7]	Al-Nuaim, 2012 [147]	Population-based survey (CS**)	RS**	Al-Ahsa**	N/S	Secondary ** school students	60 min of moderate-intensity 7 d/wk	ATLS	Self-administrated **	15-19	44.5	Male	663**	52.2**	N/S	13
Mabry, 2016[7]	Al-Nuaim, 2012 [147]	Population-based survey (CS**)	RS**	Al-Ahsa**	N/S	Secondary ** school students	60 min of moderate-intensity 7 d/wk	ATLS	Self-administrated **	15-19	4.0	Female	607**	47.8**	N/S	13
UAE (number of studies = 4)																
Yammine, 2016[6]	Guthold, 2010 [10]	GSHS**	Two-stage** RS	National level**	2005**	Adolescents	Moderate level of physical activity – (At least 60 min of PA per day on at least 5 days per week**)	Adolescent PA measure questionnaire (PACE+**)	Self-administrated **	14 (mean)	19.7**	Male and Female*	9916**	Equal	88.3**	18
Yammine, 2016[6]	Guthold, 2010 [10]	GSHS**	Two-stage** RS	National level**	2005**	Adolescents	Moderate level of physical activity – (At least 60 min of PA per day on at least 5 days per week**)	Adolescent PA measure questionnaire (PACE+**)	Self-administrated **	14 (mean)	27	Male	4849	48.9**	N/S	15
Yammine, 2016[6]	Guthold, 2010 [10]	GSHS**	Two-stage** RS	National level**	2005**	Adolescents	Moderate level of physical activity – (At least 60 min of PA per day on at least 5 days per week**)	Adolescent PA measure questionnaire (PACE+**)	Self-administrated **	15 (mean)	18	Female	5067	51.1**	N/S	15

Systematic review source	Study source	Study design	Sampling method	Setting	Years of data collection	Population	Physical activity definition/study	Physical activity instrument or used items	Instrument administration	Age group (years)	Prevalence of physical activity (%)	Gender	Sample size	♀/♂ (%)	Resp-rate (%)	QA score (/21)
Yammine, 2016[6]	Mehairi, 2013 [14]	CS**	RS	Al Ain Abu Dhabi**	2010**	School students	Moderate level of PA – (Moderate score of PA**)	IPAQ short version	Self-administrated **	12-18	29.3**	Male and Female	1018**	Equal	68.3**	17
Yammine, 2016[6]	Mehairi, 2013 [14]	CS**	RS	Al Ain Abu Dhabi**	2010**	School students	Moderate level of PA – (Moderate score of PA**)	IPAQ short version	Self-administrated **	12-18	27.4	Male	522	51.6**	N/S	15
Yammine, 2016[6]	Mehairi, 2013 [14]	CS**	RS	Al Ain Abu Dhabi**	2010**	School students	Moderate level of PA – (Moderate score of PA**)	IPAQ short version	Self-administrated **	12-18	31.2	Female	496	48.4**	N/S	15
Yammine, 2016[6]	Muhairi, 2013 [15]	CS**	RS	Al Ain Abu Dhabi**	2010**	School students	Moderate level of PA – (Moderate score of PA**)	IPAQ short version	Self-administrated **	15-18	32.4	Male and Female	315	Equal	N/S	15
Yammine, 2016[6]	Wasfi, 2008 [16]	Survey**	RSS**	Bur Dubai and Deira**	2010**	Secondary school students	Moderate level of PA - (Vigorous exercise < 3 times/wk for about 60 min and > 30 min of moderate PA most days of the week**)	Personal PA questionnaires	Self-administrated **	14-18	8.9*	Male and Female*	1475*	♂>♀	N/S	15
Yammine, 2016[6]	Wasfi, 2008 [16]	Survey**	RSS**	Bur Dubai and Deira**	2004**	Secondary school students	Moderate level of PA - (Vigorous exercise < 3 times/wk for about 60 min and > 30 min of moderate PA most days of the week**)	Personal PA questionnaires	Self-administrated **	14-18	11.7	Male	835	56.6**	N/S	15
Yammine, 2016[6]	Wasfi, 2008 [16]	Survey**	RSS**	Bur Dubai and Deira**	2004**	Secondary school students	Moderate level of PA - (Vigorous exercise < 3 times/wk for about 60 min and > 30 min of moderate PA most days of the week**)	Personal PA questionnaires	Self-administrated **	14-18	5.2	Female	640	43.4**	N/S	15

Systematic review source	Study source	Study design	Sampling method	Setting	Years of data collection	Population	Physical activity definition/study	Physical activity instrument or used items	Instrument administration	Age group (years)	Prevalence of physical activity (%)	Gender	Sample size	♀/♂ (%)	Resp-rate (%)	QA score (/21)
Yammine, 2016[6]	Mehairi, 2013 [14]	CS**	RS	Al Ain Abu Dhabi**	2010**	School students	Vigorous level of PA-(High score of PA**)	IPAQ short version	Self-administrated **	12-18	34.4**	Male and Female	1018**	Equal	68.3**	17
Yammine, 2016[6]	Mehairi, 2013 [14]	CS**	RS	Al Ain Abu Dhabi**	2010**	School students	Vigorous level of PA –(High score of PA**)	IPAQ short version	Self-administrated **	12-18	47.1	Male	522	51.6**	N/S	15
Yammine, 2016[6]	Mehairi, 2013 [14]	CS**	RS	Al Ain Abu Dhabi**	2010**	School students	Vigorous level of PA –(High score of PA**)	IPAQ short version	Self-administrated **	12-18	21.0	Female	496	48.4**	N/S	15
Yammine, 2016[6]	Muhairi, 2013 [15]	CS**	RS	Al Ain Abu Dhabi**	2010**	School students	Vigorous level of PA –(High score of PA**)	IPAQ short version	Self-administrated **	15-18	30.1	Male and Female	315	Equal	N/S	15
Yammine, 2016[6]	Wasfi, 2008 [16]	Survey**	RSS**	Bur Dubai and Deira**	2004**	School students	Vigorous level of PA-(Vigorous exercise ≥ 3 times/wk for about 20 min/session and also > 30 min of moderate PA most days of the week **)	Personal PA questionnaires	Self-administrated **	14-18	21.1*	Male and Female	1475*	♂>♀	N/S	15
Yammine, 2016[6]	Wasfi, 2008 [16]	Survey**	RSS**	Bur Dubai and Deira**	2004**	Secondary school students	Vigorous level of PA-(Vigorous exercise ≥ 3 times/wk for about 20 min/session and also > 30 min of moderate PA most days of the week **)	Personal PA questionnaires	Self-administrated **	14-18	26.0	Male	835	56.6**	N/S	15
Yammine, 2016[6]	Wasfi, 2008 [16]	Survey**	RSS**	Bur Dubai and Deira**	2004**	Secondary school students	Vigorous level of PA-(Vigorous exercise ≥ 3 times/wk for about 20 min/session and also > 30 min of moderate PA	Personal PA questionnaires	Self-administrated **	14-18	14.7	Female	640	43.4**	N/S	15

Systematic review source	Study source	Study design	Sampling method	Setting	Years of data collection	Population	Physical activity definition/study	Physical activity instrument or used items	Instrument administration	Age group (years)	Prevalence of physical activity (%)	Gender	Sample size	♀/♂ (%)	Resp-rate (%)	QA score (/21)
							most days of the week **)									
Yammine, 2016[6]	Wasfi, 2008 [16]	Survey**	RSS**	Bur Dubai and Deira**	2004**	Emirati secondary school students	Moderate level of PA - (Vigorous exercise < 3 times/week for about 60 min and > 30 min of moderate physical activity most days of the week**)	Personal PA questionnaires	Self-administrated **	14-18	12.9	Male and Female	233	Unclear	N/S	16
Yammine, 2016[6]	Wasfi, 2008 [16]	Survey**	RSS**	Bur Dubai and Deira**	2004**	Emirati secondary school students	Vigorous level of PA- (Vigorous exercise ≥ 3 times/wk for about 20 min/session and also > 30 min of moderate PA most days of the week**)	Personal PA questionnaires	Self-administrated **	14-18	33.9	Male and Female	233	Unclear	N/S	16
Yammine, 2016[6]	Wasfi, 2008 [16]	Survey**	RSS**	Bur Dubai and Deira**	2004**	Non-Emirati secondary school students	Moderate level of PA - (Vigorous exercise < 3 times/week for about 60 min and > 30 min of moderate physical activity most days of the week**)	Personal PA questionnaires	Self-administrated **	14-18	8.1	Male and Female	1242	Unclear	N/S	16
Yammine, 2016[6]	Wasfi, 2008 [16]	Survey**	RSS**	Bur Dubai and Deira**	2004**	Non-Emirati secondary school students	Vigorous level of PA- (Vigorous exercise ≥ 3 times/wk for about 20 min/session and also > 30 min of moderate PA most days of the week**)	Personal PA questionnaires	Self-administrated **	14-18	18.7	Male and Female	1242	Unclear	N/S	16

Systematic review source	Study source	Study design	Sampling method	Setting	Years of data collection	Population	Physical activity definition/study	Physical activity instrument or used items	Instrument administration	Age group (years)	Prevalence of physical activity (%)	Gender	Sample size	♀/♂ (%)	Resp-rate (%)	QA score (/21)
Oman (number of studies = 2)																
Mabry, 2016[7]	Kilani, 2013 [173]	Population-based survey (CS**)	MCRS**	Muscat**	2010**	Adolescents (Secondary-school students**)	60 min of moderate-intensity 7 d/wk	ATLS	Face-to- face interviews**	15-18	42.6**	Male and Female*	802	♂<♀ **	N/S	16
Mabry, 2016[7]	Kilani, 2013 [173]	Population-based survey (CS**)	MCRS**	Muscat**	2010**	Adolescents (Secondary-school students**)	60 min of moderate-intensity 7 d/wk	ATLS	Face-to- face interviews**	15-18	66.7	Male	360**	44.9**	N/S	16
Mabry, 2016[7]	Kilani, 2013 [173]	Population-based survey (CS**)	MCRS**	Muscat**	2010**	Adolescents (Secondary-school students**)	60 min of moderate-intensity 7 d/wk	ATLS	Face-to- face interviews**	15-18	23.1	Female	442**	55.1**	N/S	16
Yammine, 2016[6]	Guthold, 2010 $ [10]	GSHS**	Two-stage RS	National level	2005	Schoolchildren	At least 60 min of PA per day on at least 5 days per week	Validated PACE+ and Adolescent physical activity measure questionnaire	Self-administrated	14 (mean)	25.4**	Male and Female**	2158**	Equal	97	18
Yammine, 2016[6]	Guthold, 2010 $ [10]	GSHS**	Two-stage RS	National level	2005	Schoolchildren	At least 60 min of PA per day on at least 5 days per week	Validated PACE+ and Adolescent physical activity measure questionnaire	Self-administrated	14 (mean)	34	Male	1133	52.5	N/S	15
Yammine, 2016[6]	Guthold, 2010 $ [10]	GSHS**	Two-stage RS	National level	2005	Schoolchildren	At least 60 min of PA per day on at least 5 days per week	Validated PACE+ and Adolescent physical activity measure questionnaire	Self-administrated	14 (mean)	16	Female	1025	47.5	N/S	15
Kuwait (number of studies = 1)																

Systematic review source	Study source	Study design	Sampling method	Setting	Years of data collection	Population	Physical activity definition/study	Physical activity instrument or used items	Instrument administration	Age group (years)	Prevalence of physical activity (%)	Gender	Sample size	♀/♂ (%)	Resp-rate (%)	QA score (/21)
Mabry, 2016[7]	Allafi, 2013 [174]	Population-based survey (CS**)	MCRS**	6 Kuwaiti Governorates**	2009**	Adolescents (Kuwaiti secondary school students**)	60 min of moderate-intensity 7 d/wk	ATLS	Self-administrated **	14-19	55.2**	Male and Female*	906	Equal**	N/S	14
Mabry, 2016[7]	Allafi, 2013 [174]	Population-based survey (CS**)	MCRS**	6 Kuwaiti Governorates**	2009**	Adolescents (Kuwaiti secondary school students**)	60 min of moderate-intensity 7 d/wk	ATLS	Self-administrated **	14-19	70.5	Male	463**	51.1**	N/S	14
Mabry, 2016[7]	Allafi, 2013 [174]	Population-based survey (CS**)	MCRS**	6 Kuwaiti Governorates**	2009**	Adolescents (Kuwaiti secondary school students**)	60 min of moderate-intensity 7 d/wk	ATLS	Self-administrated **	14-19	39.2	Female	443**	48.9**	N/S	14
Djibouti (number of studies = 1)																
Yammine, 2016[6]	Guthold, 2010 $ [10]	GSHS**	Two-stage RS	National level**	2005	Schoolchildren	At least 60 min of PA per day on at least 5 days per week	Validated PACE+ and Adolescent physical activity measure questionnaire	Self-administrated	14.4 (mean)	16.9**	Male and Female**	882**	♂>♀	83	18
Yammine, 2016[6]	Guthold, 2010 $ [10]	GSHS**	Two-stage RS	National level**	2005	Schoolchildren	At least 60 min of PA per day on at least 5 days per week	Validated PACE+ and Adolescent physical activity measure questionnaire	Self-administrated	14.4 (mean)	21	Male	526	59.6	N/S	15
Yammine, 2016[6]	Guthold, 2010 $ [10]	GSHS**	Two-stage RS	National level	2005	Schoolchildren	At least 60 min of PA per day on at least 5 days per week	Validated PACE+ and Adolescent physical activity measure questionnaire	Self-administrated	14.4 (mean)	11	Female	356	40.4	N/S	15

Systematic review source	Study source	Study design	Sampling method	Setting	Years of data collection	Population	Physical activity definition/study	Physical activity instrument or used items	Instrument administration	Age group (years)	Prevalence of physical activity (%)	Gender	Sample size	♀/♂ (%)	Resp-rate (%)	QA score (/21)
Egypt (number of studies = 1)																
Yammine, 2016[6]	Guthold, 2010 $ [10]	GSHS**	Two-stage RS	National level	2005	Schoolchildren	At least 60 min of PA per day on at least 5 days per week	Validated PACE+ and Adolescent physical activity measure questionnaire	Self-administrated	13.5 (mean)	9.4	Male and Female**	3664**	♀>♂	87	18
Yammine, 2016[6]	Guthold, 2010 $ [10]	GSHS**	Two-stage RS	National level	2005	Schoolchildren	At least 60 min of PA per day on at least 5 days per week	Validated PACE+ and Adolescent physical activity measure questionnaire	Self-administrated	13.5 (mean)	14	Male	1975	53.9	N/S	15
Yammine, 2016[6]	Guthold, 2010 $ [10]	GSHS**	Two-stage RS	National level	2005	Schoolchildren	At least 60 min of PA per day on at least 5 days per week	Validated PACE+ and Adolescent physical activity measure questionnaire	Self-administrated	13.5 (mean)	4	Female	1689	46.1	N/S	15
Jordan (number of studies = 1)																
Yammine, 2016[6]	Guthold, 2010 $ [10]	GSHS**	Two-stage RS	National level	2005	Schoolchildren	At least 60 min of PA per day on at least 5 days per week	Validated PACE+ and Adolescent physical activity measure questionnaire	Self-administrated	14.4 (mean)	17.4**	Male and Female**	1719**	Equal	95	18
Yammine, 2016[6]	Guthold, 2010 $ [10]	GSHS**	Two-stage RS	National level	2005	Schoolchildren	At least 60 min of PA per day on at least 5 days per week	Validated PACE+ and Adolescent physical activity measure questionnaire	Self-administrated	14.4 (mean)	20	Male	829	48.2	N/S	15
Yammine, 2016[6]	Guthold, 2010 $ [10]	GSHS**	Two-stage RS	National level	2005	Schoolchildren	At least 60 min of PA per day on at least 5 days per week	Validated PACE+ and Adolescent physical	Self-administrated	14.4 (mean)	15	Female	890	51.8	N/S	15

Systematic review source	Study source	Study design	Sampling method	Setting	Years of data collection	Population	Physical activity definition/study	Physical activity instrument or used items	Instrument administration	Age group (years)	Prevalence of physical activity (%)	Gender	Sample size	♀/♂ (%)	Resp-rate (%)	QA score (/21)
								activity measure questionnaire								
Libya (number of studies = 1)																
Yammine, 2016[6]	Guthold, 2010 $ [10]	GSHS**	Two-stage RS	National level	2005	Schoolchildren	At least 60 min of PA per day on at least 5 days per week	Validated PACE+ and Adolescent physical activity measure questionnaire	Self-administrated	13.9 (mean)	18**	Male and Female**	1354**	Equal	98**	18
Yammine, 2016[6]	Guthold, 2010 $ [10]	GSHS**	Two-stage RS	National level	2005	Schoolchildren	At least 60 min of PA per day on at least 5 days per week	Validated PACE+ and Adolescent physical activity measure questionnaire	Self-administrated	13.9 (mean)	24	Male	163	50.3	N/S	15
Yammine, 2016[6]	Guthold, 2010 $ [10]	GSHS**	Two-stage RS	National level	2005	Schoolchildren	At least 60 min of PA per day on at least 5 days per week	Validated PACE+ and Adolescent physical activity measure questionnaire	Self-administrated	13.9 (mean)	12	Female	81	49.7	N/S	15
Morocco (number of studies = 1)																
Yammine, 2016[6]	Guthold, 2010 $ [10]	GSHS**	Two-stage RS	National level	2005	Schoolchildren	At least 60 min of PA per day on at least 5 days per week	Validated PACE+ and Adolescent physical activity measure questionnaire	Self-administrated	14.1 (mean)	16.7**	Male and Female**	1735**	♀>♂	84	18
Yammine, 2016[6]	Guthold, 2010 $ [10]	GSHS**	Two-stage RS	National level	2005	Schoolchildren	At least 60 min of PA per day on at least 5 days per week	Validated PACE+ and Adolescent physical activity measure questionnaire	Self-administrated	14.1 (mean)	20	Male	926	53.4	N/S	15

Systematic review source	Study source	Study design	Sampling method	Setting	Years of data collection	Population	Physical activity definition/study	Physical activity instrument or used items	Instrument administration	Age group (years)	Prevalence of physical activity (%)	Gender	Sample size	♀/♂ (%)	Resp-rate (%)	QA score (/21)
Yammine, 2016[6]	Guthold, 2010 $ [10]	GSHS**	Two-stage RS	National level	2005	Schoolchildren	At least 60 min of PA per day on at least 5 days per week	Validated PACE+ and Adolescent physical activity measure questionnaire	Self-administrated	14.1 (mean)	13	Female	809	46.6	N/S	15

Notes: All non-reported data was searched and extracted from the original study. Any additional information found relevant in the original study was added to the reported data for the purpose of completeness.

If not reported, the prevalence of the outcome among the total study population (males and/or females) was calculated using row and/or calculated data available in the original study. The calculated prevalence measure was reported and marked using two stars (**).

If not reported, the total sample size for each gender strata was calculated based on the percentage of males or females in the sample.

If not reported, the number for cases in each gender strata was calculated based on the reported prevalence and the total sample size in the strata.

If not reported, the total number for cases in the entire sample was calculated by the addition of the number of cases in each reported stratum.

If any discordance between the reported data in the SR and data available in the original study, this later was retained.

Abbreviations: SR: Systematic review; PA: Physical activity; VPA: Vigorous physical activity: MPA: Moderate physical activity; KSA: Kingdom of Saudi Arabia; UAE: United Arab Emirates; USA: United States of America; GCC: Gulf cooperation council; MENA: Middle-East and North Africa; QA: Quality assessment; ATLS: Arab teens lifestyle student questionnaire; GPAQ: Global Physical Activity Questionnaire; IPAQ: International Physical Activity Questionnaire: CS: Cross sectional: MCRS: Multistage stratified/cluster random sampling: RS: Random sampling: RCS: Random cluster sampling; RSS: Random stratified sampling; MET: Metabolic Equivalent of Task: VG: Video games; GSHS: Global School-based Student Health Survey; Resp-rate: response rate; N/S: Not stated; N/A: Not applicable;

Symbols:

† The use of a validated questionnaire was not reported in the original study.

‡ The use of Nurses' Health Study II questionnaire was not reported in the original study.

* Calculated from reported data in the SR

** Calculated or reported from data in the original study

*** Calculated from the prevalence measure reported in the original study (different from the prevalence measure reported in the SR)

$ This study was included in the SR of Yammine, 2016 [6]. PA data extracted from this study of Guthold, 2010 [10] was not reported by the SR of Yammine, 2016 [6].

♂: Males; ♀: Females; km: Kilometer; min: Minutes; wk: Week; d: Day: d-1: Per day; hrs: hours; h: hour

MJ: The megajoule is equal to one million (106) joules

Energy expenditure of >0.3 MJ/day is equivalent to walking daily for half an hour at 5 km/h.

Table S10: Physical inactivity and sedentary behavior among the adult general population of the included MENA countries.

Systematic review source	Study source	Study design	Sampling method	Setting	Years of data collection	Population	Physical inactivity or sedentary behavior definition /study	Physical inactivity or sedentary behavior instrument or used items	Instrument administration	Age group (years)	Physical inactivity or sedentary behavior measurement	Gender	Sample size	♀/♂ (%)	Resp-rate (%)	QA score (/21)
Saudi-Arabia (number of studies = 34)																
Sisson, 2008[3]; Sharara, 2018[9]; Al-Hazzaa, 2018[8]	Al-Nozha, 2007 [12]	CS**	MCRS**	National-level	1995-00	Saudi participants in the Public-School Coronary Artery Disease Study	Inactive: <600 METmin/wk	PA questionnaire**	Self-reported (Face-to- face interviews**)	30-70	96.1%**	Male and Female	17223 **	N/S	N/S	16
Sisson, 2008[3]; Sharara, 2018[9]; Al-Hazzaa, 2018[8]	Al-Nozha, 2007 [12]	CS**	MCRS**	National-level	1995-00	Saudi participants in the Public-School Coronary Artery Disease Study	Inactive: <600 METmin/wk	PA questionnaire**	Self-reported (Face-to- face interviews**)	30-70	94%	Male	8215**	N/S	N/S	16
Sisson, 2008[3]; Al-Hazzaa, 2018[8]	Al-Nozha, 2007 [12]	CS**	MCRS**	National-level	1995-00	Saudi participants in the Public-School Coronary Artery Disease Study	Inactive: <600 MET min/wk	PA questionnaire**	Self-reported (Face-to- face interviews**)	30-70	98%	Female	9008**	N/S	N/S	16
Mabry, 2010[4]	Ministry of Health, Saudi Arabia, 2005 [25]	World Health Survey (CS**)	MCRS	National-level*	2004**	Saudi general population	Mean amount of time spent sitting (Mean total time spent in sedentary activities (min/d) **)	GPAQ	Face-to- face interviews	25-64	279. 4 min d-1**	Male and Female	4758	Equal	97.7	20
Mabry, 2010[4]	Ministry of Health, Saudi Arabia, 2005 [25]	World Health Survey (CS**)	MCRS	National-level*	2004**	Saudi general population	Mean amount of time spent sitting (Mean total time spent in sedentary activities (min/d) **)	GPAQ	Face-to- face interviews	25-64	283 min d-1	Male	2284*	48	N/S	17

Systematic review source	Study source	Study design	Sampling method	Setting	Years of data collection	Population	Physical inactivity or sedentary behavior definition /study	Physical inactivity or sedentary behavior instrument or used items	Instrument administration	Age group (years)	Physical inactivity or sedentary behavior measurement	Gender	Sample size	♀/♂ (%)	Resp-rate (%)	QA score (/21)
Mabry, 2010[4]	Ministry of Health, Saudi Arabia, 2005 [25]	World Health Survey (CS**)	MCRS	National level*	2004**	Saudi general population	Mean amount of time spent sitting (Mean total time spent in sedentary activities (min/d) **)	GPAQ	Face-to- face interviews	25-64	276 min d-1	Female	2474*	52	N/S	17
Sharara, 2018[9]	Al-Baghli, 2008[18]	A community-based screening campaign **	All targeted population invited**	National level (Eastern province of Saudi Arabia) **	2004-2005	General adult population	No physical activity or mild physical activity (ordinary housework, walking)	Structured questionnaire	Face-to- face interviews**	30+	79.2%	Male and Female **	197681	N/S	33	14
Sharara, 2018[9]	Memish, 2014[19]	Saudi Health Information Survey	MCRS**	National level (Household) **	2013	Saudi nationals	Neither moderate nor vigorous PA	IPAQ	personal interviews**	15+	69.1%	Male and Female **	10735	♂<♀**	89.4 **	18
Sharara, 2018[9]	Almurshed, 2009[20]	case–control study **	Selected consecutively over 1year**	Subnational level	2003-2004	Newly diagnosed colon cancer patients	No exercise	Questionnaire	N/S	30+	52%	Male and Female **	50	Equal	N/R	12
Sharara, 2018[9]	Al-Senany, 2015[22]	CS**	NS	Sub national level (A major hospital in Jeddah) **	N/S	Elderly individuals	Less than one-hour weekly activity	Interview questionnaire**	Interviews**	60-90	69%	Male and Female **	55	N/S	N/S	6
Sharara, 2018[9]	Amin, 2014[23]	CS**	Multistage proportionate sampling method	Sub national level (PHCC) **	N/S	Adult Saudi nationals	<30 min /≥ 5 days/week	GPAQ	Interviews**	18-78	80%	Male and Female **	2127	N/S	N/S	14

Systematic review source	Study source	Study design	Sampling method	Setting	Years of data collection	Population	Physical inactivity or sedentary behavior definition /study	Physical inactivity or sedentary behavior instrument or used items	Instrument administration	Age group (years)	Physical inactivity or sedentary behavior measurement	Gender	Sample size	♀/♂ (%)	Resp-rate (%)	QA score (/21)
Sharara, 2018[9]	Garawi, 2015[24]	CS**	MSRS **	Sub national level (Household)	2004-2005	Saudi nationals	<600 MET-min/week	GPAQ	Interviews**	15-64	67%	Male and Female **	4758	Equal**	98**	20
Sharara, 2018[9]	WHO-STEPS surveys, 2005[25]	CS**	MCRS**	Nation-al level	2004	Adults	<600 MET-min/week	STEPS Instrument for NCD Risk Factors ** (GPAQ)	Face-to- face interviews**	25-64	67.6%	Male and Female **	3547	Equal	97.7 **	19
Sharara, 2018[9]	WHO-STEPS surveys, 2005[25]	CS**	MCRS**	Nation-al level	2004	Adults	<600 MET-min/week	STEPS Instrument for NCD Risk Factors ** (GPAQ)	Face-to- face interviews**	25-64	61.0%**	Male	1713**	48.3 **	N/S	16
Sharara, 2018[9]	WHO-STEPS surveys, 2005[25]	CS**	MCRS**	Nation-al level	2004	Adults	<600 MET-min/week	STEPS Instrument for NCD Risk Factors ** (GPAQ)	Face-to- face interviews**	25-64	73.7%**	Female	1835**	51.7 **	N/S	16
Al-Hazzaa, 2018[8]	Al-Hazzaa, 1990[64]	N/S	RS	Riyadh	1990	College students	No regular activity	Questionnaire	Self-reported	21.9 (+-2.1)	78.2%	Male	362	N/A	N/S	14
Al-Hazzaa, 2018[8]	Al-Gelban, 2008[65]	CS**	Systematic RS	Abha, Aseer region of Saudi Arabia	2005/2006	Teacher's Training College students	<3 times/week	Questionnaire	Self-administered	17+	83.3%	Male	456	N/A	N/S	15
Al-Hazzaa, 2018[8]	Gawwad, 2008[66]	CS**	RS	Riyadh	March-May 2007	College students	<3 days/week	Questionnaire	Self-administered	21.4+-1.8	75.4%	Male and Female	302	Equal	59.1 %	17
Al-Hazzaa, 2018[8]	Gawwad, 2008[66]	CS**	RS	Riyadh	March-May 2007	College students	<3 days/week	Questionnaire	Self-administered	21.4+-1.8	72%	Male	150	49.6	N/S	15

Systematic review source	Study source	Study design	Sampling method	Setting	Years of data collection	Population	Physical inactivity or sedentary behavior definition /study	Physical inactivity or sedentary behavior instrument or used items	Instrument administration	Age group (years)	Physical inactivity or sedentary behavior measurement	Gender	Sample size	♀/♂ (%)	Res p-rate (%)	QA score (/21)
Al-Hazzaa, 2018[8]	Gawwad, 2008[66]	CS**	RS	Riyadh	March-May 2007	College students	<3 days/week	Questionnaire	Self-administered	21.4+-1.8	78.9%	Female	152	50.3	N/S	15
Al-Hazzaa, 2018[8]	Khalaf A, 2013[67]	CS**	RS	Abha	Spring 2010	College students	<150 min/week	Validated questionnaire: ATLS	Self-reported	20.4+-1.5	37.6%	Female	663	N/A	N/A	17
Al-Hazzaa, 2018[8] / Sharara, 2018[9]	Awadalla, 2014[68]	CS**	Stratified cluster sampling	Abha	NS	College students	<600 METs-min per week	IPAQ	Self-reported	17-25	58%	Male and Female	1257	♂< ♀	N/S	11
Al-Hazzaa, 2018[8]	Awadalla, 2014[68]	CS**	N/S	Abha	NS	College students	<600 METs-min per week	IPAQ	Self-reported	17-25	56.1%	Male	426	33.8	N/A	11
Al-Hazzaa, 2018[8]	Awadalla, 2014[68]	CS**	N/A	Abha	NS	College students	<600 METs-min per week	IPAQ	Self-reported	17-25	58.8%	Female	831	66.1	N/A	11
Al-Hazzaa, 2018[8]	Majeed, 2015[69]	CS**	Stratified RS	Dammam	September to November 2013	College students	<1 time per week	Questionnaire	Self-reported	19.3+-0.95	72.5%	Female	215	N/A	N/A	15
Al-Hazzaa, 2018[8]	Samara, 2015[70]	CS**	N/S	Riyadh	2014	College students	< 600 METs-min/ week	ATLS	Self-administered	18-22	44%	Female	94	N/A	N/A	14
Al-Hazzaa, 2018[8]	Allam, 2012[71]	CS**	N/S	Madinah	January to May 2011	College students	<150 mins/ week moderate activity or <60 min/ week vigorous activity	IPAQ	N/S	21.1+-1.9	64.4%	Male and Female	196**	Equal	97%	17
Al-Hazzaa, 2018[8]	Allam, 2012[71]	CS**	N/S	Madinah	January to May 2011	College students	<150 mins/ week moderate activity or <60 min/ week vigorous activity	IPAQ	N/S	21.1+-1.9	65%	Female	100	51	97%	17
Al-Hazzaa, 2018[8]	Allam, 2012[71]	CS**	N/S	Madinah	January to May 2011	College students	<150 mins/ week moderate activity or <60 min/ week vigorous activity	IPAQ	N/S	21.1+-1.9	63.8%	Male	96	49	97%	17
Al-Hazzaa, 2018[8]	Al-Shahri, 1998[72]	N/S	RS	Riyadh	N/S	Primary care Physicians	No regular activity	Questionnaire	Self-administered	42+-6.5	76.5% **	Male	89**	N/A	99%	14

Systematic review source	Study source	Study design	Sampling method	Setting	Years of data collection	Population	Physical inactivity or sedentary behavior definition /study	Physical inactivity or sedentary behavior instrument or used items	Instrument administration	Age group (years)	Physical inactivity or sedentary behavior measurement	Gender	Sample size	♀/♂ (%)	Res p-rate (%)	QA score (/21)
Al-Hazzaa, 2018[8]	Al Alwan, 2013[73]	CS**	RS	Riyadh	July-Aug 2009	Physicians and non-physicians	No PA	Questionnaire	Self-reported	20+	43%	Male and Female	200	Equal	N/S	14
Al-Hazzaa, 2018[8]	Al Alwan, 2013[73]	CS**	RS	Riyadh	July-Aug 2009	Physicians and non-physicians	No PA	Questionnaire	Self-reported	20+	41%	Male	98	49	N/S	14
Al-Hazzaa, 2018[8]	Al Alwan, 2013[73]	CS**	RS	Riyadh	July-Aug 2009	Physicians and non-physicians	No PA	Questionnaire	Self-reported	20+	45%	Female	102	51	N/S	14
Al-Hazzaa, 2018[8]	Al Ateeq, 2014[74]	CS**	Stratified RS	Riyadh	Dec 2012-Feb 2013	Primary care professionals	Active category: 30 min or more a day of moderate PA, 5 or more days a week. OR 20 min or more a day of vigorous PA, 3 or more days a week**	"How physically active are you" questionnaire	Trained nurse collected data	39.2+-8.9	78.85%	Male and Female	322	♂< ♀	N/S	17
Al-Hazzaa, 2018[8]	Al Ateeq, 2014[74]	CS**	Stratified RS	Riyadh	Dec 2012-Feb 2013	Primary care professionals	Active category: 30 min or more a day of moderate PA, 5 or more days a week. OR 20 min or more a day of vigorous PA, 3 or more days a week**	"How physically active are you" questionnaire	Trained nurse collected data	39.2+-8.9	78.7%	Male	127	39.5	N/S	17
Al-Hazzaa, 2018[8]	Al Ateeq, 2014[74]	CS**	Stratified RS	Riyadh	Dec 2012-Feb 2013	Primary care professionals	Active category: 30 min or more a day of moderate PA, 5 or more days a week. OR 20 min or more a day of vigorous PA, 3 or more days a week**	"How physically active are you" questionnaire	Trained nurse collected data	39.2+-8.9	79%	Female	195	60.5	N/S	17

Systematic review source	Study source	Study design	Sampling method	Setting	Years of data collection	Population	Physical inactivity or sedentary behavior definition /study	Physical inactivity or sedentary behavior instrument or used items	Instrument administration	Age group (years)	Physical inactivity or sedentary behavior measurement	Gender	Sample size	♀/♂ (%)	Resp-rate (%)	QA score (/21)
Al-Hazzaa, 2018[8]	Taha, 1998[142]	N/S	RS	Eastern Province	N/S	Primary care patients	<3 times/ week for 20 min	Questionnaire	NS	36.1+-12.1	68.3%	Male and Female	227	♂<♀	N/S	12
Al-Hazzaa, 2018[8]	Taha, 1998[142]	N/S	RS	Eastern Province	N/S	Primary care patients	<3 times/ week for 20 min	Questionnaire	NS	41.5+-11.2	43.3%	Male	90	40	N/S	12
Al-Hazzaa, 2018[8]	Taha, 1998[142]	NS	RS	Eastern Province	NS	Primary care patients	<3 times/ week for 20 min	Questionnaire	NS	32.5+-11.4	84.7%	Female	137	60	N/S	12
Al-Hazzaa, 2018[8] Sharara, 2018[9]	AlQuaiz, 2009[21]	CS**	N/S	Riyadh	1 March to 30 April 2007 **	Primary care patients	Not practicing in any regular sport and leisure time physical activity**	Questionnaire	CDC web site questionnaire **	33.3+-13.3	82.4%	Male and Female	450	♂<♀	N/S	13
Al-Hazzaa, 2018[8] Sharara, 2018[9]	AlQuaiz, 2009[21]	CS**	N/S	Riyadh	1 March to 30 April 2007 **	Primary care patients	Not practicing in any regular sport and leisure time physical activity**	Questionnaire	CDC web site questionnaire **	33.3+-13.3	87.6%	Female	306	68	N/S	13
Al-Hazzaa, 2018[8] Sharara, 2018[9]	AlQuaiz, 2009[21]	CS**	N/S	Riyadh	1 March to 30 April 2007 **	Primary care patients	Not practicing in any regular sport and leisure time physical activity**	Questionnaire	CDC web site questionnaire **	33.3+-13.3	71.5%	Male	144	32	N/S	13
Al-Hazzaa, 2018[8]	Abozaid, 2010 [76]	CS**	N/S	Taif	December 2005 to January 2006**	Family clinic patients	<150 min./week moderate activity or <60 min/ week vigorous activity	IPAQ	Interview-administered	43.1+-9.3	46.5%	Male and Female	329	♂>♀	N/S	14
Al-Hazzaa, 2018[8]	Abozaid, 2010 [76]	CS**	N/S	Taif	December 2005 to January 2006**	Family clinic patients	<150 min./week moderate activity or <60 min/ week vigorous activity	IPAQ	Interview-administered	43.1+-9.3	46%	Male	190	58	N/S	14
Al-Hazzaa, 2018[8]	Abozaid, 2010 [76]	CS**	N/S	Taif	December 2005 to January 2006**	Family clinic patients	<150 min./week moderate activity or <60 min/ week vigorous activity	IPAQ	Interview-administered	43.1+-9.3	47%	Female	139	42	N/S	14

Systematic review source	Study source	Study design	Sampling method	Setting	Years of data collection	Population	Physical inactivity or sedentary behavior definition /study	Physical inactivity or sedentary behavior instrument or used items	Instrument administration	Age group (years)	Physical inactivity or sedentary behavior measurement	Gender	Sample size	♀/♂ (%)	Resp-rate (%)	QA score (/21)
Al-Hazzaa, 2018[8] / Sharara, 2018[9]	Amin, 2011[77]	CS**	Systematic RS**	Al-Hassa region	N/S	Primary healthcare patients	<600 METs-min/week	GPAQ	Trained nurse interview**	18-64	47.65%	Male and Female	2176	♂> ♀	76%	16
Al-Hazzaa, 2018[8]	Amin, 2011[77]	CS**	Systematic RS**	Al-Hassa region	N/S	Primary healthcare patients	<600 METs-min/week	GPAQ	Trained nurse interview**	32.8+-10.1	50.5%	Male	1209	55	N/S	16
Al-Hazzaa, 2018[8]	Amin, 2011[77]	CS**	Systematic RS**	Al-Hassa region	N/S	Primary healthcare patients	<600 METs-min/week	GPAQ	Trained nurse interview**	33.4+-9.2	44.8%	Female	967	45	N/S	16
Al-Hazzaa, 2018[8]	Khalid, 1995[78]	CS**	RS**	Aseer Province	N/S	Lowlanders and highlanders	No strenuous exercise for >= 3 times per week	Lipid Research Clinic Questionnaire	NS/	16-60	79.5%	Male and Female	905	N/S	N/S	12
Al-Hazzaa, 2018[8]	Khalid, 1995[78]	CS**	RS**	Aseer Province	N/S	Lowlanders and highlanders	No strenuous exercise for >= 3 times per week	Lipid Research Clinic Questionnaire	N/S	16-60	59.4%	Male	N/S	N/S	N/S	12
Al-Hazzaa, 2018[8]	Khalid, 1995[78]	CS**	RS**	Aseer Province	N/S	Lowlanders and highlanders	No strenuous exercise for >= 3 times per week	Lipid Research Clinic Questionnaire	N/S	16-60	99.5%	Female	N/S	N/S	N/S	12
Al-Hazzaa, 2018[8]	Bin Horaib, 2013[79]	CS**	MCRS**	5 regions of Saudi Arabia	January 2009 to February 2011	Military personnel	<30 min per day/ 5 days**	WHO stepwise questionnaire	Self-reported**	18-50	30.8%	Male	10229	N/A	97.4%**	20
Al-Hazzaa, 2018[8]	Al-Rafaee, 2001[80]	N/S	N/S	Riyadh	N/S	Adult population	No regular activity	Questionnaire	Self-administered	41.1+-9.7	80.9%	Male	1333	N/A	N/S	7
Al-Hazzaa 2018[8]	Al-Hazzaa, 2007[81]	N/S	N/S	Riyadh	Spring of 2003	Adult population	<600 METs-min per week	IPAQ	Telephone Interview	15-78	40.6%**	Male and Female	1064	♂> ♀	66**%	15
Al-Hazzaa 2018[8]	Al-Hazzaa, 2007[81]	N/S	N/S	Riyadh	Spring of 2003	Adult population	<600 METs-min per week	IPAQ	Telephone interview	15-78	43.7%	Male	702	66	N/A	13

Systematic review source	Study source	Study design	Sampling method	Setting	Years of data collection	Population	Physical inactivity or sedentary behavior definition /study	Physical inactivity or sedentary behavior instrument or used items	Instrument administration	Age group (years)	Physical inactivity or sedentary behavior measurement	Gender	Sample size	♀/♂ (%)	Resp-rate (%)	QA score (/21)
Al-Hazzaa 2018[8]	Al-Hazzaa, 2007[81]	N/S	N/S	Riyadh	Spring of 2003	Adult population	<600 METs-min per week	IPAQ	Telephone interview	15-78	34.3%	Female	362	34	N/A	13
Al-Hazzaa 2018[8]	Amin, 2014[82]	CS**	N/S	Al-Hassa	1st of March 2012 to May 30th, 2012	University employee	<600 METs-min per week	WHO stepwise questionnaire	Face- to-face interview**	40.4+-9.8	73%	Male and Female	691	N/A	33.1 %	15
Al-Hazzaa 2018[8]	Al-Zalabani, 2015[83]	N/S	MCRS**	National Sample	2005	Adults	<600 METs-min per week of moderate, or <60 min / week vigorous activity	GPAQ	Face- to-face interview**	15-64	66.6%	Male and Female	4758**	Equal	N/S	16
Al-Hazzaa 2018[8]	Al-Zalabani, 2015[83]	N/S	MCRS**	National Sample	2005	Adults	<600 METs-min per week of moderate, or <60 min / week vigorous activity	GPAQ	Face- to-face interview**	15-64	60.1%**	Male	2340**	49	N/S	16
Al-Hazzaa 2018[8]	Al-Zalabani, 2015[83]	N/S	MCRS**	National Sample	2005	Adults	<600 METs-min per week of moderate, or <60 min / week vigorous activity	GPAQ	Face- to-face interview**	15-64	72.9%	Female	2418	51	N/S	16
Al-Hazzaa 2018[8]	El Bcheraoui, 2016[84]	Multistage survey**	RS**	National Sample	2013	Adults	<150 MET-min/ week	IPAQ	Self-reported	18+**	66.39%	Male and Female	Unclear	N/A	89.4 %**	19
Al-Hazzaa 2018[8]	Albawardi, 2016[85]	CS**	N/S	Riyadh	N/S	Working women	<600 MET-s min per week	Modified ATLS questionnaire	Self-reported	31.7+-8.3	52.1%	Female	420	N/A	72%	13
Al-Hazzaa 2018[8]	Mandil, 2016[86]	CS**	RS**	Riyadh	Dec 2013-Jan 2014	Adults, Physicians	MVPA	Modified STEPwise questionnaire	Self-administered	24-65	36.9%	Male and Female	360	♂>♀	98.6 **	19
Al-Hazzaa 2018[8]	Al-Mountashiri, 2017[87]	Case-Control	N/S	Tabuk	Mar 2017-Jun 2017	Healthy Adults "controls"	Questionnaire	<30 min/day, 5 days/week	Face-to-face interviews	20-65+	86.7%	Male and Female	150	♂>♀	N/S	12

Systematic review source	Study source	Study design	Sampling method	Setting	Years of data collection	Population	Physical inactivity or sedentary behavior definition /study	Physical inactivity or sedentary behavior instrument or used items	Instrument administration	Age group (years)	Physical inactivity or sedentary behavior measurement	Gender	Sample size	♀/♂ (%)	Resp-rate (%)	QA score (/21)
Pakistan (number of studies = 2)																
Sisson, 2008[3]	Guthold, 2008 [11]	World Health Survey (CS**)	MCRS**	National-level	2002-03	General population	Inactivity: < 3 days of VPA at 20 min/session or <5 days MPA at 30 min/session	IPAQ short version **	Self-reported (Face-to- face interviews**)	18-69	20%***	Male and Female	5610	Equal**	85.7 **	20
Sisson, 2008[3]	Guthold, 2008 [11]	World Health Survey (CS**)	MCRS**	National-level	2002-03	General population	Inactivity: < 3 days of VPA at 20 min/session or <5 days MPA at 30 min/session	IPAQ short version **	Self-reported (Face-to- face interviews**)	18-69	14%	Male	2822***	50.3 **	N/S	17
Sisson, 2008[3]	Guthold, 2008 [11]	World Health Survey (CS**)	MCRS**	National-level	2002-03	General population	Inactivity: < 3 days of VPA at 20 min/session or <5 days MPA at 30 min/session	IPAQ short version **	Self-reported (Face-to- face interviews**)	18-69	28%	Female	2788***	49.7 **	N/S	17
Ranasinghe, 2013[5]	Khuwaja, 2010 [17]	CS**	Systematic RS	Urban regions of Karachi**	N/S	Healthy non-institutionalized population (general population)	Inactivity: (<30 minutes of moderate to vigorous activity most of the days (at least 4) in last 7 days**)	IPAQ	Face-to- face interviews**	25-64	60.1 %	Male and Female	534	Equal	88.4	17
Ranasinghe, 2013[5]	Khuwaja, 2010 [17]	CS**	Systematic RS	Urban regions of Karachi**	N/S	Healthy non-institutionalized population (general population)	Inactivity: (<30 minutes of moderate to vigorous activity most of the days (at least 4) in last 7 days**)	IPAQ	Face-to- face interviews**	25-64	52.1 %	Male	292	54.7 %**	N/S	14
Ranasinghe, 2013[5]	Khuwaja, 2010 [17]	CS**	Systematic RS	Urban regions of Karachi**	N/S	Healthy non-institutionalized population (general population)	Inactivity: (<30 minutes of moderate to vigorous activity most of the days (at least 4) in last 7 days**)	IPAQ	Face-to- face interviews**	25-64	69.8 %	Female	242	45.3 %**	N/S	14
Tunisia (number of studies = 2)																

Systematic review source	Study source	Study design	Sampling method	Setting	Years of data collection	Population	Physical inactivity or sedentary behavior definition /study	Physical inactivity or sedentary behavior instrument or used items	Instrument administration	Age group (years)	Physical inactivity or sedentary behavior measurement	Gender	Sample size	♀/♂ (%)	Resp-rate (%)	QA score (/21)
Sisson, 2008[3]; Sharara, 2018[9]	Guthold, 2008 [11]	World Health Survey (CS**)	MCRS**	National-level	2002-03	General population	Inactivity: < 3 days of VPA at 20 min/session or <5 days MPA at 30 min/session	IPAQ short version **	Self-reported (Face-to- face interviews**)	18-69	14.6%***	Male and Female	4332	Equal**	90.1 **	20
Sisson, 2008[3]; Sharara, 2018[9]	Guthold, 2008 [11]	World Health Survey (CS**)	MCRS**	National-level	2002-03	General population	Inactivity: < 3 days of VPA at 20 min/session or <5 days MPA at 30 min/session	IPAQ short version **	Self-reported (Face-to- face interviews**)	18-69	12%	Male	2149***	49.6 **	N/S	17
Sisson, 2008[3]; Sharara, 2018[9]	Guthold, 2008 [11]	World Health Survey (CS**)	MCRS**	National-level	2002-03	General population	Inactivity: < 3 days of VPA at 20 min/session or <5 days MPA at 30 min/session	IPAQ short version **	Self-reported (Face-to- face interviews**)	18-69	19%	Female	2183***	50.4 **	N/S	17
Sharara, 2018[9]	Maatoug, 2013[26]	CS**	Cluster sampling**	Subnational level (Sousse; Household) **	2009	Adults	<150 min/week of moderate level of PA	Oxford Health Alliance Community Intervention for Health Project	Personal interviews**	Mean: 37.9	44.4%	Male and Female **	1880	♂< ♀**	73.3 **	17
UAE (number of studies = 5)																
Sisson, 2008[3]; Sharara, 2018[9]	Guthold, 2008 [11]	World Health Survey (CS**)	MCRS**	National-level	2002-03	General population	Inactivity: < 3 days of VPA at 20 min/session or <5 days MPA at 30 min/session	IPAQ short version **	Self-reported (Face-to- face interviews**)	18-69	43.2%***	Male and Female	1104	♂> ♀**	94.9 **	19
Sisson, 2008[3]; Sharara, 2018[9]	Guthold, 2008 [11]	World Health Survey (CS**)	MCRS**	National-level	2002-03	General population	Inactivity: < 3 days of VPA at 20 min/session or <5 days MPA at 30 min/session	IPAQ short version **	Self-reported (Face-to- face interviews**)	18-69	40%	Male	796***	72.1 **	N/S	16
Sisson, 2008[3]; Sharara, 2018[9]	Guthold, 2008 [11]	World Health Survey (CS**)	MCRS**	National-level	2002-03	General population	Inactivity: < 3 days of VPA at 20 min/session or	IPAQ short version **	Self-reported (Face-to- face interviews**)	18-69	59%	Female	308***	27.9 **	N/S	16

Systematic review source	Study source	Study design	Sampling method	Setting	Years of data collection	Population	Physical inactivity or sedentary behavior definition /study	Physical inactivity or sedentary behavior instrument or used items	Instrument administration	Age group (years)	Physical inactivity or sedentary behavior measurement	Gender	Sample size	♀/♂ (%)	Resp-rate (%)	QA score (/21)
							<5 days MPA at 30 min/session									
Yammine, 2016[6]	Amine, 1996 [13]	N/S	RS	UAE University **	N/S	University students	Nil practice of PA – (Never participated in physical exercises**)	Personal physical activity questionnaire	Face-to- face interviews with students or their parents**	17-24	48.5%	Female	206	N/A	N/S	12
Yammine, 2016[6]	Amine, 1996 [13]	N/S	RS	UAE University **	N/S	University students	Mild level of PA- (Mild participation in physical exercises**)	Personal physical activity questionnaire	Face-to- face interviews with students or their parents**	17-24	47.1%	Female	206	N/A	N/S	11
Sharara, 2018[9]	Abdulle, 2006[27]	Case control study**	N/S	Subnational level (N/S)	2001-2005	Normotensive Controls	Less than one hour, <3 times per week	Questionnaire**	Interview**	20-75	39.4%	Male and Female **	424	♂> ♀**	N/R	11
Sharara, 2018[9]	McIlvenny, 2000[28]	N/S	Systematic RS**	Subnational level (clinic) **	N/S	Adult attenders at a Family Medicine clinic	No regular exercise	Questionnaire**	Interview**	18-94	54.0%	Male and Female **	254	♂< ♀**	N/R	10
Sharara, 2018[9]	Sabri, 2004[29]	Case control study**	MCRS**	Subnational level (clinic) **	2001-2002	Normotensive Controls	< 1 hour/week of sport	Questionnaire**	Interview**	20-65	47.5%	Male and Female **	436	Equal**	N/R	14
Kuwait (number of studies = 6)																
Mabry, 2010[4]	Ministry of Health, Kuwait, 2006 [171]	World Health Survey (CS**)	RSS	National-level*	2006**	Kuwaiti general population	Mean amount of time spent sitting	IPAQ	Face-to- face interviews	20-65	234.6 min d-1*	Male and Female	2280	♂< ♀	77.6	19
Mabry, 2010[4]	Ministry of Health, Kuwait, 2006 [171]	World Health Survey (CS**)	RSS	National-level*	2006**	Kuwaiti general population	Mean amount of time spent sitting	IPAQ	Face-to- face interviews	20-65	252 min d-1	Male	912*	40	72.2	19
Mabry, 2010[4]	Ministry of Health, Kuwait, 2006 [171]	World Health Survey (CS**)	RSS	National-level*	2006**	Kuwaiti general population	Mean amount of time spent sitting	IPAQ	Face-to- face interviews	20-66	223 min d-1	Female	1368*	60	81.6	20
Sharara, 2018[9]	Ahmed, 2013[30]	Kuwait National Nutrition Surveill	N/S	National-level*	2002-2009	Adults Kuwaiti	No deliberate non-work related exercise outside the home such as	N/S	N/S	20+	68.4%	Male and Female **	6356**	♂< ♀**	95**	10

Systematic review source	Study source	Study design	Sampling method	Setting	Years of data collection	Population	Physical inactivity or sedentary behavior definition /study	Physical inactivity or sedentary behavior instrument or used items	Instrument administration	Age group (years)	Physical inactivity or sedentary behavior measurement	Gender	Sample size	♀/♂ (%)	Resp-rate (%)	QA score (/21)
		ance System					walking, running or cycling									
Sharara, 2018[9]	Al-Zenki, 2012[31]	Survey **	MCRS**	National level (6 PHCC)	2008-2009	Adults Kuwaiti	(Neither moderately nor very Active) Lightly active**	N/S	Self-reported **	20+	57.9%	Male and Female **	1092 **	♂< ♀**	N/R	13
Sharara, 2018[9]	Alarouj, 2013[32]	CS**	RSS**	National level (6 governorates)	N/S	Kuwaiti nationals	(Neither moderate nor vigorous PA) MPA was considered if participants engaged in physical efforts that cause light sweating or a slight increase in breathing or heart rate; examples of MPA included, brisk walking, painting houses, gardening and climbing stairs**	WHO STEPS questionnaire** (GPAQ)	Interviews**	20-65	63%	Male and Female **	1970	♂< ♀**	67**	16
Sharara, 2018[9]	Naser Al-Isa, 2011[33]	CS**	RS**	Subnational level (University)	N/S	Kuwaiti college students	Not engaging in regular PA	Questionnaire**	Self-reported **	N/S	45.0%	Male and Female **	787	N/S	N/R	11
Sharara, 2018[9]	WHO-STEPS survey, 2014[34]	CS**	MCRS**	National level	2014	Adults	<600 MET-min/week	GPAQ	Face-to- face interviews**	18-69	62.6%	Male and Female **	4391	♂< ♀**	89.2 **	19
Sharara, 2018[9]	WHO-STEPS survey, 2014[175]	CS**	MCRS**	National level	2014	Adults	<600 MET-min/week	GPAQ	Face-to- face interviews**	18-69	51.4%**	Male	1429**	37.2 **	N/S	16
Sharara, 2018[9]	WHO-STEPS survey, 2014[34]	CS**	MCRS**	National level	2014	Adults	<600 MET-min/week	GPAQ	Face-to- face interviews**	18-69	72.8%**	Female	2413**	62.8 **	N/S	16
Oman (number of studies = 2)																

Systematic review source	Study source	Study design	Sampling method	Setting	Years of data collection	Population	Physical inactivity or sedentary behavior definition /study	Physical inactivity or sedentary behavior instrument or used items	Instrument administration	Age group (years)	Physical inactivity or sedentary behavior measurement	Gender	Sample size	♀/♂ (%)	Resp-rate (%)	QA score (/21)
Mabry, 2016[7]	El-Aty, 2014 [170]	Oman World Health Survey**	MCRS**	National level**	2008**	Adults (Omani household**)	Sedentary (6+ hrs/d)	GPAQ	N/S	18+	23.70%	Male and Female	3137	♂<♀**	86.3**	18
Mabry, 2016[7]	El-Aty, 2014 [170]	Oman World Health Survey**	MCRS**	National level**	2008**	Adults (Omani household**)	Sedentary (6+ hrs/d)	GPAQ	N/S	18+	21.5%	Male	1459**	46.5**	N/S	15
Mabry, 2016[7]	El-Aty, 2014 [170]	Oman World Health Survey**	MCRS**	National level**	2008**	Adults (Omani household**)	Sedentary (6+ hrs/d)	GPAQ	N/S	18+	25.6%	Female	1678**	53.5**	N/S	15
Mabry, 2016[7]	Mabry, 2012 [176]	Sur Healthy Lifestyle Survey Cross-sectional**	MCRS**	Sur**	2006**	Adults (Omani men and non-pregnant women**)	Sedentary (3+ hrs/d)	GPAQ	Face-to- face interviews**	20+	45.30%	Male and Female	1335	♂<♀**	97.2**	18
Mabry, 2016[7]	Mabry, 2012 [176]	Sur Healthy Lifestyle Survey Cross-sectional**	MCRS**	Sur**	2006**	Adults (Omani men and non-pregnant women**)	Sedentary (3+ hrs/d)	GPAQ	Face-to- face interviews**	20+	64.8%	Male	591**	44.3**	N/S	15
Mabry, 2016[7]	Mabry, 2012 [176]	Sur Healthy Lifestyle Survey Cross-sectional**	MCRS**	Sur**	2006**	Adults (Omani men and non-pregnant women**)	Sedentary (3+ hrs/d)	GPAQ	Face-to- face interviews**	20+	37.8%	Female	744**	55.7**	N/S	15
Qatar (number of studies = 3)																
Mabry, 2016[7]	Al Thani, 2015 [140]	Qatar STEPwise survey**	MCRS**	National level **	2012**	Qatari** women	Mean total sitting time (min/d)	GPAQ	Face-to- face interviews**	18-64	183.6 ± 168.3 min/d	Female	747	N/A	88**	20

Systematic review source	Study source	Study design	Sampling method	Setting	Years of data collection	Population	Physical inactivity or sedentary behavior definition /study	Physical inactivity or sedentary behavior instrument or used items	Instrument administration	Age group (years)	Physical inactivity or sedentary behavior measurement	Gender	Sample size	♀/♂ (%)	Res p-rate (%)	QA score (/21)
Sharara, 2018[9]	Al-Nakeeb, 2015[35]	CS**	N/S	Qatar University **	N/S	Young adults	<840 MET-min/week	Self-report questionnaire**	Face-to- face interviews**	Mean = 21.2	50. 8	Male and Female **	732	N/S	N/S	10
Sharara, 2018[9]	WHO-STEPS survey, 2012[141]	CS**	MCRS**	National level	2012	Qatari adults**	<600 MET-min/week	GPAQ	Face-to- face interviews**	18-64	45.9	Male and Female **	2442 (n=)	♂< ♀*	88**	20
Sharara, 2018[9]	WHO-STEPS survey, 2012[141]	CS**	MCRS**	National level	2012	Qatari adults**	<600 MET-min/week	GPAQ	Face-to- face interviews**	18-64	37.4**	Male	1030**	42.2 **	N/S	17
Sharara, 2018[9]	WHO-STEPS survey, 2012[141]	CS**	MCRS**	National level	2012	Qatari adults**	<600 MET-min/week	GPAQ	Face-to- face interviews**	18-64	54.2**	Female	1412**	57.8 **	N/S	17
Jordan (number of studies = 6)																
Sharara, 2018[9]	Zindah, 2008[37]	Behavioral Risk Factor Surveillance System	MCRS**	National level (Household)	2004	Jordanian adults **	Not engaging in moderate activity (resulting in light sweating, small increases in breathing or heart rate)	Questionnaire**	Interviews**	18+	51.8%	Males and Females**	710	N/S	85.2 **	17
Sharara, 2018[9]	Centers for Disease Control, Prevention, 2003[38]	Jordan Behavioral Risk Factor Survey	MCRS**	Sub national level (Household) **	2002	Jordanian adults **	Less than having moderate: activity that caused light sweating and small increases in heart rate or breathing for 30 min	Questionnaire**	Interviews**	18+	47.4	Male and Female **	8791	N/S	92**	18
Sharara, 2018[9]	Mohannad, 2008[42]	CS**	MCRS**	Sub national level (Household) **	2002	Jordanian residents**	No activity that caused light sweating and small increases in heart rate or breathing	Questionnaire**	Interviews**	40+	58.7	Male and Female **	3083	Equal**	95.8 **	17
Sharara, 2018[9]	Kulwicki, 2001[39]	Nonexperimental	Systematic Rando	Sub national level	N/S	Jordanian residents**	No exercise	Questionnaire**	Interviews**	17-93	22.5%	Male and	209	N/S	N/S	11

Systematic review source	Study source	Study design	Sampling method	Setting	Years of data collection	Population	Physical inactivity or sedentary behavior definition /study	Physical inactivity or sedentary behavior instrument or used items	Instrument administration	Age group (years)	Physical inactivity or sedentary behavior measurement	Gender	Sample size	♀/♂ (%)	Resp-rate (%)	QA score (/21)
		survey**	m Sampling**	(Household) **								Female **				
Sharara, 2018[9]	Madanat, 2006[40]	CS**	All students invited**	Sub national level (University) **	2003	University Students**	<30 min of PA 3 or more days/week/ Week**	Questionnaire**	Self-reported**	Mean: 21.1	81.5%	Male and Female **	431	N/S	98**	17
Sharara, 2018[9]	WHO-STEPS survey, 2007[41]	CS**	MCRS	National level	2007	Adults	<600 MET-min/week	GPAQ	Face-to- face interviews**	18+	5.2	Male and Female	3654	♂>.♀**	99.1	19
Sharara, 2018[9]	WHO-STEPS survey, 2007[41]	CS**	MCRS**	National level	2007	Adults	<600 MET-min/week	GPAQ	Face-to- face interviews**	18+	5.8**	Male	1939**	53.1 **	N/S	16
Sharara, 2018[9]	WHO-STEPS survey, 2007[41]	CS**	MCRS**	National level	2007	Adults	<600 MET-min/week	GPAQ	Face-to- face interviews**	18+	4.5**	Female	1715**	46.9 **	N/S	16
Egypt (number of studies = 4)																
Sharara, 2018[9]	WHO-STEPS survey, 2011-12[43]	CS**	MCRS**	National level	2012	Adults	<600 MET-min/week	GPAQ	Face-to- face interviews**	15-64	32.1%	Male and Female **	5300	N/S	95**	19
Sharara, 2018[9]	WHO-STEPS survey, 2011-12[43]	CS**	MCRS**	National level	2012	Adults	<600 MET-min/week	GPAQ	Face-to- face interviews**	15-64	23.3%**	Male	N/S	N/S	N/S	16
Sharara, 2018[9]	WHO-STEPS survey, 2011-12[43]	CS**	MCRS**	National level	2012	Adults	<600 MET-min/week	GPAQ	Face-to- face interviews**	15-64	42.0%**	Female	N/S	N/S	N/S	16
Sharara, 2018[9]	Abolfotouh, 2007[44]	N/S	Multistage sampling**	Subnational level (Alexandria) **	2002-2003	Students living in university hostels	(No non-VPA for at least 20 min or 3 times per week) No exercise **	Questionnaire**	Self-reported **	17-25	33.8%	Male and Female **	600	N/S	94**	17
Sharara, 2018[9]	Kamel, 2013[45]	CS**	Continuous recruitment**	Subnational level (PHCC) **	2010-2011	Elderly people**	Not active**	Questionnaire**	Self-reported **	60+	63.8%	Male and Female **	340	N/S	N/S	12

Systematic review source	Study source	Study design	Sampling method	Setting	Years of data collection	Population	Physical inactivity or sedentary behavior definition /study	Physical inactivity or sedentary behavior instrument or used items	Instrument administration	Age group (years)	Physical inactivity or sedentary behavior measurement	Gender	Sample size	♀/♂ (%)	Resp-rate (%)	QA score (/21)
Sharara, 2018[9]	Mahfouz, 2014[46]	Case control study**	RS**	Subnational level (El-Mina oncology centre) **	2011	Cases of colorectal cancer receiving treatment**	No exercise	Questionnaire**	Self-reported **	<20->60	100%	Male and Female **	150	N/S	N/S	13
Sharara, 2018[9]	Mahfouz, 2014[46]	Case control study**	RS**	Subnational level (El-Mina oncology centre) **	2011	Age and sex matched controls**	No exercise	Questionnaire**	Self-reported **	<20->60	78.3%	Male and Female **	300	N/S	N/S	13
Iraq (number of studies = 1)																
Sharara, 2018[9]	WHO-STEPS survey, 2015[47]	CS**	MCRS**	National level	2015	Adults	<600 MET-min/week	GPAQ	Face-to- face interviews**	18+	47%	Male and Female **	4120	♂<♀**	98.8	19
Sharara, 2018[9]	WHO-STEPS survey, 2015[47]	CS**	MCRS**	National level	2015	Adults	<600 MET-min /week	GPAQ	Face-to- face interviews**	18+	34.9%**	Male	1568**	39.3 **	N/S	16
Sharara, 2018[9]	WHO-STEPS survey, 2015[47]	CS**	MCRS**	National level	2015	Adults	<600 MET-min/week	GPAQ	Face-to- face interviews**	18+	60.0%**	Female	2420**	60.7 **	N/S	16
Libya (number of studies = 1)																
Sharara, 2018[9]	WHO-STEPS survey, 2009[48]	CS**	MCRS**	National level	2009	Adults	<600 MET-min/week	GPAQ	Face-to- face interviews**	25-64	43.9%	Male and Female **	3590	Equal**	N/S	16
Sharara, 2018[9]	WHO-STEPS survey, 2009[48]	CS**	MCRS**	National level	2009	Adults	<600 MET-min/week	GPAQ	Face-to- face interviews**	25-64	36.0%**	Male	1800**	50.1 **	N/S	16
Sharara, 2018[9]	WHO-STEPS survey, 2009[48]	CS**	MCRS**	National level	2009	Adults	<600 MET-min/week	GPAQ	Face-to- face interviews**	25-64	51.7%**	Female	1790**	49.9 **	N/S	16
Lebanon (number of studies = 6)																
Sharara, 2018[9]	WHO-STEPS survey, 2009[49]	CS**	MCRS **	National level	2008-2009	Adults	<600 MET-min/week	GPAQ	Face-to- face interviews**	25-64	45.8%	Male and Female **	1982	♂<♀**	N/S	16

Systematic review source	Study source	Study design	Sampling method	Setting	Years of data collection	Population	Physical inactivity or sedentary behavior definition /study	Physical inactivity or sedentary behavior instrument or used items	Instrument administration	Age group (years)	Physical inactivity or sedentary behavior measurement	Gender	Sample size	♀/♂ (%)	Res p-rate (%)	QA score (/21)
Sharara, 2018[9]	WHO-STEPS survey, 2009[49]	CS**	MCRS**	National level	2008-2009	Adults	<600 MET-min/week	GPAQ	Face-to- face interviews**	25-64	52.2%**	Male	893**	45.1 **	N/S	16
Sharara, 2018[9]	WHO-STEPS survey, 2009[49]	CS**	MCRS**	National level	2008-2009	Adults	<600 MET-min/week	GPAQ	Face-to- face interviews**	25-64	40.3%**	Female	1089**	54.9 **	N/S	16
Sharara, 2018[9]	Al-Tannir, 2008[50]	CS**	Convenience sampling**	Subnational level	2007	Adults	Less than 3 days/week	Questionnaire**	Self-reported**	18+	42.7%**	Male and Female **	346	N/S	N/S	13
Sharara, 2018[9]	Musharrafieh, 2008[51]	CS**	RS**	Subnational level (5 universities) **	2001	University students**	Physical exercise for <0.5 h/week	Questionnaire**	Interviews**	Mean: 21.0	73.6%	Male and Female **	2013	N/S	N/S	14
Sharara, 2018[9]	Tamim, 2003[52]	CS**	RS**	Subnational level (5 universities) **	2000-2001	University students**	<3 hours/week	Questionnaire**	Interviews**	Mean: 21.0	64.3%	Male and Female **	1964	N/S	N/S	16
Sharara, 2018[9]	Farah, 2015[53]	CS**	MCRS**	National level	2013-2014	Adult Lebanese residents**	Neither moderate intensity PA for at least 150 min per week or vigorous intensity PA for 75 min at least per week	Questionnaire**	Face-to- face interviews**	40+	76.0	Male and Female **	1515	N/S	N/S	16
Sharara, 2018[9]	Tohme, 2005[54]	CS**	MCRS**	National level	2003-2004	Adults	Never engaging in any PA	Questionnaire**	Face-to- face interviews**	30+	40.3	Male and Female **	2010	Equal**	95**	16
Palestine (number of studies = 3)																
Sharara, 2018[9]	WHO-STEPS survey, 2010-11[55]	CS**	MCRS	National level	2010-2011	Adults	<600 MET-min/week	GPAQ	Face-to- face interviews**	15-64	46.5%	Male and Female **	6957	N/S	N/S	16
Sharara, 2018[9]	WHO-STEPS survey, 2010-11[55]	CS**	MCRS	National level	2010-2011	Adults	<600 MET-min/week	GPAQ	Face-to- face interviews**	15-64	33.8%**	Male	N/S	N/S	N/S	16
Sharara, 2018[9]	WHO-STEPS	CS**	MCRS	National level	2010-2011	Adults	<600 MET-min/week	GPAQ	Face-to- face interviews**	15-64	59.2%**	Female	N/S	N/S	N/S	16

Systematic review source	Study source	Study design	Sampling method	Setting	Years of data collection	Population	Physical inactivity or sedentary behavior definition /study	Physical inactivity or sedentary behavior instrument or used items	Instrument administration	Age group (years)	Physical inactivity or sedentary behavior measurement	Gender	Sample size	♀/♂ (%)	Resp-rate (%)	QA score (/21)
	survey, 2010-11[55]															
Sharara, 2018[9]	Abdul-Rahim, 2003[56]	CS**	N/S	Subnational level (Rural and urban Ramallah)	N/S	Adults	Occupation-related sedentarylight PA for men AND no exercise for women	Questionnaire	Interviews**	30-65	56.2%	Male and Female **	936	N/S	N/S	7
Sharara, 2018[9]	Abu-Mourad, 2008[57]	CS**	Multistage RSS**	Subnational level (PHCCs, Gaza Strip) **	2005	Patients attending PHCC**	No home exercise or sports	Questionnaire**	N/S	18+	78.0%	Male and Female **	956	N/S	N/S	13
Algeria (number of studies = 1)																
Sharara, 2018[9]	WHO-STEPS suzvey, 2003[58]	CS**	MCRS**	Subnational level	2003	Adults	<600 MET-min/week	GPAQ	Face-to- face interviews**	25-64	40.7%	Male and Female **	4102	♂<. ♀**	N/S	16
Sharara, 2018[9]	WHO-STEPS survey, 2003[58]	CS**	MCRS**	Subnational level	2003	Adults	<600 MET-min/week	GPAQ	Face-to- face interviews**	25-64	32.5%**	Male	N/S	39**	N/S	16
Sharara, 2018[9]	WHO-STEPS survey, 2003[58]	CS**	MCRS**	Subnational level	2003	Adults	<600 MET-min/week	GPAQ	Face-to- face interviews**	25-64	45.8%**	Female	N/S	61**	N/S	16
Sudan (number of studies = 1)																
Sharara, 2018[9]	WHO-STEPS survey, 2005-2006[59]	CS**	MCRS**	Subnational level	2005-2006	Adults	<600 MET-min/week	GPAQ	Face-to- face interviews**	25-64	86.8%	Male and Female **	1573	♂<. ♀**	98.3 **	19
Sharara, 2018[9]	WHO-STEPS survey, 2005-2006[59]	CS**	MCRS**	Subnational level	2005-2006	Adults	<600 MET-min/week	GPAQ	Face-to- face interviews**	25-64	75.9%**	Male	652**	41.4 **	N/S	16
Sharara, 2018[9]	WHO-STEPS survey, 2005-2006[59]	CS**	MCRS**	Subnational level	2005-2006	Adults	<600 MET-min/week	GPAQ	Face-to- face interviews**	25-64	94.8%**	Female	921**	58.6 **	N/S	16

Systematic review source	Study source	Study design	Sampling method	Setting	Years of data collection	Population	Physical inactivity or sedentary behavior definition /study	Physical inactivity or sedentary behavior instrument or used items	Instrument administration	Age group (years)	Physical inactivity or sedentary behavior measurement	Gender	Sample size	♀/♂ (%)	Resp-rate (%)	QA score (/21)
Morocco (number of studies = 3)																
Sharara, 2018[9]	El Rhazi, 2011[60]	CS**	MCRS**	National level	2008	Adults	Less than 30 min of regular, moderate or intense PA on most days**	Questionnaire**	Face-to- face interviews**	18+	38.7% (n= 1014)	Male and Female **	2620	Equal**	N/S	16
Sharara, 2018[9]	El Rhazi, 2011[60]	CS**	MCRS	National level	2008	Adults	Less than 30 min of regular, moderate or intense PA on most days**	Questionnaire	Face-to- face interviews	18+	32.4% (n= 441)	Male	1359**	51.9 **	N/S	16
Sharara, 2018[9]	El Rhazi, 2011[60]	CS**	MCRS**	National level	2008	Adults	Less than 30 min of regular, moderate or intense PA on most days**	Questionnaire**	Face-to- face interviews**	18+	45.4% (n= 573) **	Female	1261**	48.1 **	N/S	16
Sharara, 2018[9]	Najdi, 2011[61]	CS**	MCRS**	National level (urban and rural) **	2008	Moroccan adults**	<600 METmin/wk**	IPAQ	Face-to- face interviews**	18-99	16.5%	Male and Female **	2613	N/S	96.5 **	20
Bahrain (number of studies = 1)																
Sharara, 2018[9]	Hamadeh, 2000[62]	CS**	RCS**	Subnational level (household) **	N/S	Bahraini residents**	No exercise	Questionnaire**	Interviews**	30-79	89.1%	Male and Female	516	N/S	99.2 **	14
Syria (number of studies = 1)																
Sharara, 2018[9]	Al Ali, 2011[63]	2nd Aleppo Household Survey	Two-stage cluster sampling**	Subnational level	2006	Adults	Less than 15 min/ week of sport or brisk walking	Questionnaire**	Interviews**	25+	82.3%	Male and Female **	1168	N/S	N/S	14

Notes: All non-reported data was searched and extracted from the original study. Any additional information found relevant in the original study was added to the reported data for the purpose of completeness.
If not reported, the prevalence of the outcome among the total study population (males and/or females) was calculated using row or calculated data available in the original study. The calculated prevalence measure was reported and marked using two stars (**).
If not reported, the total sample size for each gender strata was calculated based on the percentage of males or females in the sample.
If not reported, the number for cases in each gender strata was calculated based on the reported prevalence and the total sample size in the strata.
If not reported, the total number for cases in the entire sample was calculated by the addition of the number of cases in each reported stratum.
For not reported mean time measures on the total study population, the overall males and females weighted mean time was calculated using mean time and the proportion of males and females in the sample, respectively.
If any discordance between the reported data in the SR and data available in the original study, this later was retained.

Extra studies are studies included in a SR and reporting one or more PA related outcome on MENA countries that were not reported in the SR (outcome or country not included in the SR). Extra data from these studies was extracted and used for the qualitative and quantitative synthesis.
Converted data from physical inactivity to PA was used only for the quantitative synthesis and was not reported in the above tables.

Abbreviations: SR: Systematic review; PA: Physical activity; VPA: Vigorous physical activity: MPA: Moderate physical activity; KSA: Kingdom of Saudi Arabia; UAE: United Arab Emirates; USA: United States of America; GCC: Gulf cooperation council; MENA: Middle-East and North Africa; QA: Quality assessment; ATLS: Arab teens lifestyle student questionnaire; GPAQ: Global Physical Activity Questionnaire; IPAQ: International Physical Activity Questionnaire: CS: Cross sectional: MCRS: Multistage stratified/cluster random sampling; PHCC: Primary Health Care Centre; RS: Random sampling: RCS: Random cluster sampling; RSS: Random stratified sampling; MET: Metabolic Equivalent of Task: VG: Video games; Resp-rate: response rate; N/S: Not stated; N/A: Not applicable;
† The use of a validated questionnaire was not reported in the original study.
‡ The use of Nurses' Health Study II questionnaire was not reported in the original study.
* Calculated from reported data in the SR
**Calculated or reported from data in the original study
***Calculated from the prevalence measure reported in the original study (different from the prevalence measure reported in the SR)
♂: Males; ♀: Females; km: Kilometer; min: Minutes; wk: Week; d: Day: d-1: Per day; hrs: hours; h: hour
MJ: The megajoule is equal to one million (106) joules; Energy expenditure of >0.3 MJ/day is equivalent to walking daily for half an hour at 5 km/h.

Table S11: Physical inactivity and sedentary behavior among the youth general population of the included MENA countries.

Systematic review source	Study source	Study design	Sampling method	Setting	Years of data collection	Population	Physical inactivity or sedentary behavior definition /study	Physical inactivity or sedentary behavior instrument or used items	Instrument administration	Age group (years)	Physical inactivity or sedentary behavior measurement	Gender	Sample size	♀/♂ (%)	Resp - rate (%)	QA score (/21)
Saudi Arabia (number of studies = 28)																
Mabry, 2016[7]	Al-Hazzaa, 2011 [124]	Population-based survey (CS**)	MCRS**	Al-Khobar, Jeddah and Riyadh **	2009-10**	Saudi** secondary-school students	Computer/TV time >2 hrs/d	ATLS	Self-administrated**	14-19	87.7%**	Male and Female*	2908	Equal**	N/S	15
Mabry, 2016[7]	Al-Hazzaa, 2011 [124]	Population-based survey (CS**)	MCRS**	Al-Khobar, Jeddah and Riyadh **	2009-10**	Saudi** secondary-school students	Computer/TV time >2 hrs/d	ATLS	Self-administrated**	14-19	84.0%	Male	1401**	48.2**	N/S	15
Mabry, 2016[7]	Al-Hazzaa, 2011 [124]	Population-based survey (CS**)	MCRS**	Al-Khobar, Jeddah and Riyadh **	2009-10**	Saudi** secondary-school students	Computer/TV time >2 hrs/d	ATLS	Self-administrated**	14-19	91.2%	Female	1507**	51.8**	N/S	15
Mabry, 2016[7]	Al-Hazzaa, 2013 [144]	Population-based survey (CS**)	MCRS**	Al-Khobar, Jeddah and Riyadh **	2009-10**	Saudi** school students	Computer/TV time >3 hrs/d	ATLS	Self-administrated**	15-19	76.0%**	Male and Female	1373**	48.7**	N/S	15
Mabry, 2016[7]	Al-Hazzaa, 2013 [144]	Population-based survey (CS**)	MCRS**	Al-Khobar, Jeddah and Riyadh **	2009-10**	Saudi** school students	Computer/TV time >3 hrs/d	ATLS	Self-administrated**	15-19	69.8%	Male	1373**	48.7**	N/S	15
Mabry, 2016[7]	Al-Hazzaa, 2013 [144]	Population-based survey (CS**)	MCRS**	Al-Khobar, Jeddah and Riyadh **	2009-10**	Saudi** school students	Computer/TV time >3 hrs/d	ATLS	Self-administrated**	15-19	81.8%	Female	1449**	51.3**	N/S	15
Mabry, 2016[7]	Al-Hazzaa, 2013[145]	Population-based survey (CS**)	MCRS**	Al-Khobar and Riyadh **	N/S	Saudi** school students	Mean computer/TV time (hrs/d)	ATLS	N/S	14-18	5.6h**	Male and Female*	1648	Equal**	N/S	13
Mabry, 2016[7]	Al-Hazzaa, 2013 [145]	Population-based survey (CS**)	MCRS**	Al-Khobar and Riyadh **	N/S	Saudi** school students	Mean computer/TV time (hrs/d)	ATLS	N/S	14-18	5.31 ± 3.1 hrs/d	Male	797**	48.4**	N/S	13

Systematic review source	Study source	Study design	Sampling method	Setting	Years of data collection	Population	Physical inactivity or sedentary behavior definition /study	Physical inactivity or sedentary behavior instrument or used items	Instrument administration	Age group (years)	Physical inactivity or sedentary behavior measurement	Gender	Sample size	♀/♂ (%)	Resp - rate (%)	QA score (/21)
Mabry, 2016[7]	Al-Hazzaa, 2013 [145]	Population-based survey (CS**)	MCRS**	Al-Khobar and Riyadh **	N/S	Saudi** school students	Mean computer/TV time (hrs/d)	ATLS	N/S	14-18	5.89 ± 3.3 hrs/d	Female	851**	51.6**	N/S	13
Mabry, 2016[7]	Al-Hazzaa, 2013 [145]	Population-based survey (CS**)	MCRS**	Al-Khobar and Riyadh **	N/S	Saudi** school students	Computer/TV time >2 hrs/d	ATLS	N/S	14-18	87.9 %	Male and Female	1648	Equal**	N/S	12
Mabry, 2016[7]	Al-Hazzaa, 2013 [145]	Population-based survey (CS**)	MCRS**	Al-Khobar and Riyadh **	N/S	Saudi** school students	Computer/TV time >2 hrs/d	ATLS	N/S	14-18	84.2 %	Male	797**	48.4**	N/S	12
Mabry, 2016[7]	Al-Hazzaa, 2013 [145]	Population-based survey (CS**)	MCRS**	Al-Khobar and Riyadh **	N/S	Saudi** school students	Computer/TV time >2 hrs/d	ATLS	N/S	14-18	91.6 %	Female	851**	51.6**	N/S	12
Mabry, 2016[7]	Al-Hazzaa, 2007 [144]	Population-based survey (CS**)	MCRS**	Jeddah**	2006**	Saudi ** preschool children	Mean computer/TV time (min/d)	Questionnaire to parents**	Self-administrated**	3.4-6.4	154.8 ± 66.1 min/d	Male and Female	224	Equal**	75**	17
Mabry, 2016[7]	Al-Hazzaa, 2007 [144]	Population-based survey (CS**)	MCRS**	Jeddah**	2006**	Saudi ** preschool children	Mean computer/TV time (min/d)	Questionnaire to parents**	Self-administrated**	3.4-6.4	162.4 ± 69.9 min/d	Male	109**	48.7**	N/S	15
Mabry, 2016[7]	Al-Hazzaa, 2007 [144]	Population-based survey (CS**)	MCRS**	Jeddah**	2006**	Saudi ** preschool children	Mean computer/TV time (min/d)	Questionnaire to parents**	Self-administrated**	3.4-6.4	147.7 ± 61.7 min/d	Female	115**	51.3**	N/S	15
Mabry, 2016[7]	Al-Nakeeb, 2012 [146]	Population-based survey	RSS**	Al-Ahsa**	N/S	School students	Mean time watching TV (hrs/d)	ATLS	Self-administrated**	15-17	2.56** hrs/d	Male and Female*	2290 (UK+KSA) 1107 (from KSA) **	Equal**	N/S	12
Mabry, 2016[7]	Al-Nakeeb, 2012 [146]	Population-based survey	RSS**	Al-Ahsa**	N/S	School students	Mean time watching TV (hrs/d)	ATLS	Self-administrated**	15-17	2.51 hrs/d (±1.81**) hrs/d	Male	576**	52.0**	N/S	12
Mabry, 2016[7]	Al-Nakeeb, 2012 [146]	Population-based survey	RSS**	Al-Ahsa**	N/S	School students	Mean time watching TV (hrs/d)	ATLS	Self-administrated**	15-17	2.61 hrs/d (±1.91**)	Female	531**	48.0**	N/S	12

Systematic review source	Study source	Study design	Sampling method	Setting	Years of data collection	Population	Physical inactivity or sedentary behavior definition /study	Physical inactivity or sedentary behavior instrument or used items	Instrument administration	Age group (years)	Physical inactivity or sedentary behavior measurement	Gender	Sample size	♀/♂ (%)	Resp - rate (%)	QA score (/21)
Mabry, 2016[7]	Al-Nakeeb, 2012 [146]	Population-based survey	RSS**	Al-Ahsa**	N/S	School students	Mean computer time (hrs/d)	ATLS	Self-administrated**	15-17	2.83 hrs/d **	Male and Female*	2290 (UK+ KSA)<br><br>1107 (from KSA) **	Equal**	N/S	12
Mabry, 2016[7]	Al-Nakeeb, 2012 [146]	Population-based survey	RSS**	Al-Ahsa**	N/S	School students	Mean computer time (hrs/d)	ATLS	Self-administrated**	15-17	2.41 hrs/d (2.48±2.07 hrs/d **)	Male	576**	52.0**	N/S	12
Mabry, 2016[7]	Al-Nakeeb, 2012 [146]	Population-based survey	RSS**	Al-Ahsa**	N/S	School students	Mean computer time (hrs/d)	ATLS	Self-administrated**	15-17	3.18 hrs/d (±2.07**)	Female	531**	48.0**	N/S	12
Mabry, 2016[7]	Al-Nuaim, 2012 [147]	Population-based survey (CS**)	RS**	Al-Ahsa**	N/S	Secondary ** school students	Mean time watching TV (hrs/d)	ATLS	Self-administrated**	15-19	2.54 hrs/d **	Male and Female*	1270	Equal**	N/S	12
Mabry, 2016[7]	Al-Nuaim, 2012 [147]	Population-based survey (CS**)	RS**	Al-Ahsa**	N/S	Secondary ** school students	Mean time watching TV (hrs/d)	ATLS	Self-administrated**	15-19	2.49 hrs/d (±1.80**)	Male	663**	52.2**	N/S	12
Mabry, 2016[7]	Al-Nuaim, 2012 [147]	Population-based survey (CS**)	RS**	Al-Ahsa**	N/S	Secondary ** school students	Mean time watching TV (hrs/d)	ATLS	Self-administrated**	15-19	2.60 hrs/d (±1.90**)	Female	607**	47.8**	N/S	12
Mabry, 2016[7]	Al-Nuaim, 2012 [147]	Population-based survey (CS**)	RS**	Al-Ahsa**	N/S	Secondary ** school students	Mean computer time (hrs/d)	ATLS	Self-administrated**	15-19	2.81 hrs/d **	Male and Female*	1270	Equal**	N/S	12
Mabry, 2016[7]	Al-Nuaim, 2012 [147]	Population-based survey (CS**)	RS**	Al-Ahsa**	N/S	Secondary ** school students	Mean computer time (hrs/d)	ATLS	Self-administrated**	15-19	2.43 hrs/d (±2.07**)	Male	663**	52.2**	N/S	12
Mabry, 2016[7]	Al-Nuaim, 2012 [147]	Population-based survey (CS**)	RS**	Al-Ahsa**	N/S	Secondary ** school students	Mean computer time (hrs/d)	ATLS	Self-administrated**	15-19	3.19 hrs/d (±2.40**)	Female	607**	47.8**	N/S	12
Mabry, 2016[7]	Farghaly, 2007 [177]	Population-based survey (CS**)	Two-stage RS**	Abha**	2000**	Saudi** Students	Mean TV time (hrs/d)	Designed questionnaire**	Self-reported by students or parents**	7-20	1.0± 1.0 hrs/d	Male and Female	767	♂<♀**	N/S	15

Systematic review source	Study source	Study design	Sampling method	Setting	Years of data collection	Population	Physical inactivity or sedentary behavior definition /study	Physical inactivity or sedentary behavior instrument or used items	Instrument admini-stration	Age group (years)	Physical inactivity or sedentary behavior measurement	Gender	Sample size	♀/♂ (%)	Resp - rate (%)	QA score (/21)
Mabry, 2016[7]	Farghaly, 2007 [177]	Population-based survey (CS**)	Two-stage RS**	Abha**	2000**	Saudi** Students	Mean computer game time (hrs/d)	Designed questionnaire**	Self-reported by students or parents**	7-20	0.7 ± 0.9 hrs/d	Male and Female	767	♂<♀**	N/S	15
Mabry, 2016[7]	Mahfouz, 2011 [92]	Population-based survey (CS**)	RSS**	Abha and Ahad Rufeida**	2008**	Adolescent (School students**)	Watched > 3hrs TV/d	CDC Adolescent Health adapted questionnaire (Validated Arabic version**)	Face-to- face interviews**	11-19	42.9 %**	Male and Female*	1869	♂>♀**	N/S	13
Mabry, 2016[7]	Mahfouz, 2011 [92]	Population-based survey (CS**)	RSS**	Abha and Ahad Rufeida**	2008**	Adolescent (School students**)	Watched > 3hrs TV/d	CDC Adolescent Health adapted questionnaire (Validated Arabic version**)	Face-to- face interviews**	11-19	38.0 %	Male	1249**	66.8**	96.7 **	16
Mabry, 2016[7]	Mahfouz, 2011 [92]	Population-based survey (CS**)	RSS**	Abha and Ahad Rufeida**	2008**	Adolescent (School students**)	Watched > 3hrs TV/d	CDC Adolescent Health adapted questionnaire (Validated Arabic version**)	Face-to- face interviews**	11-19	52.7 %	Female	620**	33.2**	97.1 **	16
Mabry, 2016[7]	Mahfouz, 2008 [92]	Population-based survey	RSS**	Abha**	2005**	Adolescent school boys	Watched > 3hrs TV/d	Arabic version of CDC Adolescent Health Survey	Face-to- face interviews**	11-19	38.0 %	Male	2696	N/A	96.7 for intermediate schools 97.1 for second	16

Systematic review source	Study source	Study design	Sampling method	Setting	Years of data collection	Population	Physical inactivity or sedentary behavior definition /study	Physical inactivity or sedentary behavior instrument or used items	Instrument admini-stration	Age group (years)	Physical inactivity or sedentary behavior measurement	Gender	Sample size	♀/♂ (%)	Resp - rate (%)	QA score (/21)
															ary schools **	
Mabry, 2016[7]	Musaiger, 2013 [178]	Population-based survey (CS**)	MCRS**	Jeddah**	2005-06**	Girl school students	≥3 hrs TV time/d	Validated questionnaire on the frequency of intake of certain food items and lifestyle**	N/S	12-19	60.9 %	Female	512	N/A	N/S	13
Sharara, 2018[9]	Al-Hazzaa, 2011[124]	Arab Teens Lifestyle Study: CS	MRSS**	Subnational level (three major cities in Saudi Arabia) **	2009-2010	Students**	<1680 METs-min/week	ATLS	Self-reported**	15-19	61.9%	Male and Female**	2908	N/S	N/S	16
Sharara, 2018[9]	AlBuhairan, 2015[88]	School-based national CS study**	Stratified Cluster RS**	National level (278 Schools) **	N/S	Students from intermediate and secondary schools**	Complete absence of exercise	YRBSS and the GSHS Questionnaires	Self-administered	Mean: 15.8	45.2%	Male and Female**	12575	Equal**	32.7 **	12
Sharara, 2018[9]	Al-Muhaimeed, 2015[89]	CS**	Multistage RS**	Subnational level (primary schools) **	2012	Students in grade 1-4**	Not engaging in sports	Questionnaire**	Reported by parents**	6-10	27.3%	Male and Female**	601	N/S	N/S	13
Sharara, 2018[9]	Al-Mutairi, 2015 [90]	CS**	RS**	Subnational level (4 Schools) **	2013	Adolescents from secondary schools**	No regular exercise	Questionnaire**	N/S	15-22	31.9%	Male and Female**	426	Equal**	N/S	13
Sharara, 2018[9]	Al-Othman, 2012[91]	CS**	N/S	Subnational level (4 PHCCs) **	2010	Children and adolescents**	Based on weekly frequency: Never	Questionnaire**	Self-reported**	6-17	15.7%	Male and Female**	331	♂<♀**	N/S	11
Sharara, 2018[9]	Mahfouz, 2011[92]	CS**	RSS**	Subnational level	2008	Students from Ist,	Less than 30 min of	CDC Adolescent	Interviews**	11-19	34.3%	Male and	1869	♂>♀ **	97**	18

Systematic review source	Study source	Study design	Sampling method	Setting	Years of data collection	Population	Physical inactivity or sedentary behavior definition /study	Physical inactivity or sedentary behavior instrument or used items	Instrument administration	Age group (years)	Physical inactivity or sedentary behavior measurement	Gender	Sample size	♀/♂ (%)	Resp - rate (%)	QA score (/21)
				(Schools) **		2nd and 3rd year**	physical exercise during the previous week	Health Survey Questionnaire				Female**				
Al-Hazzaa, 2018[8]	Al-Hazzaa, 2011 [124]	Population-based survey (CS**)	MCRS**	Al-Khobar, Jeddah and Riyadh **	2009-10**	Adolescents**	<1680 METs-min per week	ATLS	Self-administrated**	N/S	61.3%	Male and Female*	2908	Equal **	N/S	16
Al-Hazzaa, 2018[8]	Al-Hazzaa, 2011 [124]	Population-based survey (CS**)	MCRS**	Al-Khobar, Jeddah and Riyadh **	2009-10**	Adolescents**	<1680 METs-min per week	ATLS	Self-administrated**	16.7+-1.1	44.5%	Male	1401	48.2**	N/S	16
Al-Hazzaa, 2018[8]	Al-Hazzaa, 2011 [124]	Population-based survey (CS**)	MCRS**	Al-Khobar, Jeddah and Riyadh **	2009-10**	Adolescents**	<1680 METs-min per week	ATLS	Self-administrated**	16.5+-1.1	78.1%	Female	1507	51.8**	N/S	16
Al -Hazzaa 2018[8]	Al-Hazzaa, 2007 [125]	CS**	RS**	Jeddah	Apr and May 2006**	Preschoolers	<10000 step counts per day	Electronic pedometer	Objective measurement**	5.2+-0.8	77.2%**	Male and Female	224	♂<♀	75 %**	20
Al -Hazzaa 2018[8]	Al-Hazzaa, 2007 [125]	CS**	RS**	Jeddah	Apr and May 2006**	Preschoolers	<10000 step counts per day	Electronic pedometer	Objective measurement**	5.2+-0.8	72.9%	Male	109	48.6	N/S	18
Al -Hazzaa 2018[8]	Al-Hazzaa, 2007 [125]	CS**	RS**	Jeddah	Apr and May 2006**	Preschoolers	<10000 step counts per day	Electronic pedometer	Objective measurement**	5.2+-0.8	81.4%	Female	115	51.4	N/S	18
Al -Hazzaa 2018[8]	Al-Hazzaa, 1993 [126]	N/S	N/S	Riyadh	N/S	School children	Daily heart rate <159 bpm for atleast 20 min/day	Continuous heart rate monitoring	Objective measurement**	9.5+-1.4	85%	Male	212	N/A	68.75%**	13
Al-Hazzaa, 2018[8]	Al Hazzaa, 2002 [127]	N/S	N/S	Riyadh	N/S	School children	Daily heart rate <140 bpm for atleast 30min/day	Continuous heart rate monitoring	Objective measurement**	9.6+-1.5	57.1%	Male	92	N/A	N/S	11

Systematic review source	Study source	Study design	Sampling method	Setting	Years of data collection	Population	Physical inactivity or sedentary behavior definition /study	Physical inactivity or sedentary behavior instrument or used items	Instrument admini-stration	Age group (years)	Physical inactivity or sedentary behavior measurement	Gender	Sample size	♀/♂ (%)	Resp - rate (%)	QA score (/21)
Al-Hazzaa, 2018[8]	Al Hazzaa, 2007 [128]	N/S	RS**	Riyadh	N/S	School children	<13000 step counts	Electronic pedometer	Objective measurement**	10.3+-1.3	47.1%	Male	296	N/A	N/S	14
Al-Hazzaa, 2018[8]	Al-Kutbe, 2017 [129]	CS**	N/S	Makkah	Jan-May 2014**	School children	<10000 step counts/ day	Accelerometer	Objective measurement**	8-11	Low 6757 step/day (20.2 min/ day of MVPA)	Female	78	N/A	65.3 %**	16
Al-Hazzaa, 2018[8]	Al-Rukban, 2003 [130]	CS**	MCRS	Riyadh	September 2001-January 2002 ** %	Intermediate and secondary school students **	<30 min for <4 days/ week	Questionnaire Internally validated**	Self-administered **	15.7+-1.8	72.3%	Male	894	N/A	N/S	15
Al-Hazzaa, 2018[8]	Taha, 2008 [131]	CS**	Multistage stratified self-weighing sampling **	Al-Khobar	2001-2002**	School students**	Any bodily movement produced by skeletal muscles that resulted in energy expenditure above the basal level for at least 20 minutes per session** <3 times/ week	Questionnaire	Self-reported**	16.3+-1.7	60.3%**	Male and female	2571	♂<♀	N/S	12
Al-Hazzaa, 2018[8]	Taha,2008 [131]	CS**	Multistage stratified self-weighing sampling **	Al-Khobar	2001-2002**	Adolescents	Any bodily movement produced by skeletal muscles that resulted in energy expenditure above the basal level for at least 20 minutes	Questionnaire	Self-reported**	16.3+-1.7	54.4%	Male	1240	48.2	84.1 - 100 %	15

Systematic review source	Study source	Study design	Sampling method	Setting	Years of data collection	Population	Physical inactivity or sedentary behavior definition /study	Physical inactivity or sedentary behavior instrument or used items	Instrument admini-stration	Age group (years)	Physical inactivity or sedentary behavior measurement	Gender	Sample size	♀/♂ (%)	Resp - rate (%)	QA score (/21)
							per session** <3 times/ week									
Al-Hazzaa, 2018[8]	Taha, 2008 [131]	CS**	Multistage stratified self-weighing sampling **	Al-Khobar	2001-2002**	Adolescents	Any bodily movement produced by skeletal muscles that resulted in energy expenditure above the basal level for at least 20 minutes per session** <3 times/ week	Questionnaire	Self-reported**	16.3+-1.7	66.3%	Female	1331	51.8	89.6 - 100 %	15
Al-Hazzaa, 2018[8]	Mahfouz, 2012 [132]	CS**	MCRS**	Aseer	N/S	Adolescents (students**)	<30 min of PA in previous week	Validated CDC Adolescent Health survey** Questionnaire	Face-to-face interviews**	12-19**	31.4%**	Male and female	1869	♂>♀	N/S	13
Al-Hazzaa, 2018[8]	Mahfouz, 2012 [132]	CS**	MCRS**	Aseer	N/S	Adolescents (students**)	<30 min of PA in previous week	Validated CDC Adolescent Health survey** Questionnaire	Face-to-face interviews**	17.2+-1.2	25.7%	Male	1249	66.8	96.7 **	16
Al-Hazzaa, 2018[8]	Mahfouz, 2012 [132]	CS**	MCRS**	Aseer	N/S	Adolescents (students**)	<30 min of PA in previous week	Validated CDC Adolescent Health survey** Questionnaire	Face-to-face interviews**	12-19	42.9%	Female	620	33.2	97.1 **	16
Al-Hazzaa, 2018[8]	Alzahrani, 2014 [133]	Health Behaviour in School-aged	MCRS**	Riyadh	N/S	Adolescents	<60 min, 5 day/week	Questionnaire validated in Arabic	Self-administered **	13,14, 17-18	63.7%	Male	1335	N/A	100 %	16

Systematic review source	Study source	Study design	Sampling method	Setting	Years of data collection	Population	Physical inactivity or sedentary behavior definition /study	Physical inactivity or sedentary behavior instrument or used items	Instrument admini-stration	Age group (years)	Physical inactivity or sedentary behavior measurement	Gender	Sample size	♀/♂ (%)	Resp - rate (%)	QA score (/21)
		children (HSBC)						internally validated**								
Al-Hazzaa, 2018[8]	Alsubaie, 2015 [134]	CS**	MCRS**	Riyadh	N/S	Adolescents	Moderate to vigorous PA for 60 min/ day ** <5 days a week	Standard ** 30-day recall questionnaire	Self-administered **	15-18	20.1%	Male	453	N/A	N/S	12
Al-Hazzaa, 2018[8]	Bajamal, 2017 [135]	CS**	SRS**	Jeddah	Jan-Mar 2016**	Adolescents	Sum of moderate to vigorous activity score during the past 7 days**	PAQ-A	Self-administered **	13-18	low	Female	405	N/A	98.8 **	20
Al-Hazzaa, 2018[8]	Al-Raddadi, 2018 [136]	CS**	RS**	Jeddah	N/S	Adolescents	<150 mins per week	Questionnaire	Self-administered **	17.2+-1.2	86.1%	Female	410	N/A	N/S	12
Al-Hazzaa, 2018[8]	El Bcheraoui, 2013 [84]	Multistage survey**	RS**	National Sample	2013	Adolescents	<420 MET-min/week	IPAQ	Self-reported **	15-17	55.71%**	Male and Female	Unclear	N/A	89.4 %**	20
UAE (number of studies = 6)																
Yammine, 2016[6]	Wasfi, 2008 [16]	Survey**	RSS**	Bur Dubai and Deira**	2004**	Secondary school students	Nil practice of PA- (Did not practice physical activity at all**)	Personal PA questionnaire	Self-administrated**	14-18	21.2 %**	Male and Female	1475	♂>♀	98.3 **	18
Yammine, 2016[6]	Wasfi, 2008 [16]	Survey**	RSS**	Bur Dubai and Deira**	2004**	Secondary school students	Nil practice of PA- (Did not practice physical activity at all**)	Personal PA questionnaire	Self-administrated**	14-18	10.7 %	Male	835	56.6**	N/S	15
Yammine, 2016[6]	Wasfi, 2008 [16]	Survey**	RSS**	Bur Dubai and Deira**	2004**	Secondary school students	Nil practice of PA- (Did not practice physical activity at all**)	Personal PA questionnaire	Self-administrated**	14-18	35.0 %	Female	640	43.4**	N/S	15

Systematic review source	Study source	Study design	Sampling method	Setting	Years of data collection	Population	Physical inactivity or sedentary behavior definition /study	Physical inactivity or sedentary behavior instrument or used items	Instrument administration	Age group (years)	Physical inactivity or sedentary behavior measurement	Gender	Sample size	♀/♂ (%)	Resp - rate (%)	QA score (/21)
Yammine, 2016[6]	Mehairi, 2013 [14]	CS**	RS	Al Ain**	2010**	School students	Mild level of PA – (Low score of PA**)	IPAQ short version	Self-administrated**	12-18	34.4 % **	Male and Female	1018**	Equal	68.3**	16
Yammine, 2016[6]	Mehairi, 2013 [14]	CS**	RS	Al Ain**	2010**	School students	Mild level of PA – (Low score of PA**)	IPAQ short version	Self-administrated**	12-18	24.1 %	Male	522	51.6**	N/S	14
Yammine, 2016[6]	Mehairi, 2013 [14]	CS**	RS	Al Ain**	2010**	School students	Mild level of PA – (Low score of PA**)	IPAQ short version	Self-administrated**	12-18	45.1 %	Female	496	48.4**	N/S	14
Yammine, 2016[6]	Muhairi, 2013 [15]	CS**	RS	Al Ain**	2010**	School students	Mild level of PA – (Low score of PA**)	IPAQ short version	Self-administrated**	15-18	36.5 %	Male and Female	315	Equal	N/S	14
Yammine, 2016[6]	Wasfi, 2008 [16]	Survey**	RSS**	Bur Dubai and Deira**	2004**	Secondary school students	Mild level of PA – (No vigorous activity or irregularly practiced vigorous exercise < 60 min/wk and < 30 min of moderate PA most days of the week**)	Personal PA questionnaire	Self-administrated**	14-18	48.8 %*	Male and Female	1475*	♂>♀	N/S	15
Yammine, 2016[6]	Wasfi, 2008 [16]	Survey**	RSS**	Bur Dubai and Deira**	2004**	Secondary school students	Mild level of PA – (No vigorous activity or irregularly practiced vigorous exercise < 60 min/wk and < 30 min of moderate PA most days of the week**)	Personal PA questionnaire	Self-administrated**	14-18	51.6 %	Male	835	56.6**	N/S	15

Systematic review source	Study source	Study design	Sampling method	Setting	Years of data collection	Population	Physical inactivity or sedentary behavior definition /study	Physical inactivity or sedentary behavior instrument or used items	Instrument admini-stration	Age group (years)	Physical inactivity or sedentary behavior measurement	Gender	Sample size	♀/♂ (%)	Resp - rate (%)	QA score (/21)
Yammine, 2016[6]	Wasfi, 2008 [16]	Survey**	RSS**	Bur Dubai and Deira**	2004**	Secondary school students	Mild level of PA – (No vigorous activity or irregularly practiced vigorous exercise < 60 min/wk and < 30 min of moderate PA most days of the week**)	Personal PA questionnaire	Self-administrated**	14-18	45.2 %	Female	640	43.4**	N/S	15
Yammine, 2016[6]	Wasfi, 2008 [16]	Survey**	RSS**	Bur Dubai and Deira**	2004**	Emirati secondary school students	Nil PA practice – (Did not practice PA at all**)	Personal PA questionnaire	Self-administrated**	14-18	18.9 %	Male and Female	233	Unclear	N/S	16
Yammine, 2016[6]	Wasfi, 2008 [16]	Survey**	RSS**	Bur Dubai and Deira**	2004**	Emirati secondary school students residing in UAE	Mild level of PA – (No vigorous activity or irregularly practiced vigorous exercise < 60 min/wk and < 30 min of moderate PA most days of the week**)	Personal PA questionnaire	Self-administrated**	14-18	34.3 %	Male and Female	233	Unclear	N/S	16
Yammine, 2016[6]	Wasfi, 2008 [16]	Survey**	RSS**	Bur Dubai and Deira**	2004**	Non-Emirati secondary school students residing in UAE	Nil PA practice – (Did not practice physical activity at all**)	Personal PA questionnaire	Self-administrated**	14-18	34.3 %	Male and Female	1242	Unclear	N/S	16
Yammine, 2016[6]	Wasfi, 2008 [16]	Survey**	RSS**	Bur Dubai and Deira**	2004**	Non-Emirati secondary school students	Mild level of PA - (No vigorous activity or irregularly	Personal PA questionnaire	Self-administrated**	14-18	51.5 %	Male and Female	1242	Unclear	N/S	16

Systematic review source	Study source	Study design	Sampling method	Setting	Years of data collection	Population	Physical inactivity or sedentary behavior definition /study	Physical inactivity or sedentary behavior instrument or used items	Instrument administration	Age group (years)	Physical inactivity or sedentary behavior measurement	Gender	Sample size	♀/♂ (%)	Resp - rate (%)	QA score (/21)
						residing in UAE	practiced vigorous exercise < 60 min/wk and < 30 min of moderate PA most days of the week**)									
Mabry, 2016[7]	Yousef, 2013 [179]	Population-based survey	MCRS**	Al-Ain**	2007-08**	Public** schools' children (UAE-native **)	% TV viewing/Video games > 2 hrs/d	Questions related to TV viewing/VG use time**	Self-administrated**	6-10	62.9 % (for less than 2h TV**) 37.1 %** (for more than 2h TV) **	Male and Female	197	♂>♀**	93.4 **	17
Mabry, 2016[7]	Yousef, 2013 [179]	Population-based survey	MCRS**	Al-Ain**	2007-08**	Public** schools' children (UAE-native **)	% TV viewing/Video games > 2 hrs/d	Questions related to TV viewing/VG use time**	Self-administrated**	6-10	38.5 %**	Male	130**	66**	N/S	14
Mabry, 2016[7]	Yousef, 2013 [179]	Population-based survey	MCRS**	Al-Ain**	2007-08**	Public** schools' children (UAE-native **)	% TV viewing/Video games > 2 hrs/d	Questions related to TV viewing/VG use time**	Self-administrated**	6-10	34.3 %**	Female	67**	34**	N/S	14
Yammine, 2016[6]	Guthold, 2010 $ [10]	GSHS**	Two-stage RS	National level	2005	Schoolchildren	% sitting and watching TV, computer games, talking with friends or another sitting activities ≥ 3 hrs /d	Validated PACE+ and Adolescent physical activity measure questionnaire	Self-administrated	14.0 (mean)	42 %	Male and Female**	9916**	Equal	88.3 %	16
Yammine, 2016[6]	Guthold, 2010 $ [10]	GSHS**	Two-stage RS	National level	2005	Schoolchildren	% sitting and watching TV, computer	Validated PACE+ and Adolescent	Self-administrated	14.0 (mean)	41 %	Male	4849	48.9%	N/S	13

Systematic review source	Study source	Study design	Sampling method	Setting	Years of data collection	Population	Physical inactivity or sedentary behavior definition /study	Physical inactivity or sedentary behavior instrument or used items	Instrument admini-stration	Age group (years)	Physical inactivity or sedentary behavior measurement	Gender	Sample size	♀/♂ (%)	Resp - rate (%)	QA score (/21)
							games, talking with friends or another sitting activities ≥ 3 hrs /d	physical activity measure questionnaire								
Yammine, 2016[6]	Guthold, 2010 $ [10]	GSHS**	Two-stage RS	National level	2005	Schoolchildren	% sitting and watching TV, computer games, talking with friends or another sitting activities ≥ 3 hrs /d	Validated PACE+ and Adolescent physical activity measure questionnaire	Self-administrated	14.0 (mean)	43 %	Female	5067	51.1%	N/S	13
Sharara, 2018[9]	GSHS, 2010[94]	School-based survey	Two stage cluster sample**	National level	2010	Students in grade 8,9 and 10**	< 60 min per day on five or more days during the past seven days	PACE+	Self-report on answer sheet**	13-15	72.5%	Male and Female**	2038	N/S	91**	19
Sharara, 2018[9]	GSHS, 2010[94]	School-based survey	Two stage cluster sample**	National level	2010	Students in grade 8,9 and 10**	< 60 min per day on five or more days during the past seven days	PACE+	Self-report on answer sheet**	13-15	65.5%**	Male	N/S	N/S	N/S	16
Sharara, 2018[9]	GSHS, 2010[94]	School-based survey	Two stage cluster sample**	National level	2010	Students in grade 8,9 and 10**	< 60 min per day on five or more days during the past seven days	PACE+	Self-report on answer sheet**	13-15	77.2%**	Female	N/S	N/S	N/S	16
Oman (number of studies = 5)																
Mabry, 2016[7]	Kilani, 2013 [173]	Population-based survey (CS**)	MCRS**	Muscat**	2010**	Adolescents (Secondary-school students **)	Mean hours of computer time/wk	ATLS	Face-to- face interviews**	15-18	3.3 hrs/wk	Male and Female*	802**	N/S	N/S	15

Systematic review source	Study source	Study design	Sampling method	Setting	Years of data collection	Population	Physical inactivity or sedentary behavior definition /study	Physical inactivity or sedentary behavior instrument or used items	Instrument admini-stration	Age group (years)	Physical inactivity or sedentary behavior measurement	Gender	Sample size	♀/♂ (%)	Resp - rate (%)	QA score (/21)
Mabry, 2016[7]	Kilani, 2013 [173]	Population-based survey (CS**)	MCRS**	Muscat**	2010**	Adolescents (Secondary-school students **)	Mean hours of computer time/wk	ATLS	Face-to- face interviews**	15-18	3.3 hrs/wk	Male	360**	44.9**	N/S	15
Mabry, 2016[7]	Kilani, 2013 [173]	Population-based survey (CS**)	MCRS**	Muscat**	2010**	Adolescents (Secondary-school students **)	Mean hours of computer time/wk	ATLS	Face-to- face interviews**	15-18	2.7 hrs/wk	Female	442**	55.1**	N/S	15
Mabry, 2016[7]	Kilani, 2013 [173]	Population-based survey (CS**)	MCRS**	Muscat**	2010**	Adolescents (Secondary-school students **)	Mean screen time (hrs/d)	ATLS	Face-to- face interviews**	15-18	3.31 hrs/d**	Male and Female*	802	♂<♀**	N/S	15
Mabry, 2016[7]	Kilani, 2013 [173]	Population-based survey (CS**)	MCRS**	Muscat**	2010**	Adolescents (Secondary-school students **)	Mean screen time (hrs/d)	ATLS	Face-to- face interviews**	15-18	2.86 ±SD 2.3 hrs/d	Male	360**	44.9**	N/S	15
Mabry, 2016[7]	Kilani, 2013 [173]	Population-based survey (CS**)	MCRS**	Muscat**	2010**	Adolescents (Secondary-school students **)	Mean screen time (hrs/d)	ATLS	Face-to- face interviews**	15-18	3.70 ±SD 2.9 hrs/d	Female	442**	55.1**	N/S	15
Mabry, 2016[7]	Youssef, 2013 [180]	Population-based survey (CS**)	MCRS**	Muscat**	2011**	Omani** secondary-school students	% TV time ≥3 hrs/d	Pre-tested questionnaire**	Self-administrated**	15-20	23.2 %	Male and Female	439	Equal**	N/S	14
Mabry, 2016[7]	Youssef, 2013 [180]	Population-based survey (CS**)	MCRS**	Muscat**	2011**	Omani** secondary-school students	% TV time ≥3 hrs/d	Pre-tested questionnaire**	Self-administrated**	15-20	21.1 %	Male	214**	48.7**	N/S	14
Mabry, 2016[7]	Youssef, 2013 [180]	Population-based survey (CS**)	MCRS**	Muscat**	2011**	Omani** secondary-school students	% TV time ≥3 hrs/d	Pre-tested questionnaire**	Self-administrated**	15-20	25.3 %	Female	225**	51.3**	N/S	14

Systematic review source	Study source	Study design	Sampling method	Setting	Years of data collection	Population	Physical inactivity or sedentary behavior definition /study	Physical inactivity or sedentary behavior instrument or used items	Instrument admini-stration	Age group (years)	Physical inactivity or sedentary behavior measurement	Gender	Sample size	♀/♂ (%)	Resp - rate (%)	QA score (/21)
Mabry, 2016[7]	Youssef, 2013 [180]	Population-based survey (CS**)	MCRS**	Muscat**	2011**	Omani** secondary-school students	% TV time ≥3 hrs/d	Pre-tested questionnaire**	Self-administrated**	15-20	29.20 %	Male and Female	439	Equal**	N/S	14
Mabry, 2016[7]	Youssef, 2013 [180]	Population-based survey (CS**)	MCRS**	Muscat**	2011**	Omani** secondary-school students	% TV time ≥3 hrs/d	Pre-tested questionnaire**	Self-administrated**	15-20	26.6 %	Male	214**	48.7**	N/S	14
Mabry, 2016[7]	Youssef, 2013 [180]	Population-based survey (CS**)	MCRS**	Muscat**	2011**	Omani** secondary-school students	% TV time ≥3 hrs/d	Pre-tested questionnaire**	Self-administrated**	15-20	31.5 %	Female	225**	51.3**	N/S	14
Yammine, 2016[3]	Guthold, 2010 $[10]	GSHS**	Two-stage RS**	National level**	2005**	Schoolchildren**	% sitting and watching TV, computer games, talking with friends or another sitting activities ≥ 3 hrs /d**	Validated PACE+ and Adolescent physical activity measure questionnaire**	Self-administrated**	14 (mean)**	29.7%**	Male and Female **	2158**	Equal**	97 %**	13
Yammine, 2016[3]	Guthold, 2010 $ [10]	GSHS**	Two-stage RS**	National level**	2005**	Schoolchildren**	% sitting and watching TV, computer games, talking with friends or another sitting activities ≥ 3 hrs /d**	Validated PACE+ and Adolescent physical activity measure questionnaire**	Self-administrated**	14 (mean)**	34 %**	Male**	1133**	52.5**	N/S **	13
Yammine, 2016[3]	Guthold, 2010 $ [10]	GSHS**	Two-stage RS**	National level**	2005**	Schoolchildren**	% sitting and watching TV, computer games, talking with friends or another sitting	Validated PACE+ and Adolescent physical activity measure questionnaire**	Self-administrated**	14 (mean)**	25 %**	Female**	1025**	47.5**	N/S **	13

Systematic review source	Study source	Study design	Sampling method	Setting	Years of data collection	Population	Physical inactivity or sedentary behavior definition /study	Physical inactivity or sedentary behavior instrument or used items	Instrument administration	Age group (years)	Physical inactivity or sedentary behavior measurement	Gender	Sample size	♀/♂ (%)	Resp - rate (%)	QA score (/21)
							activities ≥ 3 hrs /d**									
Sharara, 2018[9]	GSHS, 2015[95]	School-based survey	Two-stage cluster sample**	National level (school) **	2015	Students in grade 8-12**	< 60 min per day on all seven days during the past seven days	PACE+	Self-reported**	13-17	88.3%	Male and Female**	3468	N/S	92**	19
Sharara, 2018[9]	GSHS, 2015[95]	School-based survey	Two-stage cluster sample**	National level (school) **	2015	Students in grade 8-12**	< 60 min per day on all seven days during the past seven days	PACE+	Self-reported**	13-17	84.6%**	Male	N/S	N/S	N/S	16
Sharara, 2018[9]	GSHS, 2015[95]	School-based survey	Two-stage cluster sample**	National level (school) **	2015	Students in grade 8-12 **	< 60 min per day on all seven days during the past seven days	PACE+	Self-reported**	13-17	91.7%**	Female	N/S	N/S	N/S	16
Sharara, 2018[9]	Afifi, 2006[96]	School-based survey	MSRS**	National level	2004	Students from secondary schools**	Engaging in physical activities <once per week, apart from school physical education	Health practices scale **	Self-reported**	14-20	66.3%	Male and Female**	5409	Equal**	96.31**	17
Bahrain (number of studies = 2)																
Mabry, 2016[7]	Gharib, 2008 [181]	Population-based survey (CS**)	MCRS**	Eleven populated regions**	1999-01**	Bahraini primary, intermediate and secondary ** school students	Mean hours of TV/Video/wk	Pilot-tested questionnaire**	Self-reported by students or parents**	43269	21.35 * hrs/wk *	Male and Female*	2594	Equal**	N/S	15
Mabry, 2016[7]	Gharib, 2008 [181]	Population-based survey (CS**)	MCRS**	Eleven populated regions**	1999-01**	Bahraini primary, intermediate and secondary **	Mean hours of TV/Video/wk	Pilot-tested questionnaire**	Self-reported by students or parents**	43269	11.5 hrs/wk	Male	1268**	48.8**	N/S	15

Systematic review source	Study source	Study design	Sampling method	Setting	Years of data collection	Population	Physical inactivity or sedentary behavior definition /study	Physical inactivity or sedentary behavior instrument or used items	Instrument administration	Age group (years)	Physical inactivity or sedentary behavior measurement	Gender	Sample size	♀/♂ (%)	Resp - rate (%)	QA score (/21)
						school students										
Mabry, 2016 [7]	Gharib, 2008 [181]	Population-based survey (CS**)	MCRS**	Eleven populated regions**	1999-01**	Bahraini primary, intermediate and secondary** school students	Mean hours of TV/Video/wk	Pilot-tested questionnaire**	Self-reported by students or parents**	43269	31.2	Female	1326 hrs/wk **	51.1**	N/S	15
Sharara, 2018[9]	Musaiger, 2014[97]	CS**	MCRS**	National level	(2006–2007	Students in classes at levels 10, 11 and 12**	<5days/week of playing sport	Questionnaire**	Self-reported**	15-18	72.1%	Male and Female**	735	Equal**	N/S	14
Kuwait (number of studies = 3)																
Mabry, 2016[7]	Allafi, 2013 [174]	Population-based survey (CS**)	MCRS**	Six Kuwaiti governorates**	N/S	Adolescents (Kuwaiti secondary school students**)	% watch >2 hrs of TV/d	ATLS	Self-administrated**	14-19	71.2 %**	Male and Female*	906	Equal**	N/S	12
Mabry, 2016[7]	Allafi, 2013 [174]	Population-based survey (CS**)	MCRS**	Six Kuwaiti governorates**	N/S	Adolescents (Kuwaiti secondary school students**)	% watch >2 hrs of TV/d	ATLS	Self-administrated**	14-19	69.7 %	Male	463**	51.1**	N/S	12
Mabry, 2016[7]	Allafi, 2013 [174]	Population-based survey (CS**)	MCRS**	6 Kuwaiti governorates**	N/S	Adolescents (Kuwaiti secondary school students**)	% watch >2 hrs of TV/d	ATLS	Self-administrated**	14-19	72.7 %	Female	443**	48.9**	N/S	12
Mabry, 2016[7]	Allafi, 2013 [174]	Population-based survey (CS**)	MCRS**	6 Kuwaiti governorates**	N/S	Adolescents (Kuwaiti secondary school	% use computers >2 hrs/d	ATLS	Self-administrated**	14-19	66 %**	Male and Female*	906	Equal**	N/S	12

Systematic review source	Study source	Study design	Sampling method	Setting	Years of data collection	Population	Physical inactivity or sedentary behavior definition /study	Physical inactivity or sedentary behavior instrument or used items	Instrument administration	Age group (years)	Physical inactivity or sedentary behavior measurement	Gender	Sample size	♀/♂ (%)	Resp - rate (%)	QA score (/21)
						students**)										
Mabry, 2016[7]	Allafi, 2013 [174]	Population-based survey (CS**)	MCRS**	6 Kuwaiti governorates**	N/S	Adolescents (Kuwaiti secondary school students**)	% use computers >2 hrs/d	ATLS	Self-administrated**	14-19	62.1 %	Male	463**	51.1**	N/S	12
Mabry, 2016[7]	Allafi, 2013 [174]	Population-based survey (CS**)	MCRS**	6 Kuwaiti governorates**	N/S	Adolescents (Kuwaiti secondary school students**)	% use computers >2 hrs/d	ATLS	Self-administrated**	14-19	70.0 %	Female	443**	48.9**	N/S	12
Sharara, 2018[9]	GSHS, 2015[98]	School-based survey	Two-stage cluster sample**	National level	2015	Students in grade 8-11**	< 60 min per day on all seven days during the past seven days	PACE+	Self-reported**	13-17	82.9%	Male and Female**	3637	N/S	78**	18
Sharara, 2018[9]	GSHS, 2015[98]	School-based survey	Two-stage cluster sample**	National level	2015	Students in grade 8-11**	< 60 min per day on all seven days during the past seven days	PACE+	Self-reported**	13-17	81.0%**	Male	N/S	N/S	N/S	15
Sharara, 2018[9]	GSHS, 2015[98]	School-based survey	Two-stage cluster sample**	National level	2015	Students in grade 8-11 **	< 60 min per day on all seven days during the past seven days	PACE+	Self-reported**	13-17	84.9%**	Female	N/S	N/S	N/S	15
Sharara, 2018[9]	Shehab, 2005[99]	CS**	Multistage RSS**	Subnational level	N/S	Students from 5th to 12th grades**	Only performing normal daily routine with some recreational activities or walking slowly and	Questionnaire**	Interviews**	10-18	71.3%	Male and Female **	400	Equal**	N/S	10

Systematic review source	Study source	Study design	Sampling method	Setting	Years of data collection	Population	Physical inactivity or sedentary behavior definition /study	Physical inactivity or sedentary behavior instrument or used items	Instrument administration	Age group (years)	Physical inactivity or sedentary behavior measurement	Gender	Sample size	♀/♂ (%)	Resp - rate (%)	QA score (/21)
							doing no structured exercise									
Libya (number of studies = 2)																
Yammine, 2016[6]	Guthold, 2010 $ [10]	Survey	Two-stage RS	National level	2007	Schoolchildren	% sitting and watching TV, computer games, talking with friends or another sitting activities ≥ 3 hrs /d	Validated PACE+ and Adolescent physical activity measure questionnaire	Self-administrated	13.9 (mean)	28.5**	Male and Female **	1354**	Equal	98	16
Yammine, 2016[6]	Guthold, 2010 $ [10]	GSHS**	Two-stage RS	National level	2007	Schoolchildren	% sitting and watching TV, computer games, talking with friends or another sitting activities ≥ 3 hrs /d	Validated PACE+ and Adolescent physical activity measure questionnaire	Self-administrated	13.9 (mean)	30	Male	681	50.3	N/S	13
Yammine, 2016[6]	Guthold, 2010 $ [10]	GSHS**	Two-stage RS	National level	2007	Schoolchildren	% sitting and watching TV, computer games, talking with friends or another sitting activities ≥ 3 hrs /d	Validated PACE+ and Adolescent physical activity measure questionnaire	Self-administrated	13.9 (mean)	27	Female	673	49.7	N/S	13
Sharara, 2018[9]	GSHS, 2007[100]	School-based survey	Two-stage cluster sample**	National level	2007	Students in grade 7, 8 and 9 **	< 60 mi per day on all seven days during the past seven days	PACE+	Self-reported**	10-19 **	83.4% **	Male and Female	2242 **	Equal	98	19

Systematic review source	Study source	Study design	Sampling method	Setting	Years of data collection	Population	Physical inactivity or sedentary behavior definition /study	Physical inactivity or sedentary behavior instrument or used items	Instrument administration	Age group (years)	Physical inactivity or sedentary behavior measurement	Gender	Sample size	♀/♂ (%)	Resp - rate (%)	QA score (/21)
Sharara, 2018[9]	GSHS, 2007[100]	School-based survey	Two-stage cluster sample**	National level	2007	Students in grade 7, 8 and 9 **	< 60 min per day on all seven days during the past seven days	PACE+	Self-reported**	10-19 **	78.5% **	Male	1123 **	50.1 **	N/S	16
Sharara, 2018[9]	GSHS, 2007[100]	School-based survey	Two-stage cluster sample**	National level	2007	Students in grade 7, 8 and 9**	< 60 min per day on all seven days during the past seven days	PACE+	Self-reported**	10-19 **	88.4% **	Female	1119 **	49.9 **	N/S	16
Djibouti (number of studies = 2)																
Yammine, 2016[6]	Guthold, 2010 $ [10]	GSHS**	Two-stage RS	National level	2007	Schoolchildren	% sitting and watching TV, computer games, talking with friends or another sitting activities ≥ 3 hrs /d	Validated PACE+ and Adolescent physical activity measure questionnaire	Self-administrated	14.4 (mean)	33**	Male and Female **	882	♀>♂	83	16
Yammine, 2016[6]	Guthold, 2010 $ [10]	GSHS**	Two-stage RS	National level	2007	Schoolchildren	% sitting and watching TV, computer games, talking with friends or another sitting activities ≥ 3 hrs /d	Validated PACE+ and Adolescent physical activity measure questionnaire	Self-administrated	14.4 (mean)	33	Male	526	59.6**	N/S	13
Yammine, 2016[6]	Guthold, 2010 $ [10]	GSHS**	Two-stage RS	National level	2007	Schoolchildren	% sitting and watching TV, computer games, talking with friends or another	Validated PACE+ and Adolescent physical activity measure	Self-administrated	14.4 (mean)	33	Female	356	40.4**	N/S	13

Systematic review source	Study source	Study design	Sampling method	Setting	Years of data collection	Population	Physical inactivity or sedentary behavior definition /study	Physical inactivity or sedentary behavior instrument or used items	Instrument admini-stration	Age group (years)	Physical inactivity or sedentary behavior measurement	Gender	Sample size	♀/♂ (%)	Resp - rate (%)	QA score (/21)
							sitting activities ≥ 3 hrs /d	questionnaire								
Sharara, 2018[9]	GSHS, 2007[101]	School-based survey	Two-stage cluster sample**	National level	2007	Students in class 5, 4, 3 and Other **	< 60 min per day on all seven days during the past seven days	PACE+	Self-reported**	13-15	85.1%	Male and Female**	1777	N/S	83**	19
Sharara, 2018[9]	GSHS, 2007[101]	School-based survey	Two-stage cluster sample**	National level	2007	Students in class 5, 4, 3 and Other **	< 60 min per day on all seven days during the past seven days	PACE+	Self-reported**	13-15	81.2%**	Male	707	39.8	N/S	16
Sharara, 2018[9]	GSHS, 2007[101]	School-based survey	Two-stage cluster sample**	National level	2007	Students in class 5, 4, 3 and Other **	< 60 min per day on all seven days during the past seven days	PACE+	Self-reported**	13-15	90.8%**	Female	1070	60.2	N/S	16
Jordan (number of studies = 3)																
Yammine, 2016[6]	Guthold, 2010 $ [10]	GSHS**	Two-stage RS	National level	2004	Schoolchildren	% sitting and watching TV, computer games, talking with friends or another sitting activities ≥ 3 hrs /d	Validated PACE+ and Adolescent physical activity measure questionnaire	Self-administrated	14.4 (mean)	41**	Male and Female **	1719	Equal	95	16
Yammine, 2016[6]	Guthold, 2010 $ [10]	GSHS**	Two-stage RS	National level	2004	Schoolchildren	% sitting and watching TV, computer games, talking with friends or another sitting	Validated PACE+ and Adolescent physical activity measure questionnaire	Self-administrated	14.4 (mean)	41	Male	829	48.2	N/S	13

Systematic review source	Study source	Study design	Sampling method	Setting	Years of data collection	Population	Physical inactivity or sedentary behavior definition /study	Physical inactivity or sedentary behavior instrument or used items	Instrument admini-stration	Age group (years)	Physical inactivity or sedentary behavior measurement	Gender	Sample size	♀/♂ (%)	Resp - rate (%)	QA score (/21)
							activities ≥ 3 hrs /d									
Yammine, 2016[6]	Guthold, 2010 $ [10]	GSHS**	Two-stage RS	National level	2004	Schoolchildren	% sitting and watching TV, computer games, talking with friends or another sitting activities ≥ 3 hrs /d	Validated PACE+ and Adolescent physical activity measure questionnaire	Self-administrated	14.4 (mean)	41	Female	890	51.8	N/S	13
Sharara, 2018[9]	GSHS, 2007[102]	School-based survey	Two-stage cluster sample**	National level	2007	Students in grade 8, 9 and 10**	< 60 min per day on all seven days during the past seven days	PACE+	Self-reported**	13-15	85.6%	Male and Female**	2197	Equal	99.8 **	19
Sharara, 2018[9]	GSHS, 2007[102]	School-based survey	Two-stage cluster sample**	National level	2007	Students in grade 8, 9 and 10**	< 60 min per day on all seven days during the past seven days	PACE+	Self-reported**	13-15	81.8%**	Male	1109 **	50.5 **	N/S	16
Sharara, 2018[9]	GSHS, 2007[102]	School-based survey	Two-stage cluster sample**	National level	2007	Students in grade 8, 9 and 10**	< 60 min per day on all seven days during the past seven days	PACE+	Self-reported**	13-15	89.0%**	Female	1088 **	49.5 **	N/S	16
Sharara, 2018[9]	Haddad, 2009 [103]	CS**	Multistage RSS**	Subnational level (school) **	N/S	Students from 7th and 8th grade**	Not very physically nor moderately active	Modified Adolescent Wellness Appraisal (AWA)	Self-report**	12-17	4%	Male and Female**	530	Equal**	N/S	10
Morocco (number of studies = 2)																
Yammine, 2016[6]	Guthold, 2010 $ [10]	GSHS**	Two-stage RS	National level	2006	Schoolchildren	% sitting and watching TV, computer games, talking with	Validated PACE+ and Adolescent physical activity	Self-administrated	14.1 (mean)	30**	Male and Female **	1735 **	♀>♂	84	16

Systematic review source	Study source	Study design	Sampling method	Setting	Years of data collection	Population	Physical inactivity or sedentary behavior definition /study	Physical inactivity or sedentary behavior instrument or used items	Instrument admini-stration	Age group (years)	Physical inactivity or sedentary behavior measurement	Gender	Sample size	♀/♂ (%)	Resp - rate (%)	QA score (/21)
							friends or another sitting activities ≥ 3 hrs /d	measure questionnaire								
Yammine, 2016[6]	Guthold, 2010 $ [10]	GSHS**	Two-stage RS	National level	2006	Schoolchildren	% sitting and watching TV, computer games, talking with friends or another sitting activities ≥ 3 hrs /d	Validated PACE+ and Adolescent physical activity measure questionnaire	Self-administrated	14.1 (mean)	29	Male	926	53.4	N/S	13
Yammine, 2016[6]	Guthold, 2010 $ [10]	GSHS**	Two-stage RS	National level	2006	Schoolchildren	% sitting and watching TV, computer games, talking with friends or another sitting activities ≥ 3 hrs /d	Validated PACE+ and Adolescent physical activity measure questionnaire	Self-administrated	14.1 (mean)	31	Female	809	46.6	N/S	13
Sharara, 2018[9]	GSHS, 2010[104]	School-based survey	Two stage cluster sample**	National level	2010	Students in Ist, 2nd and 3rd Prep**	< 60 min per day on five or more days during the past seven days	PACE+	Self-report on answer sheet**	13-15	82.6%	Male and Female**	2924	N/S	92**	19
Sharara, 2018[9]	GSHS, 2010[104]	School-based survey	Two stage cluster sample**	National level	2010	Students in Ist, 2nd and 3rd Prep**	< 60 min per day on five or more days during the past seven days	PACE+	Self-report on answer sheet**	13-15	79.2%**	Male	1620**	55.4**	N/S	16
Sharara, 2018[9]	GSHS, 2010[104]	School-based survey	Two stage cluster	National level	2010	Students in Ist, 2nd and 3rd Prep**	< 60 min per day on five or more days during the	PACE+	Self-report on answer sheet**	13-15	86.7%**	Female	1304**	44.6**	N/S	16

Systematic review source	Study source	Study design	Sampling method	Setting	Years of data collection	Population	Physical inactivity or sedentary behavior definition /study	Physical inactivity or sedentary behavior instrument or used items	Instrument admini-stration	Age group (years)	Physical inactivity or sedentary behavior measurement	Gender	Sample size	♀/♂ (%)	Resp - rate (%)	QA score (/21)
			sample**				past seven days									
Egypt (number of studies = 4)																
Yammine, 2016[6]	Guthold, 2010 $ [10]	GSHS**	Two-stage RS	National level	2006	Schoolchildren	% sitting and watching TV, computer games, talking with friends or another sitting activities ≥ 3 hrs /d	Validated PACE+ and Adolescent physical activity measure questionnaire	Self-administrated	13.5 (mean)	22.9**	Male and Female **	3664**	♀>♂	87	16
Yammine, 2016[6]	Guthold, 2010 $ [10]	GSHS**	Two-stage RS	National level	2006	Schoolchildren	% sitting and watching TV, computer games, talking with friends or another sitting activities ≥ 3 hrs /d	Validated PACE+ and Adolescent physical activity measure questionnaire	Self-administrated	13.5 (mean)	22	Male	1975	53.9	N/S	13
Yammine, 2016[6]	Guthold, 2010 $ [10]	GSHS**	Two-stage RS	National level	2006	Schoolchildren	% sitting and watching TV, computer games, talking with friends or another sitting activities ≥ 3 hrs /d	Validated PACE+ and Adolescent physical activity measure questionnaire	Self-administrated	13.5 (mean)	24	Female	1689	46.1	N/S	13
Sharara, 2018[9]	GSHS, 2006[105]	School-based survey	Two-stage cluster sample**	National level	2006	Students in Prep first, second and third grade **	< 60 min per day on all seven days during the past seven days	PACE+	Self-reported**	13-15	90.6%	Male and Female**	5249	N/S	87**	19

Systematic review source	Study source	Study design	Sampling method	Setting	Years of data collection	Population	Physical inactivity or sedentary behavior definition /study	Physical inactivity or sedentary behavior instrument or used items	Instrument administration	Age group (years)	Physical inactivity or sedentary behavior measurement	Gender	Sample size	♀/♂ (%)	Resp - rate (%)	QA score (/21)
Sharara, 2018[9]	GSHS, 2006[105]	School-based survey	Two-stage cluster sample**	National level	2006	Students in Prep first, second and third grade**	< 60 min per day on all seven days during the past seven days	PACE+	Self-reported**	13-15	85.5%**	Male	N/S	N/S	N/S	16
Sharara, 2018[9]	GSHS, 2006[105]	School-based survey	Two-stage cluster sample**	National level	2006	Students in Prep first, second and third grade **	< 60 min per day on all seven days during the past seven days	PACE+	Self-reported**	13-15	96.6%**	Female	N/S	N/S	N/S	16
Sharara, 2018[9]	Salazar-Martinez, 2006[106]	CS**	MCRS **	National level	1997	students were in junior high, high school or at the university **	Not engaged in sports	Questionnaire**	Self-reported**	11-19	62.3%	Male and Female**	1502	Equal**	N/S	13
Sharara, 2018[9]	Shady, 2015[107]	CS**	N/S	Subnational level (2 primary schools) **	N/S	Prepubescent students**	< 4 hours/week	Questionnaire**	Self-reported**	9-11	65.5%	Male and Female**	200	Equal**	N/S	8
Lebanon (number of studies = 2)																
Sharara, 2018[9]	GSHS, 2011[108]	School-based survey	Two-stage cluster sample**	National level	2011	Students in grade 7,8 and 9**	< 60 min per day on five or more days during the past seven days	PACE+	Self-reported**	13-15	65.4%	Male and Female**	2286	N/S	87**	19
Sharara, 2018[9]	GSHS, 2011[108]	School-based survey	Two-stage cluster sample**	National level	2011	Students in grade 7,8 and 9**	< 60 min per day on five or more days during the past seven days	PACE+	Self-reported**	13-15	57.6%**	Male	N/S	N/S	N/S	16
Sharara, 2018[9]	GSHS, 2011[108]	School-based survey	Two-stage cluster	National level	2011	Students in grade 7,8 and 9**	< 60 min per day on five or more days during the	PACE+	Self-reported**	13-15	72.3%**	Female	N/S	N/S	N/S	16

Systematic review source	Study source	Study design	Sampling method	Setting	Years of data collection	Population	Physical inactivity or sedentary behavior definition /study	Physical inactivity or sedentary behavior instrument or used items	Instrument administration	Age group (years)	Physical inactivity or sedentary behavior measurement	Gender	Sample size	♀/♂ (%)	Resp - rate (%)	QA score (/21)
			sample**				past seven days									
Sharara, 2018[9]	Nasreddine, 2014[109]	CS **	MCRS**	Subnational level (households)	2009	Children and adolescents above 6yrs old**	Moderate intensity activities included: playground activities, brisk walking, dancing, and bicycle riding. Higher intensity activities included: ball games, jumping rope, active games involving running and chasing, and swimming	Questionnaire**	Face to face interviews **	Mean: 13.06	32.6%	Male and Female**	868	Equal**	N/S	15
Iraq (number of studies = 1)																
Sharara, 2018[9]	GSHS, 2012[110]	School-based survey	Two-stage cluster sample**	National level	2012	Students in grade 1st, 2nd, and 3rd Intermediate**	< 60 min per day on five or more days during the past seven days	PACE+	Self-reported**	13-15	80%	Male and Female**	2038	N/S	88**	19
Sharara, 2018[9]	GSHS, 2012[110]	School-based survey	Two-stage cluster sample**	National level	2012	Students in grade 1st, 2nd, and 3rd Intermediate**	< 60 min per day on five or more days during the past seven days	PACE+	Self-reported**	13-15	74.7%**	Male	N/S	N/S	N/S	16
Sharara, 2018[9]	GSHS, 2012[110]	School-based survey	Two-stage cluster	National level	2012	Students in grade 1st, 2nd,	< 60 min per day on five or more days	PACE+	Self-reported**	13-15	86.4%**	Female	N/S	N/S	N/S	16

Systematic review source	Study source	Study design	Sampling method	Setting	Years of data collection	Population	Physical inactivity or sedentary behavior definition /study	Physical inactivity or sedentary behavior instrument or used items	Instrument admini-stration	Age group (years)	Physical inactivity or sedentary behavior measurement	Gender	Sample size	♀/♂ (%)	Resp - rate (%)	QA score (/21)
			sample**			and 3rd Intermediate**	during the past seven days									
Palestine (number of studies = 3)																
Sharara, 2018[9]	GSHS, 2010[111]	School-based survey	Two-stage cluster sample**	National level, Gaza strip**	2010	Students in grade 7th, 8th and 9th**	< 60 min per day on five or more days during the past seven days	PACE+	Self-reported**	13-15	75.8%	Male and Female**	2677	N/S	95**	19
Sharara, 2018[9]	GSHS, 2010[111]	School-based survey	Two-stage cluster sample**	National level, Gaza strip**	2010	Students in grade 7th, 8th and 9th**	< 60 min per day on five or more days during the past seven days	PACE+	Self-reported**	13-15	74.7%**	Male	N/S	N/S	N/S	16
Sharara, 2018[9]	GSHS, 2010[111]	School-based survey	Two-stage cluster sample**	National level, Gaza strip**	2010	Students in grade 7th, 8th and 9th**	< 60 min per day on five or more days during the past seven days	PACE+	Self-reported**	13-15	86.4%**	Female	N/S	N/S	N/S	16
Sharara, 2018[9]	GSHS, 2010[112]	School-based survey	Two-stage cluster sample**	National level, West Bank**	2010	Students in grade 7th, 8th and 9th**	< 60 min per day on five or more days during the past seven days	PACE+	Self-reported**	13-15	81.7%	Male and Female**	1908	N/S	94**	19
Sharara, 2018[9]	GSHS, 2010[112]	School-based survey	Two-stage cluster sample**	National level, West Bank**	2010	Students in grade 7th, 8th and 9th**	< 60 min per day on five or more days during the past seven days	PACE+	Self-reported**	13-15	77.2%**	Male	N/S	N/S	N/S	16
Sharara, 2018[9]	GSHS, 2010[112]	School-based survey	Two-stage cluster sample**	National level, West Bank**	2010	Students in grade 7th, 8th and 9th**	< 60 mins per day on five or more days during the past seven days	PACE+	Self-reported**	13-15	86.5%**	Female	N/S	N/S	N/S	16

Systematic review source	Study source	Study design	Sampling method	Setting	Years of data collection	Population	Physical inactivity or sedentary behavior definition /study	Physical inactivity or sedentary behavior instrument or used items	Instrument admini-stration	Age group (years)	Physical inactivity or sedentary behavior measurement	Gender	Sample size	♀/♂ (%)	Resp - rate (%)	QA score (/21)
Sharara, 2018[9]	Al Sabbah, 2007[113]	HSBC: CS	RSS**	National level (West Bank and Gaza Strip) **	2003-2004	Students of grades 6, 8, 10 and 12 **	< 60 mins/day, <5/7 days per week	WHO international HBSC questionnaire	Self-reported**	12-18	80.0%	Male and Female**	8885	N/S	N/S	16
Sharara, 2018[9]	Jildeh, 2011[114]	HBSC	Multistage RSS**	Subnational level (School) **	2002-2003	Students of grades 6, 8 and 10**	<5 days per week active in sport for at least one hour + mode of transport to and from school**	First Palestinian National Health and Nutrition Survey Questionnaire (2000)	Interviews**	11-16	77.6%	Male and Female**	314	Equal**	100	19
Qatar (number of studies = 1)																
Sharara, 2018[9]	GSHS, 2011[115]	School-based survey	Two-stage cluster sample**	National level	2011	Students in grade 7th- 9th**	< 60 min per day on five or more days during the past seven days	PACE+	Self-reported**	13-15	85.0%	Male and Female**	2021	N/S	87**	19
Sharara, 2018[9]	GSHS, 2011[115]	School-based survey	Two-stage cluster sample**	National level	2011	Students in grade 7th- 9th**	< 60 min per day on five or more days during the past seven days	PACE+	Self-reported**	13-15	80.1%**	Male	N/S	N/S	N/S	16
Sharara, 2018[9]	GSHS, 2011[115]	School-based survey	Two-stage cluster sample**	National level	2011	Students in grade 7th- 9th**	< 60 min per day on five or more days during the past seven days	PACE+	Self-reported**	13-15	89.8%**	Female	N/S	N/S	N/S	16
Sudan (number of studies = 2)																
Sharara, 2018[9]	GSHS, 2012[116]	School-based survey	Two-stage cluster sample**	National level	2012	Students in grade 8th basic, 1st & 2nd secondary	< 60 min per day on five or more days during the past seven days	PACE+	Self-reported**	13-15	89.0%	Male and Female	2211	N/S	77**	18
Sharara, 2018[9]	GSHS, 2012[116]	School-based survey	Two-stage cluster	National level	2012	Students in grade 8th basic,	< 60 min per day on five or more days	PACE+	Self-reported**	13-15	89.1%**	Male	N/S	N/S	N/S	15

Systematic review source	Study source	Study design	Sampling method	Setting	Years of data collection	Population	Physical inactivity or sedentary behavior definition /study	Physical inactivity or sedentary behavior instrument or used items	Instrument administration	Age group (years)	Physical inactivity or sedentary behavior measurement	Gender	Sample size	♀/♂ (%)	Resp - rate (%)	QA score (/21)
			sample**			1st & 2nd secondary	during the past seven days									
Sharara, 2018[9]	GSHS, 2012[116]	School-based survey	Two-stage cluster sample**	National level	2012	Students in grade 8th basic, 1st & 2nd secondary	< 60 min per day on five or more days during the past seven days	PACE+	Self-reported**	13-15	88.8%**	Female	N/S	N/S	N/S	15
Sharara, 2018[9]	Moukhyer, 2008[117]	CS**	Cluster RS**	Subnational level (household) **	2001	Adolescents	Not engaging in sports activities	Self-report Questionnaire	Interviews**	10-19	33.4%	Male and Female**	1200	Equal**	N/S	13
Syria (number of studies = 1)																
Sharara, 2018[9]	GSHS, 2010[118]	School-based survey	Two-stage cluster sample**	National level	2010	Students in grade 7, 8, 9 and 10**	< 60 min per day on five or more days during the past seven days	PACE+	Self-reported**	13-15	84.9%	Male and Female**	3102	N/S	97**	19
Sharara, 2018[9]	GSHS, 2010[118]	School-based survey	Two-stage cluster sample **	National level	2010	Students in grade 7, 8, 9 and 10**	< 60 min per day on five or more days during the past seven days	PACE+	Self-reported**	13-15	81.9%**	Male	N/S	N/S	N/S	16
Sharara, 2018[9]	GSHS, 2010[118]	School-based survey	Two-stage cluster sample **	National level	2010	Students in grade 7, 8, 9 and 10**	< 60 min per day on five or more days during the past seven days	PACE+	Self-reported**	13-15	89.2%**	Female	N/S	N/S	N/S	16
Yemen (number of studies = 1)																
Sharara, 2018[9]	GSHS, 2008[119]	School-based survey	Two-stage cluster sample**	National level	2008	Students in grade 7, 8 and 9 **	< 60 min per day on all seven days during the past seven days	PACE+	Self-reported**	13-15	84.8%	Male and Female**	1175	N/S	82**	19
Sharara, 2018[9]	GSHS, 2008[119]	School-based survey	Two-stage cluster	National level	2008	Students in grade 7, 8 and 9**	< 60 min per day on all seven days during the	PACE+	Self-reported**	13-15	83.2%**	Male	N/S	N/S	N/S	16

Systematic review source	Study source	Study design	Sampling method	Setting	Years of data collection	Population	Physical inactivity or sedentary behavior definition /study	Physical inactivity or sedentary behavior instrument or used items	Instrument admini-stration	Age group (years)	Physical inactivity or sedentary behavior measurement	Gender	Sample size	♀/♂ (%)	Res p - rate (%)	QA score (/21)
			sample**				past seven days									
Sharara, 2018[9]	GSHS, 2008[119]	School-based survey	Two-stage cluster sample**	National level	2008	Students in grade 7, 8 and 9 **	< 60 min per day on all seven days during the past seven days	PACE+	Self-reported**	13-15	87.7%**	Female	N/S	N/S	N/S	16
Tunisia (number of studies = 3)																
Sharara, 2018[9]	GSHS, 2008[120]	School-based survey	Two-stage cluster sample**	National level	2008	Students in grade 7, 8 and 9	< 60 min per day on all seven days during the past seven days	PACE+	Self-reported**	13-15	81.5%	Male and Female**	2870	N/S	83**	19
Sharara, 2018[9]	GSHS, 2008[120]	School-based survey**	Two-stage cluster sample **	National level	2008	Students in grade 7, 8 and 9**	< 60 min per day on all seven days during the past seven days	PACE+	Self-reported**	13-15	73.8%**	Male	N/S	N/S	N/S	16
Sharara, 2018[9]	GSHS, 2008[120]	School-based survey**	Two-stage cluster sample **	National level	2008	Students in grade 7, 8 and 9**	< 60 min per day on all seven days during the past seven days	PACE+	Self-reported**	13-15	89.0%**	Female	N/S	N/S	N/S	16
Sharara, 2018[9]	Nouira, 2014[121]	CS**	RS**	National level	2009-2010	schoolchildren in the 7th and 9th grades	Doesn't do recommended PA	Oxford Health Alliance for community intervention for health	Self-administered	12-14	88.1%	Male and Female	3987	Equal**	91.5 **	16
Sharara, 2018[9]	Aounallah-Skhiri, 2012[122]	CS**	Two-stage cluster sample **	National level	2005	Adolescents	< 3 Mets	Locally validated questionnaire	Self-administered	15-19	29.4%	Male and Female	2870	Equal**	91.5 **	18
Algeria (number of studies = 1)																
Sharara, 2018[9]	Abbes, 2016[123]	CS**	RS**	Subnational level	2010-2011	Students from level 1-5**	Not engaged in sports	Questionnaire**	Reported by parents**	6-11	92.8%	Male and Female	293	♀>♂	100 **	16

Systematic review source	Study source	Study design	Sampling method	Setting	Years of data collection	Population	Physical inactivity or sedentary behavior definition /study	Physical inactivity or sedentary behavior instrument or used items	Instrument admini-stration	Age group (years)	Physical inactivity or sedentary behavior measurement	Gender	Sample size	♀/♂ (%)	Resp - rate (%)	QA score (/21)
				(school) **												
Sharara, 2018[9]	Abbes, 2016[123]	CS**	RS**	Subnational level (school) **	2010-2011	Students from level 1-5**	>3hrs watching TV, video games, computers **	Questionnaire**	Reported by parents**	6-11	71.67% **	Male and Female **	293	♀>♂	100 **	18

Notes: All non-reported data was searched and extracted from the original study. Any additional information found relevant in the original study was added to the reported data for the purpose of completeness.
If not reported, the prevalence of the outcome among the total study population (males and/or females) was calculated using row or calculated data available in the original study. The calculated prevalence measure was reported and marked using two stars (**).
If not reported, the total sample size for each gender strata was calculated based on the percentage of males or females in the sample.
If not reported, the number for cases in each gender strata was calculated based on the reported prevalence and the total sample size in the strata.
If not reported, the total number for cases in the entire sample was calculated by the addition of the number of cases in each reported stratum.
For not reported mean time measures on the total study population, the overall males and females weighted mean time was calculated using mean time and the proportion of males and females in the sample, respectively.
If any discordance between the reported data in the SR and data available in the original study, this later was retained.
Extra studies are studies included in a SR and reporting one or more PA related outcome on MENA countries that were not reported in the SR (outcome or country not included in the SR). Extra data from these studies was extracted and used for the qualitative and quantitative synthesis.
Converted data from physical inactivity to PA was used only for the quantitative synthesis and was not reported in the above tables.
Abbreviations: SR: Systematic review; PA: Physical activity; VPA: Vigorous physical activity: MPA: Moderate physical activity; KSA: Kingdom of Saudi Arabia; UAE: United Arab Emirates; USA: United States of America; GCC: Gulf cooperation council; MENA: Middle-East and North Africa; QA: Quality assessment; ATLS: Arab teens lifestyle student questionnaire; GPAQ: Global Physical Activity Questionnaire; IPAQ: International Physical Activity Questionnaire: CS: Cross sectional: MCRS: Multistage stratified/cluster random sampling: RS: Random sampling: RCS: Random cluster sampling; RSS: Random stratified sampling; MET: Metabolic Equivalent of Task: VG: Video games; GSHS: Global School-based Student Health Survey; Resp-rate: response rate; N/S: Not stated; N/A: Not applicable; HBSC: Health Behavior in School-aged Children Survey; YRBSS: The Youth Risk Behavior Surveillance System, GSHS: Global School-based Student Health survey
† The use of a validated questionnaire was not reported in the original study.
‡ The use of Nurses' Health Study II questionnaire was not reported in the original study.
*Calculated from reported data in the SR
**Calculated or reported from data in the original study
***Calculated from the prevalence measure reported in the original study (different from the prevalence measure reported in the SR)
$ This study was included in the SR of Yammine, 2016 [6]. Sedentary behavior data extracted from this study of Guthold, 2010 [10] was not reported by the SR of Yammine, 2016 [6].
♂: Males; ♀: Females; km: Kilometer; min: Minutes; wk: Week; d: Day: d-1: Per day; hrs: hours; h: hour
MJ: The megajoule is equal to one million (106) joules; Energy expenditure of >0.3 MJ/day is equivalent to walking daily for half an hour at 5 km/h

Table S12. Summary of physical activity/ inactivity, and sedentary behavior definitions along with validation data of the used measurement tools.

Outcome definition	Measurement tool used for the outcome measurement	Main objective of the measurement tool (what it is developed for)	Country where used [Study reference]	Systematic review source	Official primary language of the country [182]	Validated in the official primary language of the country [¥]	Validated among adults[¥]	Validated among youth[¥]
Physical activity in adult general population								
Active ≥600 METmin/wk	PA questionnaire**	Developed to assess leisure-type and sport-related physical activity. Measures type, duration, frequency, and habits of physical activity. [12]	Saudi Arabia [12]	Sisson, 2008 [3]	Arabic	No evidence	No evidence	No evidence
At least 600 MET-min/wk of vigorous or moderate activity	GPAQ	Developed to enable countries to surveil and collect information on the level of participation in physical activity in three settings. [160]	Saudi Arabia [25]	Mabry, 2010 [4]	Arabic	Yes [183]	Yes [160]	No evidence
			UAE [168]	Mabry, 2010 [4]	Arabic			
Active 600 or more MET-min/wk	Measurement tool included items regarding sports and leisure activities	Developed to assess leisure-type and sport-related physical activity. Measures type, duration, frequency, and habits of physical activity. [12]	Saudi Arabia [12]	Mabry, 2010 [4]	Arabic	No evidence	No evidence	No evidence
At least 600 MET-min/wk of vigorous or moderate activity	IPAQ	Developed and tested for the population surveillance of adults (age range 15-69 years) and their level of physical activity. Physical activity levels are assessed across multiple domains. [184]	Saudi Arabia [81]	Mabry, 2010 [4]	Arabic	Yes [185]	Yes [184]	No evidence
			Kuwait [171]	Mabry, 2010 [4]	Arabic			
150 min of moderate-intensity PA/wk	IPAQ short version	Developed and tested for the population surveillance of adults (age range 15-69 years) and their level of physical activity. Physical activity levels are assessed across multiple domains. [184]	Saudi Arabia [81] [71] [12]	Mabry, 2016 [7]	Arabic	Yes [185]	Yes [184]	No evidence
150 min of moderate-intensity PA/wk	Validated questionnaire on leisure time PA and walking†	Developed to assess leisure-type and sport-related physical activity. Measures type, duration, frequency, and habits of physical activity. [12]	Saudi Arabia [12]	Mabry, 2016 [7]	Arabic	No evidence	No evidence	No evidence
150 min of moderate-intensity PA/wk	GPAQ	Developed to enable countries to surveil and collect information on the level of participation in physical activity in three settings. [160]	Saudi Arabia [166] [167]	Mabry, 2016 [7]	Arabic	Yes [183]	Yes [160]	No evidence
			Oman [170]	Mabry, 2016 [7]	Arabic			
			Qatar [140]	Mabry, 2016 [7]	Arabic			
150 min of moderate-intensity PA/wk	ATLS	Developed to assess the lifestyle habits of Arab adolescents and to study the interrelationships between various lifestyle variables. Investigates the prevalence of physical and sedentary activity, obesity and overweight, and dietary habits among youth. [150,186]	Saudi Arabia [67]	Mabry, 2016 [7]	Arabic	Yes [150,186]	No evidence	Yes [150,186]
Vigorous level of PA - (Severe participation in physical exercises**)	Personal PA questionnaire	Developed to measure the prevalence of obesity in female university students within the United Arab Emirates. Collects data on age, obesity in	UAE [13]	Yammine, 2016 [6]	Arabic	No evidence	No evidence	No evidence

		childhood and among parents, snacking, fast food consumption, and physical activity. [13]						
150 min of moderate-intensity PA/wk	Nurses' Health Study II ‡	Developed to ascertain the determinants of breast cancer and other major illnesses in young women (age range 25-42 years). Includes a physical activity questionnaire, which reports level of recreational physical activity participation weekly. [187]	UAE [169]	Mabry, 2016 [7]	Arabic	No evidence	Yes [187]	No evidence
150 min of moderate-intensity PA/wk	GPAQ	Developed to enable countries to surveil and collect information on the level of participation in physical activity in three settings. [160]	Oman [170]	Mabry, 2016 [7]	Arabic	Yes [183]	Yes [160]	No evidence
Active: energy expenditure >0.3 MJ d-1 (equivalent to walking daily for 30 min at 5km/h**)	Measurement tool contained questions on walking, cycling and recreational activities based on which energy expenditure was calculated	Developed to assess the high prevalence of diabetes in Bahrainis through collecting data on a participant's demographics, medical history, and physical activity. [139]	Bahrain [139]	Mabry, 2010 [4]	Arabic	No evidence	No evidence	No evidence
Active: walk ≥1 km d-1	Measurement tool contained WHO Heart and Health Questionnaire (validated in Arabic**)	Developed assess the impact of physical activity and education level on obesity among adult Bahrainis by collecting data on a participant's demographics, medical history, and physical activity. [138]	Bahrain [138]	Mabry, 2010 [4]; Sharara, 2018[9]	Arabic	Unclear [188]§	Unclear [188]§	Unclear [188]§
Active: walked or cycled for at least 30 min d-1	Measurement tool contained a question if person walked or cycled at least 30 min d-1	Developed to ascertain the relationship between hypertension and cardiovascular risk factors for Qatari adults (age range 25-65 years). Collects data on: basic demographics, physical activity, lifestyle and eating habits, personal and family medical history, and BMI. [137]	Qatar [137]	Mabry, 2010 [4]; Sharara, 2018[9]	Arabic	No evidence	No evidence	No evidence
Physical activity in youth general population								
Active ≥13,000 steps/d	Pedometer	Developed to ascertain the difference between habitual physical activity (measured by a pedometer) between obese and non-obese Saudi schoolboys (age range 8-12 years). [128]	Saudi Arabia [128]	Sisson, 2008 [3]	Arabic	N/A	Yes* [154,155]	Yes* [152-155]
60 min of moderate-intensity 7 d/wk	ATLS	Developed to assess the lifestyle habits of Arab adolescents and to study the interrelationships between various lifestyle variables. Investigates the prevalence of physical and sedentary activity, obesity and overweight, and dietary habits. [186]	Saudi Arabia [143] [150] [144] [145] [146] [147]	Mabry, 2016 [7]	Arabic	Yes [150,186]	No evidence	Yes [150,186]
			Oman [173]	Mabry, 2016 [7]	Arabic			

			Kuwait[174]	Mabry, 2016[7]	Arabic			
Moderate level of physical activity – (At least 60 min of PA per day on at least 5 days per week**)	Adolescent physical activity measure questionnaire (PACE+**)	Developed to assess health habits of children in school (age range 13-15 years). Instrument investigates the number of days during the past 7 days and during a normal week a child participates in physical activity for at least 60 minutes. [10]	UAE[10]	Yammine, 2016[6]	Arabic	Yes[189]	No evidence	Yes[189]
Moderate level of PA- Moderate score of PA**	IPAQ short version	Developed and tested for the population surveillance of adults (age range 15-69 years) and their level of physical activity. Physical activity levels are assessed across multiple domains. [184]	UAE[14] [15]	Yammine, 2016[6]	Arabic	Yes[185]	Yes[184]	No evidence
Moderate level of PA - (Vigorous exercise < 3 times/wk for about 60 min and > 30 min of moderate PA most days of the week**)	Personal physical activity questionnaire	Developed to assess the knowledge level about sport practice amongst secondary-school students in Dubai. Additionally, it assesses their attitudes and level participation in sports. [16]	UAE[16]	Yammine, 2016[6]	Arabic	No evidence	No evidence	No evidence
Vigorous level of PA – (High score of PA**)	IPAQ short version	Developed and tested for the population surveillance of adults (age range 15-69 years) and their level of physical activity. Physical activity levels are assessed across multiple domains. [184]	UAE[14] [15]	Yammine, 2016[6]	Arabic	Yes[185]	Yes[184]	No evidence
Vigorous level of PA- (Vigorous exercise ≥ 3 times/wk for about 20 min/session and also > 30 min of moderate PA most days of the week **)	Personal physical activity questionnaire	Developed to assess the knowledge level about sport practice amongst secondary-school students in Dubai. Additionally, it assesses their attitudes and level participation in sports. [16]	UAE[16]	Yammine, 2016[6]	Arabic	No evidence	No evidence	No evidence
Physical inactivity in adult general population								
Inactive: <600 METmin/wk	PA questionnaire**	Developed to assess leisure-type and sport-related physical activity. Measures type, duration, frequency, and habits of physical activity. [12]	Saudi Arabia[12]	Sisson, 2008[3]<br>Sharara, 2018[9]<br>Al-Hazzaa, 2018[8]	Arabic	No evidence	No evidence	No evidence
Inactive: <600 METmin/wk	GPAQ	Developed to enable countries to surveil and collect information on the level of participation in physical activity in three settings. [160]	Saudi Arabia[77] [24 25,77,82,83]	Sharara, 2018[9]<br>Al-Hazzaa, 2018[8]	Arabic	Yes[183]	Yes[160]	No evidence
			Kuwait[34]		Arabic			
			Qatar[141]		Arabic			
			Jordan[41]		Arabic			
			Egypt[43]		Arabic			
			Iraq[47]		Arabic			
			Libya[48]		Arabic			
			Lebanon[49]		Arabic			
			Palestine[55]		Arabic			
			Algeria[58]		Arabic			

			Sudan[59]		Arabic			
<600 METmin/wk	Modified ATLS	Developed to assess the lifestyle habits of Arab adolescents and to study the interrelationships between various lifestyle variables. Investigates the prevalence of physical and sedentary activity, obesity and overweight, and dietary habits. [186]	Saudi Arabia [70,85]	Al-Hazzaa, 2018[8]	Arabic	Yes	Yes [190,191]	Yes [150,186]
<600 METmin/wk	IPAQ	Developed and tested for the population surveillance of adults (age range 15-69 years) and their level of physical activity. Physical activity levels are assessed across multiple domains. [184]	Saudi Arabia [68,81]	Al-Hazzaa, 2018[8] Sharara, 2018[9]	Arabic	Yes [185]	Yes [184]	No evidence
<840 MET-min/week	Questionnaire	To assess the PA patterns, sedentary activities (e.g., daily TV/computer/DVD viewing time) and dietary habits.	Qatar[35]	Sharara, 2018[9]	Arabic	No evidence	Yes[191]	No evidence
Inactivity: < 3 days of VPA at 20 min/session or <5 days MPA at 30 min/session	IPAQ short version **	Developed and tested for the population surveillance of adults (age range 15-69 years) and their level of physical activity. Physical activity levels are assessed across multiple domains. [184]	Pakistan [11]	Sisson, 2008 [3]	Urdu	Yes [185]	Yes [184]	No evidence
			Tunisia [11]	Sisson, 2008 [3] Sharara, 2018[9]	Arabic	Yes [185]	Yes [184]	No evidence
			UAE [11]	Sisson, 2008 [3] Sharara, 2018[9]	Arabic	Yes [185]	Yes [184]	No evidence
Inactivity: <30 minutes of moderate to vigorous activity most of the days (at least 4 days in last 7 days**	IPAQ	Developed and tested for the population surveillance of adults (age range 15-69 years) and their level of physical activity. Physical activity levels are assessed across multiple domains. [184]	Pakistan [17]	Ranasinghe, 2013 [5]	Urdu	Yes [185]	Yes [184]	No evidence
<30 minutes /≥ 5 days/week Or <150 mins/week of moderate level of physical activity	GPAQ	Developed to enable countries to surveil and collect information on the level of participation in physical activity in three settings. [160]	Saudi Arabia[23] [79]	Sharara, 2018[9] Al-Hazzaa, 2018[8]	Arabic	Yes [183]	Yes [160]	No evidence
	IPAQ	Developed and tested for the population surveillance of adults (age range 15-69 years) and their level of physical activity. Physical activity levels are assessed across multiple domains. [184]	Saudi Arabia [71,76,84]			Yes [185]	Yes [184]	No evidence
	Oxford Health Alliance Community Intervention for Health Project	To assess association between tobacco use and other lifestyle risk factors for chronic disease	Tunisia[26]			No evidence	No evidence	No evidence
	ATLS	Developed to assess the lifestyle habits of Arab adolescents and to study the interrelationships between various lifestyle variables. Investigates the prevalence of physical and sedentary	Saudi Arabia [67]			Yes	Yes [190,191]	Yes [150,186]

		activity, obesity and overweight, and dietary habits among youth. [150,186]						
	Questionnaire	To assess physical activity among diabetics and healthy population	Saudi Arabia [87]			No evidence	No evidence	No evidence
	"How physically active are you" questionnaire	To assess healthy lifestyle among primary health care professionals.	Saudi Arabia [74]			Yes	Yes[192]	No evidence
< 30 mins of regular, moderate or intense physical activity on most days	Questionnaire	Developed to assess association of obesity with demographics including physical activity. Usual physical activity in work, method of going to and from work, leisure time and sports practice were considered in the questionnaire.	Morocco[60]	Sharara, 2018[9]	Arabic	No evidence	No evidence	No evidence
<30 mins of physical activity 3 or more times per week	Questionnaire	To investigate physical activity levels across the five stages of change for physical activity and to identify motivational factors for physical activity according to these stages of change	Jordan[40]	Sharara, 2018[9]	Arabic	No evidence	No evidence	No evidence
<30 mins of physical exercise	Questionnaire	To assess association of cardiovascular disease risk factors with Hypertension	Lebanon[54]	Sharara, 2018[9]	Arabic	No evidence	No evidence	No evidence
Physical exercise for <0.5 h/week	Questionnaire	To assess determinants of physical exercise	Lebanon[51]	Sharara, 2018[9]	Arabic	No evidence	No evidence	No evidence
Neither moderate nor vigorous physical activity	GPAQ	Developed to enable countries to surveil and collect information on the level of participation in physical activity in three settings. [160]	Kuwait[32]	Sharara, 2018[9]	Arabic	Yes [183]	Yes [160]	No evidence
Neither moderate nor vigorous physical activity	IPAQ	Developed and tested for the population surveillance of adults (age range 15-69 years) and their level of physical activity. Physical activity levels are assessed across multiple domains. [184]	Saudi Arabia[68] [19,86]	Sharara, 2018[9] Al-Hazzaa, 2018[8]	Arabic	Yes [185]	Yes [184]	No evidence
Neither moderately nor very active	Questionnaire	Physical activity was assessed in relation to prevalence of metabolic syndromes. Activity levels of subjects were self-reported as sedentary, lightly active, moderately active, or very active	Kuwait[31]	Sharara, 2018[9]	Arabic	No evidence	No evidence	No evidence
Neither moderate-intensity physical activity for at least 150 min per week nor vigorous-intensity	Questionnaire	Developed to assess physical activity in terms of duration and frequency and its association with stroke symptoms among stroke free residents	Lebanon[53]	Sharara, 2018[9]	Arabic	No evidence	No evidence	No evidence

physical activity for 75 min at least per week								
Not practicing in any regular sport and leisure time physical Activity	CDC web site questionnaire	To investigate barriers to healthy lifestyle. [193]	Saudi Arabia [21]	Sharara, 2018[9] Al-Hazzaa, 2018[8]	Arabic	No evidence	Yes [193]	No evidence
Not engaging in moderate activity (resulting in light sweating, small increases in breathing or heart rate)	Questionnaire	Developed to monitor the behavioral risk factors associated with chronic diseases like obesity and diabetes.	Jordan[37,38]	Sharara, 2018[9]	Arabic	No evidence	No evidence	No evidence
No strenuous exercise for ≥ 3 times per week	Lipid Research Clinic Questionnaire	To assess association between strenuous physical activity and obesity.	Saudi Arabia[78]	Al-Hazzaa, 2018[8]	Arabic	No evidence	Yes [194]	No evidence
No non-vigorous physical activity for at least 20 minutes or 3 times per week)<br>No exercise**	Questionnaire	Physical activity behaviour and sedentary lifestyle in terms of Frequency and type of current physical activity, Frequency of participation in a sports team, Students' satisfaction with the performed physical activity, Average daily hours of watching television	Egypt[44]	Sharara, 2018[9]	Arabic	No evidence	No evidence	No evidence
No exercise	Questionnaire	Developed to investigate lifestyle risk factors and colorectal cancer	Saudi Arabia [20]	Sharara, 2018[9]	Arabic	No evidence	No evidence	No evidence
		To assess association of regular exercise with fatigue	UAE[28]					
		To assess cardiovascular risk factors. Frequency of exercise was noted	Jordan[39]					

		To ascertain role of lifestyle factors in the development of colorectal cancer. Performing regular exercise, type and the frequency per week was considered	Egypt[46]					
		Physical activity assessed to measure health behaviour	Palestine[57]					
		To investigate lifestyle patterns in smokers and non-smokers	Bahrain[62]					
No regular activity	Questionnaire	To assess physical activity profiles. Frequency and duration of the activity were accounted	Saudi Arabia [64] [72] [80]	Al-Hazzaa, 2018[8]	Arabic	No evidence	No evidence	No evidence
Not engaging in regular physical activity	Questionnaire	To assess health related risk factors associated with physical inactivity	Kuwait[30,33] Saudi Arabia[18] [21,73] Egypt[45]	Sharara, 2018[9] Al-Hazzaa, 2018[8]	Arabic	No evidence	No evidence	No evidence
Less than one-hour weekly activity	Questionnaire	To assess physical health status	Saudi Arabia[22] UAE[27,29]	Sharara, 2018[9]	Arabic	No evidence	No evidence	No evidence
< I time per week	Questionnaire	To assess association of diet and physical activity with BMI	Saudi Arabia[69]	Hazzaa, 2018[8]	Arabic	No evidence	No evidence	No evidence
<3 hours/week	Questionnaire	To assess health risk behavior	Lebanon[52]	Sharara, 2018[9]	Arabic	No evidence	No evidence	No evidence
<3 times/week Or <3 days/week	Questionnaire	To assess exercise practices and stages of change in physical activity	Saudi Arabia[65,66,142]	Hazzaa, 2018[8]	Arabic	No evidence	No evidence	No evidence
Less than 15 mins/ week of sport or brisk walking	Questionnaire	Developed to assess lifestyle related risk factors.	Syria[63]	Sharara, 2018[9]	Arabic	No evidence	No evidence	No evidence
<150 MET-min/ week	IPAQ	Developed and tested for the population surveillance of adults (age range 15-69 years) and their level of physical activity. Physical activity levels are assessed across multiple domains. [184]	Saudi Arabia[84]	Hazzaa, 2018[8]	Arabic	Yes [185]	Yes [184]	No evidence

Nil practice of PA – (Never participated in physical exercises**)	Personal physical activity questionnaire	Developed to measure the prevalence of obesity in female university students within the United Arab Emirates. Collects data on age, obesity in childhood and among parents, snacking, fast food consumption, and physical activity. [13]	UAE [13]	Yammine, 2016 [6]	Arabic	No evidence	No evidence	No evidence
Mild level of PA – (Mild participation in physical exercises**)	Personal physical activity questionnaire	Developed to measure the prevalence of obesity in female university students within the United Arab Emirates. Collects data on age, obesity in childhood and among parents, snacking, fast food consumption, and physical activity. [13]	UAE [13]	Yammine, 2016 [6]	Arabic	No evidence	No evidence	No evidence
Sedentary behavior in adult general population								
Mean amount of time spent sitting (Mean total time spent in sedentary activities (min/d) **)	GPAQ	Developed to enable countries to surveil and collect information on the level of participation in physical activity in three settings. [160]	Saudi Arabia [25]	Mabry, 2010 [4]	Arabic	Yes [183]	Yes [160]	No evidence
			Qatar [140]	Mabry, 2016 [7]	Arabic			
Mean amount of time spent sitting	IPAQ	Developed and tested for the population surveillance of adults (age range 15-69 years) and their level of physical activity. Physical activity levels are assessed across multiple domains. [184]	Kuwait [171]	Mabry, 2010 [4]	Arabic	Yes [185]	Yes [184]	No evidence
Sedentary (6+ hrs/d)	GPAQ	Developed to enable countries to surveil and collect information on the level of participation in physical activity in three settings. [160]	Oman [170]	Mabry, 2016 [7]	Arabic	Yes [183]	Yes [160]	No evidence
Sedentary (3+ hrs/d)	GPAQ	Developed to enable countries to surveil and collect information on the level of participation in physical activity in three settings. [160]	Oman [176]	Mabry, 2016 [7]	Arabic	Yes [183]	Yes [160]	No evidence
Physical inactivity in youth general population								
Nil practice of PA- (Did not practice PA at all**)	Personal PA questionnaire	Developed to assess the knowledge level about sport practice amongst secondary-school students in Dubai. Additionally, it assesses their attitudes and level participation in sports. [16]	UAE [16]	Yammine, 2016 [6]	Arabic	No evidence	No evidence	No evidence
Doesn't do recommended PA	Oxford Health Alliance for community intervention for health	To determine the association between tobacco use and other lifestyle factors among schoolchildren.	Tunisia[121]	Sharara, 2018[9]	Arabic	No evidence	No evidence	No evidence
Engaging in physical activities <once per week, apart from school physical education	Health practices scale	To assess 5 healthy practices: Sleep, breakfast, not eating between meals, not smoking and doing PA.	Oman[96]	Sharara, 2018[9]	Arabic	No evidence	No evidence	No evidence

Complete absence of exercise	Modified YRBSS and PACE+	To identify the health risk behaviors and health status of adolescents.	Saudi Arabia [88]	Sharara, 2018[9]	Arabic	No evidence	No evidence	No evidence
No regular exercise	Questionnaire	Apart from knowledge about T2DM, the questionnaire assessed the perceived benefits of and barriers to healthy lifestyle behaviours.	Saudi Arabia[90]	Sharara, 2018[9]	Arabic	No evidence	No evidence	No evidence
Moderate intensity activities included: playground activities, brisk walking, dancing, and bicycle riding. Higher intensity activities included: ball games, jumping rope, active games involving running and chasing, and swimming	Questionnaire	To ascertain dietary, lifestyle and socioeconomic correlates of overweight, obesity and central adiposity. It assessed weekly frequency of physical activity outside school setting	Lebanon[109]	Sharara, 2018[9]	Arabic	No evidence	No evidence	No evidence
< 3 Mets	Questionnaire	To determine associations between socio-demographic, lifestyle factors and anthropometric status and blood pressure status. Physical activity practised during the preceding month recorded using validated frequency questionnaire [195]	Tunisia[122]	Sharara, 2018[9]	Arabic	Yes[195]	No evidence	No evidence
Mild level of PA – (Low score of PA**)	IPAQ short version	Developed and tested for the population surveillance of adults (age range 15-69 years) and their level of physical activity. Physical activity levels are assessed across multiple domains. [184]	UAE [14] [15]	Yammine, 2016 [6] Hazzaa, 2018[8]	Arabic	Yes [185]	Yes [184]	No evidence
<420 MET-min/week			Saudi Arabia [84]		Arabic			
Mild level of PA- (No vigorous activity or irregularly practiced vigorous exercise < 60 min/wk and < 30 min of moderate PA most days of the week**)	Personal PA questionnaire	Developed to assess the knowledge level about sport practice amongst secondary-school students in Dubai. Additionally, it assesses their attitudes and level participation in sports. [16]	UAE [16]	Yammine, 2016 [6]	Arabic	No evidence	No evidence	No evidence
Only performing normal daily routine with some recreational activities or walking slowly and doing no structured exercise	Questionnaire	To investigate factors associated with low backache. The questionnaire included social factors, e.g., number of hours per week spent on recreational activities, ways of spending spare time, and level of physical activity.	Kuwait[99]	Sharara, 2018[9]	Arabic	No evidence	No evidence	No evidence
Occupation-related sedentary light PA for	Questionnaire	To investigate PA pattern apart from food consumption and smoking	Palestine[56]	Sharara, 2018[9]	Arabic	No evidence	No evidence	No evidence

men and no exercise for women								
Neither very physically nor moderately active	Modified Adolescent Wellness Appraisal (AWA)	To assess basic health knowledge and behaviors that most influence the health of adolescents.	Jordan[103]	Sharara, 2018[9]	Arabic	No evidence	No evidence	No evidence
Less than 30 mins of physical exercise during the previous week	CDC Adolescent Health Survey Questionnaire	Developed to collect information on socio-demographic factors, dietary habits, level of physical activity participation, and related behaviors. [92]	Saudi Arabia [92] [132]	Sharara, 2018[9] Hazzaa, 2018[8]	Arabic	Unclear [196]§	Unclear [196]§	Unclear [196]§
<30 min for <4 days/ week	Questionnaire	To determine correlates of overweight and obesity among adolescents.	Al-Rukban, 2003[130]	Hazzaa, 2018[8]	Arabic	No evidence	No evidence	No evidence
Not engaging in sports activities	Questionnaire	To collect sociodemographic data and information on child's diet and exercise behavior and investigate its association with overweight/obesity.	Saudi Arabia[89] Egypt[106] Sudan[117] Algeria[123]	Sharara, 2018[9]	Arabic	No evidence	No evidence	No evidence
< 60 mins per day on all seven days during the past seven days	PACE+	Developed to assess health habits of children in school (age range 13-15 years). Instrument investigates the number of days during the past 7 days and during a normal week a child participates in physical activity for at least 60 minutes. [10]	Tunisia[120], Yemen[120], Syria[118], Sudan[116], Qatar[115], Palestine[111] [112], Iraq[110], Lebanon[108], Egypt[105], Morocco[104], Jordan[102], Djibouti[101], Libya[100] Kuwait[98], Oman[95] UAE[94]	Sharara, 2018[9]	Arabic	Yes [189]	No evidence	Yes [189]
< 60 mins/day, <5/7 days per week	WHO international HBSC questionnaire	The international standard questionnaire enables the collection of common data across participating countries and thus enables the quantification of patterns of key health behaviours, health indicators and contextual variables. [197].	Palestine[113] Saudi Arabia[133]	Sharara, 2018[9] Hazzaa, 2018[8]	Arabic	No evidence	No evidence	Yes [198]
<5 days per week active in sport for at least one hour + mode of transport to and from school**			Palestine [114]		Arabic			
MVPA for 60 min/ day** <5 days a week	Questionnaire	To assess PA behavior predictors, reasons and barriers.	Saudi Arabia[134]	Hazzaa, 2018[8]	Arabic	No evidence	No evidence	No
Sum of moderate to vigorous activity score during the past 7 days**	PAQ-A	To assess physical activity among female adolescents.	Saudi Arabia[135]	Hazzaa, 2018[8]	Arabic	No evidence	No evidence	No evidence

Based on weekly frequency: Never	Questionnaire	The questionnaire sought information about sun exposure (frequency and duration of exposure) and physical activity, which is self reported in terms of frequency and type of activities performed along with duration – number of minutes per week.	Saudi Arabia [91]	Sharara, 2018[9]	Arabic	No evidence	No evidence	No evidence
< 4 hours/week			Egypt[107]					
<5 days/week of playing sport	Questionnaire	To investigate social, dietary and lifestyle factors associated with obesity.	Bahrain[97]	Sharara, 2018[9]	Arabic	No evidence	No evidence	Yes [199]
<10000 step counts/ day	Accelerometer	To assess physical activity among girls aged 8-11 years.	Saudi Arabia [129]	Hazzaa, 2018[8]	Arabic	No evidence	No evidence	Yes [200]
<13,000 step counts or <10,000 step counts/ day	Electronic pedometer	Developed to ascertain the difference between habitual physical activity (measured by a pedometer) between obese and non-obese Saudi schoolboys (age range 8-12 years). [128]	Saudi Arabia [125,128]	Hazzaa, 2018[8]	Arabic	No evidence	Yes* [154,155]	Yes* [152-155]
Daily heart rate <159 bpm for atleast 20 min/day or<br>Daily heart rate <140 bpm for at least 30min/day	Continuous heart rate monitoring[201]	Physical activity level was obtained from heart rate telemetry.	Saudi Arabia [126,127]	Hazzaa, 2018[8]	Arabic	No evidence	Yes [201]	Yes [201]
<1680 METs-min/week	ATLS	Developed to assess the lifestyle habits of Arab adolescents and to study the interrelationships between various lifestyle variables. Investigates the prevalence of physical and sedentary activity, obesity and overweight, and dietary habits. [186]	Saudi Arabia [124,145147147147171] [168 167]	Sharara, 2018[9]<br>Hazzaa, 2018[8]	Arabic	Yes [150,186]	No evidence	Yes [150,186]
Any bodily movement produced by skeletal muscles that resulted in energy expenditure above the basal level for at least 20 minutes per session** <3 times/ week	Questionnaire	To assess knowledge and pattern of physical activity among school students.	Saudi Arabia[131]	Hazzaa, 2018[8]	Arabic	No evidence	No evidence	No evidence
Sedentary behavior in youth general population								
Computer/TV time >2 hrs/d	ATLS	Developed to assess the lifestyle habits of Arab adolescents and to study the interrelationships between various lifestyle variables. Investigates the prevalence of physical and sedentary activity, obesity and overweight, and dietary habits. [186]	Saudi Arabia [124] [144] [145]	Mabry, 2016 [7]	Arabic	Yes [150,186]	No evidence	Yes [150,186]
Mean computer/TV time (hrs/d	ATLS	Developed to assess the lifestyle habits of Arab adolescents and to study the	Saudi Arabia [145]	Mabry, 2016 [7]	Arabic	Yes [150,186]	No evidence	Yes [150,186]

		interrelationships between various lifestyle variables. Investigates the prevalence of physical and sedentary activity, obesity and overweight, and dietary habits. [186]						
Mean computer/TV time (min/d)	Questionnaire to parents**	Developed to assess the levels of adiposity and physical activity in Saudi preschool children in Jeddah. Habitual physical activity was measure by electronic pedometers. [125]	Saudi Arabia [125]	Mabry, 2016 [7]	Arabic	No evidence	No evidence	No evidence
Mean time watching TV (hrs/d)	ATLS	Developed to assess the lifestyle habits of Arab adolescents and to study the interrelationships between various lifestyle variables. Investigates the prevalence of physical and sedentary activity, obesity and overweight, and dietary habits. [186]	Saudi Arabia [146] [147]	Mabry, 2016 [7]	Arabic	Yes [150,186]	No evidence	Yes [150,186]
Mean computer time (hrs/d)	ATLS	Developed to assess the lifestyle habits of Arab adolescents and to study the interrelationships between various lifestyle variables. Investigates the prevalence of physical and sedentary activity, obesity and overweight, and dietary habits. [186]	Saudi Arabia [146] [147]	Mabry, 2016 [7]	Arabic	Yes [150,186]	No evidence	Yes [150,186]
Mean TV time (hrs/d)	Designed questionnaire**	Developed to collect data on the lifestyle and behavioral activities of study participants. Data collected on demographics, dietary habits, and health-related lifestyle variables (i.e. smoking, physical activity participation, TV watching, video-game playing etc.). [177]	Saudi Arabia [177]	Mabry, 2016 [7]	Arabic	No evidence	No evidence	No evidence
Mean computer game time (hrs/d)	Designed questionnaire**	Developed to collect data on the lifestyle and behavioral activities of study participants. Data collected on demographics, dietary habits, and health-related lifestyle variables (i.e. smoking, physical activity participation, TV watching, video-game playing etc.). [177]	Saudi Arabia [177]	Mabry, 2016 [7]	Arabic	No evidence	No evidence	No evidence
Watched > 3hrs TV/d	CDC Adolescent Health adapted questionnaire (Validated Arabic version**)	Developed to collect information on socio-demographic factors, dietary habits, level of physical activity participation, and related behaviors. [92]	Saudi Arabia [92] [202]	Mabry, 2016 [7]	Arabic	Unclear [196]§	Unclear [196]§	Unclear [196]§
>3 hrs watching TV, video games, computers	Questionnaire	To assess child's diet and exercise behavior and investigate its association with overweight/obesity	Algeria[123]	Sharara, 2018[9]	Arabic	No evidence	No evidence	No evidence

≥3 hrs TV time/d	Validated questionnaire on the frequency of intake of certain food items and lifestyle**	Developed to collect information on the dietary and lifestyle habits of adolescent girls in Saudi Arabia. Measures food frequency intake per week, and lifestyle habits (i.e. hours of watching TV per day, frequency of eating breakfast, level of physical activity etc.) [178]	Saudi Arabia [178]	Mabry, 2016 [7]	Arabic	Unclear [203] §	Unclear [203] §	Unclear [203] §
% TV viewing/Video games > 2 hrs/d	Questions related to TV viewing/VG use time**	Developed to assess whether or not there is a behavioral correlation between TV watching and video game playing among children in the UAE. Collects data on socio-demographics, medical history, and psychosocial variables. [179]	UAE [179]	Mabry, 2016 [7]	Arabic	No evidence	No evidence	No evidence
Mean hours of computer time/wk	ATLS	Developed to assess the lifestyle habits of Arab adolescents and to study the interrelationships between various lifestyle variables. Investigates the prevalence of physical and sedentary activity, obesity and overweight, and dietary habits. [186]	Oman [173]	Mabry, 2016 [7]	Arabic	Yes [150,186]	No evidence	Yes [150,186]
Mean screen time (hrs/d)	ATLS	Developed to assess the lifestyle habits of Arab adolescents and to study the interrelationships between various lifestyle variables. Investigates the prevalence of physical and sedentary activity, obesity and overweight, and dietary habits. [186]	Oman [173]	Mabry, 2016 [7]	Arabic	Yes [150,186]	No evidence	Yes [150,186]
% TV time ≥3 hrs/d	Pre-tested questionnaire**	Developed to assess perceived barriers to physical activity participation among high school students in Oman. Collects data in classrooms on socio-demographics, physical education classes in school, barriers to physical activity participation, patterns in participation, frequency, intensity and types of physical and sedentary activity. [180]	Oman [180]	Mabry, 2016 [7]	Arabic	No evidence	No evidence	No evidence
Mean hours of TV/Video/wk	Pilot-tested questionnaire**	Developed to assess the prevalence of obesity, overweight, and associated factors among school children. Collects information on socio-demographics, parental education, location of school, and frequency and forms of physical and sedentary activity in a normal week. [181]	Bahrain [181]	Mabry, 2016 [7]	Arabic	No evidence	No evidence	No evidence
% watch >2 hrs of TV/d	ATLS	Developed to assess the lifestyle habits of Arab adolescents and to study the interrelationships between various lifestyle variables. Investigates the prevalence of physical and sedentary activity, obesity and overweight, and dietary habits. [186]	Kuwait [174]	Mabry, 2016 [7]	Arabic	Yes [150,186]	No evidence	Yes [150,186]

% use computers >2 hrs/d	ATLS	Developed to assess the lifestyle habits of Arab adolescents and to study the interrelationships between various lifestyle variables. Investigates the prevalence of physical and sedentary activity, obesity and overweight, and dietary habits. [186]	Kuwait [174]	Mabry, 2016 [7]	Arabic	Yes [150,186]	No evidence	Yes [150,186]

Notes: The used PA definitions in the GPAQ, IPAQ, IPAQ short version are all equivalent. Energy expenditure of >0.3 MJ/day is equivalent to walking daily for half an hour at 5 km/h
Metabolic equivalent of task (MET): "A unit that represents the metabolic cost of physical activity. One MET is the rate of energy expenditure while sitting at rest, which, for most people approximates an oxygen uptake of 3.5 ml per kg per min. The energy expenditure of other activities is expressed in multiples of METs. For example, for the average adult, sitting and reading requires about 1.3 METs, strolling or walking slowly requires about 2.0 METs, and running at 5 miles per hour requires about 8.3 METS" [41].

Symbols:
*Validated to assess ambulatory activities using the number of steps and do note capture the pattern, the intensity or type of PA. Even if some studies have reported that the steps count could be equivalent to > 60 min moderate activity in youth [151 152-155], they don't all agree on the threshold steps count. The reason we consider this definition non-standard.
** information extracted from the original publication of the primary study
† The use of a validated questionnaire reported by the SR was not mentioned in the original study.
‡ The use of Nurses' Health Study II questionnaire reported by the SR was not mentioned in the original study.
¥ Validity refers to the appropriateness, meaningfulness, and usefulness of the questionnaire measure for a specific purpose. A questionnaire was considered validated if concepts and components of validity or reliability were evaluated and published. The use of a questionnaire in multiple studies was not considered as a proof of validity.
§ No evidence of a measurement tool validation in the original study and/or listed references.

Abbreviations:

PA	Physical activity
SR	Systematic review
MET	Metabolic Equivalent of task
ATLS	Arab teens lifestyle student questionnaire
GPAQ	Global Physical Activity Questionnaire
IPAQ	International Physical Activity Questionnaire
min	Minutes
Wk	week
MJ	Megajoule (1 MJ is equal to one million 106 joules)
d	day
d-1	per day
hrs	hours
VG	Video games
VPA	Vigorous Physical Activity
MPA	Moderate Physical Activity

REFERENCES

1 Moher, D., Liberati, A., Tetzlaff, J. & Altman, D. G. Preferred reporting items for systematic reviews and meta-analyses: the PRISMA statement. *J Clin Epidemiol* **62**, 1006-1012, doi:10.1016/j.jclinepi.2009.06.005 (2009).

2 Bougioukas, K. I., Liakos, A., Tsapas, A., Ntzani, E. & Haidich, A.-B. Preferred reporting items for overviews of systematic reviews including harms checklist: a pilot tool to be used for balanced reporting of benefits and harms. *Journal of Clinical Epidemiology* **93**, 9-24, doi:https://doi.org/10.1016/j.jclinepi.2017.10.002 (2018).

3 Sisson, S. B. & Katzmarzyk, P. T. International prevalence of physical activity in youth and adults. *Obesity reviews : an official journal of the International Association for the Study of Obesity* **9**, 606-614, doi:10.1111/j.1467-789X.2008.00506.x (2008).

4 Mabry, R. M., Reeves, M. M., Eakin, E. G. & Owen, N. Evidence of physical activity participation among men and women in the countries of the Gulf cooperation council: a review. *Obesity reviews : an official journal of the International Association for the Study of Obesity* **11**, 457-464, doi:10.1111/j.1467-789X.2009.00655.x (2010).

5 Ranasinghe, C. D., Ranasinghe, P., Jayawardena, R. & Misra, A. Physical activity patterns among South-Asian adults: a systematic review. *International Journal of Behavioral Nutrition and Physical Activity* **10**, 116, doi:10.1186/1479-5868-10-116 (2013).

6 Yammine, K. The prevalence of physical activity among the young population of UAE: a meta-analysis. *Perspectives in Public Health* **137**, 275-280, doi:10.1177/1757913916675388 (2016).

7 Mabry, R., Koohsari, M. J., Bull, F. & Owen, N. A systematic review of physical activity and sedentary behaviour research in the oil-producing countries of the Arabian Peninsula. *BMC Public Health* **16**, 1003, doi:10.1186/s12889-016-3642-4 (2016).

8 Al-Hazzaa, H. M. Physical inactivity in Saudi Arabia revisited: A systematic review of inactivity prevalence and perceived barriers to active living. *Int J Health Sci (Qassim)* **12**, 50-64 (2018).

9 Sharara, E., Akik, C., Ghattas, H. & Makhlouf Obermeyer, C. Physical inactivity, gender and culture in Arab countries: a systematic assessment of the literature. *BMC Public Health* **18**, 639, doi:10.1186/s12889-018-5472-z (2018).

10 Guthold, R., Cowan, M. J., Autenrieth, C. S., Kann, L. & Riley, L. M. Physical activity and sedentary behavior among schoolchildren: a 34-country comparison. *J Pediatr* **157**, 43-49 e41, doi:10.1016/j.jpeds.2010.01.019 (2010).

11 Guthold, R., Ono, T., Strong, K. L., Chatterji, S. & Morabia, A. Worldwide variability in physical inactivity a 51-country survey. *American journal of preventive medicine* **34**, 486-494, doi:10.1016/j.amepre.2008.02.013 (2008).

12 Al-Nozha, M. M. *et al.* Prevalence of physical activity and inactivity among Saudis aged 30-70 years. A population-based cross-sectional study. *Saudi medical journal* **28**, 559-568 (2007).

13 Amine, E. K. & Samy, M. Obesity among female university students in the United Arab Emirates. *J R Soc Health* **116**, 91-96 (1996).

14 Mehairi, A. E. *et al.* Metabolic syndrome among Emirati adolescents: a school-based study. *PLoS One* **8**, e56159, doi:10.1371/journal.pone.0056159 (2013).

15 Muhairi, S. J. *et al.* Vitamin D deficiency among healthy adolescents in Al Ain, United Arab Emirates. *BMC Public Health* **13**, 33, doi:10.1186/1471-2458-13-33 (2013).
16 Wasfi, A. S., El-Sherbiny, A. A., Gurashi, E. & Al Sayegh, F. U. Sport practice among private secondary-school students in Dubai in 2004. *East Mediterr Health J* **14**, 704-714 (2008).
17 Khuwaja, A. K. & Kadir, M. M. Gender differences and clustering pattern of behavioural risk factors for chronic non-communicable diseases: community-based study from a developing country. *Chronic Illn* **6**, 163-170, doi:10.1177/1742395309352255 (2010).
18 Al-Baghli, N. A. *et al.* Overweight and obesity in the eastern province of Saudi Arabia. *Saudi medical journal* **29**, 1319-1325 (2008).
19 Memish, Z. A. *et al.* Obesity and Associated Factors - Kingdom of Saudi Arabia, 2013. *Preventing Chronic Disease* **11**, E174, doi:10.5888/pcd11.140236 (2014).
20 Almurshed, K. S. (2009).
21 AlQuaiz, A. M. & Tayel, S. A. Barriers to a healthy lifestyle among patients attending primary care clinics at a university hospital in Riyadh. *Annals of Saudi medicine* **29**, 30-35, doi:10.4103/0256-4947.51818 (2009).
22 Al Senany, S. & Al Saif, A. Assessment of physical health status and quality of life among Saudi older adults. *J Phys Ther Sci* **27**, 1691-1695, doi:10.1589/jpts.27.1691 (2015).
23 Amin, T. T. *et al.* Physical activity and cancer prevention: awareness and meeting the recommendations among adult Saudis. *Asian Pac J Cancer Prev* **15**, 2597-2606, doi:10.7314/apjcp.2014.15.6.2597 (2014).
24 Garawi, F., Ploubidis, G. B., Devries, K., Al-Hamdan, N. & Uauy, R. Do routinely measured risk factors for obesity explain the sex gap in its prevalence? Observations from Saudi Arabia. *BMC public health* **15**, 254-254, doi:10.1186/s12889-015-1608-6 (2015).
25 WHO-STEPS Survey Saudi Arabia. World Heath Organization (WHO) Stepwise Approach to NCD Surveillance - Country-Specific Standard Report. (MOH, WHO & EMRO, Riyadh, Saudi Arabia, 2005).
26 Maatoug, J. *et al.* Clustering of risk factors with smoking habits among adults, Sousse, Tunisia. *Preventing chronic disease* **10**, E211-E211, doi:10.5888/pcd10.130075 (2013).
27 Abdulle, A. M. *et al.* Under-treatment and under diagnosis of hypertension: a serious problem in the United Arab Emirates. *BMC cardiovascular disorders* **6**, 24 (2006).
28 McIlvenny, S., DeGlume, A., Elewa, M., Fernandez, O. & Dormer, P. Factors associated with fatigue in a family medicine clinic in the United Arab Emirates. *Family Practice* **17**, 408-413 (2000).
29 Sabri, S. *et al.* Some risk factors for hypertension in the United Arab Emirates. *East Mediterr Health J* **10**, 610-619 (2004).
30 Ahmed, F., Waslien, C., Al-Sumaie, M. A., Prakash, P. & Allafi, A. Trends and risk factors of hyperglycemia and diabetes among Kuwaiti adults: National Nutrition Surveillance Data from 2002 to 2009. *BMC public health* **13**, 103 (2013).
31 Al Zenki, S. *et al.* High prevalence of metabolic syndrome among Kuwaiti adults—a wake-up call for public health intervention. *International journal of environmental research and public health* **9**, 1984-1996 (2012).

32 Alarouj, M., Bennakhi, A., Alnesef, Y., Sharifi, M. & Elkum, N. Diabetes and associated cardiovascular risk factors in the State of Kuwait: the first national survey. *International journal of clinical practice* **67**, 89-96 (2013).
33 Al-Isa, A. N., Campbell, J., Desapriya, E. & Wijesinghe, N. Social and Health Factors Associated with Physical Activity among Kuwaiti College Students. *J Obes* **2011**, 512363, doi:10.1155/2011/512363 (2011).
34 WHO-STEPS Survey Kuwait. EMAN: Eastern Mediterranean Approach for Control of Non Communicable Diseases, WHO STEPS Survey of Risk Factors for Chronic Non Communicable Diseases, Kuwait, 2014. (2015).
35 Al-Nakeeb, Y., Lyons, M., Dodd, L. J. & Al-Nuaim, A. An investigation into the lifestyle, health habits and risk factors of young adults. *Int J Environ Res Public Health* **12**, 4380-4394, doi:10.3390/ijerph120404380 (2015).
36 Haj Bakri A & Al-Thani A. Chronic Disease Risk Factor Surveillance: Qatar STEPS Report 2012. (Qatar, 2013).
37 Walke, H., Mokdad, A. H., Zindah, M. & Belbeisi, A. Peer Reviewed: Obesity and Diabetes in Jordan: Findings From the Behavioral Risk Factor Surveillance System, 2004. *Preventing chronic disease* **5** (2008).
38 Centers for Disease Control and Prevention. Prevalence of selected risk factors for chronic disease--Jordan, 2002. *MMWR. Morbidity and mortality weekly report* **52**, 1042 (2003).
39 Kulwicki, A. D. & Kepler, C. K. Assessment of cardiovascular risk factors among residents of a city in Jordan. *Journal of cultural diversity* **8**, 34 (2001).
40 Madanat, H. & Merrill, R. M. Motivational factors and stages of change for physical activity among college students in Amman, Jordan. *Promotion & education* **13**, 185-190 (2006).
41 WHO-STEPS Survey Jordan. WHO STEPS survey Jordan, 2007. (2007).
42 Nsour, M., Mahfoud, Z., Kanaan, M. N. & Balbeissi, A. Prevalence and predictors of nonfatal myocardial infarction in Jordan. *East Mediterr Health J* **14**, 818-830 (2008).
43 WHO-STEPS Survey Egypt. WHO STEPS survey, Egypt 2011-2012. (2012).
44 Abolfotouh, M. A., Bassiouni, F. A., Mounir, G. M. & Fayyad, R. Health-related lifestyles and risk behaviours among students living in Alexandria University Hostels. *East Mediterr Health J* **13**, 376-391 (2007).
45 Kamel, M. H., Abdulmajeed, A. A. & Ismail, S. E.-S. Risk factors of falls among elderly living in Urban Suez-Egypt. *Pan African medical journal* **14** (2013).
46 Mahfouz, E. M., Sadek, R. R., Abdel-Latief, W. M., Mosallem, F. & Hassan, E. E. The role of dietary and lifestyle factors in the development of colorectal cancer: case control study in Minia, Egypt. *Cent Eur J Public Health* **22**, 215-222 (2014).
47 WHO-STEPS Survey Iraq. Non Communicable Diseases Risk Factors STEPS Survey Iraq, 2015. (2015).
48 WHO-STEPS Survey Libya. WHO STEPS Survey Libya 2009. (2009).
49 WHO STEPS Survey Lebanon. WHO STEPS Chronic Disease Risk Factor Surveillance Lebanon, 2009. (2009).
50 Al-Tannir, M., Kobrosly, S., Itani, T., El-Rajab, M. & Tannir, S. Prevalence of physical activity among Lebanese adults: a cross-sectional study. *Journal of Physical Activity and Health* **6**, 315-320 (2009).

51 Musharrafieh, U. *et al.* Determinants of university students physical exercise: a study from Lebanon. *Int J Public Health* **53**, 208-213, doi:10.1007/s00038-008-7037-x (2008).
52 Tamim, H. *et al.* Tobacco use by university students, Lebanon, 2001. *Addiction* **98**, 933-939 (2003).
53 Farah, R. *et al.* Prevalence of stroke symptoms among stroke-free residents: first national data from Lebanon. *International Journal of Stroke* **10**, 83-88, doi:10.1111/ijs.12563 (2015).
54 Tohme, R., Jurjus, A. & Estephan, A. The prevalence of hypertension and its association with other cardiovascular disease risk factors in a representative sample of the Lebanese population. *Journal of human hypertension* **19**, 861 (2005).
55 WHO-STEPS Survey Palestine. Palestine STEPS Survey 2010-2011 Fact Sheet (2011).
56 Abdul-Rahim, H. F. *et al.* Obesity in a rural and an urban Palestinian West Bank population. *International journal of obesity and related metabolic disorders : journal of the International Association for the Study of Obesity* **27**, 140-146, doi:10.1038/sj.ijo.0802160 (2003).
57 Abu-Mourad, T. *et al.* Individual determinants of primary healthcare utilisation in Gaza Strip, Palestine. *Journal of Epidemiology & Community Health* **62**, 701-707 (2008).
58 WHO-STEPS Survey Algeria. WHO STEPS survey Algeria, 2003 Factsheet. (2003).
59 WHO-STEPS Survey Sudan. WHO STEPS Survey Sudan, 2005-06. (2006).
60 El Rhazi, K. *et al.* Prevalence of obesity and associated sociodemographic and lifestyle factors in Morocco. *Public Health Nutr* **14**, 160-167, doi:10.1017/s1368980010001825 (2011).
61 Najdi, A. *et al.* Correlates of physical activity in Morocco. *Preventive medicine* **52**, 355-357 (2011).
62 Hamadeh, R. R. & Musaiger, A. O. Lifestyle patterns in smokers and non-smokers in the state of Bahrain. *Nicotine & tobacco research* **2**, 65-69 (2000).
63 Al Ali, R., Rastam, S., Fouad, F. M., Mzayek, F. & Maziak, W. Modifiable cardiovascular risk factors among adults in Aleppo, Syria. *International journal of public health* **56**, 653-662 (2011).
64 Al-Hazzaa. Physical activity profile of college male students. *King Saud University Journal (Educational Sciences)*
2, 383-396 (1990).
65 Al-Gelban, K. S. Dietary habits and exercise practices among the students of a Saudi Teachers' Training College. *Saudi medical journal* **29**, 754-759 (2008).
66 Gawwad, E. S. A. Stages of change in physical activity, self efficacy and decisional balance among saudi university students. *Journal of family & community medicine* **15**, 107-115 (2008).
67 Khalaf, A. *et al.* Female university students' physical activity levels and associated factors--a cross-sectional study in southwestern Saudi Arabia. *Int J Environ Res Public Health* **10**, 3502-3517, doi:10.3390/ijerph10083502 (2013).
68 Awadalla, N. J. *et al.* Assessment of physical inactivity and perceived barriers to physical activity among health college students, south-western Saudi Arabia. *East Mediterr Health J* **20**, 596-604 (2014).
69 Majeed, F. Association of BMI with diet and physical activity of female medical students at the University of Dammam, Kingdom of Saudi Arabia. *Journal of Taibah University Medical Sciences* **10**, 188-196, doi:https://doi.org/10.1016/j.jtumed.2014.11.004 (2015).

70 Samara, A., Nistrup, A., Al-Rammah, T. Y. & Aro, A. R. Lack of facilities rather than sociocultural factors as the primary barrier to physical activity among female Saudi university students. *Int J Womens Health* **7**, 279-286, doi:10.2147/ijwh.S80680 (2015).

71 Allam, A. R., Taha, I. M., Al-Nozha, O. M. & Sultan, I. E. Nutritional and health status of medical students at a university in Northwestern Saudi Arabia. *Saudi medical journal* **33**, 1296-1303 (2012).

72 Al-Shahri, M. Z. & Al-Almaei, S. M. Promotion of physical exercise by primary health care physicians in Riyadh city. *Saudi medical journal* **19**, 67-69 (1998).

73 Al Alwan, I. *et al.* Prevalence of Self-reported Cardiovascular Risk Factors among Saudi Physicians: A Comparative Study. *Int J Health Sci (Qassim)* **7**, 3-13, doi:10.12816/0006015 (2013).

74 AlAteeq, M. A. & AlArawi, S. M. Healthy lifestyle among primary health care professionals. *Saudi medical journal* **35**, 488-494 (2014).

75 Gharaibeh, M., Al-Ma'aitah, R. & Al Jada, N. Lifestyle practices of Jordanian pregnant women. *Int Nurs Rev* **52**, 92-100, doi:10.1111/j.1466-7657.2005.00257.x (2005).

76 AboZaid, H. A. & Farahat, F. M. Physical activity profile among patients attending family medicine clinics in western Saudi Arabia. *Saudi medical journal* **31**, 428-433 (2010).

77 Amin, T. T., Suleman, W., Ali, A., Gamal, A. & Al Wehedy, A. Pattern, prevalence, and perceived personal barriers toward physical activity among adult Saudis in Al-Hassa, KSA. *J Phys Act Health* **8**, 775-784, doi:10.1123/jpah.8.6.775 (2011).

78 Khalid, M. E. M. The association between strenuous physical activity and obesity in high and low altitude populations in southern Saudi Arabia. *International journal of obesity and related metabolic disorders : journal of the International Association for the Study of Obesity* **19**, 776-780 (1995).

79 Bin Horaib, G. *et al.* Prevalence of obesity among military personnel in Saudi Arabia and associated risk factors. *Saudi medical journal* **34**, 401-407 (2013).

80 Al-Rafaee, S. A. & Al-Hazzaa, H. M. Physical activity profile of adult males in Riyadh City. *Saudi medical journal* **22**, 784-789 (2001).

81 Al-Hazzaa, H. M. Health-enhancing physical activity among Saudi adults using the International Physical Activity Questionnaire (IPAQ). *Public Health Nutr* **10**, 59-64, doi:10.1017/S1368980007184299 (2007).

82 Amin, T. T., Al Sultan, A. I., Mostafa, O. A., Darwish, A. A. & Al-Naboli, M. R. Profile of non-communicable disease risk factors among employees at a Saudi university. *Asian Pac J Cancer Prev* **15**, 7897-7907, doi:10.7314/apjcp.2014.15.18.7897 (2014).

83 Al-Zalabani, A. H., Al-Hamdan, N. A. & Saeed, A. A. The prevalence of physical activity and its socioeconomic correlates in Kingdom of Saudi Arabia: A cross-sectional population-based national survey. *Journal of Taibah University Medical Sciences* **10**, 208-215, doi:https://doi.org/10.1016/j.jtumed.2014.11.001 (2015).

84 El Bcheraoui, C. *et al.* On Your Mark, Get Set, Go: Levels of Physical Activity in the Kingdom of Saudi Arabia, 2013. *J Phys Act Health* **13**, 231-238, doi:10.1123/jpah.2014-0601 (2016).

85 Albawardi, N. M., Jradi, H. & Al-Hazzaa, H. M. Levels and correlates of physical activity, inactivity and body mass index among Saudi women working in office jobs in Riyadh city. *BMC Womens Health* **16**, 33, doi:10.1186/s12905-016-0312-8 (2016).

86 Mandil, A. M., Alfurayh, N. A., Aljebreen, M. A. & Aldukhi, S. A. Physical activity and major non-communicable diseases among physicians in Central Saudi Arabia. *Saudi medical journal* **37**, 1243-1250, doi:10.15537/smj.2016.11.16268 (2016).
87 Al-Mountashiri, N. A., Al-Zhrani, A. M., Ibrahim, S. F. H. & Mirghani, H. O. Dietary habits, physical activity and diabetes perception among patients with type 2 diabetes mellitus in Tabuk City, Saudi Arabia. *Electron Physician* **9**, 5179-5184, doi:10.19082/5179 (2017).
88 AlBuhairan, F. S. *et al.* Time for an adolescent health surveillance system in Saudi Arabia: findings from "Jeeluna". *Journal of Adolescent Health* **57**, 263-269 (2015).
89 Al-Muhaimeed, A. A., Dandash, K., Ismail, M. S. & Saquib, N. Prevalence and correlates of overweight status among Saudi school children. *Annals of Saudi medicine* **35**, 275-281 (2015).
90 Al-Mutairi, R. L., Bawazir, A. A., Ahmed, A. E. & Jradi, H. Health beliefs related to diabetes mellitus prevention among adolescents in Saudi Arabia. *Sultan Qaboos University Medical Journal* **15**, e398 (2015).
91 Al-Othman, A. *et al.* Effect of physical activity and sun exposure on vitamin D status of Saudi children and adolescents. *BMC pediatrics* **12**, 92 (2012).
92 Mahfouz, A. A. *et al.* Nutrition, physical activity, and gender risks for adolescent obesity in Southwestern Saudi Arabia. *Saudi J Gastroenterol* **17**, 318-322, doi:10.4103/1319-3767.84486 (2011).
93 Organisation, W. H. Global School-based Student Health Survey United Arab Emirates, 2010 Factsheet. (2010).
94 GSHS UAE. Global School-based Student Health Survey United Arab Emirates, 2010 Factsheet. (2010).
95 GSHS Oman. Global School-based Student Health Survey, Oman 2015, Factsheet. (2015).
96 Afifi, M. Positive health practices and depressive symptoms among high school adolescents in Oman. *Singapore medical journal* **47**, 960 (2006).
97 Musaiger, A. O., Al-Roomi, K. & Bader, Z. Social, dietary and lifestyle factors associated with obesity among Bahraini adolescents. *Appetite* **73**, 197-204 (2014).
98 GSHS Kuwait. Global School-based Student Health Survey Kuwait 2015 Fact Sheet. (2015).
99 Shehab, D. K. & Al-Jarallah, K. F. Nonspecific low-back pain in Kuwaiti children and adolescents: associated factors. *Journal of adolescent health* **36**, 32-35 (2005).
100 GSHS Libya. Global School-based Student Health Survey Libya 2007 Fact Sheet (2007).
101 GSHS Djibouti. Global School-based Student Health Survey Djibouti 2007 Country Report. (2007).
102 GSHS Jordan. Global Schoolbased Student Health Survey Jordan Country Report, 2007. (2007).
103 Haddad, L. G., Owies, A. & Mansour, A. Wellness appraisal among adolescents in Jordan: a model from a developing country: a cross-sectional questionnaire survey. *Health Promot Int* **24**, 130-139, doi:10.1093/heapro/dap013 (2009).
104 GSHS Morocco. Global School-based Student Health Survey Morocco 2010 Fact Sheet (2010).
105 GSHS Egypt. Global School-based Student Health Survey Egypt 2006 Fact Sheet (2006).

106 Salazar-Martinez, E. *et al.* Overweight and obesity status among adolescents from Mexico and Egypt. *Arch Med Res* **37**, 535-542, doi:10.1016/j.arcmed.2005.10.014 (2006).
107 Shady, M. M. A., Youssef, M. M., Shehata, M. A., El-Din, E. M. S. & ElMalt, H. A. Association of serum 25-hydroxyvitamin D with life style and dietary factors in Egyptian prepubescent children. *Open access Macedonian journal of medical sciences* **3**, 80 (2015).
108 GSHS Lebanon. Global School-based Student Health Survey Lebanon 2011 Fact Sheet (2011).
109 Nasreddine, L. *et al.* Dietary, lifestyle and socio-economic correlates of overweight, obesity and central adiposity in Lebanese children and adolescents. *Nutrients* **6**, 1038-1062 (2014).
110 GSHS Iraq. Global School-based Student Health Survey Iraq 2012 Fact Sheet (2012).
111 GSHS oPt Gaza. Global School-based Student Health Survey oPt Gaza 2010 Fact Sheet (2010).
112 GSHS oPt West Bank. Global School-based Student Health Survey oPt West Bank 2010 Fact Sheet (2010).
113 Al Sabbah, H., Vereecken, C., Kolsteren, P., Abdeen, Z. & Maes, L. Food habits and physical activity patterns among Palestinian adolescents: findings from the national study of Palestinian schoolchildren (HBSC-WBG2004). *Public health nutrition* **10**, 739-746 (2007).
114 Jildeh, C. *et al.* Assessing the nutritional status of Palestinian adolescents from East Jerusalem: a school-based study 2002–03. *Journal of tropical pediatrics* **57**, 51-58 (2010).
115 GSHS Qatar. Global School-based Student Health Survey Qatar 2011 Fact Sheet (2011).
116 GSHS Sudan. Global School-based Student Health Survey Sudan 2012 Factsheet. (2012).
117 Moukhyer, M., Van Eijk, J., De Vries, N. & Bosma, H. Health-related behaviors of Sudanese adolescents. *Education for health* **21**, 184 (2008).
118 GSHS Syria. Global School-based Student Health Survey Syria 2010 Fact Sheet (2010).
119 GSHS Yemen. Global School-based Student Health Survey Yemen 2008 Fact Sheet (2008).
120 GSHS Tunisia. Global School-based Student Health Survey Tunisia 2008 Fact Sheet (2008).
121 Nouira, A. *et al.* Clustering of risk factors in the smoking habits of schoolchildren in Sousse, Tunisia. *International journal of adolescent medicine and health* **26**, 267-273 (2014).
122 Aounallah-Skhiri, H. *et al.* Blood pressure and associated factors in a North African adolescent population. a national cross-sectional study in Tunisia. *BMC Public Health* **12**, 98 (2012).
123 Abbes, M. A. & Bereksi-Reguig, K. Risk factors for obesity among school aged children in western Algeria: results of a study conducted on 293 subjects. *La Tunisie medicale* **94**, 23-28 (2016).
124 Al-Hazzaa, H. M., Abahussain, N. A., Al-Sobayel, H. I., Qahwaji, D. M. & Musaiger, A. O. Physical activity, sedentary behaviors and dietary habits among Saudi adolescents relative to age, gender and region. *International Journal of Behavioral Nutrition and Physical Activity* **8**, 140 (2011).

125 Al-Hazzaa, H. M. & Al-Rasheedi, A. A. Adiposity and physical activity levels among preschool children in Jeddah, Saudi Arabia. *Saudi medical journal* **28**, 766-773 (2007).
126 Al-Hazzaa, H., Sulaiman, M., Al-Mobairaik, K. & Al-Attas, O. Prevalence Of Coronary Artery Disease Risk Factors In Saudi Children. *Journal of the Saudi Heart Association* **5**, 126-133 (1993).
127 Al-Hazzaa, H. M. Physical activity, fitness and fatness among Saudi children and adolescents: implications for cardiovascular health. *Saudi medical journal* **23**, 144-150 (2002).
128 Al-Hazzaa, H. M. Pedometer-determined physical activity among obese and non-obese 8- to 12-year-old Saudi schoolboys. *J Physiol Anthropol* **26**, 459-465 (2007).
129 Al-Kutbe, R., Payne, A., de Looy, A. & Rees, G. A. A comparison of nutritional intake and daily physical activity of girls aged 8-11 years old in Makkah, Saudi Arabia according to weight status. *BMC Public Health* **17**, 592, doi:10.1186/s12889-017-4506-2 (2017).
130 Al-Rukban, M. O. Obesity among Saudi male adolescents in Riyadh, Saudi Arabia. *Saudi medical journal* **24**, 27-33 (2003).
131 Taha, A. Z. Self-reported knowledge and pattern of physical activity among school students in Al Khobar, Saudi Arabia. *East Mediterr Health J* **14**, 344-355 (2008).
132 Mahfouz, A. A., Shatoor, A. S., Hassanein, M. A., Mohamed, A. & Farheen, A. Gender differences in cardiovascular risk factors among adolescents in Aseer Region, southwestern Saudi Arabia. *J Saudi Heart Assoc* **24**, 61-67, doi:10.1016/j.jsha.2011.09.002 (2012).
133 Alzahrani, S. G., Watt, R. G., Sheiham, A., Aresu, M. & Tsakos, G. Patterns of clustering of six health-compromising behaviours in Saudi adolescents. *BMC Public Health* **14**, 1215, doi:10.1186/1471-2458-14-1215 (2014).
134 Alsubaie, A. S. R. & Omer, E. O. M. Physical Activity Behavior Predictors, Reasons and Barriers among Male Adolescents in Riyadh, Saudi Arabia: Evidence for Obesogenic Environment. *Int J Health Sci (Qassim)* **9**, 400-408 (2015).
135 Bajamal, E. *et al.* Physical Activity Among Female Adolescents in Jeddah, Saudi Arabia: A Health Promotion Model-Based Path Analysis. *Nurs Res* **66**, 473-482, doi:10.1097/nnr.0000000000000244 (2017).
136 Al-Raddadi, R., Bahijri, S., Borai, A. & AlRaddadi, Z. Prevalence of lifestyle practices that might affect bone health in relation to vitamin D status among female Saudi adolescents. *Nutrition* **45**, 108-113, doi:10.1016/j.nut.2017.07.015 (2018).
137 Bener, A. *et al.* The prevalence of hypertension and its associated risk factors in a newly developed country. *Saudi medical journal* **25**, 918-922 (2004).
138 Al-Mahroos, F. & Al-Roomi, K. Obesity among adult Bahraini population: impact of physical activity and educational level. *Ann Saudi Med* **21**, 183-187 (2001).
139 Al-Mahroos, F. & McKeigue, P. M. High prevalence of diabetes in Bahrainis. Associations with ethnicity and raised plasma cholesterol. *Diabetes Care* **21**, 936-942 (1998).
140 Al-Thani, M. *et al.* Lifestyle Patterns Are Associated with Elevated Blood Pressure among Qatari Women of Reproductive Age: A Cross-Sectional National Study. *Nutrients* **7**, 7593-7615, doi:10.3390/nu7095355 (2015).
141 WHO-STEPS Survey Qatar. WHO Qatar STEPWISE Report 2012 chronic disease risk factor surveillance (Doha, Qatar, 2013).

142 Taha, A. Z. & Bella, H., . Heart disease risk factors: prevalence and knowledge in a primary care setting, Saudi Arabia. *Eastern Mediterranean Healrth Journal* **4**, 293-300 (1998).

143 Al-Hazzaa, H. M. *et al.* Patterns and determinants of physical activity among Saudi adolescents. *J Phys Act Health* **11**, 1202-1211, doi:10.1123/jpah.2012-0427 (2014).

144 Al-Hazzaa, H. M. *et al.* Association of dietary habits with levels of physical activity and screen time among adolescents living in Saudi Arabia. *J Hum Nutr Diet* **27 Suppl 2**, 204-213, doi:10.1111/jhn.12147 (2013).

145 Al-Hazzaa, H. M. *et al.* A cross-cultural comparison of health behaviors between Saudi and British adolescents living in urban areas: gender by country analyses. *Int J Environ Res Public Health* **10**, 6701-6720, doi:10.3390/ijerph10126701 (2013).

146 Al-Nakeeb, Y. *et al.* Obesity, physical activity and sedentary behavior amongst British and Saudi youth: a cross-cultural study. *Int J Environ Res Public Health* **9**, 1490-1506, doi:10.3390/ijerph9041490 (2012).

147 Al-Nuaim, A. A. *et al.* The Prevalence of Physical Activity and Sedentary Behaviours Relative to Obesity among Adolescents from Al-Ahsa, Saudi Arabia: Rural versus Urban Variations. *J Nutr Metab* **2012**, 417589, doi:10.1155/2012/417589 (2012).

148 *International Physical Activity Questionnaire*, <https://docs.google.com/viewer?a=v&pid=sites&srcid=ZGVmYXVsdGRvbWFpbnx0aGVpcGFxfGd4OjE0NDgxMDk3NDU1YWRlZTM> (2005).

149 Graf, C. *et al.* Feasibility and acceptance of exercise recommendations (10,000 steps a day) within routine German health check (Check-Up 35/GOÄ29)—study protocol. *Pilot and Feasibility Studies* **2**, 52, doi:10.1186/s40814-016-0092-9 (2016).

150 Al-Hazzaa, H. M., Al-Sobayel, H. I. & Musaiger, A. O. Convergent Validity of the Arab Teens Lifestyle Study (ATLS) Physical Activity Questionnaire. *International Journal of Environmental Research and Public Health* **8**, 3810 (2011).

151 Rowlands, A. V. & Eston, R. G. Comparison of accelerometer and pedometer measures of physical activity in boys and girls, ages 8-10 years. *Research quarterly for exercise and sport* **76**, 251-257, doi:10.1080/02701367.2005.10599296 (2005).

152 McNamara, E., Hudson, Z. & Taylor, S. J. C. Measuring activity levels of young people: the validity of pedometers. *British Medical Bulletin* **95**, 121-137, doi:10.1093/bmb/ldq016 (2010).

153 Beets, M. *et al. Convergent Validity of Pedometer and Accelerometer Estimates of Moderate-to-Vigorous Physical Activity of Youth*. Vol. 8 Suppl 2 (2011).

154 Sallis, J. F. Measuring Physical Activity: Practical Approaches for Program Evaluation in Native American Communities. *Journal of public health management and practice : JPHMP* **16**, 404-410, doi:10.1097/PHH.0b013e3181d52804 (2010).

155 Tudor-Locke, C., Williams, J. E., Reis, J. P. & Pluto, D. Utility of pedometers for assessing physical activity: convergent validity. *Sports Med* **32**, 795-808, doi:10.2165/00007256-200232120-00004 (2002).

156 Human energy requirements. Scientific background papers from the Joint FAO/WHO/UNU Expert Consultation. October 17-24, 2001. . Report No. 1368-9800 (Print)
1368-9800, 929-1228 (Rome, Italy, 2005).

157 World Health Organization (WHO). in *Global Recommendations on Physical Activity for Health* (World Health Organization 2010, Geneva, 2010).

158 Pate, R. R., Pratt, M., Blair, S. N. & et al. Physical activity and public health: A recommendation from the centers for disease control and prevention and the american college of sports medicine. *JAMA* **273**, 402-407, doi:10.1001/jama.1995.03520290054029 (1995).
159 World Health Organization (WHO). in *Working version 5* (Geneva 2014).
160 World Health Organization (WHO). *Global Physical Activity Questionnaire (GPAQ): Analysis Guide* <http://www.who.int/ncds/surveillance/steps/resources/GPAQ_Analysis_Guide.pdf> (n.d.).
161 González, K., Fuentes, J. & Márquez, J. L. Physical Inactivity, Sedentary Behavior and Chronic Diseases. *Korean Journal of Family Medicine* **38**, 111-115, doi:10.4082/kjfm.2017.38.3.111 (2017).
162 Sedentary Behaviour Research, N. Letter to the Editor: Standardized use of the terms "sedentary" and "sedentary behaviours". *Applied Physiology, Nutrition, and Metabolism* **37**, 540-542, doi:10.1139/h2012-024 (2012).
163 Tremblay, M. S. *et al.* Sedentary Behavior Research Network (SBRN) – Terminology Consensus Project process and outcome. *International Journal of Behavioral Nutrition and Physical Activity* **14**, 75, doi:10.1186/s12966-017-0525-8 (2017).
164 National Institutes of Health: Division of Cancer Control and Population Sciences. *List of physical activity standard questionnaires validations studies*, <https://epi.grants.cancer.gov/paq/validation.html> (
165 Shamliyan, T. A. *et al.* in *Development of Quality Criteria To Evaluate Nontherapeutic Studies of Incidence, Prevalence, or Risk Factors of Chronic Diseases: Pilot Study of New Checklists* (Agency for Healthcare Research and Quality (US), 2011).
166 Banday, A. H., Want, F. A., Alris, F. F., Alrayes, M. F. & Alenzi, M. J. A Cross-sectional Study on the Prevalence of Physical Activity Among Primary Health Care Physicians in Aljouf Region of Saudi Arabia. *Mater Sociomed* **27**, 263-266, doi:10.5455/msm.2015.27.263-266 (2015).
167 Koura, M. R., Al-Dabal, B. K., Rasheed, P., Al-Sowielem, L. S. & Makki, S. M. Prehypertension among young adult females in Dammam, Saudi Arabia. *East Mediterr Health J* **18**, 728-734 (2012).
168 Ministry of Health United Arab Emirates. World Health Survey Results, Report of United Arab Emirates. (World Health Organization (WHO), Abu Dhabi, UAE, 2003).
169 Carter, A. O., Elzubeir, M., Abdulrazzaq, Y. M., Revel, A. D. & Townsend, A. Health and lifestyle needs assessment of medical students in the United Arab Emirates. *Med Teach* **25**, 492-496, doi:10.1080/0142159031000160563 3 (2003).
170 El-Aty, M. A. *et al.* Metabolic Syndrome and Its Components: Secondary analysis of the World Health Survey, Oman. *Sultan Qaboos Univ Med J* **14**, e460-467 (2014).
171 WHO-STEPS Survey Kuwait. World Health Organization (WHO) Stepwise Approach to NCD Surveillance, Kuwait. (MOH & WHO, Kuwait City, Kuwait, 2008).
172 Al-Hazzaa, H. M., Abahussain, N. A., Al-Sobayel, H. I., Qahwaji, D. M. & Musaiger, A. O. Physical activity, sedentary behaviors and dietary habits among Saudi adolescents relative to age, gender and region. *Int J Behav Nutr Phys Act* **8**, 140, doi:10.1186/1479-5868-8-140 (2011).

173 Kilani, H., Al-Hazzaa, H., Waly, M. I. & Musaiger, A. Lifestyle Habits: Diet, physical activity and sleep duration among Omani adolescents. *Sultan Qaboos Univ Med J* **13**, 510-519 (2013).
174 Allafi, A. *et al.* Physical activity, sedentary behaviours and dietary habits among Kuwaiti adolescents: gender differences. *Public Health Nutr* **17**, 2045-2052, doi:10.1017/S1368980013002218 (2013).
175 Cleland, C. L. *et al.* Validity of the Global Physical Activity Questionnaire (GPAQ) in assessing levels and change in moderate-vigorous physical activity and sedentary behaviour. *BMC Public Health* **14**, 1255, doi:10.1186/1471-2458-14-1255 (2014).
176 Mabry, R. M., Winkler, E. A., Reeves, M. M., Eakin, E. G. & Owen, N. Correlates of Omani adults' physical inactivity and sitting time. *Public Health Nutr* **16**, 65-72, doi:10.1017/s1368980012002844 (2013).
177 Farghaly, N. F., Ghazali, B. M., Al-Wabel, H. M., Sadek, A. A. & Abbag, F. I. Life style and nutrition and their impact on health of Saudi school students in Abha, Southwestern region of Saudi Arabia. *Saudi medical journal* **28**, 415-421 (2007).
178 Musaiger, A. O. & Zagzoog, N. Dietary and lifestyle habits among adolescent girls in Saudi Arabia. *Nutrition & Food Science* **43**, 605-610 (2013).
179 Yousef, S., Eapen, V., Zoubeidi, T. & Mabrouk, A. Behavioral correlation with television watching and videogame playing among children in the United Arab Emirates. *Int J Psychiatry Clin Pract* **18**, 203-207, doi:10.3109/13651501.2013.874442 (2013).
180 Youssef, R. M., Al Shafie, K., Al-Mukhaini, M. & Al-Balushi, H. Physical activity and perceived barriers among high-school students in Muscat, Oman. *East Mediterr Health J* **19**, 759-768 (2013).
181 Gharib, N. M. & Rasheed, P. Obesity Among Bahraini Children and Adolescents: Prevalence And Associated Factors. *Journal of the Bahrain Medical Society* **20** (2008).
182 Chaabna, K. *et al.* Gray literature in systematic reviews on population health in the Middle East and North Africa: protocol of an overview of systematic reviews and evidence mapping. *Systematic Reviews* **7**, 94, doi:10.1186/s13643-018-0751-4 (2018).
183 World Health Organization (WHO). *Global Physical Activity Surveillance*, <http://www.who.int/ncds/surveillance/steps/GPAQ/en/> (2018).
184 International Physical Activity Questionnaire (IPAQ). Guidelines for Data Processing and Analysis of the International Physical Activity Questionnaire (IPAQ) – Short and Long Forms. (IPAQ, 2005).
185 International Physical Activity Questionnaire (IPAQ). *Cultural Adaptation*, <https://sites.google.com/site/theipaq/cultural-adaptation> (n.d.).
186 Al-Hazzaa, H. M., Musaiger, A. O. & Group, A. R. Arab Teens Lifestyle Study (ATLS): objectives, design, methodology and implications. *Diabetes Metab Syndr Obes* **4**, 417-426, doi:10.2147/DMSO.S26676 (2011).
187 Wolf, A. M. *et al.* Reproducibility and validity of a self-administered physical activity questionnaire. *Int J Epidemiol* **23**, 991-999 (1994).
188 World Health Organization (WHO). WHO Study Group on Diabetes Mellitus. Diabetes mellitus: report of a WHO study group. World Health Organization Technical Report Series 727. Geneva: World Health Organization, 1985. (1985).
189 Prochaska, J. J., Sallis, J. F. & Long, B. A physical activity screening measure for use with adolescents in primary care. *Arch Pediatr Adolesc Med* **155**, 554-559 (2001).

190 Al-Ahmadi, M. & Al-Hazzaa, H. Validity of a self-reported questionnaire for youth 15–25 years: Comparison with accelerometer, pedometer and heart rate telemetry. *Saudi Sports Medicine Journal* **7**, 2-14 (2004).
191 Al-Hazzaa, H. & Al-Ahmadi, M. A self-reported questionnaire for the assessment of physical activity in youth 15–25 years: Development, reliability and construct validity. *Arab J of Food & Nutrition* **4**, 279-291 (2003).
192 Health promotion research center. *The Rapid Assessment of Physical Activity (RAPA) questionnaire*, <https://depts.washington.edu/hprc/resources/products-tools/rapa/> (
193 Centers for Disease Control and Prevention: National Center for Cahronic Disease Prevention and Health Promotion. The Behavioral Risk Factor Surveillance System (BRFSS) questionnaire. (U.S 2018).
194 Ainsworth, B. E., Jacobs, D. R., Jr. & Leon, A. S. Validity and reliability of self-reported physical activity status: the Lipid Research Clinics questionnaire. *Med Sci Sports Exerc* **25**, 92-98, doi:10.1249/00005768-199301000-00013 (1993).
195 El Ati, J. *et al.* Development, reproducibility and validity of a physical activity frequency questionnaire in North Africa. *Arab J Food Nutrition* **5**, 148-167 (2004).
196 Abalkhail, B., Shawky, S. & Ghabrah, T. Perception of body weight among saudi school children. *Journal of family & community medicine* **9**, 35-49 (2002).
197 Inchley J, C. D., Cosma A & Samdal O. Survey Methods. Health Behaviour In School-Aged Children (HBSC Study Protocol): Background, Methodology And Mandatory Study Items for the 2017/2018 Survey.
198 Booth, M. L., Chey, T. & Bauman, A. The reliability and validity of the physical activity questions in the WHO health behaviour in schoolchildren (HBSC) survey: A population study. *British journal of sports medicine* **35**, 263-267, doi:10.1136/bjsm.35.4.263 (2001).
199 Musaiger, A. O., Bader, Z., Al-Roomi, K. & D'Souza, R. Dietary and lifestyle habits amongst adolescents in Bahrain. *Food Nutr Res* **55**, doi:10.3402/fnr.v55i0.7122 (2011).
200 Froberg, K., Brage, S., Wedderkopp, N. & Andersen, L. Influence of Step Frequency on Movement Intensity Predictions With the CSA Accelerometer: A Field Validation Study in Children. *Medicine and Science in Sports and Exercise - MED SCI SPORT EXERCISE* **35**, doi:10.1097/00005768-200305001-01583 (2003).
201 Centers for Disease Control and Prevention National Center for Chronic Disease Prevention and Health Promotion. *Target Heart Rate and Estimated Maximum Heart Rate*.
202 Mahfouz, A. A. *et al.* Obesity and related behaviors among adolescent school boys in Abha City, Southwestern Saudi Arabia. *J Trop Pediatr* **54**, 120-124, doi:10.1093/tropej/fmm089 (2008).
203 Musaiger, A. O. & Radwan, H. M. Social and dietary factors associated with obesity in university female students in United Arab Emirates. *J R Soc Health* **115**, 96-99 (1995).
